# Newton’s cradle philosophy and stress relief or what goes up must come down

**DOI:** 10.17879/freeneuropathology-2026-10294

**Published:** 2026-10-21

**Authors:** Isidro Ferrer

**Affiliations:** 1 Emeritus Professor, University of Barcelona, Spain

**Keywords:** Neuropathology, History of Medicine, Brain banking, Neurodegeneration, Medical education, Bellvitge University Hospital, Autobiography

When I received the kind invitation to share my thoughts for *Free Neuropathology*, I had just returned from a visit to part of my family who live abroad. Madagascar, which was the destination of my trip, is an incredibly stunning country, with a wonderful variety of coasts, mountains, valleys, and plains, as well as unique plants and animals. Despite its land's riches, many people live in poverty, sometimes miserably. We visited a foundation based in Barcelona that shelters children and families in need, located in a village near the capital, Antananarivo, and managed by local agents. Back home, I was still thinking about the trip and the plan to increase the number of associate members and the foundation's donations.

As I write this in 2026, I am 75 years old. I stepped down from my position as neuropathologist in the Pathology Department and as head of the Institute of Neuropathology at Bellvitge University Hospital in Hospitalet de Llobregat, Barcelona, when I turned 65 in 2016. Five years later, in 2021, I retired as a professor in the Department of Pathology and Experimental Therapeutics and became a professor emeritus at the University of Barcelona.

Recently, I have spent time writing fiction. This activity is remarkably engrossing. In contrast to scientific writing, fiction allows for the liberty to create situations, characters, and scenes as the story unfolds. The characters confront their own challenges, inhabit settings that require depiction, and possess interests and aspirations that need anchoring. They embark on journeys and undergo adventures that are either imaginary or reflective of the author's experiences. Writing fiction ignites a curiosity about how the plot will progress and where it will lead. I'm not claiming that my writing is exceptional (it likely has flaws), but the enjoyment it brings is gratifying.

Retirement has allowed me to indulge in activities I can now enjoy with a slower, more fulfilling approach. While these are not solitary pursuits, I do appreciate having time alone to read, walk, or swim. I cherish the company of my family, even though some members live far away, and I enjoy spending moments with my friends. I prefer to avoid social gatherings or large functions; I have never felt at ease in such settings since childhood.

Currently residing in Barcelona, I acquired a grove of ancient olive trees along with a small house. The land also features carob trees, almond trees, and a few fig trees, all cultivated in dry stone terraces using rain-fed methods. From there, one can view the Mediterranean Sea and the Ebro river delta in the distance. I have planted around a hundred new trees along the front edges of the field and at the entrance: these include cypress, pine, mimosa, and several resilient fruit trees (**Figure 1**). So far, over fifty of them have taken root successfully. I'm not trying to present a romantic or idyllic picture; it's breathtaking but requires a tremendous deal of work.

**Figure 1:**
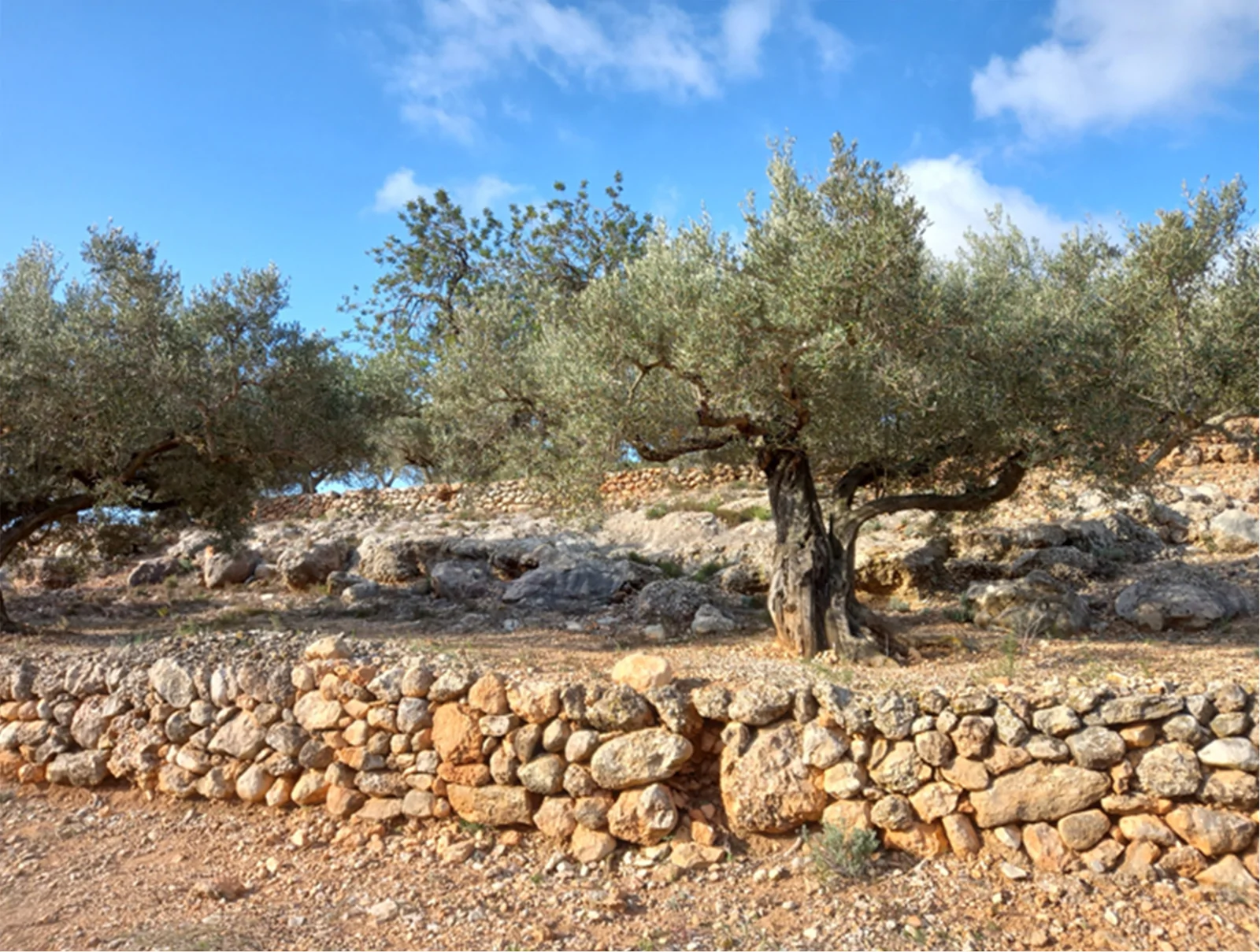
**Figure 1.** The author's olive grove near Tortosa, Tarragona.

Receiving the invitation to share my reflections in *Free Neuropathology* was a surprise, and it filled me with gratitude. It was an honor, but I wasn't entirely certain what I could contribute. The Reflections series aims to explain, without boring, the experiences of senior neuropathologists, their efforts, fortunes, and adversities, as well as other circumstances and events they have encountered throughout their professional lives. It is prudent, in this case, to take into account Oscar Wilde's quotation: *"Man is least himself when he talks in his own person. Give him a mask, and he will tell you the truth."* Here, I am not wearing a mask. Nevertheless, I will balance my true memories, false memories, forgetfulness, and others' comments. While some dates may not be entirely accurate, I believe the final account is close to what happened.

Although this is the introduction of a reflection on my professional life, I cannot start without emphasizing the support of my family and my closest friends; their mutual love, respect, and care have been the foundation of our life for so many years.

## Family, childhood, and adolescence

My paternal grandmother was the sole heir to a farm with hectares of land where thousands of olive trees were cultivated and other areas with fruit orchards. She became an orphan at a very young age, married my grandfather, and settled in Barcelona. He had prominent, pointed mustaches and a stern expression, whereas my grandmother was small and blonde. She was very religious and monarchist, and he was an atheist and republican. My grandfather was the one in charge, while my grandmother obeyed him. The old photographs support my vague memories: they were an ordinary patriarchal couple, with family roles well established since ancient times.

They had two children. The eldest, who became my father, was recruited and sent to fight in the Civil War as part of the La Quinta del Biberón battalion (*age group of the baby bottle*) on the Republican side, without any training. The battle of the Ebro was a massacre in which thousands of young people died, often without understanding why they were fighting, whom they were killing, or why they were dying. My father fought there and survived, but the brutality he witnessed left him profoundly bewildered and sorrowful. He studied law in Barcelona, established his law practice there, and became an expert in business law. His younger brother, my uncle, was also a lawyer and worked in my father's office. He remained single and had no children.

My mother’s ancestors came from a town in Huesca that bears her surname, or perhaps, more appropriately, she had the surname of her hometown. Her family settled in Barcelona a few generations earlier, around the turn of the century. I know very little about my mother's family. I only know that they were moderately well-off, that their father died when they were very young, and that my grandmother would dress me in white shoes while sitting on her lap. My mother had an older brother whom I remember as an elegant man who wore an astrakhan hat and used a walking stick after suffering a stroke. My uncle was widowed at a young age, and his only daughter lived in our house for a long time. My cousin has always been like an older sister to me.

We were five siblings. We went to different private schools: the boys to boys' schools and my sister (the youngest) to girls' schools. My parents were avid readers, and my mother played the piano very well, but there came a time when the piano at home stopped playing. I never knew the reason; my mother was a perfectionist, and perhaps, with the housework, she didn't have the necessary time to maintain the level she desired. On several weekends, we would visit Romanesque churches with a family that was friends with my parents. My parents were practicing Catholics; we were baptized, but we went to secular schools.

During my first school years, I faced learning difficulties, particularly with reading and writing. Initially, I struggled to understand the sequence of letters and words. In primary school, my teacher made me write letters, lines, and sentences repeatedly until they appeared fluid and correct. I was eager to read and worked diligently for hours, often skipping some words to grasp the main concepts and extract meaning from sentences. Over time, I began to comprehend the texts better and started including the previously overlooked words. I made this progress on my own because reading challenges were never emphasized as much as writing ones at school or home. As I continued my education, my reading skills improved significantly, and I came to enjoy reading. However, my writing skills did not improve; in fact, they worsened. During my medical studies, I frequently had to take oral exams because my professors often struggled to read my handwriting, and occasionally, I had difficulty reading it myself. Even as a doctor, writing medical histories by hand became a considerable effort for me. To this day, I still have trouble understanding my handwritten notes. Years later, doctors discovered that those difficulties were called dyslexia and dysgraphia.

During secondary school, I learned quickly and developed a strong interest in natural sciences, history, art, and philosophy. The teachers who taught these subjects undoubtedly influenced my enthusiasm; they explained concepts in an engaging way and with genuine excitement. To fit in with my peers, I often acted out, which led to frequent punishments, including being slapped by the principal. At that time, it was common for teachers to physically discipline students without hesitation, as it was considered a normal part of education.

We had additional activities typical of a certain *bourgeoisie*, such as belonging to a sports club, summering in a coastal village, sailing, and horseback riding (only I was interested; my brothers played hockey). Yet we were in the scouts that represented a kind of opposition to the Franco regime. We occasionally received priests for weeks at home who were being pursued by the police for political reasons; we had to keep it a complete secret. I remember that my parents were involved in some social works that were perhaps prohibited or little tolerated by the regime; however, my siblings and I never received a clear explanation of what their activities were. I have often wondered how many Spaniards in the post-civil war period lived with this kind of hidden duality—almost a form of soft resistance against the regime.

My parents also sent us to France with host families, who in turn sent their children to our home. French was our second language in school, and this fact marked several generations until English became dominant.

Twice a week, starting at seven in the evening, I would go to a workshop for manual crafts, clay modeling, plaster work, and painting near my house. Soon I had problems with painting: I have dichromatopsia or congenital color blindness (my third brother also had it). I stopped painting; I only managed well with white and black. I became fond of photography and set up a mobile lab in the bathroom of my parents' house to develop films and make paper copies. I also spent entire afternoons making models of wooden boats and airplanes with balsa wood and paper, which I would then varnish and paint. I obtained the models from photographs. The last one was a biplane with a small gasoline engine that flew on an authentic model airplane runway.

I can't recall an exact date, but my relationship with my parents changed during my adolescence. I remember frequent disagreements and arguments on any topic, a lack of communication, and my desire for independence. They granted me legal emancipation shortly after I turned sixteen, but, as far as I remember, everything remained largely the same for several years. The only money I made came from printing and selling white T-shirts with logos and motifs with basic colors. I didn't start being independent until well into my studies at medical school; then almost everything changed.

I was the eldest brother in my family. My second brother became an economist, while my younger sister dedicated herself to childcare. My third and fourth brothers passed away at a young age. One was a lawyer like my father, and he died in a car accident; the other had a degree in agricultural engineering and suffered a heart attack while supervising crop optimization in Brazil. They left behind two and three small children, respectively. The premature deaths of my two brothers within a few years were devastating and had a lasting impact on me.

After the war, the estates of my grandmother lost ground due to frequent neighbourhood brawls and vendettas in our towns well into the 1950s, which were partly aggravated by my grandfather's position on the defeated side. Most of the lands had been lost. I remember, as a child, some summers going through the fields, playing with my second-oldest brother among the fruit trees, swimming in the river pools, and taking excursions in a horse-drawn farm wagon among the olive trees. After those early years, we never went there again, as we spent most of the summer at the beach. Finally, after my father's premature death, his brother, my paternal uncle, ended up selling what remained, and only the house was left, which was eventually sold.

As adults, my agricultural engineer brother and I considered purchasing a new olive grove in another location. He had a deep understanding of all types of plantations and had a true passion for the countryside. When my brother passed away, I decided to move forward with our project in his memory.

## Medical studies at the University of Barcelona

I entered the Faculty of Medicine at the University of Barcelona when I was 18 years old. I could have chosen to study art, history, or biology, but I decided on medicine because it felt like the best way to help people. I figured I could always pursue art later or in my free time. During my first year at university, I learned more than I could have imagined, especially considering the circumstances. We only had a month of regular classes, as protests and strikes disrupted most of the academic year. The protest movement within the faculties was overwhelming; we faced daily confrontations with the police, and at times we witnessed assaults at the clinical hospital near the faculty. Some students even donned white coats to confuse the police or feigned illness to evade beatings.

Politically, this environment distanced me from the comfortable life I had known until then. I started to avoid parties and clubs, distancing myself from most of my old friends. I participated in demonstrations, distributed propaganda, and attended assemblies, but the tone of indoctrination bothered me, and I didn't fit in with any radical group. I read extensively on various subjects, mainly sociology, philosophy, and politics. In that first year, I became excited about the ideas of the classical Stoics, Epicureans, and existentialists. Their thoughts have remained a constant influence throughout my life.

Despite numerous social contacts, I enjoyed spending a lot of time alone—thinking, walking, and occasionally riding horses in the mountains surrounding Barcelona. I knew how to ride and belonged to an equestrian club where, with permission, I took out the horses whose owners did not ride them. The horses needed to go out, and I volunteered as a stable hand in exchange for the opportunity to ride some amazing horses in the forest.

My ideas at that time were both clear and confusing, ecletic and contradictory. I had a great deal of energy, which I devoted both to social causes and to individual pursuits for my own enjoyment.

At the same time, I was studying anatomy, biochemistry, and histology, which were the main subjects of my first year. It was only at the end of the course that classes resumed, and exams took place. I completed the year. Overall, I felt like a misfit; I hadn't found my place yet. I moved eagerly in different directions but appeared disillusioned with the world. I was furiously angry without knowing the reason or how to resolve it, yet at the same time, I felt happy about the freedom and the possibilities of discovering new experiences.

That summer, I traveled alone, hitchhiking and staying in youth hostels or places where I was invited to spend the night. I explored southern and eastern France, parts of Switzerland, and Bavaria. On my journey back, I stopped in Paris, from where I took a train to Barcelona. This experience felt like a rite of passage, teaching me for the first time that normality is relative and that the unfamiliar is simply different. The toughest part was Germany. I didn't speak German, and the people there spoke little French, making communication challenging. English was not widely spoken, especially among older people. Our accents were suboptimal on both sides. Upon my return, I began to study English more intensely, although my accent still sounds foreign. My dyslexia didn't help with learning new phonemes or with writing and pronouncing words correctly.

The second, third, and fourth years of medical school were calmer at the faculty, although I attended very few theoretical classes. In the second year, I was an intern in the physiology department; in the following years, I attended rounds as an intern in two internal medicine departments at the Clinical Hospital. One night a week, I worked in the hospital's emergency room.

During my time at university, I taught physiology and biochemistry to students from the United States who had moved to Barcelona to study medicine to avoid being drafted into the Vietnam war. Some came with partners and, more rarely, with children. They had money and were well-mannered people, very attentive to explanations and fulfilling their duties. I imagined what that war must have been like, thousands of kilometers from home, in a country you knew nothing about and that had personally caused you no harm. My students did not seem like deserters to me at all. They had pacifist convictions: they did not agree with war –- any war. Their ideas and conversations with them and other Americans living in Barcelona steered me away from confrontation as a way to resolve conflicts. It was the first time I heard anything about peace movements. I felt a stronger connection to the beatnik movement and its continuation in the hippie movement. The sense of freedom, peace, and a reconsideration of old values appealed to me. I found we were on the same wavelength.

Private lessons proved financially providential, as they provided my first regular income; later, I continued teaching local students. I also edited notes on physiology and microbiology, which the student cooperative sold to students in the lower courses. I bought a small motorcycle and, later, a second-hand Citroën 2CV. I was more financially independent.

In the following years I hitchhiked again, but this time with companions. It was a cheap way to travel, and we had the opportunity to meet many different people. Generally, folks were kind and helpful. Conversations would often spark, sometimes even intense discussions, with strangers I knew nothing about, lasting throughout the journey. I never felt in danger or encountered any unpleasant surprises. In today's world, I wouldn't consider it for myself, nor would I recommend it to others.

In the second year of medicine, I had my first girlfriend, a girl who completely captivated me; we studied together, traveled together, and had similar ideas. After two years, she left me for a psychiatrist, leaving me utterly heartbroken.

## Finding neuropathology

Rather than following a path similar to that of young Werther, I devoted myself to studying and practicing medicine to counteract my challenges. In the mornings, I attended neurology outpatient consultations with Dr. Jaime Peres-Serra at the Hospital de la Santa Creu i Sant Pau. In the afternoons, I learned about neuropathology in the pathology department at Valle de Hebrón Hospital with Dr. Carmen Navarro Fernández-Balbuena.

These two years were highly productive, and my studies were extended when a tribunal declared me exempt from military service due to a diagnosed untreatable congenital disease that made me incompatible with military duty.

I continued learning from Carmen for some time until she had to relocate to Vigo to take a position as head of service, which allowed her to pursue a robust neuropathology program. At Valle de Hebrón Hospital, her path to practicing exclusively in neuropathology was blocked. At that time, neuropathology was still viewed with some skepticism by pathologists in our country, and neuropathologists often occupied a subordinate position within pathology departments.

During those years, at the neurological service at the Hospital de la Santa Creu i Sant Pau, visits with neurological patients could last for hours, as there was no time limit until a thorough neurological examination was completed. Later, reasoning was based on clinical symptoms and their possible anatomical correlates. A similar progression occurred in the field of neuropathology. In the early days, anatomical and histological studies emphasized understanding and identifying the brain's nuclei, regions, areas, and their interconnections.

Subsequently, neuropathological studies were carried out on hemispheric sections stained with hematoxylin and eosin, Mallory, and myelin stains. These basic techniques were followed by the use of specific silver staining methods, such as Bielschowsky's method. I was very pleased with the skills I learned during this university period.

The double rejection, in love and by the army, ultimately became a blessing for my future. My girlfriend was right to reject me; we wouldn't have understood each other. The military court's decision also spared me from having to confront the issue of declaring myself a conscientious objector to avoid mandatory military service at that time.

My ex-girlfriend and I became close friends, and two years later, I was thrilled to accept her invitation to become godfather of her daughter—a girl I adored. Now, my goddaughter is a divorced woman with three children, and I regard them all as part of my extended family.

## The beginnings (1975–1985): ages 24–34

I joined Hospital Príncipes de España in 1975. This hospital, established in 1972, was later renamed Ciudad Sanitaria de Bellvitge, and eventually became the Hospital Universitario de Bellvitge: Bellvitge University Hospital (HUB) in 2003. Hereafter, I will use HUB to refer to the hospital. It is located in Hospitalet de Llobregat, which is in the province of Barcelona and near the city of Barcelona. The Catalan Institute of Health (ICS), under the Health Department of the Generalitat de Catalunya (the autonomous government), manages the hospital.

The hospital was constructed on land that previously consisted of agricultural fields with isolated farmhouses, located just a short distance from the Bellvitge hermitage, a place of worship that one would visit after crossing the Llobregat River, with the city of Barcelona still a considerable distance away at that time (**Figure 2**). Additionally, a large social housing community was built on the same land, consisting of numerous high-rise buildings arranged in parallel blocks that accommodated individuals displaced from informal settlements near the coast and on hillsides surrounding Barcelona.

**Figure 2:**
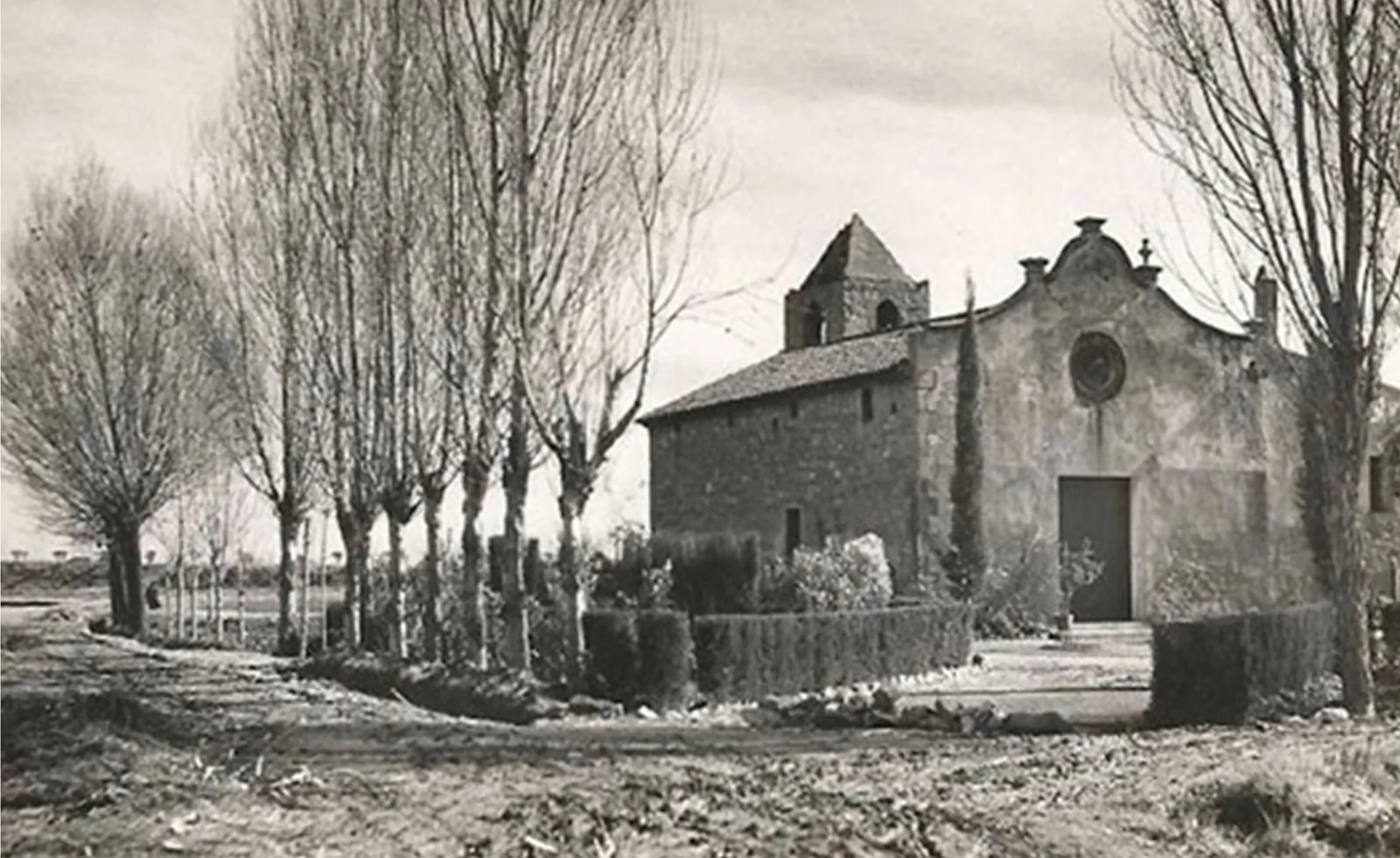
**Figure 2.** The Hermitage of Bellvitge surrounded by fields in the 1960s.

On rainy days, the village's streets and the hospital access routes would become muddy. Bellvitge was a typical new neighborhood designed for the working class in large cities, intended eventually to provide the same amenities as more central districts.

I was offered positions at other hospitals, where some personnel were hired after a successful interview without undergoing a specific examination. Still, I preferred HUB because starting nearly from scratch was an exciting adventure full of possibilities.

Today, the urbanized and tree-lined community has assimilated into the city of Hospitalet and connects southeastward toward the port of Barcelona, alongside its robust logistics and industrial zone. The Bellvitge campus comprises two hospitals that have undergone several phases of expansion, new surgical and outpatient facilities, a nursing school, a health sciences faculty, lecture halls, a library, and various laboratories and shared service research facilities (**Figure 3**).

**Figure 3:**
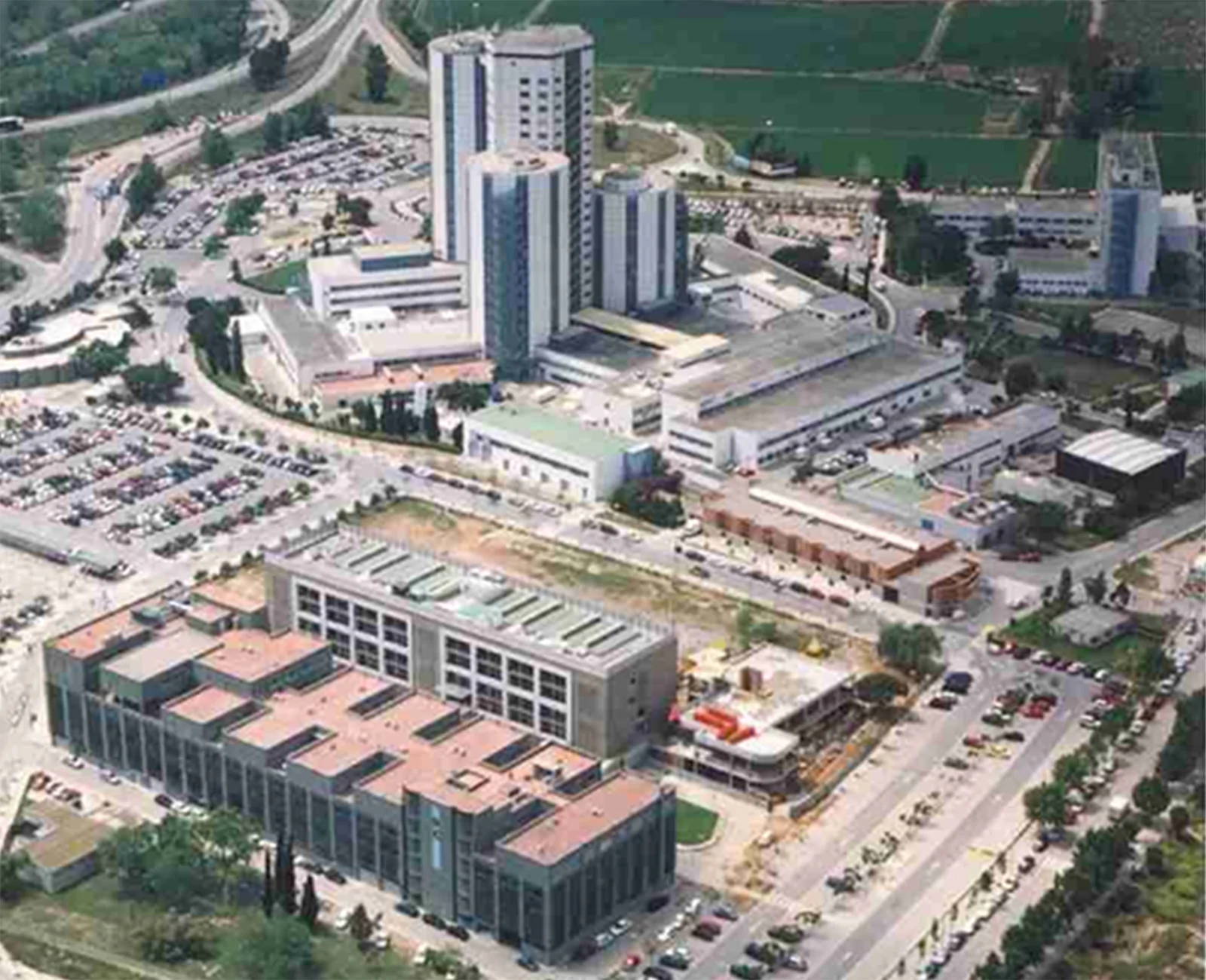
**Figure 3.** The Bellvitge campus, with the Faculty of Health Sciences at the University of Barcelona (UB) in the foreground and Bellvitge University Hospital (HUB) and its distinctive towers behind it.

In my first year as a resident physician, a strike was called to advocate for better working conditions for resident doctors and nursing staff alike. The authorities did not respond, leading to the dismissal of all residents, as well as certain nursing staff and attending physicians. Many individuals were reinstated in the months that followed.

A group of those laid off due to the strike, including me, travelled to Galicia. During the trip, a very dear friend and I decided that upon our return, we would live together. It was not a sudden infatuation but a decision made after several years of being together while living separately.

She was a dermatologist and, after completing her training, continued working in various hospitals. We married a few months after the trip, in January 1976. We have been together for fifty years; we have two children, and now we have five grandchildren. Our life together is the best thing that has ever happened to me.

After nearly a year and undergoing a trial, those of us who had still not been reinstated were allowed to return to the hospital. During that period, I studied neuropathology at San Juan de Dios Children's Hospital in Barcelona. I returned to my position at Bellvitge Hospital, but I continued my studies at San Juan de Dios Children's Hospital.

Two years after taking up a permanent position in the pathology department and after some training in general pathology and autopsy practice, I obtained the official qualification of specialist in pathological anatomy.

### Building neuropathology at Bellvitge

I never left HUB, although I have undertaken many short stays in different centers and institutions to attend courses or seminars or to learn new concepts and techniques. This form of learning, somewhat self-taught, showed me knowledge from different sources, as well as different ways of working.

The primary reason I remained in Barcelona was my determination to advance the development of neuropathology in Spain as a distinct field for understanding neurological diseases. This was my heartfelt ambition; my mind was running in parallel or somewhat behind, adapting to what seemed the best opportunities to achieve it.

I established myself as a neuropathologist, thanks to the head of the pathology department at the time, who recognized the value of this "specialty" and advocated for its establishment at HUB.

Once I had completed my daily clinical work in diagnostic neuropathology, I devoted my time to the laboratory to learn new techniques and methods, to further my training in electron microscopy, and to initiate research. I applied the rapid Golgi technique to investigate human brain pathologies, ranging from malformations and pediatric diseases, for which I had access to specimens at the San Juan de Dios Children's Hospital, to various types of dementia and other adult conditions studied using samples from the HUB and consultation cases.

The reagents were readily available, and after numerous trials with both successes and failures, the results were satisfactory. On some days, despite following the same protocol, the outcomes were disappointing for unknown reasons. At the same time, I became involved in the comparative histology of the mammalian brain and the structure and adaptation of neurons within the folds and grooves of the brain in gyrencephalic species. My admiration for the work of Santiago Ramón y Cajal inspired me to apply the Golgi method to human pathological samples.

I learned several silver techniques, particularly variants of Río Hortega's methods for studying oligodendroglia and microglia, Cajal techniques for astrocytes, and various silver techniques to distinguish intracellular elements and fibrils. That was a true work of craftsmanship (*artis-manus*), just as rewarding as building models of ships and planes during my adolescence.

I was fortunate to share my findings on silver techniques with Moisés Polak, a disciple of Don Pío del Río Hortega, whom I greatly admired both as a researcher and as a person. Additionally, I was able to discuss the results of the rapid Golgi method with remarkable individuals connected to Santiago Ramón y Cajal, including Miguel Marín-Padilla, Constantino Sotelo, Fernando de Castro, grandson of Cajal’s disciple Fernando de Castro Rodríguez, and Facundo Valverde, among others.

These techniques were challenging and required meticulous attention, but the results were spectacular. Many of these methods have not been used for years, as they have been replaced by more specific immunohistochemical techniques and *in situ* hybridization methods, as well as double- and triple-labeling staining. However, silver stains were remarkable and represented a major turning point in the history of neuroscience. I consider myself fortunate to have experienced the final chapter of this story in the company and under the guidance of irreplaceable individuals.

In its early years, the HUB's pathological anatomy department was equipped with competent staff and sufficient supplies for clinical pathology work, which expanded as new techniques and instruments were introduced. However, our department and the hospital as a whole had very limited facilities and resources for basic research. We had no facilities to maintain small animals for research. In the department of pathological anatomy, one room was adapted for experimentation, while animal housing was even more precarious.

We had animals (such as rats and mice in my case and torpedo fish for another group) housed in an ancillary room within the pathology department. Each research group took responsibility for caring for these animals. Additionally, I had hedgehogs, moles, and bats in a compartment in my in-laws' yard. Farmers routinely killed these animals to safeguard their crops and homes, so I obtained them alive for anatomical research directly from local farms.

Some farmers expressed curiosity about my experiments; there was no fixed price—just a gift: perhaps a bottle of wine, oil, liqueur, or chocolates for the family. The hedgehogs in their cages emitted a stench during the drive home that lingered for days in the newly acquired second-hand Renault 4L.

I also studied the brains of dolphins that washed ashore on the Costa Brava. After being notified by fishermen, I collected the heads of beached animals, which were transported in a refrigerated truck to the hospital's autopsy room. I caught some bats in caves where I had gone with some zoologists to mark the animals with metal rings and track the populations.

Choosing these animals was not a whim; dolphins and insectivorous bats have a neocortex with five layers, not six, as the specific and non-specific thalamic terminals end in the molecular layer, and there is no distinct fourth layer. The second layer of the neocortex is formed by extraverted neurons with large dendritic tufts. This type of cortex, characteristic of primitive insectivores (like hedgehogs and shrews), was retained in these new species when bats took to the air, and dolphins ventured into the sea about 70 million years ago.

From today's perspective, those practices seem barbaric, and they were indeed. Consequently, in 1985, the Experimental Research Unit was formed at HUB accompanied by a research committee responsible for regulating breeding and maintenance in the animal facility. Then in 1989, the Bellvitge Research Foundation was created, which transitioned to the August Pi i Sunyer Foundation in 1991 and eventually became the Bellvitge Biomedical Research Institute (IDIBELL) in 2004.

I was appointed deputy director of research at HUB until the late 1990s. This role involved organizing the physical and functional structures to enhance and optimize both clinical and basic research practices, as well as facilitating coordination between the two when feasible. All clinical trials, along with clinical and basic research activities, were documented, and each research group was required to present annual reports detailing resources, funding sources, and outcomes. These reports included information on conference presentations, publications in specialized journals, and patents. The annual reports were compiled and published to share the hospital's scientific achievements.

Another important issue during those years was the recognition of neuropathology as a specialty in Spain. This issue is crucial to understanding the poor development of neuropathology in the country. The regulation of medical specialties in Spain is the responsibility of the National Council of Specialties in Health Sciences (CNECS), which operates under the Ministry of Health. I participated in the CNECS meetings as an elected representative of specialist physicians. I proposed that neuropathology should be recognized as a separate specialty from pathological anatomy. Members of the Ministry supported the pathologists' argument that approving neuropathology could lead to the recognition of additional subspecialties, such as dermatopathology and gynecologic pathology. Consequently, the motion to establish neuropathology as an independent specialty was effectively blocked. This was a setback, and I thought the negative decision would remain unchanged for many years, perhaps indefinitely.

To overcome this stumbling block, and with the support of my department head, I began by establishing a neuropathology entity within HUB and giving it a name. Initially called the Neuropathology Unit, it was rebranded as the Institute of Neuropathology (INP) in 1985. The hospital's management and administration recognized this internal agreement. INP gained recognition as a leading center in neuropathology throughout the 1990s, expanding further in the subsequent years. The term "institute" was adopted to signify its roles in knowledge generation, scientific advancement, and innovation within the hospital environment. However, it was never officially accredited as an institute based on the standards set by the Ministry of Science, Technology, and Innovation in 2011.

This accomplishment cannot be attributed solely to my own efforts or those of my small team. These were years marked by significant growth and innovation at the hospital, spurred by robust healthcare initiatives, particularly in transplantation and renal pathology. A succession of managers and medical directors introduced novel concepts and fostered development and progress. The INP represented one of their achievements. I am grateful not only for the hospital's overall environment but also for the pathology department staff, who consistently assisted me with my neuropathology project. Without their ongoing support, it would have been impossible to advance this initiative.

Some departments raised objections to specific expansions of the INP. The establishment of a muscle pathology unit at INP caused disagreements with the neurology department. Dr. Montse Olivé Plana, an expert in electrophysiology with extensive knowledge of muscle diseases, led the unit. She was neither a pathologist nor a neurologist, although the neurology department directed neurophysiology. The creation of a hospital position assigned to the pathological anatomy department, supported by the direction of HUB, was interpreted by the neurology department as an intrusion on my part. However, her appointment and capabilities represented a breakthrough in the study of neuromuscular diseases that would never have occurred in the same way if she had continued within her former departmental hierarchy. The muscle pathology unit, which conducted clinical evaluations of patients along with complementary tests and muscle biopsy analyses, became a benchmark in the field. The later inclusion of genetic studies further improved the unit's effectiveness.

The establishment of a brain bank linked to the INP turned out to be a successful project. Initially, we preserved the brains from autopsies in separate plastic containers filled with buffered formalin, similar to other traditional brain banks. Later, we developed specific protocols to obtain fresh, small dissected areas from representative regions across different brain parts, rather than frozen hemispheric blocks (as these would shatter into multiple pieces when selecting regions for biochemical analysis). The small pieces were immediately frozen at -80 °C and stored in separately labeled plastic bags. This process was tedious, but the learning phase coincided with a period when I was on autopsy duty in the department. The technician and I spent much of our time dissecting the samples, and I instructed him in the procedure. Almost all brains with short post-mortem intervals were handled in this way. The samples were kept as remnants of the pathological analysis, in accordance with the existing regulations at that time.

This type of brain bank was distinctive, as most banks of that era predominantly housed pathological brains, rather than "normal" specimens, and had long post-mortem intervals until processing. Our particular procedure enabled us to investigate the alterations in the human brain associated with aging, employing not just histological but also biochemical and molecular approaches in tissue samples with optimal post-mortem conditions. The brain bank served as the basis of most of our research. Multiple laboratories have utilized these samples over time, either upon request or in collaborative studies.

During that period, my teaching position at the UB was based at the Hospital Clinic of Barcelona, where the Faculty of Medicine was located. I held the position of associate professor in the Department of Pathology. In 1986, I began teaching on the UB Bellvitge campus as a tenured professor after successfully completing the corresponding competitive examination. The pathological anatomy class was reduced to eighty students, allowing more individualized, focused theoretical instruction and better-prepared practical sessions for small groups.

During those years, one major accomplishment was establishing the Club Español de Neuropatología, which brought together neuropathologists in Spain. Initially, we were around ten members, but our numbers grew over time. The club operated through an annual general meeting and regular discussions and consultations among its members. These gatherings continue to occur and coincide with the Spanish Congress of Neurology and the Spanish Congress of Pathologic Anatomy each year. The club remains active today.

Alongside our work commitments, my wife and I had two children, and family life was very important to us. Although we aimed for an equitable division of responsibilities, my wife took on more childcare duties, while I handled the night-time feedings and diaper changes during the early years. To help manage our household, we soon hired a babysitter and later employed a part-time housekeeper to handle chores and meal preparation. We dedicated our weekends to our children and to spending time as a family. Family was a priority; over time, we went on outings with the children, often with the youngest carried on our shoulders, alongside other parents with children the same age. In the summer, we would go to the beach to swim or take an inflatable boat with an outboard motor around the Balearic Islands or along the Costa Brava.

I worked nights while everyone else slept, which meant I got very little rest. A few years later, I realized I could no longer maintain that demanding schedule. Things changed when the children reached adolescence. They had their own activities, and our involvement in their daily lives became more limited, except for the nights that felt endless until they got home.

## The middle years (1986–1999): ages 35–48

During this period, HUB was already a reference hospital thanks to the efforts of the majority of people involved at all levels, including managers and directors, administrative staff, doctors, nurses, technicians, and assistants. Anyone who had been there from the beginning could feel proud of what the hospital had become, despite the daily problems, just a few years after its inception.

### Research and clinical networks

The final decade of the 20th century was also crucial for clinical research in Spain. During this period, the first calls for research projects from the Health Research Fund (FIS) of the Carlos III Institute of Health (ISCIII), under the Ministry of Health, provided financial support for initiatives focused on applied clinical and experimental research related to human diseases. These FIS calls triggered a significant transformation in medical research within public hospitals. At that time, much of the research funding available to public hospitals came from industry-sponsored clinical trials which were primarily aimed at obtaining regulatory approval for pharmaceutical products rather than supporting investigator-initiated research.

In contrast, FIS calls proved advantageous for scientists in hospitals. We received several consecutive three-year grants focused on naturally occurring and pathological cell death in animal models and human diseases. Some students joined us because they secured their salaries from various institutions, while others participated as staff associated with well-funded collaborative European projects that financed their contracts. We also welcomed students who volunteered to learn specific skills; however, we preferred to avoid taking on individuals who would work unpaid for three or four years while completing their doctoral theses. Although this arrangement could be mutually beneficial, it also carried the risk of exploitation.

Additionally, we obtained a European grant to investigate the effects of ionizing radiation on brain development, a proposal prompted by the 1986 Chornobyl (or Chernobyl in the Russian spelling) nuclear incident in Ukraine. This funding enabled us to hire personnel for the European project. Funding from Spanish and European initiatives marked the beginning of a significant phase of research development.

We developed several animal models, primarily using rats, mice, and gerbils, to investigate natural phenomena in the developing brain, such as naturally occurring cell death, and to replicate human pathologies, including those induced by X-radiation. These studies modeled various types of brain malformations and microcephaly. Additionally, we examined the effects of chronic alcohol consumption on developing, adolescent, and adult brains. Our findings were compared with those associated with fetal alcohol syndrome in children and chronic ethanol intake in adults, using samples from our brain bank.

We also investigated cell death and regeneration in models of global cerebral ischemia in rodents and focal cerebral ischemia in rats. At that time, we focused on three key areas: the molecular mechanisms of cell death and survival in both the ischemic core and penumbra regions; the vulnerability of neurons in relation to calcium-binding proteins; and the remodeling of neurotrophic factors and their receptors in the penumbra and surrounding areas. The experimental studies on cerebral ischemia and hypoxia were especially stimulating thanks to the collaboration and friendship of Dr. Anna M. Planas Obrador of the Consejo Superior de Investigaciones Científicas (CSIC), one of the most creative and keen researchers I have ever known.

We also used rodents to conduct experimental trials testing specific molecules as potential treatments for neurological disorders. After several years of treatment studies in mice, we observed striking therapeutic responses. However, despite outstanding results in mice, subsequent clinical trials in humans did not yield the anticipated benefits. The challenges of translating pharmacological treatments from mice to humans have been widely discussed, highlighting the need for caution when extrapolating successful findings in rodents to humans.

At the end of this period, I had a clear idea of how I wanted to proceed. I wanted to continue my work in diagnostic clinical neuropathology and conduct clinical research using human brain bank samples. This would involve employing molecular biology techniques along with updated histological and immunohistochemical methods. Additionally, I aimed to conduct basic research using animal models to study human nervous system diseases. To achieve these goals, it was essential to secure additional funding to cover expenses for personnel, equipment, and laboratory consumables. Without a significant increase in funding, it would not be possible to undertake an independent research project.

In 1996, I was appointed to the position of Professor and Chair of Pathology at the Department of Pathology and Experimental Medicine associated with the UB campus in Bellvitge (**Figure 4**). This was accompanied by the addition of a new associate professor to our teaching staff. This role allowed us to establish a teaching program tailored to the new circumstances. The pathological anatomy course was split into two parts: general pathology in the third year and special pathology over the subsequent three years, in coordination with system-based clinical and surgical teaching.

**Figure 4:**
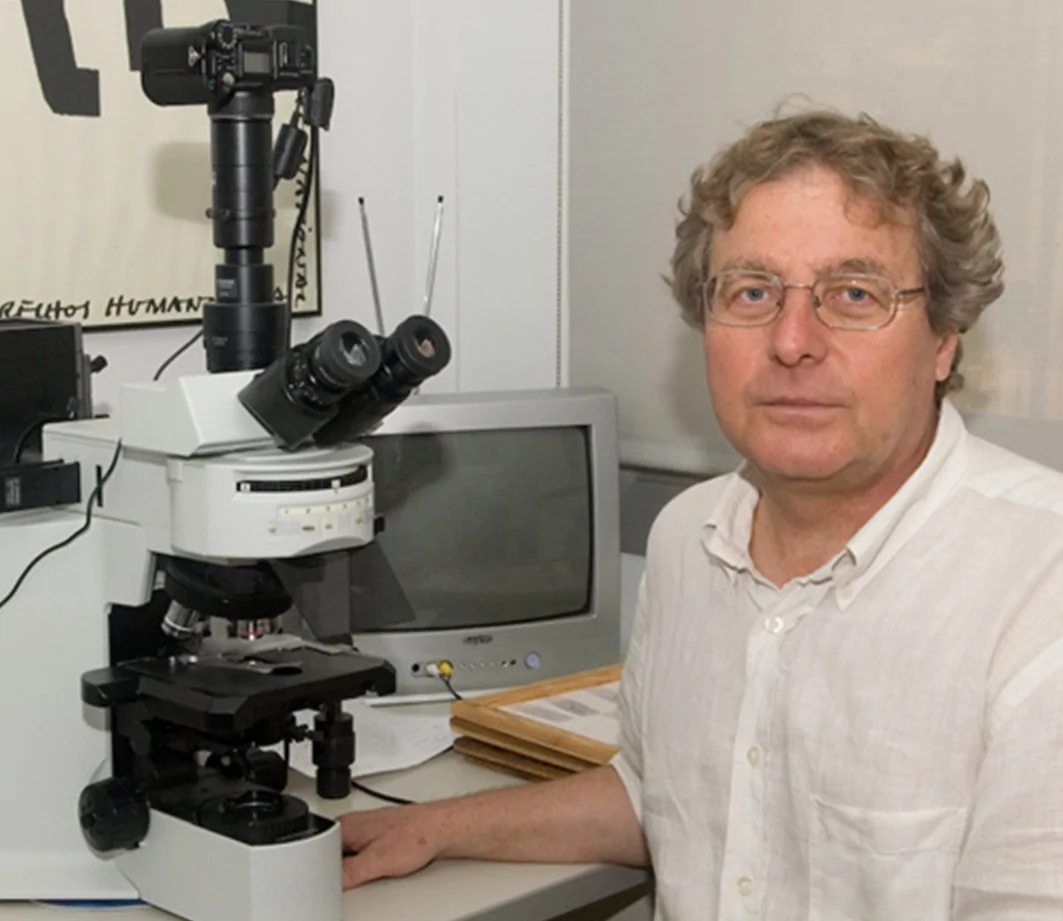
**Figure 4.** The author in 1996, when he was appointed Professor and Chair of Pathological Anatomy at the University of Barcelona.

Over the next two years, the INP expanded to include the laboratories at UB (INP-UB) and Hospital Duran i Reynals (INP-ICO). A biosafety laboratory was established at the INP by the HUB in 2004 to study the molecular aspects of prion diseases, in accordance with the directives of the Health Department of the Generalitat (autonomous government) and approved by IDIBELL.

A website was launched, managed by the audiovisual unit of the HUB, providing information on the organizational structure, resources, job opportunities, publications, and online courses. The website also offered free courses on various neuropathological topics.

An unrelated activity also deserves a short comment. Since my adolescence, I have practiced snorkeling. Eventually, I earned the Advanced Open Water Diver certification, which allowed me to dive up to 40 meters deep and gave me a better understanding of survival and underwater assistance techniques than the Open Water Diver certification. Between 15 and 25 meters was, for me, more than enough; I didn't need to go deeper: dive time shortens and decompression time lengthens as depth increases. I have gone diving on the Costa Brava and, occasionally, on tropical reefs. The practice, in silence, breathing slowly and acting as a spectator of the rocky walls, the seabed, and the animals and algae, was beautiful and soothing. Until a few years ago, diving was one of my main hobbies.

## The years of consolidation: biomedical institutions, facilities, and positions (2000–2016): ages 49–65

During this period, the autonomous government of Catalonia and the Spanish state government made several attempts to foster and organize research centers and networks. These initiatives had a significant impact on healthcare research and positively influenced the development of neuroscience, particularly in clinical and experimental neuropathology, after considerable trial and error.

In Catalonia, the Department of Health tried to establish the Catalan Institute of Neurosciences, which would consist of multiple nodes functioning as a network. This center would include a Catalan Network of Brain Banks across various hospitals in Barcelona and nearby areas, coordinated through a centralized registry at the INP.

The project was initiated, but it did not take shape for several reasons: **i.** The Department of Health's selection of clinical and basic groups, intended to cover the aspirations of the various hospitals and research centers involved, was indiscriminate and unrealistic. **ii.** Several universities each wanted to host the Catalan Institute of Neurosciences. **iii.** The budget was insufficient even for the initial centralization of administrative and management functions, and **iv.** The opposing views between the health department and the universities and research department of the Generalitat proved irreconcilable. The project was therefore cancelled.

Meanwhile, universities gradually developed their own neuroscience institutes: the Neuroscience Institute at UAB was founded in 2003, followed by the Neuroscience Institute at UB in 2015. INP, as the neuropathology group, was included as a member of the UB Institute of Neurosciences.

Regarding Catalan Brain Banks, the network made notable progress in unifying methodologies and sample processing, as well as in standardizing neuropathological diagnoses according to international nomenclature across existing and newly established brain banks. However, the network was disbanded once external funding for the collaborative project ended. From that point on, each brain bank became reliant on its respective hospital center.

In Spain, the Ministry of Health, through ISCIII, launched two initiatives: one focused on networked research centers and the other on biomedical research institutes.

The Research Center for Neurological Diseases (CIEN) comprised various Spanish clinical and basic research groups selected through a competitive process. CIEN encompassed neurological and psychiatric ailments, along with related neuroscience areas. I served as the interim coordinator of CIEN, aware that its format and makeup far exceeded the program's expectations (as with the Catalan Institute of Neurosciences). Nevertheless, moving forward with the initiative was a crucial first step toward creating a future research institution for neurological diseases in Spain, and we decided to move forward.

The failure of the unwieldy CIEN structure led the ISCIII to recognize that the structure needed to change in favor of a network of smaller, disease-focused centers. This collaboration between politicians and researchers was instrumental in the eventual functioning of the Network of Biomedical Research Centers (CIBERs). CIBERNED (Biomedical Research Network Center for Neurodegenerative Diseases) was among the earliest CIBERs.

The quote attributed to Winston Churchill, *"Success is the ability to go from one failure to another without losing enthusiasm,"* is appropriate and encouraging in this context.

Another ISCIII initiative was the establishment of Biomedical Research Institutes (BRIs), or Institutos de Investigación Biomédica (IIBs), affiliated with public hospitals within the Spanish healthcare system. These institutes aimed to foster and strengthen collaboration between clinical and basic researchers from hospitals and associated institutions, primarily universities and other research facilities. The BRI model generally proved effective, because all applicant institutions had to meet common organizational, management, and quality standards. Recognition as a BRI brought several benefits, particularly improved access to grants from the Ministry of Health of the Government of Spain. IDIBELL was inaugurated in 2004, becoming one of the first BRIs in Spain, largely due to the organizational framework the HUB had been developing in previous years.

The INP became part of CIBERNED and IDIBELL from their inception, representing one of the groups in the neuroscience sector.

At the same time as the establishment of CIBERs, ISCIII advocated the creation of a network of biobanks across Spain. An expert committee was convened to formulate specific protocols tailored to each biobank type. I participated in the meetings where the protocols already implemented by the Catalan brain banks were discussed and accepted.

**Figure 5:**
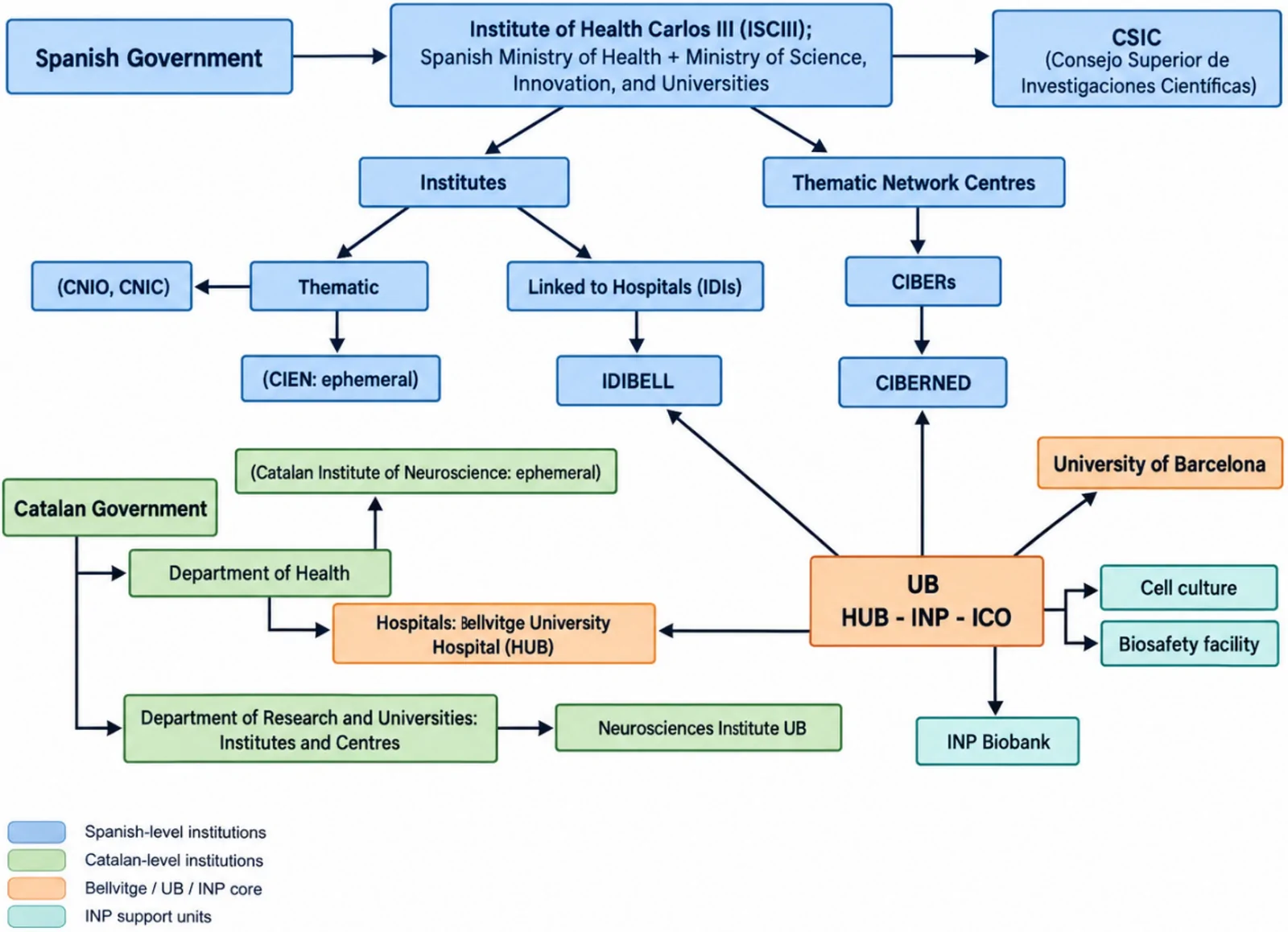
**Figure 5.** Institutional position of the Institute of Neuropathology (INP) and its relationships with other institutions. Abbreviations are defined in the list of abbreviations.

I was asked to serve as Vice-Rector for Scientific Policy in 2005 under the leadership of Rector Marius Rubiralta Alcañiz, who had previously initiated the Barcelona Science Park and advanced the modernization of applied sciences as well as the adoption of the R&D framework at the UB. The vice-rector's role was to promote and coordinate the various research institutes and science parks, as well as both established and emerging groups within the UB's scientific and technological development. The responsibilities also included coordinating the university with the various consortia to which they belonged, as well as overseeing foundation reports, primarily those connected to health sciences.

I remained in this role until 2008, when the rector assumed responsibility for the State Secretariat for Universities at the Ministry of Science and Innovation, serving in that capacity until 2012. Although the vice-rector position for scientific policy included intriguing theoretical aspects, I consistently found the bureaucratic and administrative tasks overwhelming. I am grateful to the administrative staff whose experience helped me fulfill my responsibilities successfully.

At this time, the establishment of INP as a reference center for clinical neuropathology, brain banking, experimental neuropathology, molecular pathology of the nervous system, and education would not have been possible without the financial support obtained through grants and research projects from various entities.

In addition to individual FIS projects, ISCIII initiated a call to promote collaboration among research groups within the BRIs on specific topics. We received a collaborative research grant linked to IDIBELL to investigate the role of the locus coeruleus and its connections in the context of aging, cognitive decline, and age-related depression. Another significant contribution stemmed from the neuropathology group's involvement in CIBERNED. This affiliation with the network, obtained through a competitive selection process, provided core funding as well as additional funding based on the group's performance and excellence, evaluated through annual reviews. The intra-CIBERNED projects also provided additional funding for specific and collaborative work among CIBERNED groups. Similar opportunities arose through cross-border European projects devoted to the study of prions and molecules with prion-like functional mechanisms. These projects brought together groups on both sides of the Pyrenees and included clinical and basic researchers working on human and veterinary diseases.

Lastly, our participation in various European research programs and consortia, involving groups from several countries, was paramount. BrainNet Europe was a "Network of Excellence" funded by the European Commission under the 6th Framework Program "Life Science" and supported through two consecutive calls over six years. It comprised 19 established brain bank partners across Europe. It was coordinated by Professor Hans Kretzschmar. The collaborative consortium encompassed thorough examinations and procedures in the design and operation of brain banks. The topics covered ethical and organizational aspects, clinical data management, sample collection and processing optimization, analytical techniques, and harmonization of neuropathological diagnoses. Additionally, protocols were established to optimize large-scale molecular analyses of proteins, lipids, DNA, and RNA in frozen samples. The project exceeded expectations and laid the groundwork for the standardization of molecular neuropathology in post-mortem human brains affected by neurodegenerative diseases and mental illnesses, beyond the analysis of individual molecules.

The European project Develage, led by Professor Gabor G. Kovacs, lasted from 2012 to 2014 and aimed to analyze shared molecular pathways between early developmental processes, aging, and neurodegenerative brain diseases.

Beyond the many relevant observations generated by both projects, and reflected in multiple joint publications, what I valued most was the willingness and enthusiasm of all participants, the working environment and the collaboration between the different scientific and management teams. Both European projects were highly successful and personally very rewarding.

The European Consortium Neuroprion encompassed more than 90% of the European teams working on prions (over 50% worldwide) and operated from 2003 to 2009. In addition to the multidisciplinary approach to prion diseases, Neuroprion contributed to related areas of animal and human health and food safety.

Indabip was a European collaborative project focused on the study of Parkinson's disease (PD), involving three scientific teams and two companies. Indabip started in 2006 and lasted for three years. The objective of this project was to identify specific therapeutic targets for PD. Two potential targets were registered for development by the companies. Many results were subject to a confidentiality clause; they were never published or used in subsequent studies because they did not appear to have any therapeutic relevance. I was frustrated by the amount of data generated that remained unexploited.

This overall scenario gave the INP considerable financial independence. It allowed us to offer contracts that attracted personnel well-suited to the requirements of the specific projects and to organize work around the individual responsibility of researchers and technicians. Greater emphasis was placed on the innovative ideas that could stem from creative freedom rather than on the number of hours spent in the laboratory. While this approach carried certain risks, I was pleased with the outcomes. The INP's success largely stemmed from its financial independence, with abundant, diverse funding sources that allowed it to hire personnel and acquire equipment and materials for laboratory operations. This generated high overhead revenues for IDIBELL. Without financial independence, the laboratories could not have operated.

During the years of consolidation, we continued studying, as in previous years, biology and molecular biology and incorporating methods for the study of lipids, proteins, nucleic acids, and other molecules in brain homogenates and in cellular fractions of nuclei, membranes, mitochondria, lipid rafts, and other components. Applying these approaches to the aging human brain and neurodegenerative diseases was central to the development of molecular neuropathology based on the acquisition and integration of large datasets. Working with experts in each of these fields was a unique and mutually beneficial experience. Taken together, these studies represented the most creative part of my work during these years. It was a happy period (**Figure 6**).

**Figure 6:**
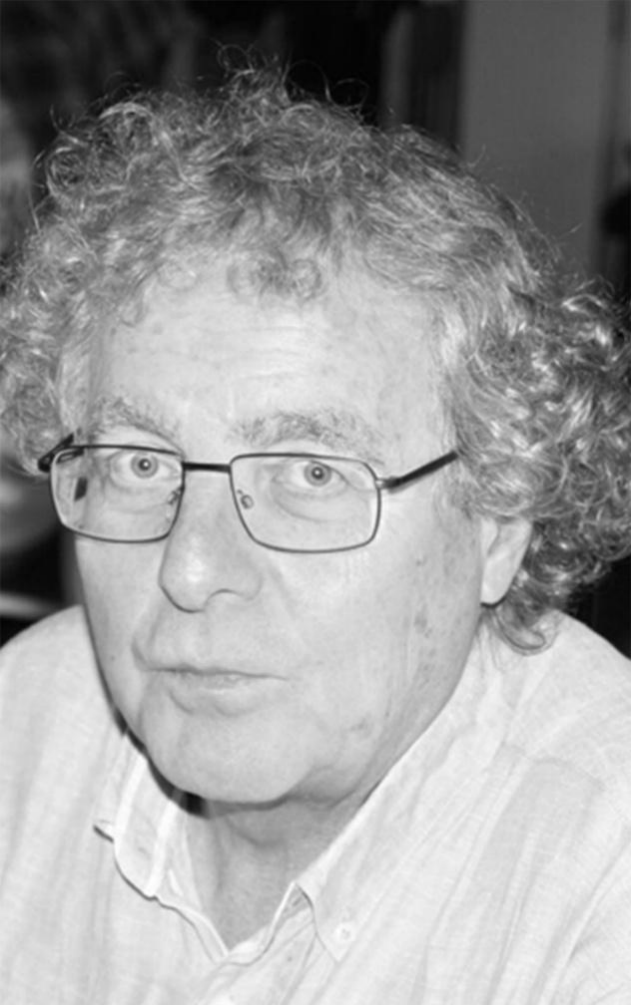
**Figure 6.** The author around 2010.

Another important relationship was with Professor Martí Pumarola, whom I mention here not out of courtesy but because of his major contribution to veterinary neuropathology in our country. Now retired, he had worked as a professor at the Veterinary Faculty of the UAB, and is widely recognized internationally as a veterinary neuropathologist and teacher.

We have spent many hours peering through the microscope, either in the faculty's laboratories or at my home, and discussing hundreds of cases over the years. The cases were seen at the veterinary hospital adjacent to his faculty or were referred to him for consultation from other centers, including Barcelona Zoo. We planned to create an animal brain bank linked to the Barcelona Zoo. However, the project generated little interest among the zoo's managers, who argued that the limited budget and personnel did not permit further commitments.

### Newton’s cradle: a different model

Some years ago, I imagined myself as a kind of Sisyphus, struggling to push a massive stone up a slope, only to watch it roll back down and start the process all over again. In the original myth, Sisyphus's toil is a divine punishment. While the work at INP was certainly challenging with its frequent ups and downs, I now think that this image, drawn from my earlier reflections, may lean too much toward a narcissistic sense of victimhood. I prefer to embrace a different model.

Newton's cradle consists of a row of identical rigid balls, suspended by parallel threads and arranged so that they barely touch one another. When one ball is displaced and released, it strikes the other ones, causing the ball at the opposite end to swing away. The same effect occurs whatever the number of balls displaced. The movement illustrates the transfer of momentum and energy through the row of balls. The balls in the center do not move, yet they are indispensable for transmitting the impulse. In much the same way, a successful project or an exciting result, could set an apparently unrelated project or experiment in motion elsewhere.

Newton's cradle is also one of the most common desk toys used to reduce stress. The continuous and perfectly symmetrical movement captivates the eye, while the steady metallic clicking of the spheres produces a rhythmic sound. Becoming absorbed in these visual and auditory rhythms can provide a momentary sense of relaxation, thereby reducing mental fatigue and anxiety.

Other ways to solve a problem are more rude and expeditious: How do you make a hole? By trying and doing it. This was a favorite saying of my friend Jorge Caralt, who has already passed away. It works well without much philosophy.

### Challenges at the INP

Looking ahead, my search for a replacement at the INP faced numerous challenges. The individuals appointed as associate professors at the UB were general pathologists in the department of pathology at the HUB, tasked with teaching general and specialized organ and system pathologies, and they showed little interest in neuropathology. Moreover, Spain has no formally recognized training pathway in neuropathology. The non-pathologists employed at the INP laboratories were biologists with limited expertise in pathology and neuropathology. In Spain, the number of general pathologists with training in nervous system pathology who work in hospital pathological anatomy departments has increased, with their neuropathological practice focused primarily on tumor diagnosis and autopsy pathology. These professionals combine neuropathology with the diagnosis of other more prevalent pathologies. Only a small number of neuropathologists in Spain work exclusively in nervous system pathology, mainly in a few brain banks.

The prerequisite for obtaining a position assigned to neuropathology in departments of pathological anatomy in Spain was qualification as a specialist in pathological anatomy. This requirement was the first bottleneck. I had two serious setbacks in this process.

In the first case, the candidate lacked the Spanish pathological anatomy specialist title. Moreover, her European Fellowship in Neuropathology (EFN) certificate from EURO-CNS (European Confederation of Neuropathological Societies) was considered, for the present purpose, of no value.

Try explaining to an administration that value sometimes lies outside the norm, and that such opportunities are worth recognizing. In trying to make this point, however, you may inadvertently offend those whose priority is maintaining order. This is where a turning point can occur. You risk acquiring the label of “outsider”. Over time, evidence accumulates, reinforcing their perception that you are different, and justifying, in their eyes, your marginalization.

My heated discussions with the hospital authorities over their refusal to recognize European qualifications, also affected my interaction with the candidate, who, through no fault of her own, was unfairly burdened by my frustration. This is the point that I regret most.

In the second instance, a few months later, after several discussions with the leadership of HUB and IDIBELL, I was informed that the INP would no longer be able to operate independently, as it had from the beginning. As a result, I could not guarantee the candidate's continued position at the INP.

I realized, for the first time, that INP's continued existence was in jeopardy. These succession problems reflected a darker and more hopeless scenario. Despite my attempts, the INP became less of a priority for IDIBELL and HUB after 2012. A few months later, HUB ordered me to dismantle urgently the molecular pathology, muscle pathology, and biosafety laboratories. Only the muscular pathology laboratory was allocated space within the Department of Pathological Anatomy. Although the dismantling of INP-HUB had been ordered as a matter of utmost urgency, it dragged on for several months, leaving people bewildered and uneasy. This situation culminated in the appointment of the new head of the Department of Pathological Anatomy, who ultimately disbanded INP-HUB. However, neuropathology at the Bellvitge campus did not disappear abruptly; the decline continued over several years.

At times of intense pressure and helplessness, I have experienced bouts of aggressive anger, which I consider my greatest flaw. Although these episodes were short-lived and did not lead to lasting resentment, they nevertheless had negative consequences. In those moments, the advice of classical Stoics, Epicureans, and later pacifists—some of the most insightful thinkers on achieving mental balance—was of no help to me.

My only solace, with time, has been to see that both candidates are now practicing as neuropathologists at institutions abroad, where their outstanding skills and expertise are acknowledged. Looking back, both have prospered in their careers and have achieved greater success and personal wellness than if they had remained here.

## The last years (2016–2024): ages 65–73

After I retired from the HUB in 2016, the new part-time head of the Pathology Department led the dissolution of the clinical and molecular neuropathology unit and the brain bank. He made immediate, drastic decisions: my office and my secretary's office were eliminated; I was left with only a corner workspace in a corridor; my secretary, who excelled at administrative management and had extensive experience, was reassigned to routine tasks. The molecular neuropathology lab and the biosafety lab dedicated to researching prion diseases were shut down, and the Biobank associated with the Neuropathology Institute (Biobank-INP) was transferred to the HUB-ICO-IDIBELL Biobank, now known as Neurobell/IDIBELL. Within weeks, the INP's clinical branch (INP-HUB) was closed. It was a death foretold.

Personnel employed by the hospital solely for neuropathology saw their workloads diminish and took on responsibilities in other areas. More specifically, the pathologist in charge of neuropathology took on new responsibilities in general pathological anatomy, reducing neuropathology to a minimum, as had been foreshadowed a few years earlier. Brain bank activity was close to zero. In addition, the contract of a biologist in charge of molecular neuropathology was not renewed on expiry.

The Muscle Pathology Unit struggled under the hospital's new dual management by the pathology and neurology departments, since the latter wanted to regain full control of muscle pathology. As a result, some time later, the unit coordinator left for Hospital de la Santa Creu i Sant Pau, which offered her better professional conditions and greater professional recognition. After her departure, the muscle pathology unit at the HUB lost its status as an internationally recognized reference unit. Personnel brought on board through external research project funding were reassigned to the INP-UB and INP-ICO laboratories. Despite these actions, external consultations for central nervous system pathology continued to be directed to the INP-UB laboratories. My concerns from previous years about the failure of neuropathology at IDIBELL were gradually and relentlessly borne out.

What happened to the INP-ICO laboratory two years later was unexpected and ruined my expectations. I offered a woman researcher a UB position in the cell culture area of INP-ICO. The general director of IDIBELL approved this plan. Once her contract was signed, IDIBELL allocated a significant portion of the INP-ICO laboratory to the new researcher a month later. Moreover, she was granted independence from the INP, allowed to establish her own research group, and given the freedom to redesign the laboratory as she wished without any further negotiation with me.

The result was a substantial reduction in the space available to INP researchers. It was especially disruptive for Dr. Marta Barrachina Castillo, a researcher who had founded a rapidly growing spin-off company. Fortunately, the company eventually became a leading independent entity outside the Bellvitge campus, focusing on the development of innovative blood biomarkers for the diagnosis and prognosis of Alzheimer's disease. Other INP-ICO members also faced space shortages during the laboratory renovations and were transferred to the UB laboratory.

### Brain aging, disease, and public health

Despite the discouraging circumstances at that time, I remained particularly dedicated to studying brain aging in animals and humans and the natural history of the spectrum of Alzheimer's disease (AD).

As in previous years, project funding was essential to continue the research. The FIS projects, the annual grants from CIBERNED, and a collaborative project funded by La Caixa made it possible to study the seeding and spreading capacity of tau obtained from different human tauopathies and inoculated intracerebrally into various transgenic mouse models. The results showed inter-neuronal transmission, axonal transport of abnormal tau along long connections and involvement of oligodendroglia. The studies also demonstrated the relative resistance of young animals compared with the greater vulnerability of adults, as well as similarities and differences in the types of tau deposits across tauopathies, and the transmission of phosphorylated tau as a phenomenon involving the activation of tau-phosphorylating kinases in recipient cells.

The continuation of my La Caixa-funded project also encountered problems. The general director of IDIBELL wanted to replace me as the principal investigator, disregarding the fact that I was emeritus professor at the UB. La Caixa categorically refused, threatening to terminate the project. IDIBELL therefore had to maintain my position, but issued me a salaried contract that I considered unnecessary and charged it to the overall budget, although it had not been included in the original project. I consequently had to pay tax on this additional income.

The pace of research was intense, and I became particularly interested in human brain aging and AD as a manifestation of it. By age 65, half the population has β-amyloid plaques; more than 80 % have neurofibrillary tangles; and more than 90 % have them by age 85 (Arnsten et al., 2021; Ferrer, 2023b). However, only one in four individuals presents with moderate or severe cognitive impairment at this age. These data suggest a continuum of neuropathological changes linked to brain aging and AD. These observations led us to propose that AD begins in young adulthood, although it only becomes clinically apparent when there is a significant burden of lesions that result in cognitive impairment and dementia. This pattern of tau pathology does not occur in other mammals. Thus, we suggested that AD is part of human brain aging (Ferrer, 2022; Ferrer, 2023a).

Moreover, aging in the human brain involves multiple molecular changes that become increasingly complex and adapt with age. These changes affect mitochondria and energy metabolism, DNA, RNA, protein synthesis, phosphorylation and other post-translational changes in proteins, lipid composition (including lipid rafts); the composition and molecular interactions of cytoplasmic and intracellular membranes, and alterations in oxidative metabolism, as well as specific alterations in astrocytes, oligodendrocytes, microglia, immunity, and blood vessels (Ferrer, 2022; Ferrer, 2023a).

These observations on the aging of the human brain and its relationship with AD, so different from those of other mammals, may help elucidate the complex molecular changes that occur in the brain with age, improve our understanding of the substrate of cognitive decline and dementia in the elderly, and help redirect therapeutic strategies beyond tau and β-amyloid in brain aging and dementia.

### Ebola

The Ebola epidemic in Sierra Leone between 2014 and 2016 had a profound personal impact on my wife and me. We worked on a project at the St. John of God Catholic Hospital, also known as Mabesseneh Hospital in Lunsar, where the Spanish missionary Manuel Garcia Viejo, a friend of my wife, played a crucial role. He served as a full-time doctor, surgeon, pediatrician, gynecologist, and essentially filled every medical specialty in the hospital. He was a generous and good man. Tragically, Manuel Garcia Viejo was the only Spanish doctor fatally infected with Ebola after coming into contact with one of his patients. He was transferred to Spain for treatment but ultimately died in Madrid. After the epidemic ended, we could neither trace the supplies we had sent nor continue our project. As has often happened during conflicts in Africa, the aid material had been most likely seized by someone along the way.

### COVID-19

The COVID-19 pandemic in 2020 also had detrimental effects on our laboratory, as it did for everyone, although fortunately they were less severe than elsewhere. During the first lockdown in Spain, from March to June 2020, the only activity was maintaining the mouse colonies in the animal facility, continuing already initiated animal treatments, and performing basic cell culture maintenance. During the second state of alert from October of the same year until May 2021, the same activity continued until "normal" activity could be resumed in shifts with a couple of people at a time in the laboratory. As a result, all experiments slowed down. The number of victims and deaths outside the laboratory put our relatively minor restrictions into perspective. Personally, I mourned the loss of friends, as many families did. However, life at home adapted well to the circumstances. My eldest son lived in Cameroon, and the youngest, a PhD in physics and later a professor, was working in Paris. Our home is a three-story family house. We could exercise every day by repeatedly climbing the stairs to the rooftop. We could also go out to a courtyard with a few trees, where we could see the sky and the sun every day.

### Prions

The situation in the UB laboratory was made worse by a tragic incident. A researcher who had been recruited two years earlier as an independent investigator to study biomarkers in human samples from patients with dementia or other neurodegenerative conditions developed a rapidly progressive neurodegenerative illness. Unexpectedly, the family formally requested that his illness should not be discussed or investigated and warned that they would take legal action for defamation. This unusual request prompted me to review the work carried out by his research group in our laboratory.

A subsequent thorough investigation found that biological samples suspected of containing prions were stored in a freezer designated exclusively for this research group. I also discovered that these samples had been handled at the INP-UB facilities without my knowledge. No authorization had been obtained for this work, and the handling of potentially infectious prion samples was not permitted at either the UB or IDIBELL. I reported these findings only to the relevant authorities at IDIBELL, UB, and CIBERNED. The laboratory personnel who had potentially been exposed helped to uncover what had occurred.

The institutions involved responded by establishing a commission to investigate the incident. Real-time quaking-induced conversion (RT-QuIC) testing confirmed the presence of prions in the suspected samples. After four years of investigation, I was not informed of the commission's conclusions. In my view, the incident raised a serious laboratory-safety and public health problem. Nevertheless, no information concerning the deceased researcher's illness or related circumstances was disclosed, in keeping with the family's request. The incident received extensive coverage in newspapers and other media, including independent journalistic investigations, some of which showed remarkable rigor. Eventually, the media coverage faded, and the incident ceased to attract public attention.

I cannot provide further detail about this public-health incident because legal proceedings are still ongoing. I am the only person against whom the family has taken legal action. I have received no support or assistance from IDIBELL in this matter.

This narrative sharply contrasts with the scenario encountered during the bovine spongiform encephalopathy (BSE) or ''mad cow disease'' epidemic, which posed a global health crisis at the beginning of the 21st century. In affected nations, most governments developed strategies to manage and prevent further cases of prion disease in both animals and humans. In Spain, an interministerial commission was formed, consisting of representatives from three ministries and supported by a scientific committee that offered current scientific insights on these diseases to guide appropriate responses.

For two years, I served on the scientific committee alongside two other specialists: a veterinarian and a physician. We participated in face-to-face meetings with all members of the interministerial commission. This effort was well-coordinated, rigorous, and effective, despite the challenges posed by the social and economic repercussions of the proposed measures. It resulted in significant improvements in traceability, transparency, and safeguarding against potential future risks.

The Government of Catalonia, for which I also served as a scientific advisor, showed similar dedication in crafting region-specific strategies. This initiative, aimed at grounding policy decisions in scientific insights, led to a fruitful collaborative effort to address a complex issue.

In the aftermath of that crisis, examining human prion diseases acquired from contaminated food, organ transplantation, contaminated medical instruments, or improper handling of prion-containing samples became increasingly important.

## Retirement and perspective

Right before I retired from the UB, I underwent quadruple coronary bypass surgery due to ischemic heart disease. Four months later, the INP-UB laboratory was relocated to another building and reassigned to a different research group, specifically the hospital's neurology department. It was disheartening to witness conflicts among other UB groups striving to claim the vacant spaces at the university, with little apparent benefit beyond the redistribution of territory.

Now that I am retired, I feel relieved of all the administrative tasks, meetings, and unnecessary daily challenges that used to consume much of my time. I was definitely tired of people who were initially nice but later became cold, calculating, egocentric, deceitful, and unscrupulous. I grew tired of dealing with inflated egos, liars, and envious individuals—people who seem to exist only to find ways to hurt others or to sit back and enjoy the damage caused by someone else. They devoted time and energy to negative actions that I neither had nor wished to waste on them. I do not understand the pleasure some people take in causing harm, nor the *Schadenfreude* of the silent witnesses.

Disillusionment has made me feel disconnected from them. It's not about forgiveness; it's about the indifference needed to survive. It's interesting how, suddenly and without warning, you can reach a limit. A firm "no" emerges, is felt, and accepted — and there is no discussion or turning back.

I first heard the proverb in Tokyo: *"The nail that sticks out gets hammered down"* (*Deru kui wa utareru*). At first glance, it suggests that conforming to expected behavior related to activities, status, seniority, and language is crucial for maintaining respect, social harmony, and hierarchical order. If someone deviates from the norm, action is often taken to restore conformity. The guide who accompanied me for a few days in Tokyo after the congress on prions in 2016 was a young woman whom I had met in Barcelona three years earlier and who had since become a friend. She worked part-time as a nurse in a hospital while supplementing her income by sharing her knowledge of history, sociology, and art as a private tour guide in Tokyo. We commented on various customs and habits in Japan that pertain to social hierarchy, age, and the influence of ancestral traditions in workplaces, such as hospitals and collaborative groups. She used the proverb to summarize these expectations. Our conversation was lively, critical of stereotypes and preconceived ideas, while also celebrating the unique and rich cultural heritage of her country.

I shared my thoughts with her, telling her that our societies had something in common. In our society, thinking, living, and working independently—merely acting outside of established or accepted norms—is not well tolerated either. Individuality is often discouraged in favor of uniformity, resulting in a homogeneous society that poses no challenges to authority, even when its laws may be mediocre or misguided. Those who stand out may find themselves excluded or rejected.

Playing with words with my guide, I replied with another quotation—this one attributed again to Oscar Wilde: *"When people agree with me, I think I am wrong"*. She said, "Look how he ended!"

In my life, I've felt an instinctive, voluntary pleasure in learning. The nervous system is a marvel when we perceive and are aware of our environment. A closer look at the mechanisms that make it work only increases our admiration. Understanding how molecules interact to produce specific functions is profoundly impressive. Exploring these processes highlights their importance in understanding health and disease and inspires a deeper appreciation for biological resilience and vulnerability. In any case, I am lucid about my limitations, and I accept I cannot go further.

Yet I am excited about the future of science and research. Advances in AI, when applied to knowledge and combined with large and complex datasets, may significantly enhance our understanding of how the nervous system functions as we age and in the context of neurodegenerative diseases.

Brain stimulation techniques and the intracerebral implants of microchips, whether linked to external devices or not, may significantly transform certain capabilities of the human nervous system. The issue is not just the individual effects of these devices, but also the disparities they will create between those who have access to these advancements and those who do not.

## Concluding comments

I remember the first time I gave an oral presentation at a conference. It was in a palace or something like it: a large, majestic hall with tapestries on the walls and portraits of serious figures everywhere. It was full of people. I was scared, but shortly after starting, everything became easier, and the images from Golgi stains in lissencephaly brains clearly showed the nature of the cortical migration disorder (**Figure 7**). I imagine that most young researchers experience something similar at some point.

**Figure 7:**
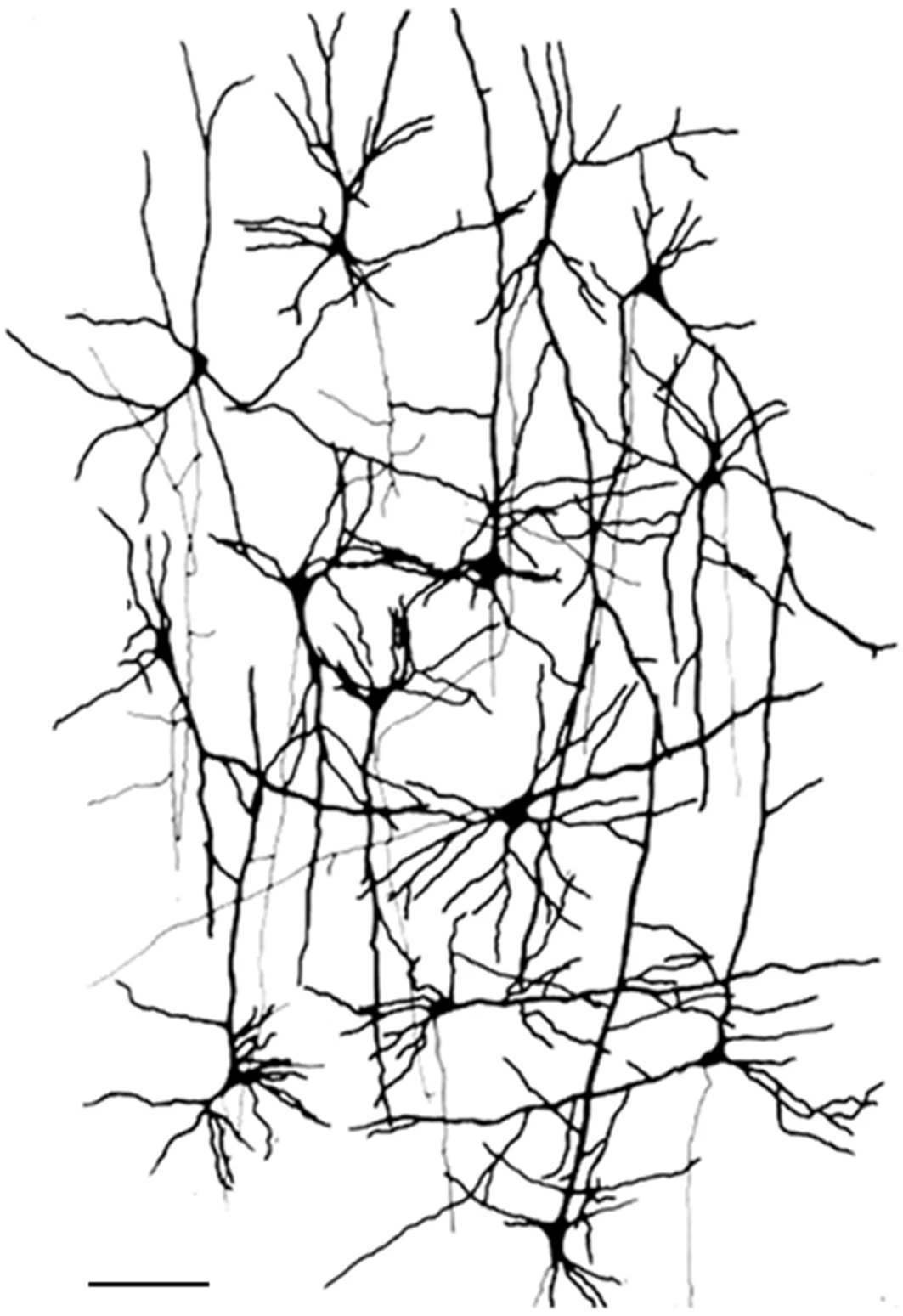
**Figure 7.** Camera-lucida drawing of Golgi-impregnated neurons in layer II of the cerebral cortex in type I lissencephaly (1976). Scale bar: 100 micrometers.

Throughout my professional life, I have given several hundred, perhaps thousands, of classes, seminars, conference presentations, and outreach lectures. I have directed about forty doctoral theses and tutored hundreds of students. I respect the audience and believe that receiving a message should also be an enjoyable experience. Every occasion was a new experience that filled me with respect, mixed satisfaction, and a slight unease. Each tutorial was singular, even in exceptional interactions where both teacher and student, or colleagues, which had incompatible personalities.

I recall many people I have met. I have learned from those who know and those who do not. I have received help from many people connected to the profession: students, doctoral students, doctors, specialists in various disciplines and fields of knowledge, technicians, administrative staff, and men and women from different countries. I am grateful for all of it and for all of them. Not mentioning their names is not a sign of laziness. On the contrary, there are too many individuals to list here, and I would not want to overlook anyone because of the limitations of my memory. I hope they understand my limitations.

I want to thank the editors of the scientific journals on whose editorial boards I have served for their trust in me and—especially in many cases—for the friendship and affection we came to share.

When I was young, I traveled to various places in Spain and other European countries. Later, conferences and seminars related to my professional work allowed me to continue traveling. I always took advantage of the trip to extend it and have a few free days to explore the place on my own. In the case of seminars and courses, I was surprised by the warm welcome and hospitality of the hosts from all the Ibero-American countries that invited me once or several times: Mexico, Cuba, Colombia, Chile, Argentina, and Brazil. Visits to China, India, Ethiopia, Georgia, Egypt, and Morocco were generally more formal, as were many of my professional visits within Spain for courses, seminars, and doctoral theses or faculty appointments.

Two or three times a year, there was a congress on neuropathology, neuroscience, or neurology somewhere in Europe: I have visited almost every European country more than once. I have also traveled to the United States, Canada, and Japan.

The congresses had a more formal atmosphere than seminars and courses; all the participants behaved in the same way, polite and smiling. The presentations were sometimes brilliant, other times simpler. Some speakers seemed to dictate the absolute truth on a subject; others were more restrained and expressed their opinions and their doubts. Many presentations were enlightening. One part of the conferences that I found interesting was the poster exhibitions with a multitude of new ideas. I liked walking among the posters and studying the details when there were no people around, during times when other activities were happening. Almost every poster reflected enormous effort, and many new and attractive ideas had clearly been developed with enthusiasm, often by young researchers. Afterward, I enjoyed speaking directly with some of the authors during the designated discussion periods. The visits to some areas of the country after the congress were as rewarding and exciting, in most instances, as the scientific event itself.

Africa has been a unique experience. I'm particularly familiar with countries in Atlantic sub-Saharan Africa, including Sierra Leone, Ghana, Senegal, Gambia, Benin, Nigeria, and Cameroon. Recently, I have started to visit Madagascar on the opposite side of the continent. My trips to these countries have had different but interconnected motivations. The first motive was to visit the countries where my eldest son has been working on European Union development programs for decades. The second incentive was volunteering with organizations and foundations that focus on protecting and educating children, assisting disabled children, and promoting women's development and rights. Some of the latter, such as those in Sierra Leone and Gambia, were closely linked to my wife's initiatives (**Figure 8**).

**Figure 8:**
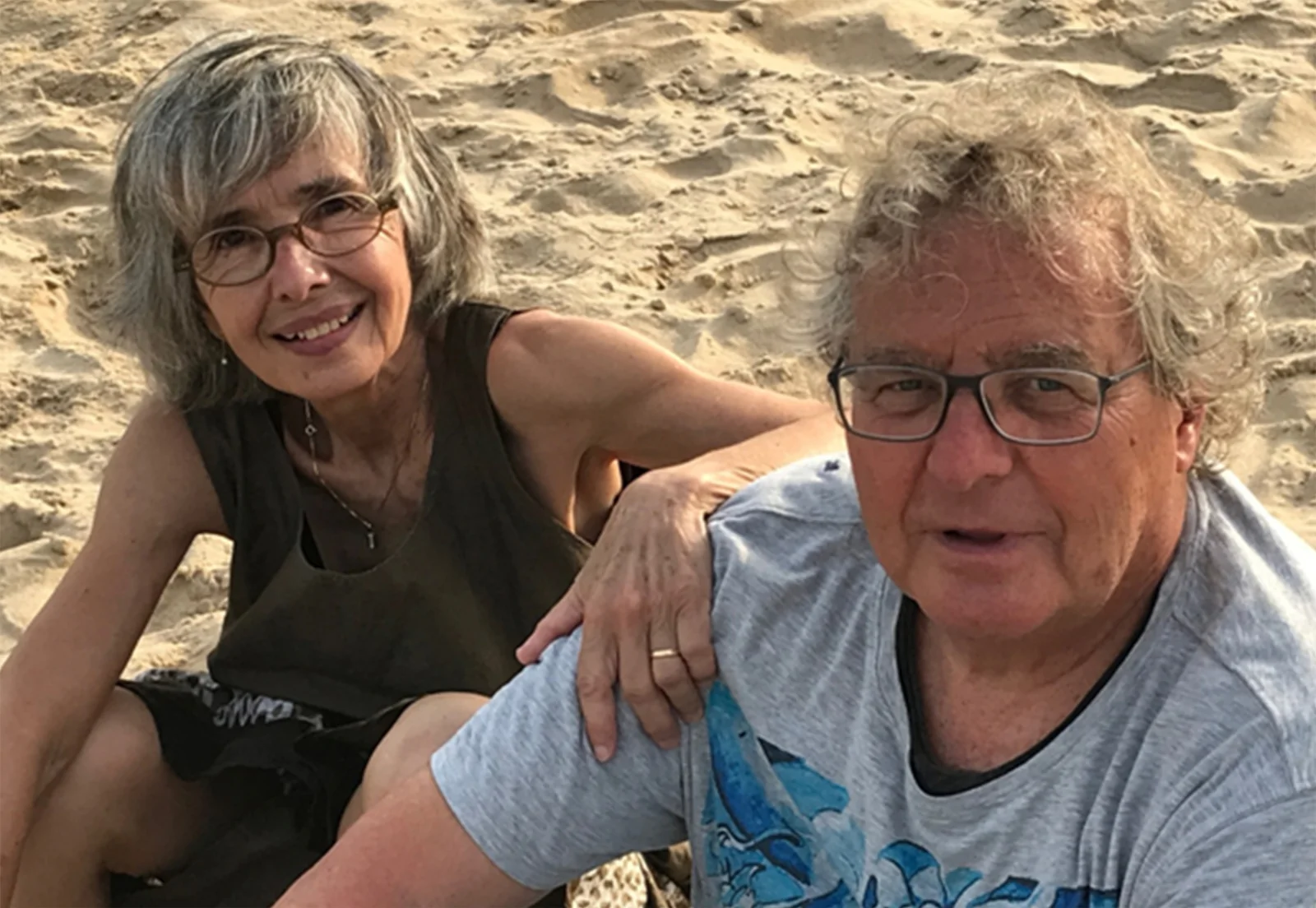
**Figure 8.** The author and his wife, Roser Savall López-Reynals, in 2026.

We have also had a long-standing collaboration with the Vicente Ferrer Foundation, headquartered in Anantapur, in the Indian state of Andhra Pradesh (despite the shared name, we have no family relationship).

Each of these trips could fill several papers with anecdotes and engaging experiences. Although I may not have the opportunity to share these adventures right now, they have left me with invaluable and often emotionally challenging, yet enriching memories.

I have also enjoyed cheerful vacations with family and friends during my travels. For me, traveling isn't merely about adding new locations to a checklist or about acquiring touristic knowledge. Each trip is an opportunity to appreciate the world's diversity and discover the beauty in people, their creations, and the places they inhabit.

I am also pleased to have contributed new knowledge and shared it in hundreds of papers and books. My publications are challenging to compile into a single list because of the different spellings of my name in Spanish or Catalan and the various affiliations I've used over time. The accompanying **supplementary table** is a complete register of journal publications and a partial list of book chapters.

One might think that I feel satisfied and proud of my accomplishments. However, that is only partly the case; I feel somewhat differently. Instead, I recognize that I have experienced significantly more blessings than most living people. I represent a small, privileged segment of our species. This disparity leads me to question whether luck should influence my sense of fairness and moral responsibility. The main constraint now is the limited time I have left to give something back to society.

## Questions without answers

One might wonder how the events of my life have shaped my current perceptions of the world. I have hesitated to express these interpretations because they represent an intimate part of me, and I'm unsure if they would interest anyone. Initially, my goal was not to present a false image of myself, but leaving this aspect unaddressed would make these reflections feel incomplete.

I have many matters left to explain, and I have not been able to resolve them. I still don't understand the meaning of life, and I don't intend to analyze it. I've already pondered it several times without reaching any conclusion. I am neither a religious believer nor an atheist; I am probably agnostic because I recognize that I do not have the capacity to understand the tremendous complexity, and I am not drawn to faith.

Regarding the universe and the program of life on our planet, I rate it a beautiful system; however, I struggle to understand the meaning behind billions of deaths, suffering, and the perseverance of living beings—from unicellular organisms to complex plants and animals—over millions of years. It is an outrageous excess to support such design. I see the evolution of humans—who pride themselves on being the pinnacle of evolution and believe everything else was created for their benefit—as a remarkable journey. However, this journey also includes a primary, alarming, and insatiable capacity to destroy nearly everything, including humanity itself. In this regard, we forget that we are a part of the world, not its masters – a disturbing illusion.

As a thought experiment, we can ask rhetorically, "Why do we live?" Because, like all living beings, we are genetically programmed to live. "For what do we live?" Unlike other mammals, insects, and plants, we have a certain margin of choice: we can decide what we want within the limitations of what we can do. Here lies the free will of human beings. Together, it is a mixture of determinism and freedom; a spark of hope.

As I grow older, I find myself facing more uncertainties than certainties regarding the big questions we humans ask ourselves. Nevertheless, I remain curious about what the world will be like in the future. Perhaps some ancient beliefs can still offer hope. The concept of reincarnation, for example, provides believers with an opportunity to rectify past mistakes and attain spiritual growth. Additionally, it could satisfy the curiosity of those who have lingering questions, allowing us a brief return to Earth every century or two.

One might conclude that my thoughts lead to a sense of universal loneliness. And so they do. That is why I have chosen to downplay the significance of my musings.

A few days ago, we were reading in bed, as we almost always do. My wife, who is now rereading some Spanish Golden Age dramatists and poets, recited aloud the monologue of Segismundo from the second act of *Life is a Dream* (1635) by Pedro Calderón de la Barca. Here is my free translation of the ending:

What is life? A frenzy.
What is life? An illusion,
a shadow, a fiction,
and the greatest good is small;
that all of life is a dream,
and dreams are dreams.

Together we enjoyed returning to the third act of the drama. Immersing ourselves in a beautiful work of art, sharing a pleasant activity, and living in harmony with loved ones are among life's greatest achievements and rewards.

Good night.

## Abbreviations

**BRI**: Biomedical Research Institute or **IIB**: Instituto de Investigación Biomédica; **CNECS**: Consejo Nacional de especialidades en ciencias de la salud; National Council of Specialties in Health Sciences; **CIBER**: Centro de Investigación Biomédica en Red; Network Biomedical Research Center; **CIBERNED**: Centro de Investigación Biomédica en Red de Enfermedades Neurodegenerativas; Network Biomedical Research Center on Neurodegenerative Diseases; **CIEN**: Centro de Investigación de Enfermedades Neurológicas; Research Center of Neurological Diseases; **CNIC: **Spanish National Center for Cardiovascular Research**; CNIO: **Centro Nacional de Investigaciones Oncológicas**; CSIC**: Consejo Superior de Investigaciones Científicas; Higher Council for Scientific Research; **FIS**: Fondo de Investigación Sanitaria del Instituto Carlos III; Health Research Fund; **HUB**: Hospital Príncipes de España, Ciudad Sanitaria de Bellvitge, Hospital Universitario de Bellvitge: Bellvitge University Hospital; **ICS**: Institut Català de la Salut, Generalitat de Catalunya; Catalan Institute of Health; **IDIBELL**: Instituto de Investigación Biomédica de Bellvitge; Biomedical Research Institute of Bellvitge; **INP**: Instituto de Neuropatología; Institute of Neuropathology; **INP-HUB**: laboratories at the HUB; **INP-ICO**: laboratories at the Hospital Duran i Reynals and Catalan Institute of Oncology (ICO); **INP-UB**: laboratories at the University of Barcelona campus Bellvitge; **ISCIII**: Instituto de Salud Carlos III, Ministerio de Salud; Carlos III Institute of Health; **OSSMA**: Office of Safety, Health, and Environment; **UAB**: Universidad Autónoma de Barcelona; Autonomous University of Barcelona ; **UB**: Universidad de Barcelona; University of Barcelona

## Conflict of interest statement

The author declares no conflict of interest.

## Supplementary Material

Supplementary material

## References

[Arnsten2021] Arnsten AFT, Datta D, Del Tredici K, Braak H. Hypothesis: Tau pathology is an initiating factor in sporadic Alzheimer's disease. Alzheimer’s Dement. 2021;17:115–124. 10.1002/alz.12192 PMC7983919 33075193

[Ferrer2022] Ferrer I. Alzheimer’s disease is an inherent, natural part of human brain aging: an integrated perspective. Free Neuropathology 2022;3:17. 10.17879/freeneuropathology-2022-3806 PMC10209894 37284149

[Ferrer2023a] Ferrer I. The unique neuropathological vulnerability of the human brain to aging. Aging Res Reviews 2023a;87:101916. 10.1016/j.arr.2023.101916 36990284

[Ferrer2023b] Ferrer I. Hypothesis review: Alzheimer’s overture guidelines. Brain Pathol 2023b;33:e13122. 10.1111/bpa.13122 PMC9836379 36223647

[Ferrer2024] Ferrer I. Amyloid-β Pathology Is the Common Nominator Proteinopathy of the Primate Brain Aging. J Alzheimers Dis 2024;100(s1):S153–S164. 10.3233/JAD-240389 PMC11380266 39031364

