# Supplementary material for: Newton’s cradle philosophy and stress relief or what goes up must come down
Source: Free Neuropathol. 2026 Oct 21;7:26. doi: 10.17879/freeneuropathology-2026-10294 (PMC13712332; doi:10.17879/freeneuropathology-2026-10294)
Supplement: Supplementary file 1 [file freeneuropathol-07-26-10294-s1.pdf]

Supplementary material

Supplementary Articles

1. Ferrer I, Fernández-Alvarez E
Lisencefalia: agiria. Un estudio con el método de Golgi.
J Neurol Sci 34: 109-120 (1976). Spanish

2. Ferrer I, Navarro C
Multicystic encephalopathy of infancy.
J Neurol Sci 38: 179-189 (1978)

3. Ferrer I, Fernández-Alvarez E, Pérez-Serra J, Arribas MA
New cases of heredoataxia with slow eye movements. Phenomenon of dominance in heredoataxia.
Rev Clin Esp 150:299-302 (1978). Spanish

4. Ferrer I, Vila Torres J, Fernández Alvarez E, Camino A, González Sastre F, Pampols T, Chavas A, Sabater J, Giner P
Gm1 gangliosidosis types 1 and 2.
Med Clin (Barc) 72:179-184 (1979). Spanish

5. Ferrer I, Fernández Alvarez E, González A, Pineda M
Congenital oculomotor apraxia.
Rev Clin Esp 153:471-473 (1979). Spanish

6. Ferrer I, Picatoste F, Rodergas E, García A, Sabria J, Blanco I
Histamine and mast cells in developing rat brain.
J Neurochem 32: 587-92 (1979)

7. Ferrer I, Isamat F, Acebes J
A Golgi and electronmicroscopic study of a dysplastic gangliocytoma of the cerebellum.
Acta Neuropathol 47: 163-165 (1979)

8. Sarmiento J, Ferrer I, Pons L, Ferrer E
Cerebral mixed tumour: osteo-chondrosarcoma-glioblastoma.
Acta Neurochir 50: 335-341 (1979)

9. Saracíbar N, Ferrer I, Arbizu T, Aguilar M
Pellagroid encephalopathy in chronic alcoholism. Clinicopathological study.
Med Clin (Barc) 75:151-5 (1980). Spanish

10. Ferrer I, Sarmiento J
Nascent microglia in the developing brain.
Acta Neuropathol 50: 61-67 (1980)

11. Ferrer I, Sarmiento J
Reactive microglia in the developing brain.
Acta Neuropathol 50: 69-76 (1980)

12. Ferrer I, Arbizu T, Peña J, Peres-Serra J
A Golgi and ultrastructural study of a dominant form of Kufs disease.
J Neurol 222: 183-190 (1980)

Copyright: © 2026 The author(s). This is an open access article distributed under the terms of the Creative Commons Attribution 4.0 International License (https://creativecommons.org/licenses/by/4.0/), which permits unrestricted use, distribution, and reproduction in any medium, provided the original author and source are credited, a link to the Creative Commons license is provided, and any changes are indicated. The Creative Commons Public Domain Dedication waiver (https://creativecommons.org/publicdomain/zero/1.0/) applies to the data made available in this article, unless otherwise stated.

13. Savall R, Ferrándiz C, Ferrer I, Peyrí J
Idiopathic guttate hypomelanosis.
Br J Dermatol 103: 635-642 (1980)

14. Pou-Sarradell A, Aguilar M, Soler L, Ferrer I
Myopathie congénitale benigne avec préponderance des fibres de type I et des rares cores chez la mère asymptomatique.
Rev Neurol (Paris) 136: 853-862 (1980) French

15. Arbizu T, Graus F, Santamaría J, Ferrer I, Peres Serra J
Peripheral neuropathy and insulinoma.
Med Clin (Barc) 76:271-4 (1981). Spanish

16. Savall R, Ferrer I
Mast cells in the skin of rats during development.
Med Cutan Ibero Lat Am 9:345-350 (1981). Spanish

17. Ferrer I, Sarmiento J
Lipid inclusions in the telencephalic neuroglia of the developing rat.
J Hirnforsch 22: 307-312 (1981)

18. Ferrer I, Costa F, Grau-Veciana JM
Creutzfeldt-Jakob disease. A Golgi study.
Neuropathol Appl Neurobiol 7: 237-242 (1981)

19. Ferrer I
Schilder's disease. A study of the cerebral cortex with the Golgi method.
Child's Brain 8: 294-298 (1981)

20. Ferrer I, Martínez-Matos JA
Development of non-pyramidal neurons in the rat sensory- motor cortex during the fetal and early postnatal periods.
J Hirnforsch 22: 555-562 (1981)

21. López-Pousa S, Ferrer I, Domenech JM
Angiogenic activity in the CSF in human malignancies.
Experientia 37: 41 (1981)

22. Ferrer I, Ferrándiz C, Henkes J, Savall R, Peyrí J
Skin pigmentation caused by amiodarone. A sequential study.
Med Clin (Barc) 79: 355-39 (1982). Spanish

23. Ferrer I, Fábregues I, Palacios G
An autoradiographic study of methyl-azoxy-methanol acetate-induced cortical malformation.
Acta Neuropathol 57: 313-315 (1982)

24. Roig C, Ferrer I
Bleeding in siringobulbia. A fatal complication
Eur Neurol 21: 189-193 (1982)

25. López-Pousa S, Vich JM, Ferrer I, Domenech JM, López-Pousa A, Real F
Angiogenic activity in fluid samples from tumoral patients.
Cáncer 52: 1365-1368 (1983)

Copyright: © 2026 The author(s). This is an open access article distributed under the terms of the Creative Commons Attribution 4.0 International License (https://creativecommons.org/licenses/by/4.0/), which permits unrestricted use, distribution, and reproduction in any medium, provided the original author and source are credited, a link to the Creative Commons license is provided, and any changes are indicated. The Creative Commons Public Domain Dedication waiver (https://creativecommons.org/publicdomain/zero/1.0/) applies to the data made available in this article, unless otherwise stated.

26. Ferrer I, Ribalta T, Digón E, Acebes J
Cerebral ganglioglioma. A Golgi study.
Virch Archiv (Pathol Anat) 400: 69-75 (1983)

27. Fábregues I, Ferrer I
Abnormal perisomatic structures in non-pyramidal neurons in Down syndrome.
Neuropathol Appl Neurobiol 9: 165-170 (1983)

28. Ferrer I, Aymamí M, Rovira A, Grau-Veciana JM
Growth of abnormal neurites in atypical Alzheimer's disease. A study with the Golgi method.
Acta Neuropathol 59: 167-170 (1983)

29. Graus F, Ferrer I, Lamarca J
Mixed carcinomatous neuropathy in patients with lung cancer and lymphoma.
Acta Neurol Scand 68: 40-48 (1983)

30. Ferrer I, Fábregues I, Pineda M, Fernández-Alvarez E, Cusí V, Vila J
Diagnosis of infantile neuroaxonal dystrophy by conjunctival biopsy.
Neuropaediatrics 14: 53-55 (1983)

31. Ferrer I, Digón E, Canto J, Sastre S, Sancho S
Morphological changes in the cerebral cortex of mice subjected to environments enriched or impoverished in sensory stimuli and their later recovery.
Arch Neurobiol (Madr) 46:177-182 (1983). Spanish

32. Castell C, Morato J, Ferrer I, Lucas T, Sarmiento J, Porta M, Prat J
Hypothalamic plasma cell granuloma causing panhypopituitarism.
Rev Clin Esp 170:141-144 (1983). Spanish

33. Soler L, Pou A, Fossas P, Bayes A, Tolosa E, Reixach R, Montserrat L, Ferrer I
Diabetic amyotrophy. An infrequent form of diabetic neuropathy.
Rev Clin Esp 168:361-364 (1983). Spanish

34. Galván P, Reixach R, Potes E, Quera MA, Subirana A, Ferrer I, Subirana M
Recurring polyradiculoneuritis. Report of an anatomo-clinical case.
Arch Neurobiol 46: 257-264 (1983). Spanish

35. Ferrer I, Xumetra A, Santamaría J
Cerebral malformation induced by prenatal X-irradiation: an autoradiographic and Golgi study.
J Anat 138: 81-93 (1984)

36. Fábregues I, I Ferrer, V Cusí, C Bugié, E Fernández- Alvarez, I Feijoó
Fine structure based on the Golgi method of the abnormal cortex and heterotopic nodules in pachigyria.
Brain Develop 6: 317-322 (1984)

37. Fábregues I, Ferrer I, Cahuana A, Giner P
The cerebral cortex of the newborn guinea pig. A quantitative Golgi study of the layer V pyramidal cell.
J Hirnforsch 25: 291-294 (1984)

38. Masó E, Ferrer I, Herraiz J, Roquer J, Serrano S
Leigh's syndrome in an adult.
J Neurol 231: 253-257 (1984)

freeneuropathology.org

Copyright: © 2026 The author(s). This is an open access article distributed under the terms of the Creative Commons Attribution 4.0 International License (https://creativecommons.org/licenses/by/4.0/), which permits unrestricted use, distribution, and reproduction in any medium, provided the original author and source are credited, a link to the Creative Commons license is provided, and any changes are indicated. The Creative Commons Public Domain Dedication waiver (https://creativecommons.org/publicdomain/zero/1.0/) applies to the data made available in this article, unless otherwise stated.

39. Ferrer I, Fábregues I, Pineda M, Gracia T, Ribalta T
A Golgi of cerebellar atrophy in human chronic alcoholism.
Neuropathol Appl Neurobiol 10: 245-253 (1984)

40. Ferrer I
A Golgi analysis of unlayered polymicrogyria.
Acta Neuropathol 65: 69-76 (1984)

41. Ferrer I, Fábregues I, Coll J, Ribalta T, Rives A
Tuberous sclerosis. A Golgi study of cerebellar tuber.
Clin Neuropathol 3: 47-51 (1984)

42. Pineda M, González A, Fábregues I, Fernández-Alvarez E, Ferrer I
Familial agenesis of the corpus callosum with hypothermia and apneic spells.
Neuropaediatrics 15: 63-67 (1984)

43. Zarranz JJ, Ferrer I
A 53-year-old woman with depressive syndrome, behavioural change and disorientation.
Med Clin (Barc) 83:214-21 (1984). Spanish

44. Roig C, Blesa R, Ferrer I
A single metastasis in the brain stem.
Rev Clin Esp 172:357-60 (1984). Spanish

45. Fábregues I, Ferrer I, Gairí JM, Cahuana A, Giner P
Effects of prenatal exposure to ethanol on the maturation of the pyramidal neurons in the cerebral cortex of the guinea-pig. A quantitative Golgi study.
Neuropathol Appl Neurobiol 11: 291-298 (1985)

46. Ferrer I
Vascular dementia.
Neurologia 1:91-93 (1986). Spanish

47. Ferrer I, Cusí V, Liarte A, Campistol J
A Golgi study of the polymicrogyric cortex in Aicardi syndrome.
Brain Develop 8: 518-525 (1986)

48. Ferrer I, Fábregues I, Reiriz J, Galofré E
Decreased numbers of dendritic spines on cortical pyramidal neurons in human chronic alcoholism.
Neurosci Lett 69: 115-119 (1986)

49. López-Tejero D, Ferrer I, Llobera M, Herrera E
Effects of prenatal ethanol exposure on physical growth, sensory reflex maturation and brain development in the rat.
Neuropathol Appl Neurobiol 12: 251-260 (1986)

50. Ferrer I, Fábregues I, Condom E
A Golgi study of the sixth layer of the cerebral cortex. I.- The lissencephalic brain of Rodentia, Lagomorpha, Insectivora and Chiroptera.
J Anat 145: 217-234 (1986)

Copyright: © 2026 The author(s). This is an open access article distributed under the terms of the Creative Commons Attribution 4.0 International License (https://creativecommons.org/licenses/by/4.0/), which permits unrestricted use, distribution, and reproduction in any medium, provided the original author and source are credited, a link to the Creative Commons license is provided, and any changes are indicated. The Creative Commons Public Domain Dedication waiver (https://creativecommons.org/publicdomain/zero/1.0/) applies to the data made available in this article, unless otherwise stated.

51. Ferrer I, Fábregues I, Condom E
A Golgi study of the sixth layer of the cerebral cortex. II.- The gyrencephalic cortex of Carnivora, Artiodactyla and Primates.
J Anat 146: 87-104 (1986)

52. Ferrer I, Galofré E, Soler T
Structure of the isolated cerebellum and related nuclei developed within the matrix of a mature ovarian teratoma.
Child's Nerv Syst 2: 266-269 (1986)

53. Ferrer I
Golgi study of the isocortex in an insectivore: the common European mole (*Erinaceus europaeus*).
Brain Behav Evol 29: 105-114 (1986)

54. Ferrer I, Ribalta T, Fábregues I, Pineda M, Cusí V
A Golgi study of the cerebellar malformation in 13 trisomy.
Clin Neuropathol 5: 53-59 (1986)

55. Ferrer I, Blesa R, Valls J, Grau JM
Motor-axonal form of Guillain-Barre syndrome.
Rev Clin Esp 178:356 (1986). Spanish

56. Ferrer I, Galofré E, Vázquez J, Pascual J, Vidal MJ, Sirvent J, Navarro C
Changes in the white matter of the brain in alcoholic malnutritional encephalopathies: Marfchiafava-Bignami disease.
Arch Neurobiol (Madr) 49:268-278 (1986). Spanish

57. Blanco I, Ferrer I, Toledo A, Sabria J, Picatoste F, Rodriguez R, Bradner R, Rodriguez J, Sentís M
Subcellular localization of brain mast cell histamine in developing rat.
Neurochem Int 11: 451-461 (1987)

58. Galofré E, Ferrer I, Fábregues I, López-Tejero D
Effects of prenatal ethanol exposure on dendritic spines of layer V pyramidal neurons in the somatosensory cortex of the rat.
J Neurol Sci 81: 185-195 (1987)

59. Ferrer I, Sirvent J, Manresa JM, Galofré E, Fernández-Alvarez E, Pineda M
Primary degeneration of the granular layer of the cerebellum (Norman type). A Golgi study.
Acta Neuropathol 75: 203-208 (1987)

60. Galofré E, Ferrer I
Development of dendritic spines in the V layer pyramidal neurons of the rat's somatosensory cortex. A qualitative and quantitative study.
J Hirnforsch 28: 653-659 (1987)

61. Pou A, Russi A, Ferrer I, Galofré E, Escudero D
Maladie de Machado-Joseph dans une famille d'origine espagnole.
Rev Neurol (Paris) 143: 520-525 (1987)

62. Ferrer I, Fábregues I, Condom E
A Golgi study of the sixth layer of the cerebral cortex. III.- Neuronal changes during normal and abnormal cortical folding.
J Anat 152: 71-82 (1987)

63. Sabria J, Ferrer I, Toledo A, Sentís M, Blanco I
Effects of altered thyroid function on histamine levels and mast cell numbers in neonatal rat brain.
J Pharmacol Exp Therap 240: 612-616 (1987)

Copyright: © 2026 The author(s). This is an open access article distributed under the terms of the Creative Commons Attribution 4.0 International License (https://creativecommons.org/licenses/by/4.0/), which permits unrestricted use, distribution, and reproduction in any medium, provided the original author and source are credited, a link to the Creative Commons license is provided, and any changes are indicated. The Creative Commons Public Domain Dedication waiver (https://creativecommons.org/publicdomain/zero/1.0/) applies to the data made available in this article, unless otherwise stated.

64. Ferrer I, Galofré E
Dendritic spine anomalies in fetal alcohol syndrome.
Neuropaediatrics 18: 161-163 (1987)

65. Ferrer I, Sancho S
Non-pyramidal neurons of layers I-III in the dog's cerebral cortex (parietal lobe).
Acta Anat 129: 43-52 (1987)

66. Ferrer I, Campistol J, Tobeña L, Cusí V, Galofré E, Prat J
Degenerescence systematisee optico-cochleo-dentelee.
J Neurol 234: 416-420 (1987)

67. Ferrer I
The basic structure of the neocortex in Insectivorous bats.
J Hirnforsch 28: 237-243 (1987)

68. Martínez Matos JA, Vila Duplá MJ, Montero J, Ferrer I, Baguñá J, Peres Serra J
Forbes-Cori disease: reversible worsening in a case with non-evolutive neuromuscular disorder.
Neurologia 2:75-80 (1987). Spanish

69. Pons JV, Pac V, Vidaller A, Moga I, Ferrer I, Martinez JA
Polyangiitis overlap syndrome: a useful subgroup.
Am J Med 83:197-198 (1987)

70. Ferrer I, Herrera M, Ciria L, Matamoros N, Galofré E, Pons Irazábal L
Encephalopathy of the acquired immunodeficiency síndrome (AIDS) in childhood. A clinicopathological study.
Neurologia 2:180-6 (1987). Spanish

71. Catalá I, Ferrer I, Galofré E, Fábregues I
Decreased numbers of dendritic spines on cortical pyramidal neurons in dementia. A quantitative Golgi study in biopsy samples.
Human Neurobiol 6: 255-259 (1988)

72. Ferrer I, Galofré E, López-Tejero D, Llobera M
Morphological recovery of hippocampal pyramidal neurons in the adult rat exposed in utero to ethanol.
Toxicology 48: 191-197 (1988)

73. Matías Guiu X, Ferrer I, Molet J, Prat J
Meningo-angiomatosis. A case report.
Br J Neurosurg 2: 637-640 (1988)

74. Ferrer I, Kulisewski J, Vázquez J, González G, Pineda M
Purkinje cells in degenerative diseases of the cerebellum and its connections. A Golgi study.
Clin Neuropathol 7: 22-28 (1988)

75. Ferrer I, Perera M
Structure and nerve cell organization in the cerebral cortex of the dolphin *Stenella coeruleoalba*. A Golgi study with special attention to the primary auditory area.
Anat Embryol 178: 161-173 (1988)

76. Kulisewski J, Martí MJ, Ferrer I, Tolosa E
Meige syndrome: Neuropathology of a case.
Mov Disord 3: 170-175 (1988)

freeneuropathology.org

Copyright: © 2026 The author(s). This is an open access article distributed under the terms of the Creative Commons Attribution 4.0 International License (https://creativecommons.org/licenses/by/4.0/), which permits unrestricted use, distribution, and reproduction in any medium, provided the original author and source are credited, a link to the Creative Commons license is provided, and any changes are indicated. The Creative Commons Public Domain Dedication waiver (https://creativecommons.org/publicdomain/zero/1.0/) applies to the data made available in this article, unless otherwise stated.

77. Ferrer I, Vidaller A, Fernández de Sevilla A, Martínez-Matos JA, Montero J, Romagosa V
Peripheral neuropathy associated with angioimmunoblastic lymphadenopathy.
Clin Neurol Neurosurg 90: 159-162 (1988)

78. Tuñón T, Ferrer I, Gallego J, Delgado G
Leucodystrophy with pigmented glial and scavenger cells (Pigmentary type of orthochromatic leucodystrophy).
Neuropathol Appl Neurobiol 14: 337-344 (1988)

79. Ferrer I, Cusí V, Pineda M, Galofré E, Vila J
Focal dendritic swellings in Purkinje cells in mucopolysaccharidoses I, II and III: A Golgi and electron microscopical study.
Neuropathol Appl Neurobiol 14: 315-323 (1988)

80. Ferrer I, Hernández-Martí E, Bernet E, Galofré E
Formation and growth of the cerebral convolutions. I.- Postnatal development of the median-suprasylvian gyrus and adjoining sulci in the cat.
J Anat 160: 89-100 (1988)

81. López-García C, Mollowny A, García Verdugo A, Ferrer I
Delayed postnatal neurogenesis in the cerebral cortex of lizards.
Devl Brain Res 45: 303-308 (1989)

82. Martinez JM, Barraquer-Bordas L, Ferrer I, Escartin AE, Rosich M, Barraquer-Feu ML
Anatomo-clinical study of Behçet syndrome with involvement of the central nervous system.
Rev Neurol (Paris) 144:130-135 (1988). French

83. Galofré E, Soler T, Martínez JM, Ferrer I
Neuropathological findings in patients subjected to orthotopic liver transplantation.
Neurologia 3:77-79 (1988). Spanish

84. Ferrer I, Hernández-Martí M, Bernet E, Calopa M
Formation and growth of the cerebral convolutions. II.-Cell death in the gyrus suprasylvius and adjoining sulci in the cat.
Devl Brain Res 45: 303-308 (1989)

85. García Verdugo JM, Llahí S, Ferrer I, López-García C
Postnatal neurogenesis in the olfactory bulbs of a lizard. A tritiated thymidine autoradiographic study.
Neurosci Lett 98: 247-252 (1989)

86. Ferrer I, García-Bach M, Aparicio MA, Acebes J, Twose J, Isamat F
Plasma cell granuloma of the hypothalamic region.
Acta Neurochir 99: 152-156 (1989)

87. Illa I, Graus F, Ferrer I, Enríquez J
Sensory neuropathy as the initial manifestation of primary biliary cirrhosis.
J Neurol Neurosurg Psychiatr 52: 1307 (1989)

88. Ferrer I, Galofré E, Fábregues I, López-Tejero D
Effects of chronic ethanol consumption beginning at adolescence: increased numbers of dendritic spines on cortical pyramidal cells in the adulthood.
Acta Neuropathol 78: 528-532 (1989)

freeneuropathology.org

Copyright: © 2026 The author(s). This is an open access article distributed under the terms of the Creative Commons Attribution 4.0 International License (https://creativecommons.org/licenses/by/4.0/), which permits unrestricted use, distribution, and reproduction in any medium, provided the original author and source are credited, a link to the Creative Commons license is provided, and any changes are indicated. The Creative Commons Public Domain Dedication waiver (https://creativecommons.org/publicdomain/zero/1.0/) applies to the data made available in this article, unless otherwise stated.

89. Ferrer I
Developmental aspects of the bat.
Neurosci Res 6: 573-580 (1989)

90. Podzamczer D, Bolao F, Pagerols X, Pasarín M, Ferrer I, Romeu A, Gudiol F, Fernández-Nogués F
Hairy leukoplakia: a new disease of the oral mucosa associated with infection by the human immunodeficiency virus.
Med Clin (Barc) 92:488-491 (1989). Spanish

91. Ferrer I, Serrano T, Soriano E
Naturally-occurring cell death in the subicular complex and hippocampus in the rat during development.
Neurosci Res 8: 60-66 (1990)

92. Graus F, Ferrer I
Analysis of neuronal antigen (Hu) expression in the developing rat brain detected by autoantibodies from patients with paraneoplastic encephalomyelitis.
Neurosci Lett 112: 14-18 (1990)

93. Matías-Guiu X, Alejo M, Soler T, Ferrer I, Noboa R, Bartumeus F
Cavernous angiomas of the cranial nerves.
J Neurosurg 73: 620-622 (1990)

94. Ferrer I, Martí E, Bella R, Serrano T, Twose J, Conesa G, Isamat F
Studies with the Golgi method in central gangliogliomas and dysplastic gangliocytoma of the cerebellum (Lhermitte-Duclos disease).
Histol Histopathol 5: 329-336 (1990)

95. Ferrer I, Gullotta F
Down's syndrome and Alzheimer's disease: dendritic spine counts in the hippocampus.
Acta Neuropathol 79: 680-685 (1990)

96. Ferrer I, Guionnet N, Cruz-Sánchez F, Tuñón T
Neuronal alterations in patients with dementia: a Golgi study on biopsy samples.
Neurosci Lett 114: 11-16 (1990)

97. Ferrer I, Bella R, Serrano T, Martí E, Guionnet N
Arteriosclerotic leucoencephalopathy in the elderly and its relation to white matter lesions in Binswanger's disease, multi-infarct encephalopathy and Alzheimer's disease.
J Neurol Sci 98: 37-50 (1990)

98. Vázquez-Cruz J, Martí-Vilalta JL, Ferrer I, Pérez-Gallofré A, Folch J
Progressing cerebral infarction in relation to plasma glucose in gerbils.
Stroke 21: 1621-1624 (1990)

99. Ferrer I, Bernet E, Soriano E, Del Rio T, Fonseca M
Naturally-occurring cell death in the cerebral cortex of the rat and removal of dead cells by transitory phagocytes.
Neuroscience 2: 451-458 (1990)

100. Ferrer I, Cruz-Sánchez F, Guionnet N, Tuñón T
A study of senile plaques with a combined method in brains of patients suffering from Alzheimer’s disease.
Arch Neurobiol (Madr) 53:222-226 (1990). Spanish

Copyright: © 2026 The author(s). This is an open access article distributed under the terms of the Creative Commons Attribution 4.0 International License (https://creativecommons.org/licenses/by/4.0/), which permits unrestricted use, distribution, and reproduction in any medium, provided the original author and source are credited, a link to the Creative Commons license is provided, and any changes are indicated. The Creative Commons Public Domain Dedication waiver (https://creativecommons.org/publicdomain/zero/1.0/) applies to the data made available in this article, unless otherwise stated.

101. Martínez Lacasa JT, Ferrer I, Burillo J, López Pousa S
Morphological differences in the vascularization of tuberculoma and tuberculous brain abscess.
Med Clin (Barc) 95:196 (1990). Spanish

102.Sierra A, Prat J, Romeu A, Martínez-Matos JA, Bella R, Ferrer I, Buendía E
Blood lymphocytes are sensitized to brachial plexus nerves in patients with neuralgic amyotrophy.
Acta Neurol Scand 83: 183-186 (1991)

103. Servitge O, Serón D, Ferrer I, Carrera M, Pagerols X, Peyrí J
Quantitative and morphometric analysis of Langerhans cells in non-exposed skin in renal transplant patients.
J Cutan Pathol 18: 106-111 (1991)

104. Cruz-Sánchez F, Martos JA, Rives A, Ribalta T, Ferrer I, Cardesa A
Adult type of leukodystrophy. Krabbe's disease?.
Clin Neurol Neurosurg 93: 217-222 (1991)

105. Cruz-Sánchez F, Ferrer I, Rossi ML
On the white matter changes in spongiform encephalopathy.
Dementia 2: 106-111 (1991)

106. Ferrer I, Soriano E, Martí E, Digón E, Reyners H, Gianfelici de Reyners E
Development of dendritic spines in the cerebral cortex of the micrencephalic rat following prenatal X-irradiation.
Neurosci Lett 125: 183-186 (1991)

107. Ferrer I, Roig C, Peiro G, Matías-Guiu X
Dementia of frontal lobe type and motor neuron disease. A Golgi study of the frontal cortex.
J Neurol Neurosurg Psychiatry 54: 932-934 (1991)

108. Ferrer I, Catalá I
Unlayered polymicrogyria: structural and developmental aspects.
Anat Embryol 184: 517-528 (1991)

109. Soriano E, del Rio JA, Martínez-Guijarro FJ, Ferrer I, López C
Immunocytochemical detection of 5'-Bromodeoxyuridine in Fluoro-Gold-labeled neurons: a simple technique to combine retrograde axonal tracing and neurogenetic characterization of neurons.
J Neurochem Cytochem 39: 1565-1570 (1991)

110. del Rio JA, Soriano E, Ferrer I
A transitory population of substance P-like immunoreactive neurons in the developing cerebral cortex of the mouse.
Devl Brain Res 64: 205-211 (1991)

111. Ferrer I, Soriano E, Martí E, Laforet E, Reyners H, Gianfelici de Reyners E
Naturally-occurring, postnatal cell death in the cerebral cortex of the micrencephalic rat induced by prenatal X-irradiation.
Neurosci Res 12: 446-451 (1991)

112. Ferrer I, Soriano E, Tuñón T, Fonseca M, Guionnet N
Parvalbumin-immunoreactive neurons in normal human temporal neocortex and in patients with Alzheimer's disease.
J Neurol Sci 106: 135-141 (1991)

113. Ferrer I, Saracibar N, González G
Spongiform encephalopathy and multisystemic degeneration.
Neurologia 6:29-33 (1991). Spanish

Copyright: © 2026 The author(s). This is an open access article distributed under the terms of the Creative Commons Attribution 4.0 International License (https://creativecommons.org/licenses/by/4.0/), which permits unrestricted use, distribution, and reproduction in any medium, provided the original author and source are credited, a link to the Creative Commons license is provided, and any changes are indicated. The Creative Commons Public Domain Dedication waiver (https://creativecommons.org/publicdomain/zero/1.0/) applies to the data made available in this article, unless otherwise stated.

114. Ferrer I, Tuñón T, Soriano E, del Rio A, Iraizoz I, Fonseca M, Guionnet N
Calbindin immunoreactivity in normal human temporal cortex.
Brain Res 572: 33-41 (1992)

115. Ferrer I, Zújar MJ, Admella C, Alcántara S
Parvalbumin and calbindin immunoreactivity in the cerebral cortex of the hedgehog.
J Anat 180: 165-174 (1992)

116. Cruz-Sánchez F, Marín C, Rossi M, Cardozo A, Ferrer I, Tolosa E
Ubiquitin in cerebral amyloid angiopathy.
J Neurol Sci 112: 46-50 (1992)

117. Cruz-Sánchez F, García-Bachs M, Rossi ML, Rodríguez Prados S, Ferrer I, Coakham HB, Ferreres JC, Figols J, Palacín A
Epithelial differentiation in gliomas, meningiomas and choroid plexus papillomas.
Virchows Archiv B Cell Pathol 62: 25-34 (1992)

118. Ferrer I, Soriano E, del Rio JÁ, Alcántara S, Auladell C
Cell death and removal in the cerebral cortex during development.
Progr Neurobiol 39: 1-43 (1992)

119. Soriano E, del Rio JA, Ferrer I, Auladell C, de Lecea L, Alcántara S
Late appearance of parvalbumin-immunoreactive neurons in the rodent cerebral cortex does not follow an "inside-out" sequence.
Neurosci Lett 142: 147-150 (1992)

120. Ferrer I, Pineda M, Tallada M, Oliver B, Russi A, Oller L, Noboa R, Zújar MJ, Alcántara S
Abnormal local-circuit neurons in epilepsia partialis continua associated with focal cortical dysplasia.
Acta Neuropathol 83: 647-652 (1992)

121. Ferrer I
Dementia of frontal lobe type.
Behav Neurol 5: 87-96 (1992)

122. Tuñón T, Insausti R, Ferrer I, Sobreviela T, Soriano E
Parvalbumin and calbindin D-28k in the human entorhinal cortex. An immunohistochemical study.
Brain Res 589: 24-32 (1992)

123. del Rio JA, Soriano E, Ferrer I
Development of GABA-immunoreactivity in the neocortex of the mouse.
J Comp Neurol 326: 501-526 (1992)

124. Ferrer I
The effect of cycloheximide on natural and X-ray-induced cell death in the developing cerebral cortex.
Brain Res 588: 351-357 (1992)

125. Olivé M, Ferrer I, Arbizu T, Calopa M, Ferrer X, Peres J
Polymicrogyria and ulegyria. Diagnosis by magnetic resonance.
Neurologia 7:117-119 (1992). Spanish.

126. Arbizu T, Espino A, Aceves J, Espinet H, Ferrer I
Vertebral hemangioblastoma: an atypical localization.
Neurologia 7:73-76 (1992). Spanish

Copyright: © 2026 The author(s). This is an open access article distributed under the terms of the Creative Commons Attribution 4.0 International License (https://creativecommons.org/licenses/by/4.0/), which permits unrestricted use, distribution, and reproduction in any medium, provided the original author and source are credited, a link to the Creative Commons license is provided, and any changes are indicated. The Creative Commons Public Domain Dedication waiver (https://creativecommons.org/publicdomain/zero/1.0/) applies to the data made available in this article, unless otherwise stated.

127. Sierra A, Ferrer I, Arbizu T, Buendía E
Multiple sclerosis: IL2, IFN-tau, and PGE2 secretion in mononuclear cell cultures stimulated by PHA and the effect of these cytokines on oligodendroglial cells.
Neurologia 7:247-53 (1992). Spanish

128. Olivé M, Ferrer I, Jauma S, Montero J, Martínez-Matos JA
Centronuclear myopathy.
Neurologia 8:122-124 (1993). Spanish

129. Podzamczer D, Martos A, Ferrer I, Santin M, Gudiol FHepatic and pulmonary pneumocystosis during primary profilaxis for Pneumocystis carinii pneumonia with dapsone/pyrimethamine.
Clin Infect Dis 16:171 (1993). Spanish

130. Ferrer I, Alcántara S, Martí E
A four-layered "lissencephalic" cortex induced by prenatal X-irradiation in the rat.
Neuropathol Appl Neurobiol 19: 74-81 (1993)

131. Ferrer I, Alcántara S, Zújar MJ, Cinós C
Structure and pathogenesis of cortical nodules induced by prenatal X-irradiation in the rat.
Acta Neuropathol 85: 205-212 (1993)

132. Ferrer I, Santamaría J, Alcántara S, Zújar MJ, Cinós C
Neuronal ectopic masses induced by prenatal irradiation in the rat.
Virchows Arch A Pathol Anat 422: 1-6 (1993)

133. Ferrer I, Alcántara S, Catalá I, Zújar MJ
Experimentally-induced laminar necrosis, status verrucosus, focal cortical dysplasia reminiscent of microgyria, and porencephaly in the rat.
Exp Brain Res 94: 261-269 (1993)

134. Ferrer I, Serrano T, Alcántara S, Tortosa A, Graus F
X-ray-induced cell death in the developing hippocampal complex involves neurons and requires protein synthesis.
J Neuropathol Exp Neurol 52: 370-378 (1993)

135. Ferrer I, Isamat F, López-Obarrio L, Conesa G, Rimbau J, Alcántara S, Español I, Zújar MJ
Parvalbumin and calbindin D-28K immunoreactivity in central ganglioglioma and dysplastic gangliocytoma of the cerebellum.
J Neurosurg 78: 133-137 (1993)

136. Alcántara S, Ferrer I, Soriano E
Postnatal development of parvalbumin and calbindin D28K immunoreactivities in the cerebral cortex of the rat.
Anat Embryol 188: 63-73 (1993)

137. Ferrer I, Tuñón T, Soriano E, del Rio JA, Iraizoz I, Fonseca M, Guionnet N
Calbindin D28k immunoreactivity in the temporal neocortex in patients with Alzheimer's disease.
Clin Neuropathol 12: 53-58 (1993)

138. Fueyo J, Ferrer I, Vallderiola F, Graus F
The expression of a neuronal nuclear antigen (Ri) recognized by the human anti-Ri autoantibody in the developing rat nervous system.
Neurosci Lett 162: 141-144 (1993)

Copyright: © 2026 The author(s). This is an open access article distributed under the terms of the Creative Commons Attribution 4.0 International License (https://creativecommons.org/licenses/by/4.0/), which permits unrestricted use, distribution, and reproduction in any medium, provided the original author and source are credited, a link to the Creative Commons license is provided, and any changes are indicated. The Creative Commons Public Domain Dedication waiver (https://creativecommons.org/publicdomain/zero/1.0/) applies to the data made available in this article, unless otherwise stated.

139. Seress L, Gulyas A, Ferrer I, Tuñón T, Soriano E, Freund TF
Distribution, morphological features, and synaptic connections of parvalbumin- and calbindin D28k-immunoreactive neurons in the human hippocampal formation.
J Comp Neurol 337: 208-230 (1993)

140. Cequier A, Maurí J, Soler T, Ferrer I, Sabaté X, Esplugas E
Cyclic changes in pulmonary wedge v waves in dilated cardiomyopathy.
Eur Heart J 14: 993-996 (1993)

141. Ferrer I, Zújar MJ, Rivera R, Soria M, Vidal A, Casas R
Parvalbumin-immunoreactive dystrophic neurites and aberrant sprouts in the cerebral cortex of patients with Alzheimer's disease.
Neurosci Lett 158: 1663-166 (1993)

142. Tortosa A, Ferrer I
Parvalbumin immunoreactivity in the hippocampus of the gerbil after transient forebrain ischaemia: a qualitative and quantitative sequential study.
Neuroscience 55: 33-43 (1993)

143. Munujos P, Vendrell M, Ferrer I
Proto-oncogene c-fos induction in thiamine-deficient encephalopathy. Protective effects of nicardipine on pyrithiamine-induced lesions.
J Neurol Sci 118: 175-180 (1993)

144. Fonseca M, Soriano E, Ferrer I, Martinez A, Tuñón T
Chandelier cell axons identified by parvalbumin-immunoreactivity in the normal human temporal cortex and in Alzheimer's disease.
Neuroscience 55: 1107-1116 (1993)

145. Ferrer I, Tuñón T, Serrano T, Casas R, Alcántara S, Zújar MJ, Rivera RM
Calbindin D-28k and parvalbumin immunoreactivity in the frontal cortex in patients with frontal lobe dementia of non-Alzheimer type associated with amyotrophic lateral sclerosis.
J Neurol Neurosurg Psychiatr 56: 257-21 (1993)

146. Ferrer I, Pumarola M, Rivera R, Zújar MJ, Cruz-Sánchez F, Vidal S
Primary central white matter degeneration in old dogs.
Acta Neuropathol 86: 172-175 (1993)

147. Ferrer I
Experimentally induced cortical malformations in rats.
Child's Nerv Syst 9: 403-407 (1993)

148. Condom E, Vidal A, Rota R, Graus F, Dalmau J, Ferrer I
Paraneoplastic intestinal pseudo-obstruction associated with high titres of Hu autoantibodies.
Virchows Arch A Pathol Anat 423: 507-511 (1993)

149. Ferrer I, Vidal A, Martinez S, Tuñón T, Saracibar N
Widespread axonal damage in patients with adult orthochromatic leucodystrophy (simple type, pigment type).
Neurodegeneration 2: 249-252 (1993)

Copyright: © 2026 The author(s). This is an open access article distributed under the terms of the Creative Commons Attribution 4.0 International License (https://creativecommons.org/licenses/by/4.0/), which permits unrestricted use, distribution, and reproduction in any medium, provided the original author and source are credited, a link to the Creative Commons license is provided, and any changes are indicated. The Creative Commons Public Domain Dedication waiver (https://creativecommons.org/publicdomain/zero/1.0/) applies to the data made available in this article, unless otherwise stated.

150. Ferrer I, Casas R, Rivera R
Parvalbumin-immunoreactive cortical neurons in Creutzfeldt-Jakob disease.
Ann Neurol 34: 864-866 (1993)

151. Tortosa A, Rivera R, Ambrosio S, Bartrons R, Ferrer I
Fructose-1,6-bisphosphate fails to ameliorate delayed neuronal death in the CA1 area after transient forebrain ischaemia in gerbils.
Neuropharmacology 32: 1367-1371 (1993)

152. Ferrer I, Serrano T, Rivera R, Olivé M, Zújar MJ, Graus F
Radiosensitive populations and recovery in x-ray-induced apoptosis in the developing cerebellum.
Acta Neuropathol 86: 491-500 (1993)

153. Díaz-Pérez C, Pérez-Tomás C, Domingo J, Ferrer I
Immunohistochemical localization of transforming growth factor-alpha in choroid plexus of the rat and chicken.
Neurosci Lett 164: 44-46 (1993)

154. Tortosa A, Rivera R, Ferrer I
Dose-related effects of cycloheximide on delayed neuronal death in the gerbil hippocampus after bilateral transitory forebrain ischemia.
J Neurol Sci 121: 10-17 (1994)

155. Ferrer I, Genís D, Dávalos A, Bernadó L, Sant F, Serrano T
The Purkinje cell in olivopontocerebellar atrophy. A Golgi and immunohistochemical study.
Neuropathol Appl Neurobiol 20: 38-46 (1994)

156. Olivé M, Sirvent J, Ferrer I
Congenital muscular dystrophy with distinct CNS involvement.
Neuropediatrics 25: 48-50 (1994)

157. Ferrer I, Tortosa A, Macaya A, Sierra A, Moreno D, Munell F, Blanco R, Squier W Evidence of nuclear DNA fragmentation following hypoxia- ischemia in the infant rat brain, and transient forebrain ischemia in the adult gerbil.
Brain Pathol 4: 115-122 (1994)

158. Espino A, Tortosa A, Bendahan G, Bartrons R, Calopa M, Ferrer I, Ambrosio S
Stereotaxic administration of 1-methyl-4-phenylpyridinium ion (MPP+) decreases striatal fructose 2,6-bisphosphate in rats.
J Neurochem 62: 1913-1920 (1994)

159. Olivé M, Ribera R, Ferrer I
Parvalbumin immunocytochemistry and calcium deposition in muscle fiber necrosis and subsequent regeneration following intramuscular injection of metoclopramide.
Muscle & Nerve 17: 494-499 (1994)

160. Ferrer I, Oliver B, Russi A, Casas R, Rivera R
Parvalbumin and calbindin-D28k immunocytochemistry in human neocortical epileptic foci.
J Neurol Sci 123: 18-25 (1994)

161. Fernández JM, Dávalos A, Ferrer I, Cervera C, Codina A, Ochoa JL
Acute sensory neuronopathy: Report of a child with remarkable clinical recovery.
Neurology 44: 762-764 (1994)

Copyright: © 2026 The author(s). This is an open access article distributed under the terms of the Creative Commons Attribution 4.0 International License (https://creativecommons.org/licenses/by/4.0/), which permits unrestricted use, distribution, and reproduction in any medium, provided the original author and source are credited, a link to the Creative Commons license is provided, and any changes are indicated. The Creative Commons Public Domain Dedication waiver (https://creativecommons.org/publicdomain/zero/1.0/) applies to the data made available in this article, unless otherwise stated.

162. Ferrer I, J Kulisewsky J, González G, Escartín A, Chivite A, Casas R
Parvalbumin-immunoreactive neurons in the cerebral cortex and striatum in Huntington's disease.
Neurodegeneration 3: 169-173 (1994)

163. Tortosa A, Ferrer I
Poor correlation between delayed neuronal death induced by transient forebrain ischemia, and immunoreactivity for parvalbumin and calbindin D-28k in developing gerbil hippocampus.
Acta Neuropathol. 88: 67-74 (1994)

164. Ferrer I, Borrás D
Effects of X-irradiation on glial cells in the developing rat brain.
Int J. Radiat Biol 66: 181-187 (1994)

165. Pol O, Ferrer I, Puig M
Diarrhea associated with intestinal inflammation increases the potency of Mu and Delta opioids on the inhibition of gastrointestinal transit in mice.
J Pharmacol Exp Therap 270: 386-391 (1994)

166. Soriano MA, Tortosa A, Planas AM, Rodriguez-Farré E, Ferrer I
Induction of HSP70 mRNA and HSP70 protein in the hippocampus of the developing gerbil following transient forebrain ischemia.
Brain Res 653: 191-198 (1994)

167. Alcántara S, Ferrer I
Postnatal development of parvalbumin immunoreactivity in the cerebral cortex of the cat.
J Comp Neurol 348: 133-149 (1994)

168. del Río JA, de Lecea L, Ferrer I, Soriano E
The development of parvalbumin immunoreactivity in the neocortex of the mouse.
Devl Brain Res 81: 247-259 (1994)

169. Olivé M, Ferrer I
Postnatal development of parvalbumin immunoreactivity in striated muscles of the rat.
Anat Embryol 190: 301-305 (1994)

170. Ferrer I, Tortosa A, Condom E, Blanco R, Macaya A, Planas A
Increased expression of bcl-2 immunoreactivity in the developing cerebral cortex of the rat.
Neurosci Lett 179: 13-16 (1994)

171. Olivé M, Ferrer I
Parvalbumin immunohistochemistry in denervated skeletal muscle.
Neuropathol Appl Neurobiol 20: 495-500 (1994)

172. Planas AM, Soriano MA, Ferrer I, Rodriguez-Farré E
Regional expression of inducible heat shock protein-70 mRNA in the rat brain following administration of convulsant drugs.
Mol Brain Res 27: 127-137 (1994)

173. Planas AM, Soriano MA, Ferrer I, Rodriguez-Farré E
Kainic acid inhibits protein amino acid incorporation in select rat brain regions.
NeuroReport 5: 2333-2336 (1994)

Copyright: © 2026 The author(s). This is an open access article distributed under the terms of the Creative Commons Attribution 4.0 International License (https://creativecommons.org/licenses/by/4.0/), which permits unrestricted use, distribution, and reproduction in any medium, provided the original author and source are credited, a link to the Creative Commons license is provided, and any changes are indicated. The Creative Commons Public Domain Dedication waiver (https://creativecommons.org/publicdomain/zero/1.0/) applies to the data made available in this article, unless otherwise stated.

174. Soriano MA, Planas AM, Rodriguez-Farré E, Ferrer I
Early 72kDa heat shock protein induction in microglial cells following focal ischemia in the rat brain.
Neurosci Lett 182: 205-207 (1994)

175. Vidal A, Blanco R, Ferrer I
Ubiquitinated structures in the white matter of the gerbil following chronic cerebral hypoperfusion.
NeuroReport 5: 2606-2608 (1994)

176. Ferrer I, Tortosa A, Blanco R, Martín F, Serrano T, Planas A, Macaya A
Naturally-occurring cell death in the developing cerebral cortex of the rat. Evidence of apoptosis-associated internucleosomal DNA fragmentation.
Neurosci Lett 182: 77-79 (1994)

177. Salazar A, Podzamczer D, Reñe R, Santin M, Perez JL, Ferrer I, Fernandez-Viladrich P, Gudiol F
Cytomegalovirus ventriculoencephalitis in AIDS patients.
Scand J Infect Dis 27:165-169 (1995)

178. Soriano MA, Ferrer I, Rodríguez-Farré E, Planas AM
Expression of c-fos and inducible hsp-70 mRNA following a transient episode of focal ischemia that had non-lethal effects on the rat brain.
Brain Res 670: 317-320 (1995)

179. Planas AM, Soriano MA, Ferrer I, Rodríguez-Farré E
Kainic-acid-induced heat shock protein-70, mRNA and protein expression is inhibited by MK-801 in certain rat brain regions.
Eur J Neurosci 7: 293-304 (1995)

180. Genis D, Matilla T, Volpini V, Rosell J, Dávalos A, Ferrer I, Molins A, Estivill X
Clinical, neuropathologic, and genetic studies of a large spinocerebellar ataxia type 1 (SCA1) kindred: (CAG)n expansion and early premonitory signs and symptoms.
Neurology 45: 24-30 (1995)

181. Ferrer I, Blanco R, Carulla M, Condom E, Alcántara S, Olivé M, Planas AM
Transforming growth factor-α immunoreactivity in the developing and adult brain
Neuroscience 66: 189-199 (1995)

182. Ferrer I, Macaya A, Blanco R, Olivé M, Cinós C, Munell F, Planas AM
Evidence of internucleosomal DNA fragmentation and identification of dying cells in X-ray-induced cell death in the developing rat brain.
Int J Dev Neurosci 13: 21-28 (1995)

183. Palacios G, Mengod G, Tortosa A, Ferrer I, Palacios JM
Increased ß-amyloid precursor protein expression in astrocytes in the gerbil hippocampus following ischaemia: association with proliferation of astrocytes.
Eur J Neurosci 7: 501-510 (1995)

184. Ferrer I, Olivé M, Rivera R, Pou A, Narberhaus B, Ugarte A
Hereditary spastic paraparesis with dementia, amyotrophy and peripheral neuropathy. A neuropathological study.
Neuropathol Appl Neurobiol 21: 255-261 (1995)

185. Ferrer I, Olivé M, Blanco R, Ballabriga J, Cinós C, Planas AM
Amoeboid microglial response following X-ray-induced apoptosis in the neonatal rat brain.
Neurosci Lett 193: 109-112 (1995)

Copyright: © 2026 The author(s). This is an open access article distributed under the terms of the Creative Commons Attribution 4.0 International License (https://creativecommons.org/licenses/by/4.0/), which permits unrestricted use, distribution, and reproduction in any medium, provided the original author and source are credited, a link to the Creative Commons license is provided, and any changes are indicated. The Creative Commons Public Domain Dedication waiver (https://creativecommons.org/publicdomain/zero/1.0/) applies to the data made available in this article, unless otherwise stated.

186. Olivé M, Blanco R, Rivera R, Cinós C, Ferrer I
Cell death induced by gamma irradiation of developing skeletal muscle.
J Anat 187: 127-132 (1995)

187. Fuentes JJ, Pritchard MA, Planas AM, Bosch A, Ferrer I, Estivill X
A new human gene from the Down syndrome critical region encodes a proline-rich protein highly expressed in fetal brain and heart.
Hum Mol Genet 4: 1935-1944 (1995)

188. Ferrer I, Soriano MA, Vidal A, Planas AM
Survival of parvalbumin-immunoreactive neurons in the gerbil hippocampus following transient forebrain ischemia does not depend on HSP-70 protein induction.
Brain Res 692: 41-46 (1995)

189. Alcántara S, Ferrer I
Postnatal development of calbindin-D28k immunoreactivity in the cerebral cortex of the cat.
Anat Embryol 192: 369-384 (1995)

190. Ferrer I, Martín F, Serrano T, Reiriz J, Pérez-Navarro E, Alberch J, Macaya A, Planas AM
Both apoptosis and necrosis occur following intrastriatal administration of excitotoxins
Acta Neuropathol 90: 504-510 (1995)

191. Espino A, Cutillas B, Tortosa A, Ferrer I, Bartrons R, Ambrosio S
Chronic effects of single intrastriatal injections of 6-hydroxydopamine or 1-methyl-4-phenylpyridinium studied by microdialysis in freely moving rats.
Brain Res 695: 151-157 (1995)

192. Ferrer I, Planas AM
Induction of heat-shock protein-70 messenger RNA and protein following systemic kainate injection in the rat: evidence of protein axonal transport.
Neuroscience 69: 1111-1118 (1995)

193. Planas AM, Soriano MA, Rodríguez-Farré E, Ferrer I
Induction of cyclooxygenase-2 mRNA and protein following transient focal ischemia in the rat brain.
Neurosci Lett 200: 187-190 (1995)

194. Ferrer I, Barrón S, Rodríguez-Farré E, Planas AM
Ionizing radiation-induced apoptosis is associated with c-Jun expression and c-Jun/AP1 activation in the developing cerebellum of the rat.
Neurosci Lett 202: 105-108 (1995)

195. Planas AM, Ferrer I, Rodríguez-Farré E
NMDA receptors mediate heat shock protein induction in the mouse brain following administration of the ibotenic acid analogue AMAA.
Brain Res 700: 289-294 (1995)

196. Ferrer I. Motor neuron disease: a type of programmed cell death.
Neurologia 11 Suppl 5:7-13 (1996) Spanish

197. Macaya A, Munell F, Reventós J, Ferrer I
Molecular factors of cerebral hypoxia.ischaemia.
Rev Neurol 24:855-864 (1996) Spanish

Copyright: © 2026 The author(s). This is an open access article distributed under the terms of the Creative Commons Attribution 4.0 International License (https://creativecommons.org/licenses/by/4.0/), which permits unrestricted use, distribution, and reproduction in any medium, provided the original author and source are credited, a link to the Creative Commons license is provided, and any changes are indicated. The Creative Commons Public Domain Dedication waiver (https://creativecommons.org/publicdomain/zero/1.0/) applies to the data made available in this article, unless otherwise stated.

198. Arboix A, Ferrer I, Martí-Vilalta JL
Clinico-anatonooathologic analysis of 25 patients with lacunar infarction.
Rev Clin Esp196:370-374 (1996) Spanish.

199. Ballabriga J, Tortosa A, Ferrer I
A 21-year-old man with myoclonic jerks, cognitive deterioration and progressive ataxia.
Rev Neurol 24:464-467 (1996).

200. Soriano MA, Ferrer I, Rodríguez-Farré E, Planas AM
Apoptosis and c-Jun in the thalamus of the rat following cortical infarction.
NeuroReport 7: 425-428 (1996)

201. Pellisé A, Roig C, Barraquer-Bordás Ll, Ferrer I
Abnormal, ubiquitinated cortical neurites in patients with diffuse Lewy body disease.
Neurosci Lett 206: 85-88 (1996)

202. Ferrer I, Blanco R, Rivera R, Carmona M, Ballabriga J, Olivé M, Planas AM
CREB-1 and CREB-2 immunoreactivity in the rat brain.
Brain Res 712: 159-164 (1996)

203. Ferrer I, Olivé M, Blanco R, Cinós C, Planas AM
Selective c-Jun overexpression is associated with ionizing radiation-induced apoptosis in the developing cerebellum of the rat.
Mol Brain Res 38: 91-100 (1996)

204. Barrientos A, Volpini V, Casademont J, Genís D, Manzanares JM, Ferrer I, Corral J, Cardellach F, Urbano- Márquez A, Estivill X, Nunes V
A nuclear defect in the 4p16 region predisposes to multiple mitochondrial DNA deletions in families with Wolfram syndrome.
J Clin Invest 97: 1570-1576 (1996)

205. Ferrer I, Olivé M, Ribera J, Planas AM
Naturally-occurring (programmed) and radiation-induced apoptosis are associated with selective c-Jun expression in the developing rat brain.
Eur J Neurosci 8: 1286-1298 (1996)

206. Alcántara S, Ferrer I, Soriano E
Transient co-localization of parvalbumin and calbindin d28k in the postnatal cerebral cortex: evidence for a phenotypic shift in developing nonpyramidal neurons.
Eur J Neurosci 8: 1329-1339 (1996)

207. Alcántara S, Soriano E, Ferrer I
Thalamic and basal forebrain afferents modulate the development of parvalbumin and calbindin D28k immunoreactivity in the barrel cortex of the rat.
Eur J Neurosci 8: 1522-153 (1996)

208. Martín G, Seguí J, Díaz-Villoslada P, Montalbán X, Planas AM, Ferrer I
Jun expression is found in neurons located in the vicinity of subacute plaques in patients with multiple sclerosis.
Neurosci Lett 212: 95-98 (1996)

209. Ferrer I, Alcántara S, Ballabriga J, Olivé M, Blanco R, Rivera R, Carmona M, Berruezo M, Pitarch S, Planas AM
Transforming growth factor-alpha (TGF-alpha) and epidermal growth factor-receptor (EGF-R) immunoreactivity in normal and pathologic brain.
Progr Neurobiol 49: 99-123 (1996)

Copyright: © 2026 The author(s). This is an open access article distributed under the terms of the Creative Commons Attribution 4.0 International License (https://creativecommons.org/licenses/by/4.0/), which permits unrestricted use, distribution, and reproduction in any medium, provided the original author and source are credited, a link to the Creative Commons license is provided, and any changes are indicated. The Creative Commons Public Domain Dedication waiver (https://creativecommons.org/publicdomain/zero/1.0/) applies to the data made available in this article, unless otherwise stated.

210. Pumarola M, Añor S, Borrás D, Ferrer I
Malignant epithelial schwannoma affecting the trigeminal nerve of a dog.
Vet Pathol 33: 434-436 (1996)

211. Ferrer I, Seguí J, Olivé M
Strong c-Jun immunoreactivity is associated with apoptotic cell death in human tumors of the central nervous system.
Neurosci Lett 214: 49-52 (1996)

212. Berciano J, JFerrer I
Glial and neuronal cytoplasmic inclusions in familial olivopontocerebellar atrophy.
Ann Neurol 40: 819 (1996)

213. Ferrer I, Seguí J, Planas AM
Amyloid deposition is associated with c-Jun expression in Alzheimer's disease and amyloid angiopathy.
Neuropathol Appl Neurobiol 22: 521-526 (1996)

214. Ferrer I
Cell death in the normal developing brain, and following ionizing radiation, methylazoxymethanol acetate, and hypoxia-ischaemia in the rat.
Neuropathol Appl Neurobiol 22:489-494 (1996)

215. Pol O, LValle Ll, Ferrer I, Puig M
The inhibitory effect of alpha2-adrenoceptor agonists on gastrointestinal transit during croton oil-induced intestinal inflammation.
Br J Pharmacol 119: 1649-1655 (1996)

216. Planas AM, Soriano MA, Berruezo M, Justicia C, Estrada A, Pitarch S, Ferrer I
Induction of Stat3, a signal transducer and transcription factor, in reactive microglia following transient focal cerebral ischemia.
Eur J Neurosci 8: 2612-2618 (1996)

217. Ferrer I, Pozas E, Martí M, Blanco R, Planas AM
Methylazoxymethanol acetate-induced apoptosis in the external granule cell layer of the developing cerebellum of the rat is associated with strong c-Jun expression and formation of high molecular weight c-Jun complexes.
J Neuropathol Exp Neurol 56: 1-9 (1997)

218. M Pumarola, Añor S, Majó N, Borrás D, Ferrer I
Spinal muscular atrophy in Holstein-Friesian calves.
Acta Neuropathol. 93: 178-183 (1997)

219. Soriano MA, Sanz O, Ferrer I, Planas AM
Cortical infarct volume is dependent on the ischemic reduction of perifocal cerebral blood flow in a three-vessel intraluminal MCA occlusion/reperfusion rat model.
Brain Res 747: 273-278 (1997)

220. Reñé R, Ferrer I, Graus F
Clinical and immunohistochemical comparison of in vivo injected anti-Hu and control IgG in the nervous system of the mouse.
Eur J Neurol 3: 319-323 (1997)

221. Ferrer I, Pozas E, Planas AM
Ubiquitination of apoptotic cells in the developing cerebellum of the rat following ionizing radiation or methylazoxymethanol injection.
Acta Neuropathol 93: 402-407 (1997)

Copyright: © 2026 The author(s). This is an open access article distributed under the terms of the Creative Commons Attribution 4.0 International License (https://creativecommons.org/licenses/by/4.0/), which permits unrestricted use, distribution, and reproduction in any medium, provided the original author and source are credited, a link to the Creative Commons license is provided, and any changes are indicated. The Creative Commons Public Domain Dedication waiver (https://creativecommons.org/publicdomain/zero/1.0/) applies to the data made available in this article, unless otherwise stated.

222. Genís D, Dávalos A, Molins A, Ferrer I
Wolfram syndrome: a neuropathological study.
Acta Neuropathol 93: 426-429 (1997)

223. Graus F, Dalmau J, Vallderiola F, Ferrer I, Reñé R, Marín C, Vecht ChJ, Arbizu T, Targa C, Moll JWB
Immunohistochemical characterization of a neuronal antibody (anti-Tr) associated with paraneoplastic cerebellar degeneration and Hodgkin's disease.
J Neuroinmunol 74: 55-61 (1997)

224. Ballabriga J, Pellisé A, Ferrer I
L-Deprenyl does not reduce brain damage in global forebrain ischemia in adult gerbils (*Meriones unguiculatus*).
J Neurol Sci 148: 1-5 (1997)

225. Ballabriga J, Pozas E, Planas AM, Ferrer I
bFGF and FGFR-3 immunoreactivity in the rat brain following systemic kainic acid administration at convulsant doses: localization of bFGF and FGFR-3 in reactive astrocytes, and FGFR-3 in reactive microglia.
Brain Res 752: 315-318 (1997)

226. Planas AM, Justicia C, Ferrer I
Stat1 in developing and adult rat brain. Induction after transient focal ischemia.
NeuroReport 8. 1359-1362 (1997)

227. Planas AM, Berruezo M, Justicia C, Barrón S, Ferrer I
Stat3 is present in the developing and adult rat cerebellum and participates in the formation of transcription complexes binding DNA at the sis-inducible element.
J Neurochem 68. 1345-1351 (1997)

228. Ferrer I, Ballabriga J, Martí E, Pozas E, Planas AM, Blasi J
BDNF and TrkB co-localize in CA1 neurons resistant to transient forebrain ischemia in the adult gerbil.
J Neuropathol Exp Neurol 56: 790-797 (1997)

229. Ferrer I, Pozas E, Ballabriga J, Planas AM
Strong c-Jun/AP-1 immunoreactivity is restricted to apoptotic cells following intracerebral ibotenic acid injection in developing rats.
Neurosci Res 28: 21-31 (1997)

230. Planas AM, Soriano MA, Estrada a, Sanz O, Martín F, Ferrer I
The heat shock stress response after brain lesions: induction of 72 kda heat shock protein (cell types involved, axonal transport, transcriptional regulation) and protein synthesis inhibition.
Progr Neurobiol 51. 607-636 (1997)

231. Ferrer I, Planas AM, Pozas E
Radiation-induced apoptosis in developing rats and kainic acid-induced excitotoxicity in adult rats are associated with distinctive morphological and biochemical c-Jun/AP-1 (N) expression.
Neuroscience 80: 449-458 (1997)

232. Ferrer I, Ballabriga J, Pozas E
Transient forebrain ischemia in the adult gerbil is associated with a complex c-Jun response.
NeuroReport 8: 2483-2487 (1997)

Copyright: © 2026 The author(s). This is an open access article distributed under the terms of the Creative Commons Attribution 4.0 International License (https://creativecommons.org/licenses/by/4.0/), which permits unrestricted use, distribution, and reproduction in any medium, provided the original author and source are credited, a link to the Creative Commons license is provided, and any changes are indicated. The Creative Commons Public Domain Dedication waiver (https://creativecommons.org/publicdomain/zero/1.0/) applies to the data made available in this article, unless otherwise stated.

233. Pozas E, Ballabriga J, Planas AM, Ferrer I
Kainic acid-induced excitotoxicity is associated with a complex c-Fos and c-Jun response which does not preclude either cell death or survival.
J Neurobiol 33: 232-246 (1997)

234. Sanz O, Estrada A, Ferrer I, Planas AM
Differential cellular distribution and dynamics of Hsp70, cyclooxygenase-2, and c-Fos in the rat brain after transient focal ischemia or kainic acid.
Neuroscience 80: 221-232 (1997)

235. de Torres C, Munell F, Ferrer I, Raventós J, Macaya A
Identification of necrotic cell death by the TUNEL assay in the hypoxic-ischemic neonatal brain.
Neurosci Lett 230: 1-4 (1997)

236. Illa I, Galiardo E, Gimeno R, Serrano C, Ferrer I, Juarez C
Signal Transducer and Activator of Transcription 1 in Human Muscle. Implications in inflammatory miopathies.
Am. J. Pathol. 151: 81-88 (1997)

237. Olivé M, Martínez-Matos JA, Montero J, Ferrer I
Apoptosis is not the mechanism of cell death of muscle fibers in human muscular dystrophies and inflammatory myopathies.
Muscle and Nerve 20: 1328-1330 (1997)

238. Ferrer I, Pozas E, López E, Ballabriga J
Bcl-2, Bax and Bcl-x expression following hypoxia-ischemia in the infant rat brain.
Acta Neuropathol 94: 583-589 (1997)

239. Olivé M, Martínez-Matos JA, Pirretas P, Povedano M, Navarro C, Ferrer I
Expression of myogenic regulatory factors (MRFs) in human neuromuscular disorders.
Neuropathol. Appl. Neurobiol. 23: 475-482 (1997)

240. Majó G, Ferrer I, Marsal J, Blasi J, Aguado F
Immunocytochemical analysis of the synaptic proteins SNAP-25 and Rab3A in human pituitary adenomas. Overexpression of SNAP-25 in the mammosomatotroph lineages.
J Pathol 183: 440-446 (1997)

241. Soriano MA, Justicia C, Ferrer I, Rodríguez-Farré E, Planas AM
Striatal infarction in the rat causes a transient reduction of tyrosine hydroxylase immunoreactivity in the ipsilateral substantia nigra.
Neurobiol Dis 4: 376-385 (1997)

242. Tortosa A, Lopez E, Ferrer I
Bcl-2 and Bax proteins in Lewy bodies from patients with Parkinson's disease and Diffuse Lewy body disease.
Neurosci. Lett. 238: 78-80 (1997)

243. Martí E, Ferrer I, Ballabriga J, Blasi J
Increase in SNAP-25 immunoreactivity in the mossy fibers following transient forebrain ischemia in the gerbil.
Acta Neuropathol 95: 254-260 (1998)

244. Ferrer I, Martí E
Distribution of fibroblast growth factor receptor-1 (FGFR-1) and FGFR-3 in the hippocampus of patients with Alzheimer's disease.
Neurosci Lett 240: 139-142 (1998)

Copyright: © 2026 The author(s). This is an open access article distributed under the terms of the Creative Commons Attribution 4.0 International License (https://creativecommons.org/licenses/by/4.0/), which permits unrestricted use, distribution, and reproduction in any medium, provided the original author and source are credited, a link to the Creative Commons license is provided, and any changes are indicated. The Creative Commons Public Domain Dedication waiver (https://creativecommons.org/publicdomain/zero/1.0/) applies to the data made available in this article, unless otherwise stated.

245. Ferrer I, Martí E, Tortosa A, Blasi J
Dystrophic neurites of senile plaques are defective in proteins involved in exocytosis and neurotransmission.
J Neuropathol Exp Neurol 57: 218-225 (1998)

246. Ferrer I, Ballabriga J, Martí E, Pozas E, Alberch J, Arenas E
BDNF up-regulates TrkB protein and prevents the death of CA1 neurons following transient forebrain ischemia.
Brain Pathol. 8: 253-261 (1998)

247. Tortosa A, López E, Ferrer I
Bcl-2 and Bax protein expression in Alzheimer's disease.
Acta Neuropathol 95: 407-412 (1998)

248. Ferrer I, López E, Pozas E, Ballabriga J, Mart E
Multiple neurotrophic signals converge in surviving CA1 neurons in the gerbil hippocampus following transient forebrain ischemia.
Comp Neurol 394: 416-430 (1998)

249. Aguado F, Majó G, Gómez de Aranda I, Ferrer I
Trk neurotrophin receptor family immunoreactivity in rat and human pituitary tissues.
Neurosci Lett 243: 13-16 (1998)

250. Sáez-Torres I, Díaz-Villoslada P, Martínez-Cáceres E, Ferrer I, Montalbán X
Gangliosides do not elicit experimental autoimmune encephalomyelitis in Lewis rats and SJL mice.
J Neuroimmunol 84: 24-29 (1998)

251. Tortosa A, Blanco R, Ferrer I
Bcl-2 and Bax protein expression in neurofibrillary tangles in progressive supranuclear palsy.
NeuroReport 9: 1049-1052 (1998)

252. Majós C, Aguilera C, Ferrer I, López L, Pons LC
Intraventricular ganglioglioma: case report.
Neuroradiology 40: 377-379 (1998)

253. Ferrer I, López E, Blanco R, Rivera R, Ballabriga J, Pozas E, Martí E
Bcl-2, Bax, and Bcl-x expression in the CA1 area of the hippocampus following transient forebrain ischemia in the adult gerbil.
Exp Brain Res 121: 167-173 (1998)

254. Graus F, Humayun Gultekin S, Ferrer I, Reiriz J, Alberch J, Dalmau J
Localization of the neuronal antigen recognized by anti-Tr antibodies from patients with paraneoplastic cerebellar degeneration and Hodgkin's disease in the rat nervous system.
Acta Neuropathol 96: 1-7 (1998)

255. Ferrer I, E Martí, E López, A Tortosa
NF-kB immunoreactivity is observed in association with ßA4 diffuse plaques in patients with Alzheimer's disease.
Neuropathol Appl Neurobiol 24: 271-277 (1998)

256. Macaya A, Munell F, Ferrer I, de Torres C, Reventós J
Cell death and associated c-jun induction in perinatal hypoxia-ischemia. Effect of the neuroprotective drug dexamethasone.
Mol Brain Res 56: 29-37 (1998)

freeneuropathology.org

Copyright: © 2026 The author(s). This is an open access article distributed under the terms of the Creative Commons Attribution 4.0 International License (https://creativecommons.org/licenses/by/4.0/), which permits unrestricted use, distribution, and reproduction in any medium, provided the original author and source are credited, a link to the Creative Commons license is provided, and any changes are indicated. The Creative Commons Public Domain Dedication waiver (https://creativecommons.org/publicdomain/zero/1.0/) applies to the data made available in this article, unless otherwise stated.

257. Planas AM, Justicia C, Soriano MA, Ferrer I
Epidermal growth factor receptor in proliferating reactive glia following transient focal ischemia in the rat brain.
Glia 23: 120-129 (1998)

258. Tate AR, Griffiths JR, Martinez-Perez I, Moreno A, IBarba I, Cabañas ME, Watson D, Alonso J, Bartumeus F, Isamat F, Ferrer I, Vila F, Ferrer E, Capdevila A, Arús C
Towards a method for automated classification of 1H MRS spectra from brain tumours.
NMR Biomedicine 11: 177-191 (1998)

259. Goutan E, Martí E, Ferrer I
BDNF, and full-length and truncated TrkB expression in the hippocampus of the rat following kainic acid excitotoxic damage. Evidence of complex time-dependent and cell-specific responses.
Mol Brain Res 59: 154-164 (1998)

260. Aguado F, Ballabriga J, Pozas E, Ferrer I
TrkA immunoreactivity in reactive astrocytes in human neurodegenerative diseases and colchicine-treated rats.
Acta Neuropathol 96: 495-501 (1998)

261. Ferrer I, Goutan E, Martí E, Arenas E
Brain-derived neurotrophic factor does not prevent ionizing radiation-induced apoptosis in the developing rat brain.
Neurosci Lett 257: 85-88 (1998)

262. Pàmpols T, Pineda M, Girós ML, Ferrer I, Cusi V, Chabás A, Sanmartí FX, Vanier MT, Christomanou H.
Neuronopathic juvenile glucosylceramidosis due to sap-C deficiency: clinical course, neuropathology and brain lipid composition in this Gaucher disease variant
Acta Neuropathol 97: 91-97 (1999)

263. Ferrer I
Nuclear DNA fragmentation in Creutzfeldt-Jakob disease: does a mere positive in situ nuclear end-labeling indicate apoptosis?
Acta Neuropathol 97: 5-12 (1999)

264. Pumarola M, Fondevila D, Borrás D, Majó N, Ferrer I
Neuronal vacuolation in young Rottweiler dogs.
Acta Neuropathol 97: 192-195 (1999)

265. López E, Pozas E, Rivera R, Ferrer I
Bcl-2 and Bax expression following methylazoxymethanol acetate-induced apoptosis in the external granule cell layer of the developing rat cerebellum.
Dev Brain Res 112: 149-153 (1999)

266. Martí E, Blasi J, Gómez de Aranda I, Ribera R, Blanco R, Ferrer I
Selective early induction of synaptosomal-associated protein (molecular weight 25,000) following systemic administration of kainate at convulsant doses in the rat.
Neuroscience 90: 1421-1432 (1999)

267. Martí E, Ferrer I, Blasi J
Transient increase of synapsin-I immunoreactivity in the mossy fiber layer of the hippocampus after transient forebrain ischemia in the Mongolian gerbil
Brain Res. 824: 153-160 (1999)

freeneuropathology.org

Copyright: © 2026 The author(s). This is an open access article distributed under the terms of the Creative Commons Attribution 4.0 International License (https://creativecommons.org/licenses/by/4.0/), which permits unrestricted use, distribution, and reproduction in any medium, provided the original author and source are credited, a link to the Creative Commons license is provided, and any changes are indicated. The Creative Commons Public Domain Dedication waiver (https://creativecommons.org/publicdomain/zero/1.0/) applies to the data made available in this article, unless otherwise stated.

268. López E, Pozas E, Rivera R, Ferrer I
Bcl-2, Bax and BclX expression following kainic acid administration at convulsant doses in the rat.
Neuroscience 91: 1461-1470 (1999)

269. Ferrer I, Rivera R, Blanco R, Martí E
Expression of proteins linked to exocytosis and neurotransmission in patients with Creutzfeldt-Jakob disease.
Neurobiol Dis 6: 92-100 (1999)

270. Pozas E, Aguado F, Ferrer I
Fra-1 immunoreactivity in the rat brain during normal postnatal development and after injury in adulthood.
Neurosci Res 33: 137-145 (1999)

271. Borrás D, Ferrer I, Pumarola M
Aged-related changes in the brain of the dog.
Vet Pathol 36: 202-211 (1999)

272. Olivé M, Ferrer I
Bcl-2 and Bax protein expression in human myopathies.
J Neurol Sci 164: 76-81 (1999)

273. Ferrer I
Neurons and their dendrites in frontotemporal dementia.
Dement Geriat Cogn Dis 10 (suppl 1): 55-60 (1999)

274. Pozas E, Aguado F, Ferrer I
Localization and expression of Jun-like immunoreactivity in apoptotic neurons induced by colchicine administration in vivo and in vitro depends on the antisera used.
Acta Neuropathol 98: 119-128 (1999)

275. Goutan E, Martí E, Ferrer I
Expression of synaptic proteins in the developing rat cerebellum following ionizing radiation.
Int J Devl Neuroscience 17: 275-283 (1999)

276. Cutillas B, Espejo M, Gil J, Ferrer I, Ambrosio S
Caspase inhibition protects nigral neurons against 6-OHDA-induced retrograde degeneration.
NeuroReport 10: 2605-2608 (1999)

277. Ferrer I, Marín C, Rey MJ, Ribalta T, Goutan E, Blanco R, Tolosa E, Martí E
BDNF and Full-length and Truncated TrkB expression in Alzheimer disease. Implications in therapeutic strategies.
J Neuropathol Exp Neurol 58: 729-739 (1999)

278. Ferrer I
Role of caspases in ionizing radiation-induced apoptosis in the developing cerebellum.
J Neurobiol 41: 549-558 (1999)

279. Ferrer I, Pozas E, Planas AM
c-Jun/AP-1 (N) expression and apoptosis.
Neuroscience 96: 447-48 (2000)

280. Ferrer I, Marín C, Rey MJ, Ribalta T
Brain-derived neurotrophic factor in patients with frontotemporal dementia.
Neurosci Lett 279: 33-36 (2000)

Copyright: © 2026 The author(s). This is an open access article distributed under the terms of the Creative Commons Attribution 4.0 International License (https://creativecommons.org/licenses/by/4.0/), which permits unrestricted use, distribution, and reproduction in any medium, provided the original author and source are credited, a link to the Creative Commons license is provided, and any changes are indicated. The Creative Commons Public Domain Dedication waiver (https://creativecommons.org/publicdomain/zero/1.0/) applies to the data made available in this article, unless otherwise stated.

281. Ferrer I, López E, Blanco R, Rivera R, Krupinski J, Martí E
Differential c-Fos and caspase expression following kainic acid excitotoxicity.
Acta Neuropathol 99: 245-256 (2000)

282. Borrás D, Pumarola M, Ferrer I
Neuronal nuclear DNA fragmentation in the aged canine brain: apoptosis or nuclear DNA fragility?
Acta Neuropathol 99: 402-408 (2000)

283. Anglada M, Vidaller A, Bolao F, Ferrer I, Olivé M
Diabetic muscular infarction.
Muscle & Nerve 825-826 (2000)

284. Ferrer I, Goutan E, Marín C, Rey MJ, Ribalta T
Brain-derived neurotrophic factor in Huntington disease.
Brain Res 866: 257-261 (2000)

285. Ferrer I, Puig B, Blanco R, Martí E
Prion protein deposition and abnormal synaptic protein expression in the cerebellum in Creutzfeldt-Jakob disease.
Neuroscience 97: 715-726 (2000)

286. Ferrer I, Blanco R
N-myc and c-myc expression in Alzheimer disease, Huntington disease and Parkinson disease.
Mol Brain Res 77: 270-276 (2000)

287. Cid J, Revilla M, Cervera A, Cervantes F, Muñoz E, Ferrer I, Montserrat E
Progressive multifocal leukoencephalopathy following oral fludarabine treatment of chronic lymphocytic leukemia.
Ann Hematol 79: 392-395 (2000)

288. Krupinski J, López E, Martí E, Ferrer I
Expression of caspases and their substrates in the rat model of focal cerebral ischemia.
Neurobiol Dis 7: 332-342 (2000)

289. Ferrer I, Blanco R, Cutillas B, Ambrosio S
Fas and Fas-L expression in Huntington's disease and Parkinson's disease.
Neuropathol Appl Neurobiol 26: 424-433 (2000)

290. Olivé M, Ferrer I
Bcl-2 and Bax immunohistochemistry in denervation-reinnervation and necrosis-regeneration of rat skeletal muscles.
Muscle & Nerve 23: 1862-1867 (2000)

291. López E, Ferrer I
Staurosporine- and H-7-induced cell death in SH-SY5Y neuroblastoma cells is associated with caspase-2 and caspase-3 activation, but not with activation of the FAS/FAS-L-caspase-8 signaling pathway.
Mol Brain Res 85: 61-67 (2000)

292. Ferrer I, Blanco R, Carmona M, Puig B, Ribera R, Rey MJ, Ribalta T
Prion protein expression in senile plaques in Alzheimer disease.
Acta Neuropathol 101: 49-56 (2001)

freeneuropathology.org

Copyright: © 2026 The author(s). This is an open access article distributed under the terms of the Creative Commons Attribution 4.0 International License (https://creativecommons.org/licenses/by/4.0/), which permits unrestricted use, distribution, and reproduction in any medium, provided the original author and source are credited, a link to the Creative Commons license is provided, and any changes are indicated. The Creative Commons Public Domain Dedication waiver (https://creativecommons.org/publicdomain/zero/1.0/) applies to the data made available in this article, unless otherwise stated.

293. Ferrer I, Blanco R, Carmona M, Ribera R, Goutan E, Puig B, Rey MJ, Cardozo A, Viñals F, Ribalta T
Phosphorylated MAP kinase (ERK1, ERK2) expression is associated with early tau deposition in neurones and glial cells, but not with increased nuclear DNA vulnerability and cell death, in Alzheimer disease, Pick's disease, progressive supranuclear palsy and corticobasal degeneration
Brain Pathol 11: 144-158 (2001)

294. Gómez C, Reiriz J, Piqué M, Gil J, Ferrer I, Ambrosio S
Low concentrations of 1-methyl-4-phenylpyridinium ion induce caspase-mediated apoptosis in human SH-SY5Y neuroblastoma cells.
J Neurosci Res 63: 421-428 (2001)

295. Martí E, Ferrer I, Blasi J
Differential regulation of chromogranin A, chromogranin B and secretoneurin protein expression after transient forebrain ischemia in the gerbil.
Acta Neuropathol 101:159-166 (2001)

296. Ferrer I, Krupinski J, Goutan E, MartíE, Ambrosio S, Arenas E
Brain-derived neurotrophic factor reduces cortical cell death by ischemia after middle cerebral artery occlusion in the rat.
Acta Neuropathol 101:229-238 (2001)

297. Ferrer I, Puig B, Goutan E, Gombau L, Muñoz-Cánoves P
Methylazoximethanol acetate-induced cell death in the granule cell layer of the developing mouse cerebellum is associated with caspase-3 activation, but does not depend on the tissue-type plasminogen activator.
Neurosci Lett 299: 77-80 (2001)

298. López-Bigas N, Olivé M, Rabionet R, Ben-David O, Martínez-Matos JA, Bravo O, Banchs I, Volpini V, Gasparini P, Avraham KB, Ferrer I, Arbonés ML, Estivill X
Connexin 31 (GJB3) is expressed in the peripheral and auditory nerves and causes neuropathy and hearing impairment.
Hum Mol Genet 10: 947-952 (2001)

299. Ferrer I
Facteurs neurotrophiques et maladie d'Alzheimer.
Alzheimer Actualites 157: 6-9 (2001)

300. Puig B, Tortosa A, Ferrer I
Cleaved caspase-3, caspase-7 and poly (ADP-ribose) polymerase are complementarily but differentially expressed in human medulloblastomas.
Neurosci Lett 306: 85-88 (2001)

301. Saiz A, Nos C, Yagüe J, Domíngues A, Graus F, Muñoz P, Catalan Collaborative Study group for CJD (pathologists: Ferrer I, Ortega A, Rey MJ, Ribalta T)
The impact of the introduction of the 14-3-3 protein assay in the surveillance of sporadic Creutzfeldt-Jakob disease in Catalonia.
J Neurol 248:592-594 (2001)

302. Ferrer I, Puig B, Krupinski J, Carmona M, Blanco R
Fas and Fas ligand expression in Alzheimer's disease.
Acta Neuropathol 102: 121-131 (2001)

303. Puig B, Ferrer I
Cell death signaling in the cerebellum in Creutzfeldt-Jakob disease.
Acta Neuropathol 102: 207-215 (2001)

Copyright: © 2026 The author(s). This is an open access article distributed under the terms of the Creative Commons Attribution 4.0 International License (https://creativecommons.org/licenses/by/4.0/), which permits unrestricted use, distribution, and reproduction in any medium, provided the original author and source are credited, a link to the Creative Commons license is provided, and any changes are indicated. The Creative Commons Public Domain Dedication waiver (https://creativecommons.org/publicdomain/zero/1.0/) applies to the data made available in this article, unless otherwise stated.

304. Sastre-Garriga J, Tintoré M, Montalbán X, Bagó J, Ferrer I
Response to botulinum toxin in a case of rigid spine syndrome.
J Neurol Neurosurg Psychiatr 71: 564-565 (2001)

305. Ferrer I, Blanco R, Carmona M, Puig B
Phosphorylated c-Myc expression in Alzheimer disease, Pick disease, progressive supranuclear palsy and corticobasal degeneration.
Neuropathol Appl Neurobiol 27: 343-351 (2001)

306. Ferrer I, Blanco R, Carmona M
Differential expression of active, phosphorylation-dependent MAP kinases, MAPK/ERK, SAPK/JNK and p38, and specific transcription factor substrates following quinolinic acid excitotoxicity in the rat.
Mol Brain Res 94: 48-58 (2001)

307. Sisó S, Ferrer I, Pumarola M
Juvenile neuroaxonal dystrophy in a Rottweiler: accumulation of synaptic proteins in dystrophic axons.
Acta Neuropathol 102: 501-504 (2001)

308. Ferrer I, Blanco R, Carmona M, Puig B, Barrachina M, Gómez C, Ambrosio S
Active, phosphorylation-dependent mitogen-activated protein kinase (MAPK/ERK), stress-activated protein kinase/c-Jun N-terminal kinase (SAPK/JNK), and p38 kinase expression in Parkinson's disease and Dementia with Lewy bodies.
J Neural Transm 108: 1383-1396 (2001)

309. Ferrer I, Blanco R, Carmona M, Puig B
Phosphorylated mitogen-activated protein kinase (MAPK/ERK-P), protein kinase of 38 kDa (p38-P), stress-activated protein kinase (SAPK/JNK-P), and calcium/calmodulin-dependent kinase II (CaM kinase II) are differentially expressed in tau deposits in neurons and glial cells in tauopathies.
J Neural Transm 108: 1397-1415 (2001)

310. Queralt R, Ezquerra M, Lleó A, Castellví M, Gelpí J, Ferrer I, Acarín N, Pasarín L, Blesa R, Oliva R
A novel mutation (V89L) in the presenilin 1 gene in a family with early onset Alzheimer's disease and marked behavioral disturbances.
J Neurol Neurosurg Psychiatr 72: 266-269 (2002)

311. Ferrer I, Blanco R, Carmona M, Puig B, Domínguez I, Viñals F
Active, phosphorylation-dependent MAP kinases, MAPK/ERK, SAPK/JNK and p38, and specific transcription factor substrates are differentially expressed following systemic administration of kainic acid to the adult rat.
Acta Neuropathol 103: 391-407 (2002)

312. Puig B, Ferrer I
Caspase-3-associated apoptotic cell death in excitotoxic necrosis of the entorhinal cortex following intraperitoneal injection of kainic acid in the rat.
Neurosci Lett 321: 182-186 (2002)

313. Ferrer I, Lizcano JM, Hernández M, Unzeta M
Overexpression of semicarbazide sensitive amine oxidase in the cerebral blood vessels in patients with Alzheimer's disease and cerebral autosomal dominant arteriopathy with subcortical infarcts and leukoencephalopathy.
Neurosci Lett 321: 21-24 (2002)

314. Gómez-Santos C, Ferrer I, Reiriz J, Viñals F, Barrachina M, Ambrosio S
MPP+ increases alpha-synuclein expression and ERK/MAP-kinase phosphorylation in human neuroblastoma SH-SY5Y cells.
Brain Res 935: 32-39 (2002)

Copyright: © 2026 The author(s). This is an open access article distributed under the terms of the Creative Commons Attribution 4.0 International License (https://creativecommons.org/licenses/by/4.0/), which permits unrestricted use, distribution, and reproduction in any medium, provided the original author and source are credited, a link to the Creative Commons license is provided, and any changes are indicated. The Creative Commons Public Domain Dedication waiver (https://creativecommons.org/publicdomain/zero/1.0/) applies to the data made available in this article, unless otherwise stated.

315. Martí E, Blasi J, Ferrer I
Early induction of secretoneurin expresión following kainic acid administration at convulsant doses in the rat and gerbil hippocampus.
Hippocampus 12: 174-185 (2002)

316. Soler-Botija C, Ferrer I, Gich I, Baiget M, Tizzano E
Neuronal death is enhanced and begins during foetal development in type I spinal muscular atrophy.
Brain 125: 1624-1634 (2002)

317. Siso S, Puig B, Varea R, Vidal E, Acín C, Prinz M, Montrasio F, Badiola J, Aguzzi A, Pumarola M, Ferrer I
Abnormal synaptic protein expression and cell death in murine scrapie.
Acta Neuropathol 103: 615-626 (2002)

318. Krupinski J, Ferrer I, Barrachina M, Secades JJ, Mercadal J, Lozano R
CDP-choline reduces pro-caspase and cleaved caspase-3 expression, nuclear DNA fragmentation, and specific PARP-cleaved products of caspase activation following middle cerebral artery occlusion in the rat.
Neuropharmacology 42: 846-854 (2002)

319. Ferrer I
Synaptic pathology and cell death in the cerebellum in Creutzfeldt-Jakob disease.
The Cerebellum 1: 213-222 (2002)

320. Muñoz E, Rey MJ, Mila M, Cardozo A, Ribalta T, Tolosa E, Ferrer I
Intranuclear inclusions, neuronal loss and CAG mosaicism in two patients with Machado-Joseph disease.
J Neurol Sci 200: 19-25 (2002)

321. Berciano J, Valderiola F, Ferrer I, Rumiá J, Pascual J, Marín C, Rey MJ, Tolosa E
Presynaptic parkinsonism in multiple system atrophy mimicking Parkinson's disease: a clinicopathological study.
Mov Dis 17: 812-816 (2002)

322. Ferrer I, Barrachina M, Puig B
Glycogen synthase kinase-3 is associated with neuronal and glial hyperphosphorylated tau deposits in Alzheimer's disease, Pick's disease, progressive supranuclear palsy and corticobasal degeneration.
Acta Neuropathol 104: 583-591 (2002)

323. Ferrer I, Barrachina M, Puig B
Anti-tau phospho-specific Ser262 antibody recognizes a variety of abnormal hyperphosphorylated tau deposits in taupathies including Pick bodies and argyrophilic grains.
Acta Neuropathol 104: 658-664 (2002)

324. Barrachina M, Secades J, Lozano R, Gómez-Santos C, Ambrosio S, Ferrer I
Citicoline increases glutathione redox ratio and reduces caspase-3 activation and cell death in staurosporine-treated SH-SY5Y human neuroblastoma cells.
Brain Res 957: 84-90 (2002)

325. Ferrer I
Differential expression of phosphorylated translation initiation factor 2 alpha in Alzheimer's disease and Creutzfledt-Jakob's disease.
Ferrer I
Neuropathol Appl Neurobiol 28: 441-451 (2002)

Copyright: © 2026 The author(s). This is an open access article distributed under the terms of the Creative Commons Attribution 4.0 International License (https://creativecommons.org/licenses/by/4.0/), which permits unrestricted use, distribution, and reproduction in any medium, provided the original author and source are credited, a link to the Creative Commons license is provided, and any changes are indicated. The Creative Commons Public Domain Dedication waiver (https://creativecommons.org/publicdomain/zero/1.0/) applies to the data made available in this article, unless otherwise stated.

326. Murphy M, Loosemore A, ferrer I, Wesseling P, Wilkins PR, Bell B
Neuropathological diagnostic accuracy.
Br J Neurosurg 16: 461-464 (2002)

327. Gómez-Santos C, Ambrosio S, Ventura F, Ferrer I, Reiriz J
TGF-beta1 increases tyrosine hydroxylase expression by a mechanism blocked by BMP-2 in human neuroblastoma SH-SY5Y cells.
Brain Res 958: 152-160 (2002)

328. Tortosa A, Vinolas N, Villa S, Verger E, Gil JM, Brell M, Caral L, Pujol T, Acebes JJ, Ribalta T, Ferrer I, Graus F
Prognostic implication of clinical, radiologic, and pathologic features in patients with anaplastic gliomas.
Cancer 97: 1063-1071 (2003)

329. Martí E, Altafaj X, Dierssen M, de la Luna S, Fotaki V, Alvarez M, Perez-Riba M, Ferrer I, Estivill X
Dyrk1A expression pattern supports specific roles of this kinase in the adult central nervous system.
Brain Res 964: 250-263 (2003)

330. Ferrer I, Pastor P, Rey MJ, Muñoz E, Puig B, Pastor E, Oliva R, Tolosa E
Tau phosphorylation and kinase activation in familial tauopathy linked to deln296 mutation.
Neuropathol Appl Neurobiol 29: 23-34 (2003)

331. Ferrer I, Barrachina M, Tolnay M, Rey MJ, Vidal N, Carmona M, Blanco R, Puig B
Phosphorylated protein kinases associated with neuronal and glial tau deposits in argyrophilic grain disease.
Brain Pathol 13: 62-78 (2003)

332. Sisó S, Pumarola M, Ferrer I
Cell death and synaptic protein expression in the ventral horn of Holstein-Friesian calves with spinal musculatr atrophy.
J Comp Path 128: 132-139 (2003)

333. Ferrer I, Friguls B, Dalfó E, Planas AM
Early modifications in the expression of mitogen-activated protein kinase (MAPK/ERK), stress-activated kinases SAPK/JNK and p38, and their phosphorylated substrates following focal cerebral ischemia.
Acta Neuropathol 105: 425-437 (2003)

334. Ferrer I, Planas AM
Signaling of cell death and cell survival following focal cerebral ischemia: Life and death struggle in the penumbra.
J Neuropathol Exp Neurol 62: 329-339 (2003)

335. Soler-Botija C, Ferrer I, Alvarez JL, Baiget M, Tizzano EF
Downregulation of Bcl-2 proteins in type I Spinal Muscular Atrophy motor neurons during fetal development.
J Neuropathol Exp Neurol 62:420-426 (2003)

336. Roig M, Gratacós M, Vazquez E, del Toro M, Foguet A, Ferrer I, Macaya A
Brainstem dysgenesis: report of five patients with congenital hypotonia, multiple cranial nerve involvement, and ocular motor apraxia.
Develop Med Child Neurol 45: 489-493 (2003)

337. Gómez-Santos C, Ferrer I, Santidrián AF, Barrachina M, Gil J, Ambrosio S
Dopamine induces autophagic cell death and alpha-synuclein increase in human neuroblastoma SH-SY5Y cells.
J Neurosci Res 73: 341-350 (2003)

Copyright: © 2026 The author(s). This is an open access article distributed under the terms of the Creative Commons Attribution 4.0 International License (https://creativecommons.org/licenses/by/4.0/), which permits unrestricted use, distribution, and reproduction in any medium, provided the original author and source are credited, a link to the Creative Commons license is provided, and any changes are indicated. The Creative Commons Public Domain Dedication waiver (https://creativecommons.org/publicdomain/zero/1.0/) applies to the data made available in this article, unless otherwise stated.

338. Olivé M. Goldfarb L, Dagvadorj A, Sambuughin N, Paulin D, Li Z, Goudeau B, Vicart P, Ferrer I
Expression of the intermediate filament protein synemin in myofibrillar myopathies and other muscle diseases.
Acta Neuropathol 1906: 1-7 (2003)

339. Sisó S, Lorenzo V, Ferrer I, Villagrassa M, Pumarola M
An anaplasic astrocytoma (optic chiasmatic-hypothalamic glioma) in a dog.
Vet Pathol 40: 567-569 (2003)

340. Ferrer I, Friguls B, Dalfó E, Justicia C, Planas A
Caspase-dependent and caspase-independent signalling of apoptosis in the penumbra following middle cerebral artery occlusion in the adult rat.
Neuropathol Appl Neurobiol 29: 472-481 (2003)

341. Ferrer I, Puig B
GluR2/3, NMDAE1 and GABAA receptors in Creutzfeldt-Jakob disease.
Acta Neuropathol 106: 311-318 (2003)

342. Sisó S, Ferrer I, Pumarola M
Abnormal synaptic protein expression in two Arabian horses with equine degenerative myeloencephalopathy.
Vet J 166: 238-243 (2003)

343. Ferrer I, Hernández I, Boada M, Llorente A, Rey MJ, Cardozo A, Ezquerra M, Puig B
Primary progressive aphasia as the initial manifestation of corticobasal degeneration and unusual tauopathies.
Acta Neuropathol 106: 419-435 (2003)

344. Barrachina M, Dominguez I, Ambrosio S, Secades J, Lozano R, Ferrer I
Neuroprotective effect of citicoline in 6-hydroxydopamine-lesioned rats and in 6-hydroxydopamine-treated SH-SY5Y human neuroblastoma cells.
J Neurol Sci 215: 105-110 (2003)

345. Diaz-Hernandez M, Hernadez F, Martin-Aparicio E, Gomez-Ramos P, Moran MA, Castaño JG, Ferrer I, Avila J, Lucas JJ
Neuronal induction of the immunoproteasome in Huntington's disease.
J Neurosci 23: 11653-11661 (2003)

346. Angulo E, Casado V, Mallol J, Canela EI, Viñals F, Ferrer I, Lluisa C, Franco R
A1 adenosine receptors accumulate in neurodegenerative structures in Alzheimer's disease and mediate both amylioid precursor protein processing and tau phosphorylation and translocation.
Brain Pathol 13: 4440-451 (2003)

347. Ferrer I, Hernandez I, Puig B, Rey MJ, Ezquerra M, Tolosa E, Boada M
Ubiquitin-negative mini-pick like bodies in the dentate gyrus of p30l tauopathy.
J Alzheimers Dis 5: 45-454 (2003)

348. Olive M, Unzeta M, Moreno D, Ferrer I
Overexpression of semicarbazide-sensitive amine oxidase in human myopathies.
Muscle and Nerve 29: 261-266 (2004)

349. Ferrer I, Boada Rovira M, Sanchez Guerra M, Rey MJ, Costa-Jussa F
Neuropathology and pathogenesis of encephalitis following amyloid-β immunization in Alzheimer's disease.
Brain Pathol 14: 11-20 (2004)

freeneuropathology.org

Copyright: © 2026 The author(s). This is an open access article distributed under the terms of the Creative Commons Attribution 4.0 International License (https://creativecommons.org/licenses/by/4.0/), which permits unrestricted use, distribution, and reproduction in any medium, provided the original author and source are credited, a link to the Creative Commons license is provided, and any changes are indicated. The Creative Commons Public Domain Dedication waiver (https://creativecommons.org/publicdomain/zero/1.0/) applies to the data made available in this article, unless otherwise stated.

350. Kaminska A, Strelkov SV, Goudeau B, Olive M, Dagvadoroj A, Fidzianska A, Simon-Costeras M, Shatunov A, Dalakas MC, Ferrer I, Kwiecinski H, Vicart P, Goldfarb LG
Small deletions disturb desmin architecture leading to breakdown of muscle cells and development of skeletal or cardioskeletal myopathy.
Hum Genet 114: 306-313 (2004)

351. Dagvadorj A, Olive M, Utizberea JA, Halle M, Shatunov A, Bönnemann C, Park KY, Goebel H, Ferrer I, Vicart P, Dalakas MC, Goldfarb LG
A series of West European patients with severe cardiac and skeletal myopathy associated with a de novo R406W mutation.
J Neurol 251: 143-149 (2004)

352. Puig B, Viñals F, Ferrer I
Active stress kinase p38 enhances and perpetuates abnormal tau phosphorylation and deposition in Pick's disease.
Acta Neuropathol 107: 185-189 (2004)

353. Olivé M, Goldfarb L, Moreno D, Laforet E, Dagvadorj A, Sambuughin N, Martinez-Matos JA, Martinez F, Alio J, Farrero E, Vicart P, Ferrer I
Desmin-related myopathy: clinical, electrophysiological, radiological, neuropathological and genetic studies.
J Neurol Sci 219: 125-137 (2004)

354. Alias L, Galiano P, Moreno D, Pujol R, Martínez-Matos JA, Baiget M, Ferrer I, Olivé M
A novel mutation in the caveolin-3 gene causing familial isolated hyperCKaemia.
Neuromuscular Dis 14: 321-324 (2004)

355. Dalfo E, Gómez-Isla T, Rosa JL, Nieto Bodelón M, Cuadrado Tejedor M, Barr china M, Ambrosio S, Ferrer I
Abnormal alpha-synuclein interactions with Rab proteins in alpha-synuclein A30P transgenic mice.
J Neuropathol Exp Neurol 63: 302-313 (2004)

356. Ferrer I, Matín B, Castaño JG, Lucas JJ, Moreno D, Olivé M
Protesomal expression, induction of immunoproteasome subunits, and local MHC class I presentation in myofibrillar myopathy and inclusion body myositis.
J Neuropathol Exp Neurol 63: 484-498 (2004)

357. Dalfó E, Barrachina M, Rosa JL, Ambrosio S, Ferrer I
Abnormal alpha-synuclein interactions with rab3a and rabphilin in diffuse Lewy body disease.
Neurobiol Dis 16: 92-97 (2004)

358. Vidal E, Montoliu P, Añor S, Sisó S, Ferrer I, Pumarola M
A novel spongiform degeneration of the grey matter in the brain of a kitten.
J Comp Pathol 131: 98-103 (2004)

359. Muñoz E, Campdelacreu J, Ferrer I, Rey MJ, Cardozo A, Gómez B, Tolosa E
Severe cerebral white matter involvement in a case of dentatorubropallidoluysian atrophy studied at autopsy.
Arch Neurol 61: 946-949 (2004)

360. Ferrer I, Freixas M, Blanco R, Carmona M, Puig B
Selective PrP-like protein, doppel immunoreactivity in dystrophic neurites of senile plaques in Alzheimer's disease.
Neuropathol Appl Neurobiol 30: 329-337 (2004)

361. Valente T, Dominguez MI, Bellmann A, Journot L, Ferrer I, Auladell C
Zac1 is up-regulated in neural cells of the limbic system of mouse brain following seizures that provoque strong cell activation.
Neuroscience 128: 323-336 (2004)

Copyright: © 2026 The author(s). This is an open access article distributed under the terms of the Creative Commons Attribution 4.0 International License (https://creativecommons.org/licenses/by/4.0/), which permits unrestricted use, distribution, and reproduction in any medium, provided the original author and source are credited, a link to the Creative Commons license is provided, and any changes are indicated. The Creative Commons Public Domain Dedication waiver (https://creativecommons.org/publicdomain/zero/1.0/) applies to the data made available in this article, unless otherwise stated.

362. Rojas-Marcos I, Encarnación M, Martínez-Yelamos S, Ferrer, Arbizu T, Gil-Peralta A, García-Lozano JR
Cadasil. Gene symbol: Notch3 Accession number Hm040053; codon number 108; nucleotide substitution TGC-TGG; amino acid substitution Cys-Trp.
Human Genet 115: 175 (2004)

363. Dalfó E, Albasanz JL, Martín M, Ferrer I
Abnormal metabotropic glutamate receptor expression and signaling in the cerebral cortex in Diffuse Lewy body disease is associated with irregular alpha-synuclein/phospholipase C interactions.
Brain Pathol 14: 388-398 (2004)

364. Puig B, Gómez-Isla T, Ribé E, Cuadrado M, Torrejón-Escribano B, Dalfó E, Ferrer I
Expression of stress-activated kinase c-Jun N-terminal kinase (SAPK/JNK-P) and p38 (p38-P), and tau hyperphosphorylation in neurites surrounding βA plaques in APP Tg2576 mice.
Neuropathol Appl Neurobiol 30: 491-502 (2004)

365. Armstrong J, Boada M, Rey MJ, Vidal N, Ferrer I
Familial Alzheimer disease associated with A713T mutation in APP.
Neurosci Lett 370: 241-243 (2004)

366. Buesa C, Maes T, Subirada F, Barrachina M, Ferrer I
DNA chip technology in brain banks: confronting a degrading world.
J Neuropathol Exp Neurol 63: 1003-1014 (2004)

367. Diaz-Hernandez M, Moreno-Herrero F, Gómez-ramos P, Morán MA, Ferrer I, Baró AM, Avila J, Hernández F, Lucas JJ
Biochemical, ultrastructural, and reversiblity studies on Huntingtin filaments isolated from mouse and human brain.
J Neurosci 24: 9361-9371 (2004)

368. Freixes M, Puig B, Rodriguez A, Torrejón-Escribano B, Blanco R, Ferrer I
Clusterin solubility and aggregation in Creutzfeldt-Jakob disease.
Acta Neuropathol 108: 295-301 (2004)

369. Beyer K, Lao JI, Carrato C, Mate JL, López D, Ferrer I, Ariza A
Upregulation of amyloid precursor protein isoforms containing Kunitz protease inhibitor in dementia with Lewy bodies.
Mol Brain Res 131: 131: 135 (2004)

370. Beyer K, Lao JL, Carrato C, Mate JL, López D, Ferrer I, Ariza A
Differential expression of alpha-synuclein isoforms in dementia with Lewy bodies.
Neuropathol Appl Neurobiol 30: 601-607 (2004)

371. Angulo E, Noe V, Casado V, Mallol J, Gómez-Isla T, Lluis C, Ferrer I, Ciudad CJ, Franco F
Up-regulation of the Kv3.4 potassium channel subunit in early stages of Alzheimer's disease.
J Neurochem 91: 547-557 (2004)

372. Pujol A, Ferrer I, Camps C, Metzger E, Hindelang C, Callizot N, Ruiz M, Pàmpols T, Giròs M, Mandel JL
Functional overlap between ABCD1 (ALD) and ABCD2 (ALDR) transporters: a therapeutic target for X-adrenoleukodystrophy.
Hum Mol Genet 13: 2997-3006 (2004)

373. Baltrons MA, Pifarre P, Ferrer I, Carot JM, García A
Reduced expression of NO-sensitive guanylyl cyclase in reactive astrocytes of Alzheimer disease, Creutzfeldt-Jakob disease, and multiple sclerosis brain.
Neurobiol Dis 17: 462-472 (2004)

Copyright: © 2026 The author(s). This is an open access article distributed under the terms of the Creative Commons Attribution 4.0 International License (https://creativecommons.org/licenses/by/4.0/), which permits unrestricted use, distribution, and reproduction in any medium, provided the original author and source are credited, a link to the Creative Commons license is provided, and any changes are indicated. The Creative Commons Public Domain Dedication waiver (https://creativecommons.org/publicdomain/zero/1.0/) applies to the data made available in this article, unless otherwise stated.

374. Martín-Clemente B, Alvarez-Castelao B, Mayo I, Sierra AB, Díaz V, Milán M, Fariñas I, Gómez-Isla T, Ferrer I, Castaño JG
Alpha-synuclein expression levels do not significantly affect proteasome function and expression in mice and stably transfected PC12 cell lines.
J Biol Chem 279: 52984-52990 (2004)

375. Ferrer I
Stress kinases involved in tau phosphorylation in Alzheimer's disease, tauopathies and APP transgenic mice.
Neurotoxicity Res 6: 469-475 (2004)

376. Gómez-Santos C, Barrachina M, Gimenez-Xavier P, Dalfo E, Ferrer I, Ambrosio S
Induction of C/EBPβ and GADD153 expression by dopamine in human neuroblastoma cells. Relationship with synuclein increase and cell damage.
Brain Res Bull 65: 87-95 (2005)

377. Barrachina M, Dalfó E, Puig B, Vidal N, Freixes M, Castaño E, Ferrer I
Amyloid deposition in the cerebral cortex in dementia with Lewy bodies is accompanied by a relative increase in APP mRNA isoforms containing the Kunitz protease inhibitor.
Neurochem Int 46: 253-260 (2005)

378. Ferrer I, Gomez-Isla T, Puig B, Freixes M, Ribe E, Dalfó E, Avila J
Current advances on different kinases involved in tau phosphorylation, and implications in Alzheimer's disease and tauopathies
Curr Alzheimer Res 2: 3-18 (2005)

379. Perez M, Ribe E, Rubio A, Lim F, Moran MA, Gomez-Ramos P, Ferrer I, G. Isla T, Avila J
Characterization of a double (amyloid precursor protein-tau) transgenic: tau phosphorylation and aggregation
Neuroscience 130: 339-347 (2005)

380. Pedraza CE, Podlesniy P, Vidal N, Arévalo JC, Lee R, Hempstead B, Ferrer I, Iglesias M, Espinet C
Pro-NGF isolated from the human brain affected by Alzheimer's disease induces neuronal apoptosis mediated by p75NTR.
Am J Pathol 166: 533-543 (2005)

381. Soler-Botija C, Cuscó I, López E, Clua A, Gich I, Baiget M, Ferrer I, Tizzano E
Choline acetyltransferase expression does not identify early pathogenetic events in fetal SMA spinal cord.
Neuromusc Dis 15: 253-258 (2005)

382. Rodríguez A, Freixes M, Dalfo E, Martín M, Puig B, Ferrer I
Metabotropic glutamate receptor/phospholipase C pathway: a vulnerable target to Creutzfeldt Jacob disease in the cerebral cortex.
Neuroscience 131: 825-832 (2005)

383. Ferrer I, M Carmona, R Blanco, D Moreno, B Torrejón-Escribano, M Olivé
Involvement of clusterin and the aggresome in abnormal protein deposits in myofibrillar myopathies and inclusión body myositis.
Brain Pathol 15: 101-108 (2005)

384. Berciano J, Ferrer I
Glial cell cytoplasmic inclusions in SCA2 do not express α-synuclein.
J Neurol 252: 742-744 (2005)

385. Dalfó E, Ferrer I
Alpha-synuclein binding to rab-3a in multiple system atrophy.
Neurosci Lett 380: 170-175 (2005)

freeneuropathology.org

Copyright: © 2026 The author(s). This is an open access article distributed under the terms of the Creative Commons Attribution 4.0 International License (https://creativecommons.org/licenses/by/4.0/), which permits unrestricted use, distribution, and reproduction in any medium, provided the original author and source are credited, a link to the Creative Commons license is provided, and any changes are indicated. The Creative Commons Public Domain Dedication waiver (https://creativecommons.org/publicdomain/zero/1.0/) applies to the data made available in this article, unless otherwise stated.

386. Zarranz JJ, Ferrer I, Lezcano E, Forcadas MI, Eizaguirre B, Atares B, Puig B, Gomez-Esteban JG, Fernandez-Maiztegui C, Rouco I, Perez-Concha T, Fernandez M, Rodriguez O, Rodriguez-Martinez AB, Martinez de Pancorbo M, Pastor P, Perez-Tur J
A novel mutation (K317M) in the MAPT gene causes FTDP and motor neuron disease.
Neurology 64: 1578-1585 (2005)

387. Armstrong J, Bonaventura I, Rojo A, Gonzalez G, Corral J, Nadal N, Volpini V, Ferrer I
Spinocerebellar ataxia type 2 (SCA2) with white matter involvement.
Neurosci Lett 381: 247-251 (2005)

388. Pamplona R, Dalfó E, Ayala V, Bellmunt J, Prat J, Ferrer I, Portero M
Proteins in human brain cortex are modified by oxidation, glycoxidation, and lipoxidation. Effects of Alzheimer disease and identification of lipoxidation targets.
J Biol. Chem 280: 21522-21530 (2005)

389. Puig B, Ferrer I, Ludueña RF, Avila J
BetaII-tubulin and phospho-tau aggregates in Alzheimer's disease and Pick's disease.
J Alzheimer's Dis 7: 213-220 (2005)

390. Brell M, Tortosa A, Verger E, Gil JM, Vinolas N, Villa S, Acebes JJ, Caral L, Pujol T, Ferrer I, Ribalta T, Graus F
Prognostic significance of O6-methylguanine-DNA methyltransferase determined by promoter hypermethylation and immunohistochemical expresión in anaplastic gliomas.
Clin Cancer Res 11: 5167-5174 (2005)

391. Dalfó E, Albasanz JL, Rodríguez A, Martín M, Ferrer I
Abnormal group I metabotropic glutamate receptor expresión and signaling in the frontal cortex in Pick disease.
J Neuropathol Exp Neurol 64: 638-647 (2005)

392. Scaravilli T, Tolosa E, Ferrer I
Progressive supranuclear palsy and corticobasal degeneration: lumping versus splitting.
Mov Disor 20; suppl 12: S21-S28 (2005)

393. Pumarola M, Vidal E, Trens JM, Serafín A, Marquez M, Ferrer I
Neuronal intranuclear inclusión disease in a horse.
Acta Neuropathol 110: 191-195 (2005)

394. Dalfó E, Portero-Otín M, Ayala V, Martinez A, Pamplona R, Ferrer I
Evidence of oxidative stress in the neocortex in incidental Lewy body disease.
J Neuropathol Exp Neurol 64: 816-830 (2005)

395. Olive M, Goldfarb LG, Shatunov A, Fischer D, Ferrer I
Myotilinopathy: refining the clinical and myopathological phenotype.
Brain 128: 2315-2326 (2005)

396. Ferrer I, Barrachina M, Puig B, Martinez de Lagrán M, Martí E, Avila J, Diersssen M
Constitutive Dyrk1A is abnormally expressed in Alzheimer disease, Down syndrome, Pick disease, and related transgenic models.
Neurobiol Dis 20: 392-400 (2005)

397. Puig B, Rey MJ, Ferrer I
Individual and regional variations of phospho-tau species in progressive supranuclear palsy.
Acta Neuropathol 110: 261-268 (2005)

freeneuropathology.org

Copyright: © 2026 The author(s). This is an open access article distributed under the terms of the Creative Commons Attribution 4.0 International License (https://creativecommons.org/licenses/by/4.0/), which permits unrestricted use, distribution, and reproduction in any medium, provided the original author and source are credited, a link to the Creative Commons license is provided, and any changes are indicated. The Creative Commons Public Domain Dedication waiver (https://creativecommons.org/publicdomain/zero/1.0/) applies to the data made available in this article, unless otherwise stated.

398. Albasanz JL, Dalfo E, Ferrer I, Martín M
Impaired metabotropic glutamate receptor/phospholipase C signaling pathway in the cerebral cortex in Alzheimer's disease and dementia with Lewy bodies correlates with stage of Alzheimer's-disease-related changes.
Neurobiol Dis 20: 685-693 (2005)

399. Ribe EM, Perez M, Puig B, Gich I, Lim F, Cuadrado M, Sesma T, Catena S, Sanchez B, Nieto M, Gómez-Ramos P, Moran MA, Cabodevilla F, Samaranch L, Ortiz L, Perez A, Ferrer I, Avila J, Gómez-Isla T
Accelerated amyloid deposition, neurofibrillary degeneration and neuronal loss in double mutant APP/tau transgenic mice.
Neurobiol Dis 20: 814-822 (2005)

400. Ferrer I, Kapfhammer JP, Hindelang C, Kempt S, Troffer-Charlier N, Broccoli V, Callyzot N, Mooyer P, Selhorst J, Vreken P, Wanders RJA, Mandel JL, Pujol A
Inactivation of the peroxisomal ABCD2 transporter in the mouse leads to late-onset ataxia involving mitochondria, Golgi and endoplasmic reticulum damage.
Hum Mol Genet 14: 3565-3577 (2005)

401. Quintana C, Bellefqih S, Laval JY, Guerquin-Kern JL, Wu TD, Avila J, Ferrer I, Arranz R, Patiño C
Study of the localization of iron, ferritin, and hemosiderin in Alzheimer's disease hippocampus by analytical microscopy at the subcellular level.
J Struct Biology 153: 42-54 (2006)

402. Santpere G, Nieto M, Puig B, Ferrer I
Abnormal SP1 transcription factor expression in Alzheimer disease and tauopathies.
Neurosci Lett 397: 30-34 (2006)

403. Santepere G, Puig B, Ferrer I
Low molecular weight species of tau in Alzheimer's disease are dependent on tau phosphorylation sites but not on delayed post-mortem delay in tissue processing.
Neurosci Lett 399: 106-110 (2006)

404. Nieto Bodelon M, Santpere G, Torrejón-Escribano G, Puig B, Ferrer I
Expression of transcription factors c-Fos, c-Jun, CREB-1, and ATF-2, and caspase-3 uin relation with abnormal tau deposits in Pick's disease.
Acta Neuropathol 111: 341-350 (2006)

405. Ferrer I
Apoptosis: Future targets for neuroprotective strategies.
Cerebrovasc Dis 21 (Suppl 2): 9-20 (2006)

406. Nieto M, Gil-Bea J, Dalfó E, Cuadrado M, Cabodevilla F, Sánchez B, Catena S, Sesma T, Ribe E, Ferrer I, Ramirez MJ, Gomez-Isla T
Increased sensitivity to MPTP in human alpha-synuclein A30P transgenic mice.
Neurobiol Aging 27: 848-856 (2006)

407. Rojo A, Campos Y, Sánchez JM, Bonaventura I, Aguilar M, Garcia A, Gonzalez L, Rey MJ, Arenas J, Olive M, Ferrer I
NARP-MILS syndrome caused by 8993T G mitochondrial DNA mutation: a clinical, genetic and neuropathological study.
Acta Neuropathol 111: 610-616 (2006)

408. Barrachina M, Castaño E, Dalfó E, Maes T, Buesa C, Ferrer I
Reduced Ubiquitin C-terminal hydrolase-1 expression levels in dementia with Lewy bodies.
Neurobiol Dis 22: 265-273 (2006)

freeneuropathology.org

Copyright: © 2026 The author(s). This is an open access article distributed under the terms of the Creative Commons Attribution 4.0 International License (https://creativecommons.org/licenses/by/4.0/), which permits unrestricted use, distribution, and reproduction in any medium, provided the original author and source are credited, a link to the Creative Commons license is provided, and any changes are indicated. The Creative Commons Public Domain Dedication waiver (https://creativecommons.org/publicdomain/zero/1.0/) applies to the data made available in this article, unless otherwise stated.

409. Dalfó E, Martinez A, Muntane G, Ferrer I
Abnormal alpha-synuclein solubility, aggregation and nitration in the frontal cortex in Pick's disease.
Neurosci Lett 400: 125-129 (2006)

410. Gil V, Nicolas O, Mingorance A, Ureña JM, Lueng Tang B, Hirata T, Sáez-valero J, Ferrer I, Soriano E, del Rio JA
Nogo-A expression in the human hippocampus in normal aging and in Alzheimer disease.
J Neuropathol Exp Neurol 65: 433-444 (2006)

411. Fischer D, Clemen CS, Olive M, Ferrer I, Goudeau B, Roth U, Badorf P, Wattjes MP, Lutterbey G, Kral T, van der Ven PF, Furst DO, Vicart P, Goldfarb LG, Moza M, Carpen O, Reichelt J, Shroder R
Different early pathogenesis in myotilinopathy compared to primary desminopathy.
Neuromusc Disord 16: 361-367 (2006)

412. Podlesniy P, Kichev A, Pedraza C, Saurat J, Encinas M, Perez B, Ferrer I, Espinet C
Pro-NGF from Alzheimer's disease and normal human brain displays distinctive abilities to induce processing and nuclear translocation of intracellular domain of p75NTR and apoptosis.
Am J Pathol 169: 119-131 (2006)

413. Barrachina M, Castaño E, Ferrer I
TaqMan PCR assay in the control of RNA normalization in human post-mortem brain tissue.
Neurochem Int 49: 276-284 (2006)

414. Pugliese M, Marcort J, Mahy N, Ferrer I
Diffuse beta-amyloid plaques and hyperphosphorylated tau are unrelated processes in aged dogs with behavioral deficits.
Acta Neuropathol 112: 175-183 (2006)

415. Alafuzoff I, Pikkarainen M, Al-Sarraj S, Arzberger T, Bell J, Bodi I, Bogdanovic N, Budka H, Bugiani O, Ferrer I, Gelpi E, Giaccone G, Graeber MB, Hauw JJ, Kamphorst W, King A, Kopp N, Korkolopoulou P, Kovacs GG, Meyronet D, Parchi P, Patsouris E, Preusser M, Ravid R, Roggendorf W, Seilhean D, Streichenberger N, Thal DR, Kretzschmar H
Interlaboratory Comparison of Assessments of Alzheimer Disease-Related Lesions: A Study of the BrainNet Europe Consortium.
J Neuropathol Exp Neurol 65:740-757 (2006)

416. Patton RL, Kalback WM, Esh CL, Kokjohn TA, Van Vickle GD, Luehrs DC, Kuo YM, Lopez J, Brune D, Ferrer I, Masliah E, Newel AJ, Beach TG, Castano EM, Roher AE.
Amyloid-beta Peptide Remnants in AN-1792-Immunized Alzheimer's Disease Patients: A Biochemical Analysis
Am J Pathol 169: 1048-1063 (2006)

417. Gomez-Santos C, Gimenez-Xavier P, Ferrer I, Ambrosio S
Intranigral dopamine toxicity and alpha-synuclein response in rats.
Neurochem Res 31: 861-868 (2006)

418. Muntane G, Dalfo E, Martinez A, Rey MJ, Avila J, Perez M, Poertero M, Pamplona R, Ayala V, Ferrer I
Glial fibrillary acidic protein is a major target of glycoxidative and lipoxidative damage in Pick's disease.
J Neurochem 99: 177-185 (2006)

419. Barrachina M, Maes T, Buesa C, Ferrer I
Lysosome-associated membrane protein 1 (LAMP-1) in Alzheimer's disease.
Neuropathol Appl Neurobiol 32: 505-516 (2006)

420. Beyer K, Humbert J, Ferrer A, Lao JI, Carrato C, Lopez D, Ferrer I, Ariza A
Low alpha-synuclein 126 mRNA levels in Dementia with Lewy bodies and Alzheimer's disease.
NeuroReport 17: 1327-1330 (2006)

Copyright: © 2026 The author(s). This is an open access article distributed under the terms of the Creative Commons Attribution 4.0 International License (https://creativecommons.org/licenses/by/4.0/), which permits unrestricted use, distribution, and reproduction in any medium, provided the original author and source are credited, a link to the Creative Commons license is provided, and any changes are indicated. The Creative Commons Public Domain Dedication waiver (https://creativecommons.org/publicdomain/zero/1.0/) applies to the data made available in this article, unless otherwise stated.

421. Rodriguez A, Martin M, Albasanz JL, Barrachina M, Espinosa JC, Torres JM, Ferrer I Adenosine A1 receptor protein levels and activity is increased in the cerebral cortex in Creutzfeldt-Jakob disease and in Bovine sponfgiform encephalopathy-infected bovine-PrP mice.
J Neuropathol Exp Neurol 65: 964-975 (2006)

422. Freixes M, Rodriguez A, Dalfó E, Ferrer I
Oxidation, glycoxidation, lipoxidation, nitration, and responses to oxidative stress in the cerebral cortex in Creutzfledt-Jakob disease.
Neurobiol Aging 27: 1807-1815 (2006)

423. Rodriguez A, Martin M, Albasanz JL, Barrachina M, Espinosa JC, Torres JM, Ferrer I
Group I mGluR signaling in BSE-infected bovine-PrP transgenic mice.
Neuroscience Letters 410: 115-120 (2006)

424. Nicoll JA, Barton E, Boche D, Neal JW, Ferrer I, Thompson P, Vlachouli C, Wilkinson D, Bayer A, Games D, Seubert P, Schenk D, Holmes C
Abeta species removal after abeta42 immunization.
J Neuropathol Exp Neurol 65: 1040-1048 (2006)

425. Rodriguez A, Perez-Gracia E, Espinosa JC, Pumarola M, Torres JM, Ferrer I
Increased expression of water channel aquaporin 1 and aquaporin 4 in Creutzfeldt-Jakob disease and in bovine spongiform encephalopathy-infected bovine-PrP transgenic mice.
Acta Neuropathol 112: 573-585 (2006)

426. Dierssen M, Gratacos M, Sahun I, Matin M, Gallego X, Amador-Arjona A, Martinez de Legran M, Murtra P, Mart´E, Pujama MA, Ferrer I, Dalfó E, Martínez-Cué C, Florez J, Torres-Peraza JF, Alberch J, Maldonado R, Fillat C, Estivill X
Transgenic mice overexpressing the full-length neurotrophin receptor TrkC exhibit increased catecholaminergic neuron density in specific brain areas and increased anxiety-like behavior and panic reaction.
Neurobiol Dis 24: 403-418 (2006)

427. Albasanz JL, Rodriguez A, Ferrer I, Martin M
Adenosine A2A receptors are up-regulated in Pick's disease frontal cortex.
Brain Pathol 16: 249-255 (2006)

428. Abad MA, Enguita M, DeGregorio-Rocasolano N, Ferrer I, Trullas R
Neuronal pentraxin 1 contributes to the neuronal damage evoked by amyloid-beta and is overexpressed in dystrophic neurites in Alzheimer's brain.
J Neurosci 26: 12735-12747 (2006)

429. Reñe R, Campdelacreu J, Ferrer I, Escrig A, Povedano M, Gascon-Bayarri J, Moral E
Familial Creutzfeldt-Jakob disease with E200K mutation presenting with neurosensorial hypoacusis.
J Neurol Neurosurg Psychiatr 78: 103-104 (2007)

430. Perez E, Barrachina M, Rodriguez A, Torrejón-Escribano B, Boada M, Hernandez I, Sánchez M, Ferrer I
Aquaporin expression in the cerebral cortex is increased at aearly stages of Alzheimer's disease.
Brain Res 1128: 164-174 (2007)

431. Ferrer I, Santpere G, Arzberger T, Bell J, Blanco R, Boluda S, Budka H, Carmona M, Giaccone G, Krebs B, Limido L, Parchi P, Puig B, Strammiello R, Strobel T, Kretzschmar H Brain protein preservation largely depends on the postmortem storage temperature: implications for study of proteins in human neurologic diseases and management of brain banks: a BrainNet Europe Study.
J Neuropathol Exp Neurol 66:35-46 (2007)

freeneuropathology.org

Copyright: © 2026 The author(s). This is an open access article distributed under the terms of the Creative Commons Attribution 4.0 International License (https://creativecommons.org/licenses/by/4.0/), which permits unrestricted use, distribution, and reproduction in any medium, provided the original author and source are credited, a link to the Creative Commons license is provided, and any changes are indicated. The Creative Commons Public Domain Dedication waiver (https://creativecommons.org/publicdomain/zero/1.0/) applies to the data made available in this article, unless otherwise stated.

432. Ferrer I, Perez E, Dalfo E, Barrachina M
Abnormal levels of prohibitin and ATP synthase in the substantia nigra and frontal cortex in Parkinson's disease.
Neurosci Lett 415: 205-209 (2007)

433. Terni B, Rey MJ, Boluda S, Torrejon-Escribano B, Sabate MP, Calopa M, van Leeuwen FW, Ferrer I
Mutant ubiquitin and p62 immunoreactivity in cases of combined multiple system atrophy and Alzheimer's disease.
Acta Neuropathol 113: 403-416 (2007)

434. Bruna J, Brell M, Ferrer I, Gimenez-Bonafe P, Tortosa A
Ki67 proliferative index predicts clinical outcome in patients with atypical or anaplastic meningioma.
Neuropathology 27: 114-120 (2007)

435. Beyer K, Humbert J, Ferrer A, Lao JI, Latorre P, Lopez D, Tolosa E, Ferrer I, Ariza A
A variable poly-T-sequence modulates alpha-synuclein isoform expression and is associated with aging.
J Neurosci Res 85: 1538-1546 (2007)

436. Schmidt A, Bauer M, Heinsen H, Feiden W, Falkai P, Alafuzoff I, Arzberger T, Al-Sarraj S, Bell JE, Bogdanovich N, Bruck W, Budka H, Ferrer I, Giaconne G, Kovacs GG, Meyronet D, Palkovits M, Parchi P, Patsouris E, Ravid R, Reynolds R, Riederer P, Roiggendorf W, Schwalber A, Seilhean D, Kretszschmar H
How a neuropsychiatric brain bank should be run: a consensus paper of Brainnet Europe II.
J Neural Transm 114: 527-537 (2007)

437. Santpere G, Puig B, Ferrer I
Oxidative damage of 14-3-3 zeta and gamma isoforms in Alzheimer’s disease and cerebral amyloid angiopathy.
Neuroscience 146: 1640-1651 (2007)

438. Humbert J, Beyer K, Carrato C, Mate JL, Ferrer I, Ariza A
Parkin sand synphilin-1 isoform expression changes in Lewy body diseases.
Neurobiol Dis 26: 681-687 (2007)

439. Ferrer I, Armstrong J, Capellari S, Parchi P, Arzberger T, Bell J, Budka H, Ströbel T, Giaccone G, Rossi G, Bogdanovic N, Fakai P, Schmitt A, Riederer P, Al-Sarraj S, Ravid R, Kretzschmar H
Effects of formalin fixation, paraffin embedding, and time of storage on DNA preservation in brain tissue: A BrainNet Europe study.
Brain Pathology 17: 297-303 (2007)

440. Rodriguez A, Ferrer I
Expression of transcription factors CREB and c-Fos in the brains of terminal Creutzfeldt-Jakob disease cases.
Neurosci Lett 421: 10-15 (2007)

441. Olive M, Armstrong J, Miralles F, Pou A, Fardeau M, Gonzalez L, Martinez F, Fischer D, Martinez Matos JA, Shatunov A, Goldfarb L, Ferrer I
Phenotypic patterns of desminopathy associated with three novel mutations in the desmin gene.
Neuromusc Disor 17: 443-450 (2007)

442. Ilieva EV, Ayala V, Jove M, Dalfo E, Cacabelos D, Povedano M, Bellmunt MJ, Ferrer I, Pamplona R, Portero-Otin M
Oxidative and endoplasmic reticulum stress interplay in sporadic amyotrophic lateral sclerosis
Brain 130: 3111-3123 (2007)

443. Perez-Buira S, Barrachina M, Rodriguez A, Albasanz JL, Martin M, Ferrer I
Expression levels of adenosine receptors in hippocampus and frontal cortex in argyrophilic grain disease.
Neurosci Lett 423: 194-199 (2007)

Copyright: © 2026 The author(s). This is an open access article distributed under the terms of the Creative Commons Attribution 4.0 International License (https://creativecommons.org/licenses/by/4.0/), which permits unrestricted use, distribution, and reproduction in any medium, provided the original author and source are credited, a link to the Creative Commons license is provided, and any changes are indicated. The Creative Commons Public Domain Dedication waiver (https://creativecommons.org/publicdomain/zero/1.0/) applies to the data made available in this article, unless otherwise stated.

444. Janue A, Odena MA, Oliveira E, Olive M, Ferrer I
Desmin is oxidized and nitrated in affected muscles in myotilinopathies and desminopathies.
J Neuropathol Exp Neurol 66: 711-723 (2007)

445. Janue A, Olive M, Ferrer I
Oxidative stress in desminopathies and myotilinopathies: a link between oxidative damage and abnormal protein aggregation.
Brain Pathol 17: 377-388 (2007)

446. Barrachina M, Moreno J, Juves S, Moreno D, Olive M, Ferrer I
Target genes of neuron-restrictive silencer factor are abnormally up-regulated in human myotilinopathy.
Am J Pathol 171: 1312-1323 (2007)

447. Costa C, Tortosa R, Rodriguez A, Ferrer I, Torres JM, Bassols A, Pumarola M
Aquaporin 1 and aquaporin 4 overexpression in bovine spongiform encephalopathy in a transgenic murine model and in cattle field cases.
Brain Res 117: 96-106 (2007)

448. Albasanz JL, Rodriguez A, Ferrer I, Martin M
Up-regulation of adenosine A1 receptors in frontal cortex from Pick's disease cases.
Eur J Neurosci 26: 3501-3508 (2007)

449. LLado A, Sanchez-Valle R, Reñe R, Ezquerra M, Rey MJ, Tolosa E, Ferrer I, Molinuevo JL
Late-onset frontotemporal dementia associated with a novel PGRN mutation
J Neural Transm 114: 1051-1054 (2008)

450. Martinez A, Dalfo E, Muntane G, Ferrer I
Glycoplytic enzymes are targets of oxidation in aged human frontal cortex and oxidative damage of these proteins is increased in progressive supranuclear palsy.
J Neural Transm 115: 59-66 (2008)

451. Beyer K, Domingo-Sabat M, Lao JI, Carrato C, Ferrer I, Ariza A
Identification and characterization of a new alpha-synuclein isoform and its role in Lewy body diseases.
Neurogenetics 9: 15-23 (2008)

452. Molina-Porcel L, Lladó A, Rey MJ, Molinuevo JL, Martinez-Lage M, esteve FX, Ferrer I, Tolosa E, Blesa R
Clinical and pathological heterogeneity of neuronal intermediate filament inclusion disease.
Arch Neurol 65: 272-275 (2008)

453. Dalfo E, Ferrer I
Early-alpha-synuclein lipoxidation in neocortex in Lewy body diseases.
Neurobiol Aging 29: 408-417 (2008)

454. Alafuzoff I, Parkkinen L, Al-Sarraj S, Arzberger T, Bell J, Bodi I, Bogdanovic N, Budka H, Ferrer I, Gelpi E, Gentleman S, Giaccone G, Kamphorst W, King A, Korkolopoulou P, Kovács GG, Larionov S, Meyronet D, Monoranu C, Morris J, Parchi P, Patsouris E, Roggendorf W, Seilhean D, Streichenberger N, Thal DR, Kretzschmar H
Assessment of alpha-Synuclein Pathology: A Study of the BrainNet Europe Consortium.
J Neuropathol Exp Neurol 67: 125-143 (2008)

455. Albasanz JL, Perez S, Barrachina M, Ferrer I, Martin M
Up-regulation of adenosine receptors in the frontal cortex in Alzheimer's disease.
Brain Pathol 18: 211-219 (2008)

Copyright: © 2026 The author(s). This is an open access article distributed under the terms of the Creative Commons Attribution 4.0 International License (https://creativecommons.org/licenses/by/4.0/), which permits unrestricted use, distribution, and reproduction in any medium, provided the original author and source are credited, a link to the Creative Commons license is provided, and any changes are indicated. The Creative Commons Public Domain Dedication waiver (https://creativecommons.org/publicdomain/zero/1.0/) applies to the data made available in this article, unless otherwise stated.

456. Olive M, van Leeuwen FW, Janué A, Moreno D, Torrejón-Escribano B, Ferrer I
Expression of mutant ubiquitin (UBB+1) and p62 in myotilinopathies and desminopathies.
Neuropathol Appl Neurobiol 34:76-87 (2008)

457. Crecelius A, Götz A, Arzberger T, Frölich T, Arnold GJ, Ferrer I, Kretzschmar H
Assessing quantitative post-mortem changes in the gray matter of the human frontal cortex proteome by 2-D DIGE.
Proteomics 8: 1276-1291 (2008)

458. Muntane G, Dalfo E, Martinez A, Ferrer I
Phosphorylation of tau and alpha-synuclein in synaptic-enriched fractions of the frontal cortex in Alzheimer’s disease, and in Parkinson’s disease and related alpha-synucleinopathies.
Neuroscience 152: 913-923 (2008)

459. Alafuzoff I, Arzberger T, Al-Sarraj S, Bodi I, Bogdanovic N, Braak H, Bugiani O, Del Teredici K, Ferrer I, Gelpi E, Giaccone G, Graeber M, Ince P, Kamphorst W, King A, Korkolopoulou P, Kovács GG, Larionov S, Meyronet D, Monoranu C, Parchi P, Patsouris E, Roggendorf W, Seilhean D, Tagliavini F, Stadelmann C, Streichenberger N, Thal DR, Wharton SB, Kretzschmar H
Staging of neurofibrillary tangle pathology in Alzheimer’s disease: A study of the BrainNet Europe Consortium.
Brain Pathol 18: 484-496 (2008)

460. Alafuzoff I, Pikkarainen M, Arzberger T, Thal DR, Al-Sarraj S, Bell J, Bodi I, Budka H, Capetillo-Zarate E, Ferrer I, Gelpi E, Gentleman S, Giaccone G, Kavantzas N, King A, Korkolopoulou P, Kovács GG, Meyronet D, Monoranu C, Parchi P, Patsouris E, Roggendorf W, Stadelmann C, Streichenberger N, Tagliavini F, Kretzschmar H
Inter-laboratory comparison of neuropathological assessments of beta-amyloid protein: a study of the BrainNet Europe consortium.
Acta Neuropathol 115: 533-546 (2008)

461. Bell JE, Alafuzoff I, Al-Sarraj S, Arzberger T, Bogdanovic N, Budka H, Dexter DT, Falkai P, Ferrer I, Gelpi E, Gentleman SM, Giaccone G, Huitinga I, Ironside JW, Klioueva N, Kovacs GG, Meyronet D, Palkovits M, Parchi P, Patsouris E, Reynolds R, Riederer P, Roggendorf W, Seilhean D, Schmitt A, Schmitz P, Streichenberger N, Schwalber A, Kretzschmar H
Management of a twenty-first century brain bank: experience in the BrainNet Europe consortium.
Acta Neuropathol 115: 497-507 (2008)

462. Gamez J, Corbera-Bedllalta M, Mila M, López-Lisbona R, Boluda S, Ferrer I
Chorea-ballism assoiciated with familial amyotrophic lateral sclerosis. A clinical, genetic and neuropathological study.
Mov Dis 23: 434-438 (2008)

463. Pamplona R, Ilievas K, Ayala V., Bellmunt MJ, Cacabelos D, Dalfo E, Ferrer I, Portero-Otin M
Maillard reaction versus other nonenzymatic modifications in neurodegenerative processes
Ann NY Acad Sci 1126: 315-319 (2008)

464. López de Munain A, Alzualde A, Gorostidi A, Otaegui D, Ruiz-Martinez J, Indakoetxea B, Ferrer I, Perez-Tur J, Saenz A, Bergareche A, Barandiaran M, Poza JJ, Zabalza R, Ruiz I, Urtasum M, Fernández-Manchola I, Olasagasti B, Espinal JB, Olaskoaga J, Rubial M, Moreno F, Carrera N, Masso FJF
Mutations in progranulin gene: clinical, pathological, and ribonucleic acid expresión findings.
Biol Psychiatr 63: 946-952 (2008)

465. Santamaria I, Santpere G, MacDonald MJ, Gomez de Barreda E, Hernandez GF, Moreno FJ, Ferrer I, Avila J
Coenzyme Q induces tau aggregation, tau filament, and Hirano bodies.
J Neuropathol Exp Neurol 67: 428-434 (2008)

Copyright: © 2026 The author(s). This is an open access article distributed under the terms of the Creative Commons Attribution 4.0 International License (https://creativecommons.org/licenses/by/4.0/), which permits unrestricted use, distribution, and reproduction in any medium, provided the original author and source are credited, a link to the Creative Commons license is provided, and any changes are indicated. The Creative Commons Public Domain Dedication waiver (https://creativecommons.org/publicdomain/zero/1.0/) applies to the data made available in this article, unless otherwise stated.

466. Lladó A, Sánchez-Valle R, Rey MJ, Ezquerra M, Tolosa E, Ferrer I, Molinuevo JL, Catalan collaborative study group for FTLD
Clinicopathological and genetic correlates of frontotemporal lobar degeneration and corticobasal degeneration
J Neurol 255: 488-494 (2008)

467. Ferrer I, Santpere G, van Leeuwen FW
Argyrophilic grain disease.
Brain 131: 1416-1432 (2008)

468. Tajes M, Gutierrez-Cuesta J, Folch J, Ferrer I, Caballero B, Smith MA, Casadesus G, Camins A, Pallas M
Lithium treatment decreases activities of tau kinases in a murine model of senescence.
J Neuropathol Exp Neurol 67: 612-623 (2008)

469. Fourcade S, López-Erauskain J, Galino J, Duval C, Naudi A, Jove M, Kemp S, Villarroya F, Ferrer I, Pamplona R, Portero-Otin M, Pujol A
Early oxidative damage underlying neurodegeneration in X-adrenoleukodystrophy.
Hum Mol Genet 17:1762-1773 (2008)

470. Marquez M, Serafin A, Fernandez-Bellon H, Serrat S, Ferrer-Admetlla A, Bertrantpetit J, Ferrer I, Pumarola M
Neuropathologic findings in an aged albino gorilla.
Vet Pathol 45: 531-537 (2008)

471. Pallas M, Pizarro JG, Gutierrez-Cuesta J, Crespo-Biel N, Alvira D, Tajes M, Yeste-Velkasco M, Flch J, Canudas AM, Sureda FX, Ferrer I, Camins A
Modulation of SIRT1 expression in different neurodegenerative models and human pathologies.
Neuroscience 154: 1388-1397 (2008)

472. Ferrer I, Martinez A, Boluda S, Parchi P, Barrachina M
Brain banks: benefits, limitations and cautions concerning the use of post-mortem brain tissue for molecular studies.
Cell Tissue Bank 9: 181-194 (2008)

473. Perez-Gracia E, Torrejon-Escribano B, Ferrer I
Dystrophic neurites of senile plaques in Alzheimer's disease are deficient in cytochrome C oxidase.
Acta Neuropathol 116: 261-268 (2008)

474. Alvira D, Ferrer I, Gutierrez-Cuesta J, Garcia-Castro B, Pallas M, Camins A.
Activation of the calpain/cdk5/p25 pathway in the gyrus cinguli in Parkinson's disease. Parkinsonism Relat Disord 14: 309-313 (2008)

475. Beyer K, Domingo-Sabat M, Humbert J, Carrato C, Ferrer I, Ariza A
Differential expression of alpha-synuclein, parkin, and synphilin-1 isoforms in Lewy body diseases.
Neurogenetics 9: 163-172 (2008)

476. Sorolla MA, Reverter-Branchat G, Tamarit J, Ferrer I, Cabiscol E
Proteomic and oxidative stress analysis in human brain samples of Huntington's disease.
Free Radic Biol Med 45: 667-678 (2008)

477. Tortosa R, Vidal E, Costa C, Alamillo E, Torres JM, Ferrer I, Pumarola M
Stress response in the central nervous system of a transgenic mouse model of bovine spongiform encephalopathy.
Vet J 178: 126-129 (2008)

Copyright: © 2026 The author(s). This is an open access article distributed under the terms of the Creative Commons Attribution 4.0 International License (https://creativecommons.org/licenses/by/4.0/), which permits unrestricted use, distribution, and reproduction in any medium, provided the original author and source are credited, a link to the Creative Commons license is provided, and any changes are indicated. The Creative Commons Public Domain Dedication waiver (https://creativecommons.org/publicdomain/zero/1.0/) applies to the data made available in this article, unless otherwise stated.

478. Ferrer I, Olive M
Molecular pathology of myofibrillar myopathies.
Expert Rev Mol Med 10:e25 (2008)

479. Villoslada P, Moreno B, Melero I, Pablos JL, Martino G, Uccelli A, Montalban X, Avila J, Rivest S, Acarin L, Appel S, Khoury SJ, McGeer P, Ferrer I, Delgado M, Obeso J, Schwartz M
Immunotherapy for neurological diseases.
Clini Immunol 128: 294-305 (2008)

480. Perez M, Moran MA, Ferrer I, Avila J, Gomez-Ramos P
Phosphorylated tau in neuritic plaques of APPsw/tauvlw transgenic mice and Alzheimer disease.
Acta Neuropathol 116: 409-418 (2008)

481. Pelegrí C, Duran-Vilaregut J, del Valle J, Crespo-Biel N, Ferrer I, Pallàs M, Camins A, Vilaplana J
Cell cycle activation in striatal neurons from Huntington's disease patients and rats treated with 3-nitropropionic acid.
Int J Dev Neurosci 26: 665-671 (2008)

482. Olivé M, Shatunov A, Gonzalez L, Carmona O, Moreno D, Quereda LG, Martinez-Matos JA, Goldfarb LG, Ferrer I
Transcription-terminating mutation in telethonin causing autosomal recessive muscular dystrophy type 2G in a European patient.
Neuromuscul Disord 18: 929-933 (2008)

483. Pamplona R, Naudí A, Gavín R, Pastrana MA, Sajnani G, Ilieva EV, Del Río JA, Portero-Otín M, Ferrer I, Requena JR
Increased oxidation, glycoxidation, and lipoxidation of brain proteins in prion disease.
Free Radic Biol Med 45: 1159-1166 (2008)

484. Guerrero D, Martinez-Velilla N, Caballero MC, Mendioroz MT, Tuñon T, Masdeu J, Rodriguez A, Armstrong J, Ferrer I
A patient with MV2 subtype of sporadic Creutzfeldt-Jakob disease and atypical clinical presentation.
Clin Neuropathol 27: 408-413 (2008)

485. Martinez A, Carmona M, Portero-Otin M, Naudi A, Pamplona R, Ferrer I
Type-dependent oxidative damage in frontotemporal lobar degeneration: cortical astrocytes are targets of oxidative damage
J Neuropathol Exp Neurol 67: 1122-1136 (2008)

486. Kovacs GG, Alafuzoff I, Al-Sarraj S, Arzberger T, Bogdanovic N, Capellari S, Ferrer I, Gelpi E, Kövari V, Kretzschmar H, Nagy Z, Parchi P, Seilhean D, Soininen H, Troakes C, Budka HMixed brain pathologies in dementia: the brainNet Europe consortium experience.
Dementia 26:343-350 (2008)

487. Clarimón J, Molina-Porcel L, Gómez-Isla T, Blesa R, Guardia-Laguarta C, González-Neira A, Estorch M, Ma Grau J, Barraquer L, Roig C, Ferrer I, Lleó A
Early-Onset Familial Lewy Body Dementia with Extensive Tauopathy: A Clinical, Genetic, and Neuropathological Study.
J Neuropathol Exp Neurol 68: 73-82 (2009)

488. Gamez J, Armstrong J, Shatunov A, Selva-O'Callaghan A, Dominguez-Oronoz R, Ortega A, Goldfarb L, Ferrer I, Olivé M
Generalized muscle pseudo-hypertrophy and stiffness associated with the myotilin Ser55Phe mutation: A novel myotilinopathy phenotype?
J Neurol Sci 277: 167-171 (2009)

489. Knafo S, Alonso-Manclares L, Gonzalez-Soriano J, Merino-Serais P, Fernaud-Espinosa I, Ferrer I, DeFelipe J
Widespread Changes in Dendritic Spines in a model of Alzheimer disease.
Cerebral Cortex 19:586-592 (2009)

freeneuropathology.org

Copyright: © 2026 The author(s). This is an open access article distributed under the terms of the Creative Commons Attribution 4.0 International License (https://creativecommons.org/licenses/by/4.0/), which permits unrestricted use, distribution, and reproduction in any medium, provided the original author and source are credited, a link to the Creative Commons license is provided, and any changes are indicated. The Creative Commons Public Domain Dedication waiver (https://creativecommons.org/publicdomain/zero/1.0/) applies to the data made available in this article, unless otherwise stated.

490. Rubio de la Torre E, Luzón-Toro B, Forte-Lago I, Minguez-Castellanos A, Ferrer I, Hilfiker S
Combined kinase inhibition modulates parkin inactivation.
Hum Mol Genet. 18: 809-823 (2009)

491. Kellner A, Matschke J, Bernreuther C, Moch H, Ferrer I, Glatzel M
Autoantibodies against beta-amyloid are common in Alzheimer's disease and help control plaque burden.
Ann Neurol 65: 24-31 (2009)

492. Gomez A, Ferrer I
Increased oxidation of certain glycolysis and energy metabolism enzymes in the frontal cortex in Lewy body diseases.
J Neurosci Res 87: 1002-1013 (2009)

493. Olivé M, Janue A, Moreno D, Gamez J, Torrejon-Escribano B, Ferrer I
TAR DNA-binding protein 43 accumulation in protein aggregate myopathies.
J Neuropathol Exp Neurol 68: 262-273 (2009)

494. Santpere G, Ferrer I
LRRK2 and neurodegeneration.
Acta Neuropathol 117: 227-246 (2009)

495. Santpere G, Ferrer I
Delineation of early changes in cases with progressive supranuclear palsy-like pathology. Astrocytes in striatum are primary targets of tau phosphorylation and GFAP oxidation.
Brain Pathol 19: 177-187 (2009)

496. Shatunov A, Olivé M, Odgerel Z, Stadelmann-Nessler C, Irlbacher K, van Landeghem F, Bayarsaikhan M, Lee HS, Goudeau B, Chinnery PF, Straub V, Hilton-Jones D, Damian MS, Kaminska A, Vicart P, Bushby K, Dalakas MC, Sambuughin N, Ferrer I, Goebel HH, Goldfarb LG
In-frame deletion in the seventh immunoglobulin-like repeat of filamin C in a family with myofibrillar myopathy.
Eur J Hum Genet 17: 656-663 (2009)

497. Monoranu CM, Apfelbacher M, Grünblatt E, Puppe B, Alafuzoff I, Ferrer I, Al-Saraj S, Keyvani K, Schmitt A, Falkai P, Schittenhelm J, Halliday G, Kril J, Harper C, McLean C, Riederer P, Roggendorf W
pH measurement as quality control on human postmortem brain tissue: A Study of the BrainNet Europe Consortium.
Neuropathol Appl Neurobiol 35: 329-337 (2009)

498. Navarro A, Boveris A, Bández MJ, Sanchez-Pinto MJ, Gómez C, Muntane G, Ferrer I
Human brain cortex: mitochondrial oxidative damage and adaptative response in Parkinson disease and dementia with Lewy bodies.
Free Radic Biol Med 46: 1574-1580 (2009)

499. Genis D, Ferrer I, Valls Sole J, Corral J, Volpini V, San Nicolas H, Gich J, Ramió- Torrenta L, Ferrándiz M, Puig J, Márquez FA kindred with cerebellar ataxia and thermoanalgesia.
J Neurol Neurosurg Psychiatr 80: 518-523 (2009)

500. Jimenez-Mallebrera C, Nascimento A, Corbera JR, Rolland MO, Froissant R, Olivé M, Ferrer I, Colomer M
Glycogen branching enzyme deficiency in an infant with severe congenital hypotonia: an emerging diagnosis of muscle weakness in the perinatal period.
Histopathology 54: 763-779 (2009)

freeneuropathology.org

Copyright: © 2026 The author(s). This is an open access article distributed under the terms of the Creative Commons Attribution 4.0 International License (https://creativecommons.org/licenses/by/4.0/), which permits unrestricted use, distribution, and reproduction in any medium, provided the original author and source are credited, a link to the Creative Commons license is provided, and any changes are indicated. The Creative Commons Public Domain Dedication waiver (https://creativecommons.org/publicdomain/zero/1.0/) applies to the data made available in this article, unless otherwise stated.

501. Alafuzoff I, Ince PG, Arzberger T, Al-Sarraj S, Bell J, Bodi I, Bogdanovic N, Bugiani O, Ferrer I, Gelpi E, Gentleman S, Giaccone G, Ironside JW, Kavantzas N, King A, Korkolopoulou P, Kovács GG, Meyronet D, Monoranu C, Parchi P, Parkkinen L, Patsouris E, Roggendorf W, Rozemuller A, Stadelmann-Nessler C, Streichenberger N, Thal DR, Kretzschmar H
Staging/typing of Lewy body related alpha-synuclein pathology: a study of the BrainNet Europe Consortium.
Acta Neuropathol 117: 635-652 (2009)

502. Ferrer I
Early involvement of the cerebral cortex in Parkinson's disease: Convergence of multiple metabolic defects.
Progr Neurobiol 88: 89-103 (2009)

503. Parchi P, Notari S, Weber P, Schimmel H, Budka H, Ferrer I, Haik S, Hauw JJ, Head MW, Ironside JW, Limido L, Rodriguez A, Ströbel T, Tagliavini F, Kretzschmar H
Inter-laboratory assessment of PrPres typing in Creutzfeldt-Jakob disease: A western blot study within the Neuroprion consortium.
Brain Pathol 19, 384-391 (2009)

504. Barrachina M, Ferrer I
DNA methylation of Alzheimer disease and tauopathy-related genes in postmortem brain.
J Neuropathol Exp Neurol 68: 880-891 (2009)

505. Biagioli M, Pinto M, Cesselli D, Zaninello M, Lazarevic D, Roncaglia P, Simone R, Vlachouli C, Polessy C, Bertin N, Beltrami A, Kobayashi P, Gallo V, Santoro C, Ferrer I, Rivella S, Beltrami CA, Carninci P, Raviola E, Gustincich S
Unexpected expression of alpha- and beta-globin in mesencephalic dopaminergic neurons and glial cells.
PNAS 106: 15446-15451 (2009)

506. Knafo S, Venero C, Merino-Serrais P, Fernaud- Espinosa I, Gonzalez-Soriano J, Ferrer I, Santpere D, DeFelipe J
Morphological alterations to neurons of the amygdala and impaired fear conditioning in a transgenic mouse model of Alzheimer's disease.
J Pathol 219: 41-51 (2009)

507. Ramirez CM, Gonzalez M, Diaz M, Alonso R, Ferrer I, Santpere G, Puig B, Meyer G, Marin R
VDAC and ERα interactions in cavolae from human cortex are altered in Alzheimer's disease.
Mol Cell Neurosci 42: 172-183 (2009)

508. Perez-Gracia E, Blanco R, Carmona M, Carro E, Ferrer I
Oxidative stress damage and oxidative stress responses in the choroid plexus in Alzheimer's disease.
Acta Neuropathol 118: 497-504 (2009)

509. Antequera D, Vargas T, Ugalde C, Spuch C, Molina JA, Ferrer I, Bermejo-Pareja F, Carro E
Cytoplasmic gelsolin increases mitochondrial activity and reduces Aβ burden in a mouse model of Alzheimer's disease.
Neurobiol Dis 36: 42-50 (2009)

510. Martí-Massó JF, Ruiz-Matínez J, Bolaño MJ, Ruiz I, Gorostidi A, Moreno F, Ferrer I, López de Munain A
Neuropathology of Parkinson's disease with the R1441G mutation in LRRK2.
Mov Disord 24: 1998-2001 (2009)

511. Gründblatt E, Monoranuy CM, Apfelbacher M, Keller D, Michel TM, Alafuzoff I, Ferrer I, Al-Saraj S, Keyvanbi K, Schmitt A, Falkai P, Schiienhelm J, McLean C, Halliday GM, Harper C, Deckert J, Roggendorf W, Riederer P
Tryptophan is a marker of human postmortem brain tissue quality.
J Neurochem 110: 1400-1408 (2009)

freeneuropathology.org

Copyright: © 2026 The author(s). This is an open access article distributed under the terms of the Creative Commons Attribution 4.0 International License (https://creativecommons.org/licenses/by/4.0/), which permits unrestricted use, distribution, and reproduction in any medium, provided the original author and source are credited, a link to the Creative Commons license is provided, and any changes are indicated. The Creative Commons Public Domain Dedication waiver (https://creativecommons.org/publicdomain/zero/1.0/) applies to the data made available in this article, unless otherwise stated.

512. Boada M, Ortiz P, Anaya F, Hernandez I, Munoz J, Nunez L, Olazaran J, Roca I, Cuberas G, Tarraga L, Buendia M, Pla RP, Ferrer I, Paez A.
Amyloid-targeted therapeutics in Alzheimer's disease: use of human albumin in plasma exchange as a novel approach for Abeta mobilization.
Drug News Perspect 22: 325-339 (2009)

513. Ferrer I
Altered mitochondria, energy metabolism, voltage-dependent anion channel, and lipid rafts converge to exhaust neurons in Alzheimer's disease.
J Bioenerg Biomembr 41: 425-431 (2009)

514. Kichev A, Ilieva EV, Piñol-Ripoll G, Podlesniy P, Ferrer I, Portero-Otin M, Pamplona M, Espinet C
Cell death and learning impairment in mice caused by in vitro modified pro-NGF can be related to its increased oxidative modifications in Alzheimer disease.
Am J Pathol 175: 2574-2585 (2009)

515. Garcia-Marin V, Blazquez-Llorca L, Rodriguez JR, Boluda S, Munaten G, Ferrer I, de Felipe J
Diminished peri-somatic GABAergic terminals on cortical neurons adjacent to amyloid plaques.
Frontiers Neuroanatomy 3: 1-17 (2009)

516. Costa C, Tortosa R, Vidal E, Padilla D, Torres JM, Ferrer I, Pumarola M, Bassols A
Central nervous system extracellular matrix changes in a transgenic mouse model of bovine spongiform encephalopathy.
Vet J 182: 306-314 (2009)

517. Huesa G, Baltrons MA, Gómez-Ramos P, Moranc A, García A, Hidalgo J, Francesa S, Santpere G, Ferrer I, Galea E
Altered Distribution of RhoA in Alzheimer's Disease and AβPP Overexpressing Mice.
J Alzh Dis 19: 37-56 (2010)

518. Terni B, Boada J, Portero-Otin M, Pamplona R, Ferrer I
Mitochondrial ATP synthase in the entorhinal cortex is a target of oxidative stress at stages I/II of Alzheimer disease pathology.
Brain Pathol 20: 222-233 (2010)

519. Guardia-Laguarta C, Pera M, Clariomon J, Molinuevo KL, Sáncjhez-Valle R, Llado A, Coma M, Gómez-Isla T, Blesa R, Ferrer I, Lleó A
Clinical, neuropathologic, and biochemical profile of the amyloid precursor protein I716F mutation.
J Neuropath Exp Neurol 69: 53-59 (2010)

520. Durrenberger PF, Fernando S, Kashefi SN, Ferrer I, Hauw JJ, Seilhean D, Smith C, Walker R, Al-Sarraj S, Troakes C, Palkovits M, Kasztner M, Huitinga I, Arzberger T, Dexter DT, Kretzschmar H, Reynolds R
Effects of antemortem and postmortem variables on human brain mRNA quality: a BrainNet Europe Study.
J Neuropathol Exp Neurol 69: 70-81 (2010)

521. Martin V, Fabelo N, Dsantpere G, Puig B, Marín R, Ferrer I, Díaz M
Lipid alterations in lipid rafts from Alzheimer's disease human brain cortex.
J Alzh Dis 19: 489-502 (2010)

522. Janué A, Olivé M, Ferrer I
Tau phosphorylation in myotilinopathies and desminopathies.
The Open Pathol J 4: 1-10 (2010)

Copyright: © 2026 The author(s). This is an open access article distributed under the terms of the Creative Commons Attribution 4.0 International License (https://creativecommons.org/licenses/by/4.0/), which permits unrestricted use, distribution, and reproduction in any medium, provided the original author and source are credited, a link to the Creative Commons license is provided, and any changes are indicated. The Creative Commons Public Domain Dedication waiver (https://creativecommons.org/publicdomain/zero/1.0/) applies to the data made available in this article, unless otherwise stated.

523. Gavín R, Ferrer I, Del Río JA
Involvement of Dab1 in APP processing and beta-amyloid deposition in sporadic Creutzfeldt-Jakob patients.
Neurobiol Dis 37: 324-329 (2010)

524. Buira SP, Albasanz JL, Dentesano G, Moreno J, Martín M, Ferrer I, Barrachina M
DNA methylation regulates adenosine A(2A) receptor cell surface expression levels.
J Neurochem 112: 1273-1285 (2010)

525. Lisboa Fernández L, Carmona M, Portero-Otin M, Naudi A, Pamplona R, Schröeder N, Ferrer I
Effects of increased iron intake during the neonatal period on the brain of adult AβPP/PS1 transgenic mice.
J Alzh Dis 19: 1069-1080 (2010)

526. Muntane G, Janue A, Fernández N, Odena MA, Oliveira E, Boluda S, Portero-Otín M, Naudí A, Boada J, Pamplona R, Ferrer I
Modification of brain lipids but not phenotype in synucleinopathy transgenic mice by long-term dietary n-3 fatty acids.
Neurochem Int 56; 318-328 (2010)

527. Martinez A, Portero-Otín M, Pamplona R, Ferrer I
Protein targets of oxidative damage in human neurodegenerative diseases with abnormal protein aggregates.
Brain Pathol 20: 281-297 (2010)

528. Van Deerlin VM, Sleiman PM, Martinez-Lage M, Chen-Plotkin A, Wang LS, Graff-Radford NR, Dickson DW, Rademakers R, Boeve BF, Grossman M, Arnold SE, Mann DM, Pickering-Brown SM, Seelaar H, Heutink P, van Swieten JC, Murrell JR, Ghetti B, Spina S, Grafman J, Hodges J, Spillantini MG, Gilman S, Lieberman AP, Kaye JA, Woltjer RL, Bigio EH, Mesulam M, Al-Sarraj S, Troakes C, Rosenberg RN, White CL 3rd, Ferrer I, Lladó A, Neumann M, Kretzschmar HA, Hulette CM, Welsh-Bohmer KA, Miller BL, Alzualde A, de Munain AL, McKee AC, Gearing M, Levey AI, Lah JJ, Hardy J, Rohrer JD, Lashley T, Mackenzie IR, Feldman HH, Hamilton RL, Dekosky ST, van der Zee J, Kumar-Singh S, Van Broeckhoven C, Mayeux R, Vonsattel JP, Troncoso JC, Kril JJ, Kwok JB, Halliday GM, Bird TD, Ince PG, Shaw PJ, Cairns NJ, Morris JC, McLean CA, Decarli C, Ellis WG, Freeman SH, Frosch MP, Growdon JH, Perl DP, Sano M, Bennett DA, Schneider JA, Beach TG, Reiman EM, Woodruff BK, Cummings J, Vinters HV, Miller CA, Chui HC, Alafuzoff I, Hartikainen P, Seilhean D, Galasko D, Masliah E, Cotman CW, Tuñón MT, Martínez MC, Munoz DG, Carroll SL, Marson D, Riederer PF, Bogdanovic N, Schellenberg GD, Hakonarson H, Trojanowski JQ, Lee VM
Common variants at 7p21 are associated with frontotemporal lobar degeneration with TDP-43 inclusions.
Nat Genet 42: 234-239 (2010)

529. Illieva EV, Naudi A, Kichev A, Ferrer I, Pamplona R, Portero-Otin M
Depletioon of oxidative and endoplasmic reticulum stress regulators in Pick disease.
Fre Radical Biol Med 48: 1302-1310 (2010)

530. Samaranch L, Lorenzo-Betancor O, Arbelo JM, Ferrer I, Lorenzo E, Irigoyen J, Pastor MA, Marrero C, Isla C, Herrera-Henriquez J, Pastor P
PINK1-linked parkinsonism is associated with Lewy body pathology.
Brain 133: 1128-1142 (2010)

531. Serrano-Pozo A, William CM, Ferrer I, Uro-Coste E, Delisle MB, Maurage CA, Hock C, Nitsch RM, Masliah E, Growdon JH, Frosch MP, Hyman BT
Beneficial effect of human anti-amyloid-beta active immunization on neurite morphology and tau pathology.
Brain 133: 1312-27 (2010)

532. Gascon-Bayarri J, Campdelacreu J, Monasterio C, Prats E, Ferrer I, Reñé R
Central apnea in a sporadic case of Creutzfeldt-Jakob disease with brainstem involvement.
Acta Neurol Belg 110: 113-115 (2010)

freeneuropathology.org

Copyright: © 2026 The author(s). This is an open access article distributed under the terms of the Creative Commons Attribution 4.0 International License (https://creativecommons.org/licenses/by/4.0/), which permits unrestricted use, distribution, and reproduction in any medium, provided the original author and source are credited, a link to the Creative Commons license is provided, and any changes are indicated. The Creative Commons Public Domain Dedication waiver (https://creativecommons.org/publicdomain/zero/1.0/) applies to the data made available in this article, unless otherwise stated.

533. Ferrer I, Aubourg P, Pujol A
General Aspects and Neuropathology of X-Linked Adrenoleukodystrophy.
Brain Pathol 20: 817-830 (2010)

534. Gómez A, Ferrer I
Involvement of the cerebral cortex in Parkinson disease linked with G2019S LRRK2 mutation without cognitive impairment.
Acta Neuropathol 120:155–167 (2010)

535. Sorolla MA, Rodríguez-Colman MJ, Tamarit J, Tamarit J, Ortega Z, Lucas JJ, Ferrer I, Rosa J, Cabiscol E
Protein oxidation in Huntington disease affects energy production and vitamin B6 metabolism.. Free Radic Biol Med 49: 612-621 (2010)

536. Alzualde A, Indakoetxea B, Ferrer I, Moreno F, Barandiaran M, Gorostidi A, Estanga A, Ruiz I, Calero M, van Leeuwen FW, Atares B, Juste R, Rodriguez-Martínez AB, López de Munain A
A Novel PRNP Y218N Mutation in Gerstmann-Sträussler-Scheinker Disease With Neurofibrillary Degeneration.
J Neuropathol Exp Neurol 69: 789-800 (2010)

537. Caballol Pons N, Montalà N, Valverde J, Brell M, Ferrer I, Martínez-Yélamos S
Isolated cerebral vasculitis associated with rheumatoid arthritis.
Joint Bone Spine 77: 361-363 (2010)

538. Vargas T, Ugalde C, Spuch C, Antequera D, Morán MJ, Martín MA, Ferrer I, Bermejo-Pareja F, Carro E
Abeta accumulation in choroid plexus is associated with mitochondrial-induced apoptosis. Neurobiol Aging 31: 1569-1581 (2010)

539. Buira SP, Dentesano G, Albasanz JL, Moreno J, Martín M, Ferrer I, Barrachina M
DNA methylation and Yin Yang-1 repress adenosine A2A receptor levels in human brain.
J Neurochem 115: 283-295 (2010)

540. Zucchelli S, Codrich M, Marcuzzi F, Pinto M, Vilotti S, Biagioli M, Ferrer I, Gustincich STRAF6 promotes atypical ubiquitination of mutant DJ-1 and alpha-synuclein and is localized to Lewy bodies in sporadic Parkinson's disease brains.
Hum Mol Genet 19: 3759-3770 (2010)

541. de Lemos L, Junyent F, Verdaguer E, Folch J, Romero R, Pallàs M, Ferrer I, Auladell C, Camins A.
Differences in activation of ERK1/2 and p38 kinase in Jnk3 null mice following KA treatment.
J Neurochem 114: 1315-1322 (2010)

542. Alzualde A, Moreno F, Martínez-Lage P, Ferrer I, Gorostidi A, Otaegui D, Blázquez L, Atares B, Cardoso S, Martínez de Pancorbo M, Juste R, Rodríguez-Martínez AB, Indakoetxea B, de Munain AL
Somatic mosaicism in a case of apparently sporadic Creutzfeldt-Jakob disease carrying a de novo D178N mutation in the PRNP gene.
Am J Med Genet B Neuropsychiatr Genet 153B(7):1283-1291 (2010)

543. Martí E, Pantano L, Bañez-Coronel M, Llorens F, Miñones-Moyano E, Porta S, Sumoy L, Ferrer I, Estivill X
A myriad of miRNA variants in control and Huntington's disease brain regions detected by massively parallel sequencing.
Nucleic Acids Res2010 38: 7219-7235 (2010)

544. Camins A, Pizarro JG, Alvira D, Gutierrez-Cuesta J, de la Torre AV, Folch J, Sureda FX, Verdaguer E, Junyent F, Jordán J, Ferrer I, Pallàs M
Activation of ataxia telangiectasia muted under experimental models and human Parkinson's disease. Cell Mol Life Sci 67: 3865-3882 (2010)

Copyright: © 2026 The author(s). This is an open access article distributed under the terms of the Creative Commons Attribution 4.0 International License (https://creativecommons.org/licenses/by/4.0/), which permits unrestricted use, distribution, and reproduction in any medium, provided the original author and source are credited, a link to the Creative Commons license is provided, and any changes are indicated. The Creative Commons Public Domain Dedication waiver (https://creativecommons.org/publicdomain/zero/1.0/) applies to the data made available in this article, unless otherwise stated.

545. Lladó L, Baliellas C, Casasnovas C, Ferrer I, Fabregat J, Ramos E, Castellote J, Torras J, Xiol X, Rafecas A
Risk of transmission of systemic transthyretin amyloidosis after domino liver transplantation. Liver Transpl 16: 1386-1392 (2010)

546. Riera-Mestre A, Martínez-Yelamos S, Martínez-Yelamos A, Ferrer I, Pujol R, Vidaller A. Clinicopathologic features and outcomes of neuro-Behçet disease in Spain: A study of 20 patients.
Eur J Intern Med 21: 536-541 (2010)

547. Ferrer I
Cognitive impairment of vascular origin: Neuropathology of cognitive impairment of vascular origin.
J Neurol Sci 299: 139-149 (2010)

548. Beyer K, Domingo-Sàbat M, Santos C, Tolosa E, Ferrer I, Ariza A
The decrease of {beta}-synuclein in cortical brain areas defines a molecular subgroup of dementia with Lewy bodies.
Brain 133: 3724-3733 (2010)

549. Alvarez-Erviti L, Rodriguez-Oroz MC, Cooper JM, Caballero C, Ferrer I, Obeso JA, Schapira AH
Chaperone-mediated autophagy markers in Parkinson disease brains.
Arch Neurol 67: 1464-1472 (2010)

550. Rodríguez-Martínez AB, Garrido JM, Zarranz JJ, Arteagoitia JM, de Pancorbo MM, Atarés B, Bilbao MJ, Ferrer I, Juste RA
A novel form of human disease with a protease-sensitive prion protein and heterozygosity methionine/valine at codon 129: Case report.
BMC Neurol 10:99 (2010)

551. Fernandez-Fernandez MR, Ferrer I, Lucas JJImpaired ATF6α processing, decreased Rheb and neuronal cell cycle re-entry in Huntington's disease.
Neurobiol Dis 41: 23-32 (2011)

552. Ferrer I
Neuropathology and Neurochemistry of Nonmotor Symptoms in Parkinson's disease.
Parkinson's Disease (SAGE-Hindawi) 2011, ID 708404 (doi:10.4061/2011/708404)

553. Veloso A, Astigarra E, Barreda-Gomez G, Manuel I, Ferrer I, Giralt MT, Ochoa B, Fresnedo O, Rodriguez-Pueretas R, J.A. Fernandez.
Anatomical distribution of lipids in human brain cortex by imaging mass spectrometry.
J Am Soc Mass Spetrom 22: 329-338 (2011)

554. Ferrer I, Gómez A, Carmona M, Huesa G, Porta S, Riera-Codina M, Biagioli M, Gustincich S, Aso E
Neuronal haemoglobin is reduced in Alzheimer's disease, argyrophilic grain disease, Parkinson's disease, and dementia with Lewy bodies.
J Alzheimer's Dis 23: 537-550 (2011)

555. Ilieva EV, Kichev A, Naudí A, Ferrer I, Pamplona R, Portero-Otín M
Mitochondrial dysfunction and oxidative and endoplasmic reticulum stress in argyrophilic grain disease.
J Neuropathol Exp Neurol 70: 253-263 (2011)

556. Ferrer I, Carmona M, Blanco R, Rey Recio MJ, San Segundo RM
Gerstmann-Straüssler-Scheinker PRNP P102L-129V mutation.
Translational Neurosci 2: 23-32 (2011)

Copyright: © 2026 The author(s). This is an open access article distributed under the terms of the Creative Commons Attribution 4.0 International License (https://creativecommons.org/licenses/by/4.0/), which permits unrestricted use, distribution, and reproduction in any medium, provided the original author and source are credited, a link to the Creative Commons license is provided, and any changes are indicated. The Creative Commons Public Domain Dedication waiver (https://creativecommons.org/publicdomain/zero/1.0/) applies to the data made available in this article, unless otherwise stated.

557. Giaccone G, Arzberger T, Alafuzoff I, Al-Sarraj S, Budka H, Duyckaerts C, Falkai P, Ferrer I, Ironside JW, Kovács GG, Meyronet D, Parchi P, Patsouris E, Revesz T, Riederer P, Rozemuller A, Schmitt A, Winblad B, Kretzschmar H; on behalf of the BrainNet Europe consortium
New lexicon and criteria for the diagnosis of Alzheimer's disease.
Lancet Neurol 10: 298-299 (2011)

558. Junyent F, de Lemos L, Verdaguer E, Folch J, Ferrer I, Ortuño-Sahagun D, Beas-Zarate C, Romero R, Pallas M, Auladell C, Camins A
Gene expression profile in JNK3 null mice: a novel specific activaytion of the PI3K/AKT pathway.
J Neurochem 117: 244-252 (2011)

559. Monoranu CM, Grünblatt E, Bartl J, Meyer A, Apfelbacher M, Keller D, Michel TM, Al-Saraj S, Schmitt A, Falkai P, Roggendorf W, Deckert J, Ferrer I, Riederer P
Methyl- and acetyltransferases are stable epigenetic markers postmortem.
Cell Tissue Bank 12: 289-297 (2011)

560. Ferrer I, Martinez A, Blanco R, Dalfo E, Carmona M
Neuropathology of sporadic Parkinson disease before the appearance of parkinsonism: preclinical Parkinson disease.
J Neural Transm 118: 821-839 (2011)

561. Sepulveda-Falla D, Matschke J, Bernreuther C, Hagel C, Puig B, Villegas A, Garcia G, Zea J, Gomez-Mancilla B, Ferrer I, Lopera F, Glatzel M
Deposition of hyperphosphorylated tau in cerebellum of PS1 E280A Alzheimer'ds disease.
Brain Pathol 21: 452-463 (2011)

562. Veloso A, Fernandez R, Astirraga E, Barreda-Gomez G, Manuel I, Giralt T, Ferrer I, Ochoa B, Rodriguez Puertas R, Fernandez JA
Distribution of lipids in human brain.
Anal Bioanal Chem 401: 89-101 (2011)

563. Calero O, Bullido MJ, Clarimón J, Frank-García A, Martínez-Martín P, Lleó A, Rey MJ, Rábano A, Blesa R, Gómez-Isla T, Valdivieso F, de Pedro-Cuesta J, Ferrer I, Calero M
Genetic Cross-Interaction between APOE and PRNP in Sporadic Alzheimer's and Creutzfeldt-Jakob Diseases.
PLoS One 6: e22090 (2011)

564. López-Erauskin J, Fourcade S, Galino J, Ruiz M, Schlüter A, Naudi A, Jove M, Portero-Otin M, Pamplona R, Ferrer I, Pujol A
Antioxidants halt axonal degeneration in a mouse model of X-adrenoleukodystrophy.
Ann Neurol 70: 84-92 (2011)

565. López-Hernández T, Sirisi S, Capdevila-Nortes X, Montolio M, Fernández-Dueñas V, Scheper GC, van der Knaap MS, Casquero P, Ciruela F, Ferrer I, Nunes V, Estévez R
Molecular mechanisms of MLC1 and GLIALCAM mutations in megalencephalic leukoencephalopathy with subcortical cysts.
Hum Mol Genet 20: 3266-3277 (2011)

566. Carulla P, Bribián A, Rangel A, Gavín R, Ferrer I, Caelles C, Del Río JA, Llorens F
Neuroprotective role of PrPC against kainate-induced epileptic seizures and cell death depends on the modulation of JNK3 activation by GluR6/7-PSD-95 binding.
Mol Biol Cell 22: 3041-3054 (2011)

Copyright: © 2026 The author(s). This is an open access article distributed under the terms of the Creative Commons Attribution 4.0 International License (https://creativecommons.org/licenses/by/4.0/), which permits unrestricted use, distribution, and reproduction in any medium, provided the original author and source are credited, a link to the Creative Commons license is provided, and any changes are indicated. The Creative Commons Public Domain Dedication waiver (https://creativecommons.org/publicdomain/zero/1.0/) applies to the data made available in this article, unless otherwise stated.

567. Miñones-Moyano E, Porta S, Escaramís G, Rabionet R, Iraola S, Kagerbauer B, Espinosa-Parrilla Y, Ferrer I, Estivill X, Martí E
MicroRNA profiling of Parkinson's disease brains identifies early downregulation of miR-34b/c which modulate mitochondrial function.
Hum Mol Genet 20: 3067-3078 (2011)

568. Sisó S, González L, Blanco R, Chianini F, Reid HW, Jeffrey M, Ferrer I
Neuropathological changes correlate temporally but not spatially with selected neuromodulatory responses in natural scrapie.
Neuropathol Appl Neurobiol 37: 484-499 (2011)

569. Ayala V, Granado-Serrano AB, Cacabelos D, Naudí A, Ilieva EV, Boada J, Caraballo-Miralles V, Lladó J, Ferrer I, Pamplona R, Portero-Otin M
Cell stress induces TDP-43 pathological changes associated with ERK1/2 dysfunction: implications in ALS.
Acta Neuropathol 122: 259-270 (2011)

570. Olivé M, Odgerel Z, Martínez A, Poza JJ, Bragado FG, Zabalza RJ, Jericó I, Gonzalez-Mera L, Shatunov A, Lee HS, Armstrong J, Maraví E, Arroyo MR, Pascual-Calvet J, Navarro C, Paradas C, Huerta M, Marquez F, Rivas EG, Pou A, Ferrer I, Goldfarb LG
Clinical and myopathological evaluation of early- and late-onset subtypes of myofibrillar myopathy.
Neuromuscul Disord 21: 533-542 (2011)

571. Molin J, Marquez M, Raurell X, Matiasek K, Ferrer I, Pumarola M
Acute clinical onset chronic inflammatory demyelinating polyneuropathy in a dog.
Muscle & Nerve 44: 441-444 (2011)

572. Mazzitelli S, Xu P, Ferrer I, Davis RJ, Tournier C
The Loss of c-Jun N-Terminal Protein Kinase Activity Prevents the Amyloidogenic Cleavage of Amyloid Precursor Protein and the Formation of Amyloid Plaques In Vivo.
J Neurosci 31: 16969-16976 (2011)

573. Fabelo N, Martín V, Santpere G, Marín R, Torrent L, Ferrer I, Díaz M
Severe alterations in lipid composition of frontal cortex lipid rafts from Parkinson's disease and incidental Parkinson's disease.
Mol Med 17(9-10): 1107-1118 (2011)

574. Galino J, Ruiz M, Fourcade S, Schlüter A, López-Erauskin J, Guilera C, Jove M, Naudi A, García-Arumí E, Andreu AL, Starkov AA, Pamplona R, Ferrer I, Portero-Otin M, Pujol AOxidative damage compromises energy metabolism in the axonal degeneration mouse model of X-adrenoleukodystrophy.
Antioxid Redox Signal 15: 2095-2107 (2011)

575. Muntané G, Ferrer I, Martinez-Vicente M
α-synuclein phosphorylation and truncation are normal events in the adult human brain.
Neuroscience 2012:106-19 (2012).

576. Tomás-Zapico C, Díez-Zaera M, Ferrer I, Gómez-Ramos P, Morán MA, Miras-Portugal MT, Díaz-Hernández M, Lucas JJ
α-Synuclein accumulates in huntingtin inclusions but forms independent filaments and its deficiency attenuates early phenotype in a mouse model of Huntington's disease.
Hum Mol Genet 21: 495-510 (2012)

577. Beyer K, Muñoz Marmol AM, Sanz C, Marginet-Flinch R, Ferrer I, Ariza A
New brain-specific beta-synuclein isoforms show expression ratio changes in Lewy body diseases.
Neurogenetics 13: 61-72 (2012)

Copyright: © 2026 The author(s). This is an open access article distributed under the terms of the Creative Commons Attribution 4.0 International License (https://creativecommons.org/licenses/by/4.0/), which permits unrestricted use, distribution, and reproduction in any medium, provided the original author and source are credited, a link to the Creative Commons license is provided, and any changes are indicated. The Creative Commons Public Domain Dedication waiver (https://creativecommons.org/publicdomain/zero/1.0/) applies to the data made available in this article, unless otherwise stated.

578. Bañez-Coronel M, Porta S, Kagerbauer B, Mateu-Huertas E, Pantano L, Ferrer I, Guzman M, Estivill X, Marti E
A pathogenic mechanism in Huntington's disease involves small CAG-repeateds RNAs with neurotoxic activity.
PLos Genetics 8:2 e1002481 (2012)

579. Ravid R, Ferrer I
Brain banks as key part of biochemical and molecular studies on cerebral cortex involvement in Parkinson's disease.
FEBS J 279: 1167-1176 (2012)

580. Martinez-Saez E, Gelpi E, Rey MJ, Ferrer I, Ribalta T, Botta-Orfila T,Nos C, Yagüe J, Sanchez-Valle R
Hirano body-rich subtypes of Creutzfeldt-Jakob disease.
Neuropathol Appl Neurobiol 38: 153-161 (2012)

581. Fernandez AF, Assenov Y, Martin-Subero JI, Balint B, Siebert R, Taniguchi H, Yamamoto H, Hidalgo M, Tan AC, Galm O, Ferrer I, Sanchez-Cespedes M, Villanueva A, Carmona J, Sanchez-Mut JV, Berdasco M, Moreno V, Capella G, Monk D, Ballestar E, Ropero S, Martinez R, Sanchez-Carbayo M, Prosper F, Agirre X, Fraga MF, Graña O, Perez-Jurado L, Mora J, Puig S, Prat J, Badimon L, Puca AA, Meltzer SJ, Lengauer T, Bridgewater J, Bock C, Esteller M
A DNA methylation fingerprint of 1628 human samples.
Genome Res 22: 407-419 (2012)

582. Torrent L, Ferrer I
PP2A and Alzheimer Disease.
Curr Alzheimer Res 9: 248-256 (2012)

583. Rodriguez-Diehl R, Rey MJ, Gironell A, Martinez-Saez E, Ferrer I, Sánchez-Valle R, Jagüe J, Nos C, Gelpi EPreclinical MSA in definite Creutzfeldt-Jakob disease.
Neuropathology 32: 158-163 (2012)

584. Ferrer I
Defining Alzheimer as a common age-related neurodegenerative process not inevitably leading to dementia.
Prog Neurobiol 97: 38-51 (2012)

585. Sanchez-Valle R, Yagüe J, Turón A, Aróstegui JI, Nos C, Rey MJ, Ferrer I, Gelpi EInherited prion disease with 4-octapeptide repeat insertion linked to valine at codón 129.
Brain 135:e212 (2012)

586. Vilotti S, Codrich M, Dal Ferro M, Pinto M, Ferrer I, Collavin L, Gustincich S, Zucchelli S
Parkinson's Disease DJ-1 L166P Alters rRNA Biogenesis by Exclusion of TTRAP from the Nucleolus and Sequestration into Cytoplasmic Aggregates via TRAF6.
PLoS One. 7:e35051 (2012)

587. Perez-Poyato MS, Mila Recensens M, Ferrer Abizanda I, Domingo Jimenez R, Lopez Lafuente A, Cusí Sanchez V, Rodriguez Revenga L, Coll Rosell MJ, Gort L, Póo Argüelles P, Pineda Marfá M
Infantile neuronal ceroid lipofuscinosis: follow-up on a Spanish series.
Gene 499: 297-302 (2012)

588. Ferrer I, López-Gonzalez I, Carmona M, Dalfó E, Pujol A, Martínez A
Neurochemistry and the non-motor aspects of PD.
Neurobiol Dis 46: 508–526 (2012)

freeneuropathology.org

Copyright: © 2026 The author(s). This is an open access article distributed under the terms of the Creative Commons Attribution 4.0 International License (https://creativecommons.org/licenses/by/4.0/), which permits unrestricted use, distribution, and reproduction in any medium, provided the original author and source are credited, a link to the Creative Commons license is provided, and any changes are indicated. The Creative Commons Public Domain Dedication waiver (https://creativecommons.org/publicdomain/zero/1.0/) applies to the data made available in this article, unless otherwise stated.

589. Antequera D, Bolos M, Spuch C, Pascual C, Ferrer I, Fernandez-Bachiller MI, Rodriguez-Franco MI, Carro E
Effects of tacrine-8-hydroxyquinoline hybrid (IQM-622) on Abeta accumulation and cell death: Involvement in hippocampal neuronal loss in Alzheimer's disease.
Neurobiol Dis 46: 682-691 (2012)

590. Aso E, Palomer E, Juvés S, Maldonado R, Muñoz FJ, Ferrer I
CB1 agonist ACEA protects neurons and reduces the cognitive impairment of AβPP/PS1 mice.
J Alzheimers Dis 30: 439-359 (2012)

591. Semak V, Semakova J, Halbaut L, Aso E, Ferrer I, Calpena A, Escolano C, Perales JC
Synthesis of tripheptanoin and formulation as a solid diet for rodents.
Eur J Lipid Sci Technol 114: 889-898 (2012)

592. Del Mar O'Callaghan M, Emperador S, López-Gallardo E, Jou C, Buján N, Montero R, Garcia-Cazorla A, Gonzaga D, Ferrer I, Briones P, Ruiz-Pesini E, Pineda M, Artuch R, Montoya J
New mitochondrial DNA mutations in tRNA associated with three severe encephalopamyopathic phenotypes: neonatal, infantile, and childhood onset.
Neurogenetics 13: 245-250 (2012)

593. Lomoio S, López-González I, Aso E, Carmona M, Torrejón-Escribano B, Scherini E, Ferrer I
Cerebellar Amyloid-β Plaques: Disturbed Cortical Circuitry in AβPP/PS1 Transgenic Mice as a Model of Familial Alzheimer's Disease.
J Alzheimers Dis 31: 285-300 (2012)

594. Gelpi E, Lladó A, Clarimón J, Rey MJ, Rivera RM, Ezquerra M, Antonell A, Navarro-Otano J, Ribalta T, Piñol-Ripoll G, Pérez A, Valldeoriola F, Ferrer I
Phenotypic variability within the inclusion body spectrum of basophilic inclusion body disease and neuronal intermediate filament inclusion disease in frontotemporal lobar degenerations with FUS-positive inclusions.
J Neuropathol Exp Neurol 71: 795-805 (2012)

595. Aso E, Lomoio S, López-González I, Joda L, Carmona M, Fernández-Yagüe N, Moreno J, Juvés S, Pujol A, Pamplona R, Portero-Otin M, Martín V, Díaz M, Ferrer I
Amyloid generation and dysfunctional immunoproteasome activation with disease progression in animal model of familial Alzheimer's disease.
Brain Pathol 22: 636-653 (2012)

596. Galea E, Launay N, Portero-Otin M, Ruiz M, Pamplona R, Aubourg P, Ferrer I, Pujol A
Oxidative stress underlying axonal degeneration in adrenoleukodystrophy: a paradigm for multifactorial neurodegenerative diseases?
Biochem Biophys Acta 1822: 1475-1488 (2012)

597. Calero O, Bullido MJ, Clarimón J, Frank-García A, Martínez-Martín P, Lleó A, Rey MJ, Sastre I, Rábano A, de Pedro-Cuesta J, Ferrer I, Calero M
A Common BACE1 Polymorphism Is a Risk Factor for Sporadic Creutzfeldt-Jakob Disease.
PLoS One 7: e43926 (2012).

598. Kley RA, Serdaroglu-Oflazer P, Leber Y, Odgerel Z, van der Ven PF, Olivé M, Ferrer I, Onipe A, Mihaylov M, Bilbao JM, Lee HS, Höhfeld J, Djinovic-Carugo K, Kong K, Tegenthoff M, Peters SA, Stenzel W, Vorgerd M, Goldfarb LG, Fürst DO
Pathophysiology of protein aggregation and extended phenotyping in filaminopathy.
Brain 135: 2642-2660 (2012)

Copyright: © 2026 The author(s). This is an open access article distributed under the terms of the Creative Commons Attribution 4.0 International License (https://creativecommons.org/licenses/by/4.0/), which permits unrestricted use, distribution, and reproduction in any medium, provided the original author and source are credited, a link to the Creative Commons license is provided, and any changes are indicated. The Creative Commons Public Domain Dedication waiver (https://creativecommons.org/publicdomain/zero/1.0/) applies to the data made available in this article, unless otherwise stated.

599. Paco S, Ferrer I, Jou C, Cusí V, Corbera J, Torner F, Gualandi F, Sabatelli P, Orozco A, Gómez-Foix AM, Colomer J, Nascimento A, Jimenez-Mallebrera C
Muscle Fiber Atrophy and Regeneration Coexist in Collagen VI-Deficient Human Muscle: Role of Calpain-3 and Nuclear Factor-κB Signaling.
J Neuropathol Exp Neurol 71: 894-906 (2012)

600. Fabelo N, Martín V, Marín R, Santpere G, Aso E, Ferrer I, Díaz M
Evidence for Premature Lipid Raft Aging in APP/PS1 Double-Transgenic Mice, a Model of Familial Alzheimer Disease.
J Neuropathol Exp Neurol 71: 868-881 (2012)

601. Parchi P, de Boni L, Saverioni D, Cohen ML, Ferrer I, Gambetti P, Gelpi E, Giaccone G, Hauw JJ, Höftberger R, Ironside JW, Jansen C, Kovacs GG, Rozemuller A, Seilhean D, Tagliavini F, Giese A, Kretzschmar HA
Consensus classification of human prion disease histotypes allows reliable identification of molecular subtypes: an inter-rater study among surveillance centres in Europe and USA.
Acta Neuropatho 124: 517-529 (2012)

602. Calero O, Bullido MJ, Clarimón J, Hortigüela R, Frank-García A, Martínez-Martín P, Lleó A, Rey MJ, Sastre I, Rábano A, de Pedro-Cuesta J, Ferrer I, Calero M
Genetic variability of the gene cluster CALHM 1-3 in sporadic Creutzfeldt-Jakob disease.
Prion 6: 407-412 (2012)

603. Carrieri C, Cimatti L, Biagioli M, Beugnet A, Zucchelli S, Fedele S, Pesce E, Ferrer I, Collavin L, Santoro C, Forrest AR, Carninci P, Biffo S, Stupka E, Gustincich S
Long non-coding antisense RNA controls Uchl1 translation through an embedded SINEB2 repeat.
Nature 491: 454-457 (2012)

604. Durrenberger PF, Fernando FS, Magliozzi R, Kashefi SN, Bonnert TP, Ferrer I, Seilhean D, Nait-Oumesmar B, Schmitt A, Gebicke-Haerter PJ, Falkai P, Grünblatt E, Palkovits M, Parchi P, Capellari S, Arzberger T, Kretzschmar H, Roncaroli F, Dexter DT, Reynolds R
Selection of novel reference genes for use in the human central nervous system: a BrainNet Europe Study.
Acta Neuropathol 124: 893-903 (2012)

605. Alafuzoff I, Gelpi E, Al-Sarraj S, Arzberger T, Attems J, Bodi I, Bogdanovic N, Budka H, Bugiani O, Englund E, Ferrer I, Gentleman S, Giaccone G, Graeber MB, Hortobagyi T, Höftberger R, Ironside JW, Jellinger K, Kavantzas N, King A, Korkolopoulou P, Kovács GG, Meyronet D, Monoranu C, Parchi P, Patsouris E, Roggendorf W, Rozemuller A, Seilhean D, Streichenberger N, Thal DR, Wharton SB, Kretzschmar H
The need to unify neuropathological assessments of vascular alterations in the ageing brain: Multicentre survey by the BrainNet Europe consortium.
Exp Gerontol 47: 825-833 (2012)

606. Rodriguez-Martinez A, Loppez de Munain A, Ferrer I, Zarranz JJ, Atares B, Villagra NT, Arteagoitia JM, Garrido JM, Juste RA
Coexistence of protease sensitive and resistant prion protein in 129VV homozygous sporadic
Creutzfeldt–Jakob disease: a case report.
Journal of Medical Case Reports 6: 348 (2012)

607. López-Erauskin J, Galino J, Bianchi P, Fourcade S, Andreu AL, Ferrer I, Muñoz-Pinedo C, Pujol AOxidative stress modulates mitochondrial failure and cyclophilin D function in X-linked adrenoleukodystrophy.
Brain 135: 3584-3598 (2012)

608. Barrachina M, Moreno J, Villar-Menendez I, Juves S, Ferrer I
Histone tail acetylation in brain occurs in an unpredictable manner after death.
Cell Tissue Bank 13: 597-606 (2012)

freeneuropathology.org

Copyright: © 2026 The author(s). This is an open access article distributed under the terms of the Creative Commons Attribution 4.0 International License (https://creativecommons.org/licenses/by/4.0/), which permits unrestricted use, distribution, and reproduction in any medium, provided the original author and source are credited, a link to the Creative Commons license is provided, and any changes are indicated. The Creative Commons Public Domain Dedication waiver (https://creativecommons.org/publicdomain/zero/1.0/) applies to the data made available in this article, unless otherwise stated.

609. Kwon KH, Kim JY, Kim SY, Min HK, Lee HJ, Ji IJ, Kang T, Park GW, An HJ, Lee B, Ravid R, Ferrer I, Chung CK, Paik YK, Hancock WS, Park YM, Yoo JS.
Chromosome 11-centric human proteome analysis of human brain hippocampus tissue.
J Proteome Res12: 97-105 (2013)

610. López-Menéndez C, Gamir-Morralla A, Jurado-Arjona J, Higuero AM, Campanero MR, Ferrer I, Hernández F, Avila J, Díaz-Guerra M, Iglesias T
Kidins220 accumulates with tau in human Alzheimer's disease and related models: modulation of its calpain-processing by GSK3β/PP1 imbalance.
Hum Mol Genet 22: 466-482 (2013)

611. Pera M, Alcolea D, Sánchez-Valle R, Guardia-Laguarta C, Colom-Cadena M, Badiola N, Suárez-Calvet M, Lladó A, Barrera-Ocampo AA, Sepulveda-Falla D, Blesa R, Molinuevo JL, Clarimón J,Ferrer I, Gelpi E, Lleó A
Distinct patterns of APP processing in the CNS in autosomal-dominant and sporadic Alzheimer disease.
Acta Neuropathol 125: 201-213 (2013)

612. Kovacs GG, Rozemuller AJM, van Swieten JC, Gelpi E, Majtenyi K, Al-Sarraj S, Troakes C, Bodi I, King A, Hortebagui T, Esiri M, Ansorge O, Giaconne G, Ferrer I, Arzberger T, Bogdanovic N, Nilsson T, Leisser I, Alafuzoff I, Ironside JW, Kretzschmar H, Budka
Neuropathology of the hippocampus in FTLD-Tau with Pick bodies: a study of the BrainNet Europe Consortium.
Neuropathol Appl Neurobiol 39: 166-178 (2013)

613. López-Gonzalez I, Carmona M, Blanco R, Luna-Muñoz J, Martinez-Maldonado A, Mena R, Ferrer I
Characterization of thorn-shped astrocytes in White matter of temoral lobe in Alzheimer’s disease brains.
Brain Pathol 23: 144-153 (2013)

614. Launay N, Ruiz M, Fourcade S, Schlüter A, Guilera C, Ferrer I, Knecht E, Pujol A
Oxidative stress regulates the ubiquitin-proteasome system and immunoproteasome functioning in a mouse model of X-adrenoleukodystrophy
Brain 136: 891-904 (2013)

615. Antonell A, Lladó A, Altirriba J, Botta-Orfila T, Balasa M, Fernández M, Ferrer I, Sánchez-Valle R, Molinuevo JL
A preliminary study of the whole-genome expression profile of sporadic and monogenic early-onset Alzheimer's disease.
Neurobiol Aging 34: 1772-1778 (2013)

616. Aso E, Semakova J, Joda L, Semak V, Halbaut L, Calpena A, Escolano C, Perales JC, Ferrer I
Triheptanoin Supplementation to Ketogenic Diet Curbs Cognitive Impairment in APP/PS1 Mice Used as a Model of Familial Alzheimer's Disease.
Curr Alzheimer Res 10: 290-297 (2013)

617. Plá V, Paco S, Ghezali G, Ciria V, Pozas E, Ferrer I, Aguado F
Secretory Sorting Receptors Carboxypeptidase E and Secretogranin III in Amyloid β-Associated Neural Degeneration in Alzheimer's Disease.
Brain Pathol 23: 274-284 (2013)

618. Gelpi E, Soler Insa JM, Parchi P, Saveroni D, Yagüe J, Nos C, Martínez-Saez E, Ribalta T, Ferrerr I, Sanchez-Valle R
Atypical neuropathological sCJD-MM phenotype with abundant White matter kuru-type plaques sparing the cerebellar cortex
Neuropathology 33: 204-208 (2013)

Copyright: © 2026 The author(s). This is an open access article distributed under the terms of the Creative Commons Attribution 4.0 International License (https://creativecommons.org/licenses/by/4.0/), which permits unrestricted use, distribution, and reproduction in any medium, provided the original author and source are credited, a link to the Creative Commons license is provided, and any changes are indicated. The Creative Commons Public Domain Dedication waiver (https://creativecommons.org/publicdomain/zero/1.0/) applies to the data made available in this article, unless otherwise stated.

619. Pinacho R, Villalmanzo N, Roca M, Iniesta R, Monje A, Haro JM, Meana JJ, Ferrer I, Gill G, Ramos B
Analysis of Sp transcription factors in the postmortem brain of chronic schizophrenia: A pilot study of relationship to negative symptoms.
J Psychiatr Res 47: 926-934 (2013)

620. Villar-Menéndez I, Blanch M, Tyebji S, Pereira-Veiga T, Albasanz JL, Martín M,**Ferrer I**, Pérez-Navarro E, Barrachina M
Increased 5-methylcytosine and decreased 5-hydroxymethylcytosine levels are associated with reduced striatal A2AR levels in Huntington's disease.
Neuromolecular Med 15: 295-309 (2013)

621. Garcia-Esparcia P, Schlüter A, Carmona M, Moreno J, Ansoleaga B, Torrejón-Escribano B, Gustincich S, Pujol A, Ferrer I
Functional Genomics Reveals Dysregulation of Cortical Olfactory Receptors in Parkinson Disease: Novel Putative Chemoreceptors in the Human Brain.
J Neuropathol Exp Neurol.72: 524-539 (2013)

622. Aso E, Juvés S, Maldonado R, Ferrer I
CB2 Cannabinoid Receptor Agonist Ameliorates Alzheimer-Like Phenotype in AβPP/PS1 Mice.
J Alzheimers Dis 35: 847-58 (2013)

623. Perez-Gonzalez R, Pascual C, Antequera D, Bolos M, Redondo M, Perez DI, Perez-Grijalba V, Kryzanowska A, Sarasa M, Gil C, Ferrer I, Martinez A, Carro E
Phosphodiesterase 7 inhibitor reduced cognitive impairment and pathological hallmarks in a mouse modelo of Alzheimer's disease.
Neurobiol Aging 34: 2133-2145 (2013)

624. Ansoleaga B, Garcia-Esparcia P, Llorens F, Moreno J, Aso E, Ferrer I
Dysregulation of brain olfactory and taste receptors in AD, PSP and CJD, and AD-related model.
Neuroscience 248: 369-382 (2013)

625. Morató L, Galino J, Ruiz M, Calingasan NY, Starkov AA, Dumont M, Naudí A, Martínez JJ, Aubourg P, Portero-Otín M, Pamplona R, Galea E, Beal MF, Ferrer I, Fourcade S, Pujol A
Pioglitazone halts axonal degeneration in a mouse model of X-linked adrenoleukodystrophy.
Brain 136: 2432-2443 (2013)

626. López-Erauskin J, Galino J, Ruiz M, Cuezva JM, Fabregat I, Cacabelos D, Boada J, Martínez J, Ferrer I, Pamplona R, Villarroya F, Portero-Otín M, Fourcade S, Pujol A
Impaired mitochondrial oxidative phosphorylation in the peroxisomal disease X-linked adrenoleukodystrophy.
Hum Mol Genet 22: 3296-3305 (2013)

627. López-Erauskin J, Ferrer I, Galea E, Pujol A
Cyclophilin D as a potential target for antioxidants in neurodegeneration: the X-ALD case.
Biol Chem 394: 621-629 (2013)

628. Bolos M, Spuch C, Ordoñez-Gutierrez L, Wandosell F, Ferrer I, Carro E
Neurogenic effects of β-amyloid in the choroid plexus epithelial cells in Alzheimer's disease.
Cell Mol Life Sci 70: 2787-2797 (2013)

629. Aso E, Ferrer I
It may be possible to delay the onset of neurodegenerative diseases with an immunosuppressive drug (rapamycin).
Expert Opin Biol Ther 13: 1215-1219 (2013)

freeneuropathology.org

Copyright: © 2026 The author(s). This is an open access article distributed under the terms of the Creative Commons Attribution 4.0 International License (https://creativecommons.org/licenses/by/4.0/), which permits unrestricted use, distribution, and reproduction in any medium, provided the original author and source are credited, a link to the Creative Commons license is provided, and any changes are indicated. The Creative Commons Public Domain Dedication waiver (https://creativecommons.org/publicdomain/zero/1.0/) applies to the data made available in this article, unless otherwise stated.

630. Sanchez-Mut JV, Aso E, Panayotis N, Lott I, Dierssen M, Rabano A, Urdinguio RG, Fernandez AF, Astudillo A, Martin-Subero JI, Balint B, Fraga MF, Gomez A, Gurnot C, Roux JC, Avila J, Hensch TK, Ferrer I, Esteller M
DNA methylation map of mouse and human brain identifies target genes in Alzheimer's disease.
Brain 136: 3018-3027 (2013)

631. Ahmed Z, Bigio EH, Budka H, Dickson DW, Ferrer I, Ghetti B, Giaccone G, Hatanpaa KJ, Holton JL, Josephs KA, Powers J, Spina S, Takahashi H, White CL 3rd, Revesz T, Kovacs GGGlobular glial tauopathies (GGT): consensus recommendations.
Acta Neuropathol 126: 537-544 (2013)

632. Klementieva O, Aso E, Filippini D, Benseny-Cases N, Carmona M, Juvés S, Appelhans D, Cladera J, Ferrer I
Effect of Poly(propylene imine) Glycodendrimers on β-Amyloid Aggregation in Vitro and in APP/PS1 Transgenic Mice, as a Model of Brain Amyloid Deposition and Alzheimer's Disease.
Biomacromolecules 14: 3570-3580 (2013)

633. LLorens F, Carulla P, Villa A, Torres JM, Fortes P, Ferrer I, del Rio JA
PrPC regulates epidermal growth factor receptor function and cell shape dynamics in Neuro2a cels.
J Neurochem 127: 124-138 (2013)

634. Rosenkranz SC, Geissen M, Härter K, Szalay B, Ferrer I, Vogel J, Smith S, Glatzel M
Amyloid-precursor-protein-lowering small molecules for disease modifying therapy of Alzheimer's disease
PLoS One 8: e82255 (2013)

635. Torres JM, Castilla J, Pintado B, Gutiérrez-Adan A, Andréoletti O, Aguilar-Calvo P, Arroba AI, Parra-Arrondo B, Ferrer I, Manzanares J, Espinosa JC
Spontaneous generation of infectious prion disease in transgenic mice
Emerg Infect Dis 19: 1938-1947 (2013)

636. Llorens F, Ansoleaga B, Garcia-Esparcia P, Zafar S, Grau-Rivera O, López-González I, Blanco R, Carmona M, Yagüe J, Nos C, Del Río JA, Gelpí E, Zerr I, Ferrer I
PrP mRNA and protein expression in brain and PrP(c) in CSF in Creutzfeldt-Jakob disease MM1 and VV2.
Prion 7: 383-393 (2013)

637. Miñones-Moyano E, Friedländer MR, Pallares J, Kagerbauer B, Porta S, Escaramís G, Ferrer I, Estivill X, Martí E
Upregulation of a small vault RNA (svtRNA2-1a) is an early event in Parkinson disease and induces neuronal dysfunction.
RNA Biol 10: 1093-1106 (2014)

638. Ferrer I, López-González I, Carmona M, Arregui L, Dalfó E, Torrejón-Escribano B, Diehl R, Kovacs GG
Glial and neuronal tau pathology in tauopathies: characterization of disease-specific phenotypes and tau pathology progression
J Neuropathol Exp Neurol 73: 81-97 (2014)

639. Kalko SG, Paco S, Jou C, Rodríguez MA, Meznaric M, Rogac M, Jekovec-Vrhovsek M, Sciacco M, Moggio M, Fagiolari G, De Paepe B, De Meirleir L, Ferrer I, Roig-Quilis M, Munell F, Montoya J, López-Gallardo E, Ruiz-Pesini E, Artuch R, Montero R, Torner F, Nascimento A, Ortez C, Colomer J, Jimenez-Mallebrera C
Transcriptomic profiling of TK2 deficient human skeletal muscle suggests a role for the p53 signalling pathway and identifies growth and differentiation factor-15 as a potential novel biomarker for mitochondrial myopathiesBMC Genomics 15: 91 (2014)

640. Fourcade S, López-Erauskin J, Ruiz M, Ferrer I, Pujol A
Mitochondrial dysfunction and oxidative damage cooperatively fuel axonal degeneration in X-linked adrenoleukodystrophy.
Biochimie 98:143-149 (2014)

freeneuropathology.org

Copyright: © 2026 The author(s). This is an open access article distributed under the terms of the Creative Commons Attribution 4.0 International License (https://creativecommons.org/licenses/by/4.0/), which permits unrestricted use, distribution, and reproduction in any medium, provided the original author and source are credited, a link to the Creative Commons license is provided, and any changes are indicated. The Creative Commons Public Domain Dedication waiver (https://creativecommons.org/publicdomain/zero/1.0/) applies to the data made available in this article, unless otherwise stated.

641. Llorens F, Ferrer I, Del Río JA
Gene expression resulting from PrP(C) ablation and PrP (C) overexpression in murine and cellular models
Mol Neurobiol 49: 413-23 (2014)

642. Villar-Menendez I, Diaz-Sanchez S, Blanch M, Albasanz JL, Pereira-Veiga T, Monje A, Planchat LM, Ferrer I, Martin M, Barrachina M
Reduced striatal adenosine A2A receptor levels define a molecular subgroup in schizophrenia
J Psychiatr Res 51: 49-59 (2014)

643. Aso E, Ferrer I
Cannabinoids for tratment of Alzheimer's disease: moving toward the clinic
Frontiers Pharmacol 5, (37) (2014)

644. Gerschütz A, Heinsen H, Grünblatt E, Wagner AK, Bartl J, Meissner C, Fallgatter AJ, Al-Sarraj S, Troakes C, Ferrer I, Arzberger T, Deckert J, Riederer P, Fischer M, Tatschner T, Monoranu CM
Neuron-Specific Alterations in Signal Transduction Pathways associated with Alzheimer's Disease.
J Alzheimers Dis 40: 135-142 (2014)

645. Cabello J, Sämann J, Gomez-Orte E, Erazo E, Coppa T, Pujol A, Büssing I, Schultze B, Lizcano JM, Ferrer I, Baumeister R, Dalfo E
PDR-1/hParkin negatively regulates the phagocytosis of apoptotic cell corpses in Caenorhabditis elegans
Cell Death Dis 5, e1120 (2014)

646. Hoegg-Beiler MB, Sirisi S, Orozco IJ, Ferrer I, Hohensee S, Auberson M, Gödde K, Vilches C, de Heredia ML, Nunes V, Estévez R, Jentsch TJ
Disrupting MLC1 and GlialCAM and ClC-2 interactions in leukodystrophy entails glial chloride channel dysfunction.
Nature Commun 5: 3475 (2014)

647. Sepulveda-Falla D, Barrera-Ocampo A, Hagel C, Korwitz A, Vinueza-Veloz MF, Zhou K, Schonewille M, Zhou H, Velazquez-Perez L, Rodriguez-Labrada R, Villegas A, Ferrer I, Lopera F, Langer T, De Zeeuw CI, Glatzel M
Familial Alzheimer's disease-associated presenilin-1 alters cerebellar activity and calcium homeostasis.
J Clin Invest 124: 1552-1567 (2014)

648. Sanchez-Mut JV, Aso E, Heyn H, Matsuda T, Bock C, Ferrer I, Esteller M
Promoter hypermethylation of the phosphatase DUSP22 mediates PKA-dependent TAU phosphorylation and CREB activation in Alzheimer's disease.
Hippocampus 24: 363-368 (2014)

649. Parra-Damas A, Valero J, Chen M, España J, Martín E, Ferrer I, Rodríguez-Alvarez J, Saura CA
Crtc1 activates a transcriptional program deregulated at early Alzheimer's disease-related stages.
J Neurosci 34: 5776-5787 (2014)

650. Fabelo N, Martín V, Marín R, Moreno D, Ferrer I, Díaz M
Altered lipid composition in cortical lipid rafts occurs at early stages of sporadic Alzheimer's disease and facilitates APP/BACE1
Neurobiol Aging 35: 1801-1812 (2014)

651. Jové M, Portero-Otín M, Naudí A, Ferrer I, Pamplona R
Metabolomics of human brain aging and age-related neurodegenerative diseases
J Neuropathol Exp Neurol 73: 640-657 (2014)

Copyright: © 2026 The author(s). This is an open access article distributed under the terms of the Creative Commons Attribution 4.0 International License (https://creativecommons.org/licenses/by/4.0/), which permits unrestricted use, distribution, and reproduction in any medium, provided the original author and source are credited, a link to the Creative Commons license is provided, and any changes are indicated. The Creative Commons Public Domain Dedication waiver (https://creativecommons.org/publicdomain/zero/1.0/) applies to the data made available in this article, unless otherwise stated.

652. Villar-Menendez I, Porta S, Buira SP, Pereira-Vega T, Diaz-Sanchez S, Albasanz JL, Ferrer I, Martín M, Barrachina M
Increased striatal adenosine A2A receptor levels is an early event in Parkinson's disease-related pathology and it is potentially regulated by miR-34b
Neurobiol Dis 69: 206-214 (2014)

653. Llorens F, López-González I, Thüne K, Carmona M, Zafar S, Andreoletti O, Zerr I, Ferrer I Subtype and regional-specific neuroinflammation in sporadic Creutzfeldt-Jakob disease
Front Aging Neurosci 6: a198 (2014)

654. Suárez-Calvet M, Dols-Icardo O, Lladó A, Sánchez-Valle R, Hernández I, Amer G, Antón-Aguirre S, Alcolea D, Fortea J, Ferrer I, van der Zee J, Dillen L, Van Broeckhoven C, Molinuevo JL, Blesa R, Clarimón J, Lleó A
Plasma phosphorylated TDP-43 levels are elevated in patients with frontotemporal dementia carrying a C9orf72 repeat expansion or a GRN mutation
J Neurol Neurosurg Psychiatry 85: 684-691 (2014)

655. Ferrer I, Mohan P, Chen H, Castellsague J, Gómez-Baldó L, Carmona M, García N, Aguilar H, Jiang J, Skowron M, Nellist M, Ampuero I, Russi A, Lázaro C, Maxwell CA, Pujana MA
Tubers from patients with tuberous sclerosis complex are characterized by changes in microtubule biology through ROCK2 signalling
J Pathol 233: 247-257 (2014)

656. Fernández-Nogales M, Cabrera JR, Santos-Galindo M, Hoozemans JJ, Ferrer I, Rozemuller AJ, Hernández F, Avila J, Lucas JJ
Huntington's disease is a four-repeat tauopathy with tau nuclear rods
Nat Med 20: 881-885 (2014)

657. Grison A, Zucchelli S, Urzì A, Zamparo I, Lazarevic D, Pascarella G, Roncaglia P, Giorgetti A, Garcia-Esparcia P, Vlachouli C, Simone R, Persichetti F, Forrest AR, Hayashizaki Y, Carloni P, Ferrer I, Lodovichi C, Plessy C, Carninci P, Gustincich S
Mesencephalic dopaminergic neurons express a repertoire of olfactory receptors and respond to odorant-like molecules.
BMC Genomics 15(1):729 (2014)

658. Kang T, Kim JH, Hong I, Park N, Heinsen H, Lee JY, Ravid R, Ferrer I, Yoo JS, Kwon KH, Park YM.
Large-scale analysis of posttranslational modifications in the hippocampus of patients with Alzheimer's disease using pI shift and label-free quantification without enrichment
Anal Bioanal Chem 406: 5433-5446 (2014)

659. Pinacho R, Valdizán EM, Pilar-Cuellar F, Prades R, Tarragó T, Haro JM, Ferrer I, Ramos B
Increased SP4 and SP1 transcription factor expression in the postmortem hippocampus of chronic schizophrenia
J Psychiatr Res 58: 189-196 (2014)

660. Fernandez-Echevarria C, Díaz M, Ferrer I, Canerina-Amaro A, Marin R
Aβ promotes VDAC1 channel dephosphorylation in neuronal lipid rafts. Relevance to the mechanisms of neurotoxicity in Alzheimer's disease.
Neuroscience 278: 354-366 (2014)

661. Manuel I, de San Román EG, Giralt MT, Ferrer I, Rodríguez-Puertas R
Type-1 cannabinoid receptor activity during Alzheimer's disease progression.
J Alzheimers Dis 42: 761-766 (2014)

662. Porquet D, Griñan-Ferrer C, Ferrer I, Camins A, Sanfeliu C, del Valle J, Pallás M
Neuroprotective role of trans-resveratrol in a murine model of familial Alzheimer's disease
J Alzheimer's Disease 42: 1209-1220 (2014)

Copyright: © 2026 The author(s). This is an open access article distributed under the terms of the Creative Commons Attribution 4.0 International License (https://creativecommons.org/licenses/by/4.0/), which permits unrestricted use, distribution, and reproduction in any medium, provided the original author and source are credited, a link to the Creative Commons license is provided, and any changes are indicated. The Creative Commons Public Domain Dedication waiver (https://creativecommons.org/publicdomain/zero/1.0/) applies to the data made available in this article, unless otherwise stated.

663. Grau C, Arató K, Fernández-Fernández JM, Valderrama A, Sindreu C, Fillat C, Ferrer I, De la Luna S, Altafaj X
Dyrk1A-mediated phosphorylation of GluN2A at Ser 1048 regulates the Surface expression and channel activity of GluN1/GluN2A receptors.
Front Cell Neurosci 8: 331 (2014)

664. Garcia-Esparcia P, Llorens F, Carmona M, Ferrer I
Complex deregulation of cytokines and mediators of the immune response in Parkinson’s disease brain is región dependent
Brain Pathol 24: 584-598 (2014)

665. Benseny-Cases N, Klementieva O, Cotte M, Ferrer I, Cladera J
Microspectroscopy (µFTIR) Reveals Co-localization of Lipid Oxidation and Amyloid Plaques in Human Alzheimer Disease Brains.
Anal Chem 86: 12047-12054 (2014)

666. Gómez-Suaga P, Rivero-Ríos P, Fdez E, Blanca Ramírez M, Ferrer I, Aiastui A, López De Munain A, Hilfiker S
LRRK2 delays degradative receptor trafficking by impeding late endosomal budding through decreasing Rab7 activity.
Hum Mol Genet 23: 6779-6796 (2014)

667. Morató L, Bertini E, Verrigni D, Ardissone A, Ruiz M, Ferrer I, Uziel G, Pujol A
Mitochondrial dysfunction in central nervous system white matter disorders.
Glia 62: 1878-1894 (2014)

668. Villar-Menéndez I, Nuñez F, Díaz-Sánchez S, Albasanz JL, Taura J, Fernández-Dueñas V, Ferrer I, Martín M, Ciruela F, Barrachina M
Striatal adenosine A2A receptor expression is controlled by S-adenosyl-L-methionine-mediated methylation
Purinergic Signal 10: 523-528 (2014)

669. López-González I, Carmona M, Arregui L, Kovacs GG, Ferrer I
αB-crystallin and HSP27 in glial cells in tauopathies.
Neuropathology 34: 517-526 (2014)

670. Cacabelos D, Ayala V, Ramírez-Nunez O, Granado-Serrano AB, Boada J, Serrano JC, Cabré R, Nadal-Rey G, Bellmunt MJ, Ferrer I, Pamplona R, Portero-Otin M
Dietary lipid unsaturation influences survival and oxidative modifications of an amyotrophic lateral sclerosis model in a gender-specific manner.
Neuromolecular Med 16: 669-685 (2014)

671. Ansoleaga B, Jové M, Schlüter A, Garcia-Esparcia P, Moreno J, Pujol A, Pamplona R, Portero-Otín M, Ferrer I.
Deregulation of purine metabolism in Alzheimer's disease.
Neurobiol Aging 36: 68-80 (2015)

672. Ansoleaga B, Garcia-Esparcia P, Pinacho R, Haro JM, Ramos B, Ferrer I.
Decrease in olfactory and taste receptor expression in the dorsolateral prefrontal cortex in chronic schizophrenia.
J Psychiatr Res 60: 109-116 (2015)

673. Diaz M, Fabelo N, Martın V, Ferrer I, Gomez T, Marín R
Biophysical alterations in lipid rafts from human cerebral cortex associate with increased BACE1/APP interaction in early stages of Alzheimer’s disease.
J Alzheimer’s Disease 43: 1185–1198 (2015)

674. Aso E, Sánchez-Pla A, Vegas-Lozano E, Maldonado R, Ferrer I
Cannabis-Based Medicine Reduces Multiple Pathological Processes in AβPP/PS1 Mice
J Alzheimers Dis 43: 977-991 (2015)

Copyright: © 2026 The author(s). This is an open access article distributed under the terms of the Creative Commons Attribution 4.0 International License (https://creativecommons.org/licenses/by/4.0/), which permits unrestricted use, distribution, and reproduction in any medium, provided the original author and source are credited, a link to the Creative Commons license is provided, and any changes are indicated. The Creative Commons Public Domain Dedication waiver (https://creativecommons.org/publicdomain/zero/1.0/) applies to the data made available in this article, unless otherwise stated.

675. Kalia LV, Lang AE, Hazrati LN, Fujioka S, Wszolek ZK, Dickson DW, Ross OA, Van Deerlin VM, Trojanowski JQ, Hurtig HI, Alcalay RN, Marder KS, Clark LN, Gaig C, Tolosa E, Ruiz-Martínez J, Marti-Masso JF, Ferrer I, López de Munain A, Goldman SM, Schüle B, Langston JW, Aasly JO, Giordana MT, Bonifati V, Puschmann A, Canesi M, Pezzoli G, Maues De Paula A, Hasegawa K, Duyckaerts C, Brice A, Stoessl AJ, Marras C
Clinical Correlations With Lewy Body Pathology in LRRK2-Related Parkinson Disease.
JAMA Neurol 72: 100-105 (2015)

676. Porquet D, Andrés-Benito P, Griñán-Ferré C, Camins A, Ferrer I, Canudas AM, Del Valle J, Pallàs M
Amyloid and tau pathology of familial Alzheimer's disease APP/PS1 mouse model in a senescence phenotype background (SAMP8)
Age 37: 9747 (2015)

677. Fuso A, Ferraguti G, Scarpa S, Ferrer I, Lucarelli M
Disclosing Bias in Bisulfite Assay: MethPrimers Underestimate High DNA Methylation.
PLoS One 10(2):e0118318 (2015)

678. Launay N, Aguado C, Fourcade S, Ruiz M, Grau L, Riera J, Guilera C, Giròs M,Ferrer I, Knecht E, Pujol A
Autophagy induction halts axonal degeneration in a mouse model of X-adrenoleukodystrophy.
Acta Neuropathol 129: 399-415 (2015)

679. O'Callaghan MM, Emperador S, Pineda M, López-Gallardo E, Montero R, Yubero D, Jou C, Jimenez-Mallebrera C, Nascimento A, Ferrer I, García-Cazorla A, Ruiz-Pesini E, Montoya J, Artuch R
Mutation loads in different tissues from six pathogenic mtDNA point mutations.
Mitochondrion 22: 17-22 (2015)

680. López-González I, Schlüter A, Aso E, Garcia-Esparcia P, Ansoleaga B, LLorens F, Carmona M, Moreno J, Fuso A, Portero-Otin M, Pamplona R, Pujol A, Ferrer I
Neuroinflammatory Signals in Alzheimer Disease and APP/PS1 Transgenic Mice: Correlations With Plaques, Tangles, and Oligomeric Species.
J Neuropathol Exp Neuro 74: 319-344 (2015)

681. Krzyzanowska A, García-Consuegra I, Pascual C, Antequera D, Ferrer I, Carro E
Expression of regulatory proteins in choroid plexus changes in early stages of Alzheimer disease.
J Neuropathol Exp Neurol 74: 359-369 (2015)

682. Ferrer I, Legati A, García-Monco JC, Gomez-Beldarrain M, Carmona M, Blanco R, Seeley WW, Coppola G
Familial behavioral variant frontotemporal dementia associated with astrocyte-predominant tauopathy.
J Neuropathol Exp Neurol 74: 370-379 (2015)

683. Petrov D, Pedrós I, Artiach G, Sureda FX, Barroso E, Pallàs M, Casadesús G, Beas-Zarate C, Carro E, Ferrer I, Vazquez-Carrera M, Folch J, Camins A
High-fat diet-induced deregulation of hippocampal insulin signaling and mitochondrial homeostasis deficiences contribute to Alzheimer disease pathology in rodents.
Biochim Biophys Acta 1852: 1687-1699 (2015)

684. Zafar S, Schmitz M, Younus N, Tahir W, Shafiq M, Llorens F, Ferrer I, Andéoletti O, Zerr I
Creutzfeldt-Jakob Disease Subtype-Specific Regional and Temporal Regulation of ADP Ribosylation Factor-1-Dependent Rho/MLC Pathway at Pre-Clinical Stage.
J Mol Neurosci 56: 329-348 (2015)

Copyright: © 2026 The author(s). This is an open access article distributed under the terms of the Creative Commons Attribution 4.0 International License (https://creativecommons.org/licenses/by/4.0/), which permits unrestricted use, distribution, and reproduction in any medium, provided the original author and source are credited, a link to the Creative Commons license is provided, and any changes are indicated. The Creative Commons Public Domain Dedication waiver (https://creativecommons.org/publicdomain/zero/1.0/) applies to the data made available in this article, unless otherwise stated.

685. Garcia-Ovejero D, Arevalo-Martin A, Paniagua-Torija B, Florensa-Vila J, Ferrer I, Grassner L, Molina-Holgado E
The ependymal region of the adult human spinal cord differs from other species and shows ependymoma-like features.
Brain 138(Pt 6): 1583-1597 (2015)

686. Olivé M, Abdul-Hussein S, Oldfors A, González-Costello J, van der Ven PF, Fürst DO, González L, Moreno D, Torrejón-Escribano B, Alió J, Pou A, Ferrer I, Tajsharghi H
New cardiac and skeletal protein aggregate myopathy associated with combined MuRF1 and MuRF3 mutations.
Hum Mol Genet 24: 3638-3650 (2015)

687. Ho Kim J, Franck J, Kang T, Heinsen H, Ravid R, Ferrer I, Hee Cheon M, Lee JY, Shin Yoo J, Steinbusch HW, Salzet M, Fournier I, Mok Park Y
Proteome-wide characterization of signalling interactions in the hippocampal CA4/DG subfield of patients with Alzheimer's disease.
Nature Sci Rep 5:11138 (2015)

688. González de San Román E, Manuel I, Giralt MT, Chun J, Estivill-Torrús G, Rodríguez de Fonseca F, Santín LJ, Ferrer I, Rodríguez-Puertas R
Anatomical location of LPA1 activation and LPA phospholipid precursors in rodent and human brain.
J Neurochem 134: 471-485 (2015)

689. Armand-Ugón M, Aso E, Moreno J, Riera-Codina M, Sánchez A, Vegas E, Ferrer I
Memory Improvement in the AβPP/PS1 Mouse Model of Familial Alzheimer’s Disease Induced by Carbamylated-Erythropoietin is Accompanied by Modulation of Synaptic Genes.
J Alzheimers Dis 45: 407-421 (2015)

690. Llorens F, Zafar S, Ansoleaga B, Shafiq M, Blanco R, Carmona M, Grau-Rivera O, Nos C, Gelpi E, del Rio JA, Zerr I, Ferrer I
Subtype and regional regulation of prion biomarkers in sporadic Creutzfeldt-Jakob disease.
Neuropathol Appl Neurobiol 41: 631-645 (2015)

691. Carulla P, Llorens F, Matamoros-Angles A, Aguilar-Calvo P, Espinosa JC, Gavín R, Ferrer I, Legname G, Torres JM, Del Río JA
Involvement of PrP(C) in kainate-induced excitotoxicity in several mouse strains.
Nature Sci Rep 5:11971 (2015)

692. Ferrer I
Selection of controls in the study of human neurodegenerative diseases in old age.
J Neural Transm 122:941–947 (2015)

693. Strobel S, Grünblatt E, Riederer P, Heinsen H, Arzberger T, Al-Sarraj S, Troakes C, Ferrer I, Monoranu CM
Changes in the expression of genes related to neuroinflammation over the course of sporadic Alzheimer's disease progression: CX3CL1, TREM2, and PPARγ.
J Neural Transm 122: 1069-1076 (2015)

694. Alafuzoff I, Pikkarainen M, Neumann M, Arzberger T, Al-Sarraj S, Bodi I, Bogdanovic N, Bugiani O, Ferrer I, Gelpi E, Gentleman S, Giaccone G, Graeber MB, Hortobagyi T, Ince PG, Ironside JW, Kavantzas N, King A, Korkolopoulou P, Kovács GG, Meyronet D, Monoranu C, Nilsson T, Parchi P, Patsouris E, Revesz T, Roggendorf W, Rozemuller A, Seilhean D, Streichenberger N, Thal DR, Wharton SB, Kretzschmar H
Neuropathological assessments of the pathology in frontotemporal lobar degeneration with TDP43-positive inclusions: an inter-laboratory study by the BrainNet Europe consortium.
J Neural Transm 122: 957-972 (2015)

Copyright: © 2026 The author(s). This is an open access article distributed under the terms of the Creative Commons Attribution 4.0 International License (https://creativecommons.org/licenses/by/4.0/), which permits unrestricted use, distribution, and reproduction in any medium, provided the original author and source are credited, a link to the Creative Commons license is provided, and any changes are indicated. The Creative Commons Public Domain Dedication waiver (https://creativecommons.org/publicdomain/zero/1.0/) applies to the data made available in this article, unless otherwise stated.

695. Barjaoui S, Povedano M, Garcia-Esparcia P, Carmona M, Aso E, Ferrer I
Complex inflammation mRNA-related response in ALS is región dependent.
Neural Plasticity ID573784 (2015)

696. Sola I, Aso E, Frattini D, López-González I, Espargaró A, Sabaté R, Di Pietro O, Luque FJ, Clos MV, Ferrer I, Muñoz-Torrero D
Novel Levetiracetam Derivatives That Are Effective against the Alzheimer-like Phenotype in Mice: Synthesis, in Vitro, ex Vivo, and in Vivo Efficacy Studies.
J Med Chem 58: 6018-6032 (2015)

697. López-González I, Aso E, Carmona M, Armand-Ugon M, Blanco R, Naudí A, Cabré R, Portero-Otin M, Pamplona R, Ferrer I
Neuroinflammatory Gene Regulation, Mitochondrial Function, Oxidative Stress, and Brain Lipid Modifications With Disease Progression in Tau P301S Transgenic Mice as a Model of Frontotemporal Lobar Degeneration-Tau.
J Neuropathol Exp Neurol 74: 975-99 (2015)

698. Naudí A, Cabré R, Jové M, Ayala V, Gonzalo H, Portero-Otín M, Ferrer I, Pamplona R.
Lipidomics of Human Brain Aging and Alzheimer's Disease Pathology.
Int Rev Neurobiol 122:133-89 (2015)

699. Terni B, Ferrer I
Abnormal Expression and Distribution of MMP2 at Initial Stages of Alzheimer's Disease-Related Pathology.
J Alzheimers Dis 46: 461-9 (2015)

700. Aso E, Serrano AL, Muñoz-Cánoves P, Ferrer I
Fibrinogen-Derived γ377-395 Peptide Improves Cognitive Performance and Reduces Amyloid-β Deposition, without Altering Inflammation, in AβPP/PS1 Mice.
J Alzheimers Dis 47: 403-12 (2015)

701. Morató L, Ruiz M, Boada J, Calingasan NY, Galino J, Guilera C, Jové M, Naudí A, Ferrer I, Pamplona R, Serrano M, Portero-Otín M, Beal MF, Fourcade S, Pujol A
Activation of sirtuin 1 as therapy for the peroxisomal disease adrenoleukodystrophy.
Cell Death Differ 22: 1742-53 (2015)

702. Garcia-Esparcia P, Hernández-Ortega K, Ansoleaga B, Carmona M, Ferrer I
Purine metabolism gene deregulation in Parkinson's disease
Neuropathol Appl Neurobiol 41: 926-40 (2015)

703. Grau-Rivera O, Gelpi E, Nos C, Gaig C, Ferrer I, Saiz A, Lladó A, Molinuevo JL, Graus F, Sánchez-Valle R; Neurological Tissue Bank Collaborative Group
Clinicopathological Correlations and Concomitant Pathologies in Rapidly Progressive Dementia: A Brain Bank Series.
Neurodegener Dis 15:350-360 (2015)

704. Paniagua-Torija B, Arevalo-Martin A, Ferrer I, Molina-Holgado E, Garcia-Ovejero D
CB1 cannabinoid receptor enrichment in the ependymal region of the adult human spinal cord. Sci Rep 5:17745 (2015)

705. Garcia-Esparcia P, Hernández-Ortega K, Koneti A, Gil L, Delgado-Morales R, Castaño E, Carmona M, Ferrer I
Altered machinery of protein synthesis is region- and stage-dependent and is associated with α-synuclein oligomers in Parkinson's disease.
Acta Neuropathol Comm 3: 76 (2015)

Copyright: © 2026 The author(s). This is an open access article distributed under the terms of the Creative Commons Attribution 4.0 International License (https://creativecommons.org/licenses/by/4.0/), which permits unrestricted use, distribution, and reproduction in any medium, provided the original author and source are credited, a link to the Creative Commons license is provided, and any changes are indicated. The Creative Commons Public Domain Dedication waiver (https://creativecommons.org/publicdomain/zero/1.0/) applies to the data made available in this article, unless otherwise stated.

706. Zelaya MV, Pérez-Valderrama E, de Morentin XM, Tuñon T, Ferrer I, Luquin MR, Fernandez-Irigoyen J, Santamaría E
Olfactory bulb proteome dynamics during the progression of sporadic Alzheimer's disease: identification of common and distinct olfactory targets across Alzheimer-related co-pathologies.
Oncotarget 6: 39437-56 (2015)

707. Naudi A, Cabre R, Jove M, Ayala V, Gonzalo H, Portero-Otin M, Ferrer I, Pamplona R
Lipidomics of human brain aging and Alzheimer's disease pathology.
Int Rev Neurobiol 122: 133-189 (2015)

708. Nager M, Santacana M, Bhardwaj D, Valls J, Ferrer I, Nogues P, Canti C, Herreros J
Nuclear phosphorylated Y142 beta-catenin accumulates in astrocytomas and glioblastomas and regulates cell invasion.
Cell cycle 14: 3644-3655 (2015)

709. Vergara C, Ordóñez-Gutiérrez L, Wandosell F, Ferrer I, del Río JA, Gavín R
Role of PrP(C) Expression in Tau Protein Levels and Phosphorylation in Alzheimer's Disease Evolution.
Mol Neurobiol 51: 1206-1220 (2015)

710. Sanchez-Mut JV, Heyn H, Vidal E, Moran S, Sayols S, Delgado-Morales R, Schultz MD, Ansoleaga B, Garcia-Esparcia P, Pons-Espinal M, de Lagran MM, Dopazo J, Rabano A, Avila J, Dierssen M, Lott I, Ferrer I, Ecker JR, Esteller M
Human DNA methylomes of neurodegenerative diseases show common epigenomic patterns.
Transl Psychiatry 6:e718 (2016)

711. Blanch M, Mosquera JL, Ansoleaga B, Ferrer I, Barrachina M
Altered Mitochondrial DNA Methylation Pattern in Alzheimer Disease-Related Pathology and in Parkinson Disease.
Am J Pathol 186: 385-397 (2016)

712. Cacabelos D, Ramírez-Núñez O, Granado-Serrano AB, Torres P, Ayala V, Moiseeva V, Povedano M, Ferrer I, Pamplona R, Portero-Otin M, Boada J
Early and gender-specific differences in spinal cord mitochondrial function and oxidative stress markers in a mouse model of ALS.
Acta Neuropathol Commun 4:3 (2016)

713. Kovacs GG, Ferrer I, Grinberg LT, Alafuzoff I, Attems J, Budka H, Cairns NJ, Crary JF, Duyckaerts C, Ghetti B, Halliday GM, Ironside JW, Love S, Mackenzie IR, Munoz DG, Murray ME, Nelson PT, Takahashi H, Trojanowski JQ, Ansorge O, Arzberger T, Baborie A, Beach TG, Bieniek KF, Bigio EH, Bodi I, Dugger BN, Feany M, Gelpi E, Gentleman SM, Giaccone G, Hatanpaa KJ, Heale R, Hof PR, Hofer M, Hortobágyi T, Jellinger K, Jicha GA, Ince P, Kofler J, Kövari E, Kril JJ, Mann DM, Matej R, McKee AC, McLean C, Milenkovic I, Montine TJ, Murayama S, Lee EB, Rahimi J, Rodriguez RD, Rozemüller A, Schneider JA, Schultz C, Seeley W, Seilhean D, Smith C, Tagliavini F, Takao M, Thal DR, Toledo JB, Tolnay M, Troncoso JC, Vinters HV, Weis S, Wharton SB, White CL 3rd, Wisniewski T, Woulfe JM, Yamada M, Dickson DW
Aging-related tau astrogliopathy (ARTAG): harmonized evaluation strategy.
Acta Neuropathol 131: 87-102 (2016)

714. Fernández-Vega I, Pérez de Heredia-Goñi K, Santos-Juanes J, Goñi Imizcoz M, Zaldumbide L, Zarranz JJ, Ferrer I
Sporadic adult-onset leucodystrophy with axonal spheroids and pigmented glia with no mutations in the known targeted genes.
Histopathology 68: 308-312 (2016)

715. Cacabelos D, Ayala V, Granado-Serrano AB, Jove M, Torres P, Boada J, Cabre R, Ramirez-Nuñez O, Gonzalo H, Soler-Cantero A, Serrano JCE, Bellmunt MJ, Romero MP, Motilva MJ, Nonaka T, Hasegawa M, Ferrer I, Pamplona R, Portero-Otin M
Interplay between TDP-43 and docosahexaenoic acid-related processes in amyotrophic lateral sclerosis.
Neurobiol Dis 88: 148-160 (2016)

Copyright: © 2026 The author(s). This is an open access article distributed under the terms of the Creative Commons Attribution 4.0 International License (https://creativecommons.org/licenses/by/4.0/), which permits unrestricted use, distribution, and reproduction in any medium, provided the original author and source are credited, a link to the Creative Commons license is provided, and any changes are indicated. The Creative Commons Public Domain Dedication waiver (https://creativecommons.org/publicdomain/zero/1.0/) applies to the data made available in this article, unless otherwise stated.

716. Lopez-Gonzalez I, Garcia-Esparcia P, Llorens F, Ferrer I
Genetic and transcriptomic profiles of inflammation in neurodegenerative diseases: Alzheimer, Parkinson, Creutzfeldt-Jakob and tauopathies.
Int J Mol Sci 17: 206 (2016)

717. López-González I, Pérez-Mediavilla A, Zamarbide M, Carmona M, Torrejón Escribano B, Glatzel M, Galliciotti G, Ferrer I
Limited unfolded protein response and inflammation in neuroserpinopathy.
J Neuropathol Exp Neurol 75: 121-131 (2016)

718. Grau-Rivera O, Sánchez-Valle R, Bargalló N, Lladó A, Gaig C, Nos C, Ferrer I, Graus F, Gelpi E
Sporadic MM2-thalamic + cortical Creutzfeldt-Jakob disease: Utility of diffusion tensor imaging in the detection of cortical involvement in vivo.
Neuropathology 36: 199-204 (2016)

719. Llorens F, Schmitz M, Ferrer I, Zerr I
CSF biomarkers in neurodegenerative and vascular dementias
Prog Neurobiol 138-140: 36-53 (2016)

720. Aso E, Andrés-Benito P, Carmona M, Maldonado R, Ferrer I
Cannabinoid receptor 2 participates in amyloid-β processing in a mouse model of Alzheimer's disease but plays a minor role in the therapeutic properties of a cannabis-based medicine.
J Alzheimers Dis 51: 489-500 (2016)

721. Martìn-Clemente B, Alvarez-Castelao B, Mayo I, Sierra AB, Díaz V, Milán M, Fariñas I, Gómez-Isla T, Ferrer I, Castaño JG
α-Synuclein expression levels do not significantly affect proteasome function and expression in mice and stably transfected PC12 cell lines.
J Biol Chem 291: 8986 (2016)

722. Ferrer I, Climent F, Vidal N, Escobar LA, Sánchez JJ, Fernandez-Coello A
74 Year-old woman with progressive weakness.
Brain Pathol 26: 423-424 (2016).

723. Alexander J, Kalev O, Mehrabian S, Traykov L, Raycheva M, Kanakis D, Drineas P, Lutz MI, Ströbel T, Penz T, Schuster M, Bock C, Ferrer I, Paschou P, Kovacs GG
Familial early-onset dementia with complex neuropathologic phenotype and genomic background.
Neurobiol Aging 42: 199-204 (2016)

724: Aso E, Ferrer I
CB2 Cannabinoid Receptor As Potential Target against Alzheimer's Disease.
Front Neurosci 31:10: 243 (2016)

725. Domínguez M, de Oliveira E, Odena MA, Portero M, Pamplona R, Ferrer I
Redox proteomic profiling of neuroketal-adducted proteins in human brain: Regional vulnerability at middle age increases in the elderly.
Free Radic Biol Med 95: 1-15 (2016)

726. Ferrer I, Garcia-Esparcia P, Carmona M, Carro E, Aronica E, Kovacs GG, Grison A, Gustincich S.
Olfactory receptors in non-chemosensory organs: the nervous system in health and disease.
Front Aging Neurosci 8: 163 (2016)

freeneuropathology.org

Copyright: © 2026 The author(s). This is an open access article distributed under the terms of the Creative Commons Attribution 4.0 International License (https://creativecommons.org/licenses/by/4.0/), which permits unrestricted use, distribution, and reproduction in any medium, provided the original author and source are credited, a link to the Creative Commons license is provided, and any changes are indicated. The Creative Commons Public Domain Dedication waiver (https://creativecommons.org/publicdomain/zero/1.0/) applies to the data made available in this article, unless otherwise stated.

727. Pantano L, Friedländer MR, Escaramís G, Lizano E, Pallarès-Albanell J, Ferrer I, Estivill X, Martí E
Specific small-RNA signatures in the amygdala at premotor and motor stages of Parkinson's disease revealed by deep sequencing analysis.
Bioinformatics 32: 673-681 (2016)

728. Lachén-Montes M, González-Morales A, de Morentin XM, Pérez-Valderrama E, Ausín K, Zelaya MV, Serna A, Aso E, Ferrer I, Fernández-Irigoyen J, Santamaría E
An early dysregulation of FAK and MEK/ERK signaling pathways precedes the β-amyloid deposition in the olfactory bulb of APP/PS1 mouse model of Alzheimer's disease.
J Proteomics148: 149-158 (2016)

729. Ansoleaga B, Garcia-Esparcia P, Llorens F, Hernández-Ortega K, Carmona Tech M, Antonio Del Rio J, Zerr I, Ferrer I
Altered mitochondria, protein synthesis machinery, and purine metabolism are molecular contributors to the pathogenesis of Creutzfeldt-Jakob disease.
J Neuropathol Exp Neurol 75: 755-769 (2016)

730. Barrera-Ocampo A, Arlt S, Matschke J, Hartmann U, Puig B, Ferrer I, Zürbig P, Glatzel M, Sepulveda-Falla D, Jahn H
Amyloid-β precursor protein modulates the sorting of Testican-1 and contributes to its accumulation in brain tissue and cerebrospinal fluid from patients with Alzheimer disease.
J Neuropathol Exp Neurol 75: 903-916 (2016)

731. López-González I, Palmeira A, Aso E, Carmona M, Fernandez L, Ferrer I
FOXP2 expression in frontotemporal lobar degeneration-tau.
J Alzheimers Dis 54: 471-475 (2016)

732. Tavares E, Antequera D, López-González I, Ferrer I, Miñano FJ, Carro E
Potential Role of Aminoprocalcitonin in the Pathogenesis of Alzheimer Disease.
Am J Pathol 186: 2723-2735 (2016)

733. Fernández B, Fdez E, Gómez-Suaga P, Gil F, Molina-Villalba I, Ferrer I, Patel S, Churchill GC, Hilfiker S
Iron overload causes endolysosomal deficits modulated by NAADP-regulated two pore channels and RAB7A.
Autophagy 2: 1487-506 (2016)

734. Pinacho R, Vila E, Prades R, Tarrago T, Castro E, Ferrer I, Ramos B
The glial phosphorylase of glycogen isoforms is reduced in the dorsolateral prefrontal cortex in chronic schizophrenia.
Schizophrenia Res 177: 37-43 (2016)

735. Pinacho R, Villalmanzo N, Meana JJ, Ferrer I, Berengueras A, Haro JM, Villen J, Ramos B
Altered CSNK1E, FABP4 and NEFH protein levels in the dorsolateral prefrontal cortex in schizophrenia
Schizophrenia Res 177: 88-97 (2016)

736. Cabre R, Jove M, Naudi A, Ayala V, Piñol-Ripoll G, Gil-Villar MP, Dominhguez-Gonzalez M, Obis E, Berdun R, Mota-Martirell N, Portero-Otin M, Ferrer I, Pamplona R
Specific metabolomics adaptations define a differential regional vulnerability in the adult human cerebral cortex.
Front Mol Neurosci 9, 138 (2016)

737. Schmitz M, Llorens F, Pracht A, Thom T, Correia Â, Zafar S, Ferrer I, Zerr I
Regulation of human cerebrospinal fluid malate dehydrogenase 1 in sporadic Creutzfeldt-Jakob disease patients.
Aging 8: 2927-2935 (2016)

Copyright: © 2026 The author(s). This is an open access article distributed under the terms of the Creative Commons Attribution 4.0 International License (https://creativecommons.org/licenses/by/4.0/), which permits unrestricted use, distribution, and reproduction in any medium, provided the original author and source are credited, a link to the Creative Commons license is provided, and any changes are indicated. The Creative Commons Public Domain Dedication waiver (https://creativecommons.org/publicdomain/zero/1.0/) applies to the data made available in this article, unless otherwise stated.

738. Aso E, Andrés-Benito P, Ferrer I
Delineating the Efficacy of a Cannabis-Based Medicine at Advanced Stages of Dementia in a Murine Model.
J Alzheimers Dis 54: 903-912 (2016)

739. Sorolla MA, Rodríguez-Colman MJ, Vall-Llaura N, Vived C, Fernández-Nogales M, Lucas JJ, Ferrer I, Cabiscol E
Impaired PLP-dependent metabolism in brain samples from Huntington disease patients and transgenic R6/1 mice.
Metab Brain Dis 31: 579-586 (2016)

740. Ferrer I
Mini-symposium: Huntington's disease.
Brain Pathol 26: 724-725 (2016)

741. Martínez-Maldonado A, Luna-Muñoz J, Ferrer I
Incidental corticobasal degeneration.
Neuropathol Appl Neurobiol 42: 659-663 (2016)

742. Llorens F, Thüne K, Schmitz M, Ansoleaga B, Frau-Méndez MA, Cramm M, Tahir W, Gotzmann N, Berjaoui S, Carmona M, Silva CJ, Fernandez-Vega I, Zarranz JJ, Zerr I, Ferrer I
Identification of new molecular alterations in Fatal Familial Insomnia.
Hum Mol Genet 25: 2417-2436 (2016)

743. Hernández-Ortega K, Garcia-Esparcia P, Gil L, Lucas JJ, Ferrer I
Altered machinery of protein synthesis in Alzheimer's: from the nucleolus to the ribosome.
Brain Pathol 26: 593-605 (2016)

744. Cabré R, Naudí A, Dominguez-Gonzalez M, Ayala V, Jové M, Mota-Martorell N, Piñol-Ripoll G, Gil-Villar MP, Rué M, Portero-Otín M, Ferrer I, Pamplona R
Sixty years old is the breakpoint of human frontal cortex aging.
Free Radic Biol Med 103:14-22 (2016)

745. Gamir-Morralla A, Belbin O, Fortea J, Alcolea D, Ferrer I, Lleó A, Iglesias T
Kidins220 Correlates with Tau in Alzheimer's Disease Brain and Cerebrospinal Fluid.
J Alzheimers Dis 55: 1327-1333 (2017)

746. Canerina-Amaro A, Hernandez-Abad LG, Ferrer I, Quinto-Alemany D, Mesa-Herrera F, Ferri C, Puertas Avendaño RA, Diaz M, Marin R
Lipid raft ER signalosome malfunctions in menopause in Alzheimer's disease.
Frontiers in Bioscience Scholar 9: 111-126 (2017)

747. Frau-Méndez MA, Fernández-Vega I, Ansoleaga B, Blanco R, Carmona M, Antonio Del Rio J, Zerr I, Llorens F, Zarranz JJ, Ferrer I
Fatal familial insomnia: Mitochondrial and protein synthesis machinery decline in the mediodorsal thalamus.
Brain Pathol 27: 95-106 (2017)

748. Marin R, Fabelo N, Martín V, Garcia-Esparcia P, Ferrer I, Quinto-Alemany D, Díaz M
Anomalies occurring in lipid profiles and protein distribution in frontal cortex lipid rafts in dementia with Lewy bodies disclose neurochemical traits partially shared by Alzheimer's and Parkinson's diseases.
Neurobiol Aging 49: 52-59 (2017)

749. Nicolia V, Cavallaro R, López-Gonzalez I, Maccarrone M, Scarpa S, Ferrer I, Fuso A
DNA methylation profiles of selected pro-inflammatory cytokines in Alzheimer's disease.
J Neuropathol Exp Neurol 76: 27-31 (2017)

freeneuropathology.org

Copyright: © 2026 The author(s). This is an open access article distributed under the terms of the Creative Commons Attribution 4.0 International License (https://creativecommons.org/licenses/by/4.0/), which permits unrestricted use, distribution, and reproduction in any medium, provided the original author and source are credited, a link to the Creative Commons license is provided, and any changes are indicated. The Creative Commons Public Domain Dedication waiver (https://creativecommons.org/publicdomain/zero/1.0/) applies to the data made available in this article, unless otherwise stated.

750. Zafar S, Younas N, Correia S, Shafiq M, Tahir W, Schmitz M, Ferrer I, Andréoletti O, Zerr I
Strain-specific altered regulatory response of Rab7a and Tau in Creutzfeldt-Jakob disease and Alzheimer's disease.
Mol Neurobiol 54: 697-709 (2017)

751. Boada M, Anaya F, Ortiz P, Olazarán J, Shua-Haim JR, Obisesan TO, Hernández I, Muñoz J, Buendia M, Alegret M, Lafuente A, Tárraga L, Núñez L, Torres M, Grifols JR, Ferrer I, Lopez OL, Páez A
Efficacy and safety of plasma exchange with 5% albumin to modify cerebrospinal fluid and plasma amyloid-β concentrations and cognition outcomes in Alzheimer's disease patients: A multicenter, randomized, controlled clinical trial.
J Alzheimers Dis 56: 129-143 (2017)

752. Launay N, Ruiz M, Grau L, Ortega FJ, Ilieva EV, Martínez JJ, Galea E, Ferrer I, Knecht E, Pujol A, Fourcade S
Tauroursodeoxycholic bile acid arrests axonal degeneration by inhibiting the unfolded protein response in X-linked adrenoleukodystrophy.
Acta Neuropathol 133: 283-301 (2017)

753. Garcia-Esparcia P, López-González I, Grau-Rivera O, García-Garrido MF, Konetti A, Llorens F, Zafar S, Carmona M, del Rio JA, Zerr I, Ferrer I
Dementia with Lewy bodies: molecular pathology in the frontal cortex in typical and rapidly progressive forms.
Front Neurol 8: 89 (2017)

754. Naudí A, Cabré R, Dominguez-Gonzalez M, Ayala V, Jové M, Mota-Martorell N, Piñol-Ripoll G, Gil-Villar MP, Rué M, Portero-Otín M, Ferrer I, Pamplona R
Region-specific vulnerability to lipid peroxidation and evidence of neuronal mechanisms for polyunsaturated fatty acid biosynthesis in the healthy adult human central nervous system.
Biochim Biophys Acta 1862: 485-495 (2017)

755. Llorens F, Zarranz JJ, Fischer A, Zerr I, Ferrer I
Fatal Familial Insomnia: Clinical Aspects and Molecular Alterations.
Curr Neurol Neurosci Rep 17: 30 (2017)

756. Andrés-Benito P, Moreno J, Aso E, Povedano M, Ferrer I
Amyotrophic lateral sclerosis, gene deregulation in the anterior horn of the spinal cord and frontal cortex area 8: implications in frontotemporal lobar degeneration.
Aging (Albany NY). 9: 823-851 (2017)

757. López-González I, Tebé Cordomí C, Ferrer I
Regional gene expression of inflammation and oxidative stress responses does not predict neurodegeneration in aging.
J Neuropathol Exp Neurol 76: 135-150 (2017)

758. Fernandez B, Ferrer I, Gil F, Hilfiker S
Biomonitorization of iron accumulation in the substantia nigra from Lewy body disease patients.
Tocxicology Reports 4: 188-193 (2017)

759. Llorens F, Thüne K, Sikorska B, Schmitz M, Tahir W, Fernández-Borges N, Cramm M, Gotzmann N, Carmona M, Streichenberger N, Michel U, Zafar S, Schuetz AL, Rajput A, Andréoletti O, Bonn S, Fischer A, Liberski PP, Torres JM, Ferrer I, Zerr I
Altered Ca_{2+} homeostasis induces Calpain-Cathepsin axis activation in sporadic Creutzfeldt-Jakob disease.
Acta Neuropathol Commun 5: 35 (2017)

760. Fernández-Nogales M, Santos-Galindo M, Merchán-Rubira J, Hoozemans J, Rábano A, Ferrer I, Avila J, Hernández F, Lucas JJ
Tau-positive nuclear indentations in P301S tauopathy mice.
Brain Pathol 27: 314-322 (2017)

freeneuropathology.org

Copyright: © 2026 The author(s). This is an open access article distributed under the terms of the Creative Commons Attribution 4.0 International License (https://creativecommons.org/licenses/by/4.0/), which permits unrestricted use, distribution, and reproduction in any medium, provided the original author and source are credited, a link to the Creative Commons license is provided, and any changes are indicated. The Creative Commons Public Domain Dedication waiver (https://creativecommons.org/publicdomain/zero/1.0/) applies to the data made available in this article, unless otherwise stated.

761. Garcia-Esparcia P, Sideris-Lampretsas G, Hernandez-Ortega K, Grau-Rivera O, Sklaviadis T, Gelpi E, Ferrer I.
Altered mechanisms of protein synthesis in frontal cortex in Alzheimer disease and a mouse model.
Am J Neurodegener Dis 6: 15-25 (2017)

762. Zafar S, Shafiq M, Younas N, Schmitz M, Ferrer I, Zerr I
Prion Protein Interactome: Identifying Novel Targets in Slowly and Rapidly Progressive Forms of Alzheimer's Disease.
J Alzheimers Dis 59(1):265-275 (2017)

763. Gónzalez de San Román E, Manuel I, Giralt MT, Ferrer I, Rodríguez-Puertas R
Imaging mass spectrometry (IMS) of cortical lipids from preclinical to severe stages of Alzheimer's disease.
Biochim Biophys Acta 1859(9 Pt B):1604-1614 (2017)

764. Lachén-Montes M, González-Morales A, Zelaya MV, Pérez-Valderrama E, Ausín K, Ferrer I, Fernández-Irigoyen J, Santamaría E.
Olfactory bulb neuroproteomics reveals a chronological perturbation of survival routes and a disruption of prohibitin complex during Alzheimer's disease progression.
Sci Rep 2017; 7: 9115.

765. Ferrer I
Diversity of astroglial responses across human neurodegenerative disorders and brain aging.
Brain Pathol 27: 645-674 (2017)

766. Kovacs GG, Xie SX, Lee EB, Robinson JL, Caswell C, Irwin DJ, Toledo JB, Johnson VE, Smith DH, Alafuzoff I, Attems J, Bencze J, Bieniek KF, Bigio EH, Bodi I, Budka H, Dickson DW, Dugger BN, Duyckaerts C, Ferrer I, Forrest SL, Gelpi E, Gentleman SM, Giaccone G, Grinberg LT, Halliday GM, Hatanpaa KJ, Hof PR, Hofer M, Hortobágyi T, Ironside JW, King A, Kofler J, Kövari E, Kril JJ, Love S, Mackenzie IR, Mao Q, Matej R, McLean C, Munoz DG, Murray ME, Neltner J, Nelson PT, Ritchie D, Rodriguez RD, Rohan Z, Rozemuller A, Sakai K, Schultz C, Seilhean D, Smith V, Tacik P, Takahashi H, Takao M, Rudolf Thal D, Weis S, Wharton SB, White CL 3rd, Woulfe JM, Yamada M, Trojanowski JQ
Multisite Assessment of Aging-Related Tau Astrogliopathy (ARTAG).
J Neuropathol Exp Neurol 2017; 76: 605-619.

767. Andrés-Benito P, Fernández-Dueñas V, Carmona M, Escobar LA, Torrejón-Escribano B, Aso E, Ciruela F, Ferrer I
Locus coeruleus at asymptomatic early and middle Braak stages of neurofibrillary tangle pathology.
Neuropathol Appl Neurobiol 2017; 43: 373-392.

768. Urrea L, Ferrer I, Gavín R, Del Río JA
The cellular prion protein (PrP_C) as neuronal receptor for α-synuclein.
Prion 2017; 11: 226-233.

769. López-González I, Viana R, Sanz P, Ferrer I
Inflammation in Lafora disease: evolution with disease progression in laforin and malin knock-out mouse models
Mol Neurobiol 54: 3119-3130 (2017)

770. Schmitz M, Dittmar K, Llorens F, Gelpi E, Ferrer I, Schulz-Schaeffer WJ, Zerr I
Hereditary Human Prion Diseases: an Update.
Mol Neurobiol 54: 4138-4149 (2017)

771. Sanchez-Mut JV, Heyn H, Vidal E, Delgado-Morales R, Moran S, Sayols S, Sandoval J, Ferrer I, Esteller M, Gräff J
Whole Genome Grey and White matter DNA Methylation Profiles in Dorsolateral Prefrontal cortex.
Synapse 71(6) (2017)

freeneuropathology.org

Copyright: © 2026 The author(s). This is an open access article distributed under the terms of the Creative Commons Attribution 4.0 International License (https://creativecommons.org/licenses/by/4.0/), which permits unrestricted use, distribution, and reproduction in any medium, provided the original author and source are credited, a link to the Creative Commons license is provided, and any changes are indicated. The Creative Commons Public Domain Dedication waiver (https://creativecommons.org/publicdomain/zero/1.0/) applies to the data made available in this article, unless otherwise stated.

772. Nicolia V, Ciraci V, Cavallaro RA, Ferrer I, Scarpa S, Fuso A
GSK3β 5'-flanking DNA Methylation and Expression in Alzheimer's Disease Patients.
Curr Alzheimer Res 14: 753-759 (2017)

773. Gil L, Federico C, Pinedo F, Bruno F, Rebolledo AB, Montoya JJ, Olazabal IM, Ferrer I, Saccone S
Aging dependent effect of nuclear tau.
Brain Res 1677: 129-137 (2017)

774. Ferrer I, Vidal N
Neuropathology of cerebrovascular diseases.
Handb Clin Neurol 145: 79-114 (2017)

775. Fourcade S, Morató L, Parameswaran J, Ruiz M, Ruiz-Cortés T, Jové M, Naudí A, Martínez-Redondo P, Dierssen M, Ferrer I, Villarroya F, Pamplona R, Vaquero A, Portero-Otín M, Pujol A
Loss of SIRT2 leads to axonal degeneration and locomotor disability associated with redox and energy imbalance.
Aging Cell 16: 1404-1413 (2017)

776. Llorens F, Thüne K, Andrés-Benito P, Tahir W, Ansoleaga B, Hernández-Ortega K, Martí E, Zerr I, Ferrer I
MicroRNA Expression in the Locus Coeruleus, Entorhinal Cortex, and Hippocampus at Early and Middle Stages of Braak Neurofibrillary Tangle Pathology.
J Mol Neurosci 63: 206-215 (2017)

777. Andrés-Benito P, Moreno J, Domínguez R, Aso E, Povedano M, Ferrer I
Inflammatory Gene Expression in Whole Peripheral Blood at Early Stages of Sporadic Amyotrophic Lateral Sclerosis.
Front Neuro 8: 546 (2017)

778. Arena A, Iyer AM, Milenkovic I, Kovács GG, Ferrer I, Perluigi M, Aronica E
Developmental expression and dysregulation of miR-146a and miR-155 in Down's syndrome and mouse models of Down's syndrome and Alzheimer's disease.
Curr Alzheimer Res 14: 1305-1317 (2017)

779. Vanni S, Moda F, Zattoni M, Bistaffa E, De Cecco E, Rossi M, Giaccone G, Tagliavini F, Haïk S, Deslys JP, Zanusso G, Ironside JW, Ferrer I, Kovacs GG, Legname G
Differential overexpression of SERPINA3 in human prion diseases.
Sci Rep 7: 15637 (2017)

780. Reumann R, Vierk R, Zhou L, Gries F, Kraus V, Mienert J, Romswinkel E, Morellini F, Ferrer I, Nicolini C, Fahnestock M, Rune G, Glatzel M, Galliciotti G
The serine protease inhibitor neuroserpin is required for normal synaptic plasticity and regulates learning and social behavior.
Learn Mem 24: 650-659 (2017)

781. Llorens F, Thüne K, Tahir W, Kanata E, Diaz-Lucena D, Xanthopoulos K, Kovatsi E, Pleschka C, Garcia-Esparcia P, Schmitz M, Ozbay D, Correia S, Correia Â, Milosevic I, Andréoletti O, Fernández-Borges N, Vorberg IM, Glatzel M, Sklaviadis T, Torres JM, Krasemann S, Sánchez-Valle R, Ferrer I, Zerr I
YKL-40 in the brain and cerebrospinal fluid of neurodegenerative dementias.
Mol Neurodegener 12: 83 (2017)

782. Armand-Ugon M, Ansoleaga B, Berjaoui S, Ferrer I
Reduced mitochondrial activity is early and steady in the entorhinal cortex but it is mainly unmodified in the frontal cortex in Alzheimer's disease.
Curr Alzheimer Res 14: 1327-1334 (2017)

Copyright: © 2026 The author(s). This is an open access article distributed under the terms of the Creative Commons Attribution 4.0 International License (https://creativecommons.org/licenses/by/4.0/), which permits unrestricted use, distribution, and reproduction in any medium, provided the original author and source are credited, a link to the Creative Commons license is provided, and any changes are indicated. The Creative Commons Public Domain Dedication waiver (https://creativecommons.org/publicdomain/zero/1.0/) applies to the data made available in this article, unless otherwise stated.

783. Pose-Utrilla J, García-Guerra L, Del Puerto A, Martín A, Jurado-Arjona J, De León-Reyes NS, Gamir-Morralla A, Serrano ÁS, García-Gallo M, Kremer L, Fielitz J, Ireson C, Pérez-Álvarez MJ, Ferrer I, Hernández F, Ávila J, Lasa M, Campanero MR, Iglesias T
Excitotoxic inactivation of constitutive oxidative stress detoxification pathway in neurons can be rescued by PKD1.
Nat Commun 8: 2275 (2017)

784. Rodriguez-Rodriguez P, Sandebring-Matton A, Merino-Serrais P, Parrado-Fernandez C, Rabano A, Winblad B, Ávila J, Ferrer I, Cedazo-Minguez A
Tau hyperphosphorylation induces oligomeric insulin accumulation and insulin resistance in neurons.
Brain 40: 3269-3285 (2017)

785. Mata A, Urrea L, Vilches S, Llorens F, Thüne K, Espinosa JC, Andréoletti O, Sevillano AM, Torres JM, Requena JR, Zerr I, Ferrer I, Gavín R, Del Río JA
Reelin expression in Creutzfeldt-Jakob disease and experimental models of transmissible spongiform encephalopathies.
Mol Neurobiol 54: 6412-6425 (2017)

786. Garcia-Esparcia P, Koneti A, Rodríguez-Oroz MC, Lago B, Del Rio JA, Ferrer I
Mitochondrial activity in the frontal cortex area 8 and angular gyrus in Parkinson's disease and Parkinson's disease with dementia.
Brain Pathol 28: 43-57 (2018)

787. Llorens F, Thüne K, Martí E, Kanata E, Dafou D, Díaz-Lucena D, Vivancos A, Shomroni O, Zafar S, Schmitz M, Michel U, Fernández-Borges N, Andréoletti O, Del Río JA, Díez J, Fischer A, Bonn S, Sklaviadis T, Torres JM, Ferrer I, Zerr I
Regional and subtype-dependent miRNA signatures in sporadic Creutzfeldt-Jakob disease are accompanied by alterations in miRNA silencing machinery and biogenesis.
PLoS Pathog 14(1): e1006802 (2018)

788. Tahir W, Zafar S, Llorens F, Arora AS, Thüne K, Schmitz M, Gotzmann N, Kruse N, Mollenhauer B, Torres JM, Andréoletti O, Ferrer I, Zerr I
Molecular alterations in the cerebellum of sporadic Creutzfeldt-Jakob disease subtypes with DJ-1 as a key regulator of oxidative stress.
Mol Neurobiol 55: 517-537 (2018)

789. Vanni S, Zattoni M, Moda F, Giaccone G, Tagliavini F, Haïk S, Deslys JP, Zanusso G, Ironside JW, Carmona M, Ferrer I, Kovacs GG, Legname G
Hemoglobin mRNA Changes in the Frontal Cortex of Patients with Neurodegenerative Diseases.
Front Neurosci 12: 8 (2018)

790. Ferrer I
Proteomics and lipidomics in the human brain.
Handb Clin Neurol 150: 285-302 (2018)

791. Urrea L, Segura-Feliu M, Masuda-Suzukake M, Hervera A, Pedraz L, Aznar JM, Vila M, Samitier J, Torrents E, Ferrer I, Gavín R, Hagesawa M, Del Río JA
Involvement of cellular prion protein in α-synuclein transport in neurons.
Mol Neurobiol 55:1847-1860 (2018)

792. Matamoros-Angles A, Gayosso LM, Richaud-Patin Y, di Domenico A, Vergara C, Hervera A, Sousa A, Fernández-Borges N, Consiglio A, Gavín R, López de Maturana R, Ferrer I, López de Munain A, Raya Á, Castilla J, Sánchez-Pernaute R, Del Río JA
iPS cell cultures from a Gerstmann-Sträussler-Scheinker patient with the Y218N PRNP mutation recapitulate tau pathology.
Mol Neurobiol 55: 3033-3048 (2018)

freeneuropathology.org

Copyright: © 2026 The author(s). This is an open access article distributed under the terms of the Creative Commons Attribution 4.0 International License (https://creativecommons.org/licenses/by/4.0/), which permits unrestricted use, distribution, and reproduction in any medium, provided the original author and source are credited, a link to the Creative Commons license is provided, and any changes are indicated. The Creative Commons Public Domain Dedication waiver (https://creativecommons.org/publicdomain/zero/1.0/) applies to the data made available in this article, unless otherwise stated.

793. Ferrer I
Sisyphus in Neverland.
J Alzheimers Dis 62: 1023-1047 (2018)

794. Diaz M, Fabelo N, Ferrer I, Marín R
Lipid raft aging in the human frontal cortex during nonpathological aging: gender influences and potential implications in Alzehimer's disease.
Neurobiol Aging 67: 42-52 (2018)

795. Santpere G, Garcia-Esparcia P, Andres-Benito P, Lorente-Galdos B, Navarro A, Ferrer I
Transcriptional network analysis in frontal cortex in Lewy body diseases with focus on dementia with Lewy bodies.
Brain Pathol 28: 315-333 (2018)

796. Cabre R, Naudi A, Dominguez-Gonzalez M, Jove M, Ayala V, Mota-Martirell N, Portero-Otin M, Ferrer I, Pamplona R
Lipid profile in human frontal cortex is sustained throughout healthy adult life span to decay at advanced ages.
J Gerontol Biol Sci 73: 703-710 (2018)

797. Garcia-Esparcia P, Diaz-Lucena D, Ainciburu M, Torrejón-Escribano B, Carmona M, Llorens F, Ferrer I
Glutamate transporte GLT1 expression in Alzheimer's disease and Dementia with Lewy Bodies.
Front Aging Neurosci 10:122 (2018)

798. Zerr I, Schmitz M, Karch A, Villar-Piqué A, Kanata E, Golanska E, Díaz-Lucena D, Karsanidou A, Hermann P, Knipper T, Goebel S, Varges D, Sklaviadis T, Sikorska B, Liberski PP, Santana I, Ferrer I, Zetterberg H, Blennow K, Calero O, Calero M, Ladogana A, Sánchez-Valle R, Baldeiras I, Llorens F
Cerebrospinal fluid neurofilament light levels in neurodegenerative dementia: Evaluation of diagnostic accuracy in the differential diagnosis of prion diseases.
Alzheimers Dement 14: 751-763 (2018).

799. Andrés-Benito P, Delgado-Morales R, Ferrer I
Altered regulation of KIAA0566, and katanin signaling expression in the locus coeruleus with neurofibrillary tangle pathology.
Front Cell Neurosci 12: 131 (2018)

800. Andres Benito P, Gelpi E, Povedano M, Santpere G, Ferrer I
Gene expression profile in frontal córtex in sporadic frontotemporal lobar degeneration-TDP.
J Neuropathol Exp Neurol 77: 608-627 (2018)

801. Cipriani S, Ferrer I, Aronica E, Kovacs GG, Verney C, Nardelli J, Khung S, Delezoide AL, Milenkovic I, Rasika S, Manivet P, Benifla JL, Deriot N, Gressens P, Adle-Bissette H
Hippocampal radial glial subtypes and their neurogenic potential in human fetuses and healthy and Alzheimer's disease adults.
Cerebral Cortex 28: 2458-2478 (2018)

802. Sanchez-Mut JV, Heyn H, Silva BA, Dixsaut L, Garcia-Esparcia P, Vidal E, Sayols S, Glauser L, Monteagudo-Sanchez A, Perez-Tur J, Ferrer I, Monk D, Schneider B, Esteller M, Gräft J
PM20D1 is a quantitative trait locus associated with Alzheimer's disease.
Nat Medicine 24: 598-603 (2018)

803. Del Río JA, Ferrer I, Gavín R
Role of cellular prion protein in interneuronal amyloid transmission.
Prog Neurobiol 165-167:87-102 (2018)

Copyright: © 2026 The author(s). This is an open access article distributed under the terms of the Creative Commons Attribution 4.0 International License (https://creativecommons.org/licenses/by/4.0/), which permits unrestricted use, distribution, and reproduction in any medium, provided the original author and source are credited, a link to the Creative Commons license is provided, and any changes are indicated. The Creative Commons Public Domain Dedication waiver (https://creativecommons.org/publicdomain/zero/1.0/) applies to the data made available in this article, unless otherwise stated.

804. Mata-Balaguer T, Cuchillo-Ibañez I, Calero M, Ferrer I, Sáez-Valero J
Decreased generation of C-terminal fragments of ApoER2 and increased reelin expression in Alzheimer's disease.
FASEB J 32: 3536-3546 (2018)

805. Ferrer I
Astrogliopathy in tauopathies.
Neuroglia 1: 10 (2018)

806. Kanata E, Thune K, Xanthopoulos K, Ferrer I, Dafou D, Zerr I, Sklaviadis T, Llorens F.
MicroRNA alterations in the brain and body fluids of human and animal príon disease models: current status and perspectives
Front Aging Neurosci 10:e220 (2018)

807. Zafar S, Younas N, Sheikh N, Tahir W, Shafiq M, Schmitz M, Ferrer I, Andréoletti O, Zerr I
Cytoskeleton-associated risk modifiers involved in early and rapid progression of sporadic Creutzfeldt-Jakob disease.
Mol Neurobiol 55: 4009-4029 (2018)

808 Ranea-Robles P, Launay N, Ruiz M, Calingasan NY, Dumont M, Naudí A, Portero-Otín M, Pamplona R, Ferrer I, Beal MF, Fourcade S, Pujol A
Aberrant regulation of the GSK-3β/NRF2 axis unveils a novel therapy for adrenoleukodystrophy.
EMBO Mol Med 10: e8640 (2018)

809. Dominguez-Gonzalez M, Puigpinos M, Jove M, Naudi A, Portero-Otin M, Pamplona R, Ferrer I
Regional vulnerability to lipoxidative damage and inflammation in normal human aging brain
Exp Gerontol 111: 218-228 (2018)

810. Hou X, Fiesel FC, Truban D, Castanedes Casey M, Lin WL, Soto AI, Tacik P, Rousseau LG, Diehl NN, Heckman MG, Lorenzo-Betancor O, Ferrer I, Arbelo JM, Steele JC, Farrer MJ, Cornejo-Olivas M, Torres L, Mata IF, Graff-Radford NR, Wszolek ZK, Ross OA, Murray ME, Dickson DW, Springer W
Age- and disease-dependent increase of the mitophagy marker phospho-ubiquitin in normal aging and Lewy body disease.
Autophagy 14:1404-1418 (2018)

811. Torres P, Ramírez-Núñez O, Romero-Guevara R, Barés G, Granado-Serrano AB, Ayala V, Boada J, Fontdevila L, Povedano M, Sanchís D, Pamplona R, Ferrer I, Portero-Otín M
Cryptic exon splicing function of TARDBP interacts with autophagy in nervous tissue.
Autophagy 14: 1398-1403 (2018)

812. Petyuk VA, Chang R, Ramirez-Restrepo M, Beckmann ND, Henrion MYR, Piehowski PD, Zhu K, Wang S, Clarke J, Huentelman MJ, Xie F, Andreev V, Engel A, Guettoche T, Navarro L, De Jager P, Schneider JA, Morris CM, McKeith IG, Perry RH, Lovestone S, Woltjer RL, Beach TG, Sue LI, Serrano GE, Lieberman AP, Albin RL, Ferrer I, Mash DC, Hulette CM, Ervin JF, Reiman EM, Hardy JA, Bennett DA, Schadt E, Smith RD, Myers AJThe human brainome: network analysis identifies HSPA2 as a novel Alzheimer's disease target.
Brain 141: 2721-2739 (2018)

813. Ferrer I
Oligodendrogliopathy in neurodegenerative diseases with abnormal protein aggregates: the forgotten partner.
Prog Neurobiol 169: 24-54 (2018)

814. Silva-Abreu M, Calpena AC, Aso E, Romero IA, Roig-Carles D, Gromnicova R, Espinna M, Ferrer I, Garcia ML, Male D
PPARγ agonist-loaded PLGA-PEG nanocarriers as a potential treatment for Alzheimer's disease: in vitro and in vivo studsies
Int J Nanomedicine 13: 5577-5590 (2018)

Copyright: © 2026 The author(s). This is an open access article distributed under the terms of the Creative Commons Attribution 4.0 International License (https://creativecommons.org/licenses/by/4.0/), which permits unrestricted use, distribution, and reproduction in any medium, provided the original author and source are credited, a link to the Creative Commons license is provided, and any changes are indicated. The Creative Commons Public Domain Dedication waiver (https://creativecommons.org/publicdomain/zero/1.0/) applies to the data made available in this article, unless otherwise stated.

815. Fernández-Vega I, Díaz-Lucena D, Azkune Calle I, Geijo M, Juste RA, Llorens F, Vicente Etxenausia I, Santos-Juanes J, Zarranz Imirizaldu JJ, Ferrer I
Sporadic Creutzfeldt-Jakob disease with glial PrP^{Res} nuclear and perinuclear immunoreactivity.
Neuropathology 38: 561-567 (2018)

816. Vicente-Pascual M, Rossi M, Gámez J, Lladó A, Valls J, Grau-Rivera O, Ávila Polo R, Llorens F, Zerr I, Ferrer I, Nos C, Parchi P, Sanchez-Vall R, Gelpi E
Variably protease-sensitive prionopathy presenting within ALS/FTD spectrum.
Ann Clin Transl Neurol 5: 1297-1302 (2018)

817. Andrés-Benito P, Domínguez-González M, Ferrer I
Altered gene transcription linked to astrocytes and oligodendrocytes in frontal cortex in Creutzfeldt-Jakob disease.
Prion 12: 216-225 (2018)

818. Aso E, Andrés-Benito P, Ferrer I
Genetic deletion of CB_1 cannabinoid receptors exacerbates the Alzheimer-like symptoms in a transgenic animal model.
Biochem Pharmacol 157; 210-216 (2018)

819. Fleitas C, Piñol-Ripoll G, Marfull P, Rocandio D, Ferrer I, Rampon C, Egea J, Espinet CproBDNF is modified by advanced glycation end products in Alzheimer's disease and causes neuronal apoptosis by inducing p75 neurotrophin receptor processing.
Mol Brain 11: 68 (2018)

820. Alonso-Andrés P, Luis Albasanz J, Ferrer I, Martín M
Purine-related metabolites and their converting enzymes are altered in frontal, parietal, and temporal cortex at early stages of Alzheimer’s disease pathology.
Brain Pathol 28: 933-946 (2018)

821. Ferrer I, Aguiló García M, López González I, Diaz Lucena D, Roig Villalonga A, Carmona Tech M, Llorens F, Garcia-Esparcia P, Martinez-Maldonado A, Frau Mendez M, Torrejón Escribano B, Bech Serra JJ, Sabido E, de la Torre Gómez C, Del Rio JA
Aging-related tau astrogliopathy (ARTAG): Not only tau phosphorylation in astrocytes.
Brain Pathol 28: 965-985 (2018)

822. Aymerich MS, Aso E, Abellanas MA, Tolon RM, Ramos JA, Ferrer I, Romero J, Fernández-Ruiz J
Cannabinoid pharmacology/therapeutics in chronic degenerative disorders affecting the central nervous system.
Biochem Pharmacol 157: 67-84 (2018)

823. Andrés-Benito P, Domínguez R, Colomina MJ, Llorens F, Povedano M, Ferrer I
YKL40 in sporadic amyotrophic lateral sclerosis: cerebrospinal fluid levels as a prognosis marker of disease progression.
Aging (Albany NY) 10: 2367-2382 (2018)

824. Lachén-Montes M, González-Morales A, Iloro I, Elortza F, Ferrer I, Gveric D, Fernández-Irigoyen J, Santamaría E
Unveiling the olfactory proteostatic dissangement in Parkinson’s disease by proteome-wide profiling.
Neurobiol Aging 73: 123-134 (2019)

825. Pinho R, Paiva I, Jercic KG, Fonseca-Ornelas L, Gerhardt E, Fahlbusch C, Garcia-Esparcia P, Kerimoglu C, Pavlou MA, Villar-Piqué A, Szego É, Fonseca TL, Odoardi F, Soeroes S, Rego AC, Fischle W, Schwamborn JC, Meyer T, Kügler S, Ferrer I, Attems J, Fischer A, Becker S, Zweckstetter M, Borovecki F, Outeiro TF
Nuclear localization and phosphorylation modulate pathological effects of α-synuclein.
Hum Mol Genet 28: 31-50 (2019)

Copyright: © 2026 The author(s). This is an open access article distributed under the terms of the Creative Commons Attribution 4.0 International License (https://creativecommons.org/licenses/by/4.0/), which permits unrestricted use, distribution, and reproduction in any medium, provided the original author and source are credited, a link to the Creative Commons license is provided, and any changes are indicated. The Creative Commons Public Domain Dedication waiver (https://creativecommons.org/publicdomain/zero/1.0/) applies to the data made available in this article, unless otherwise stated.

826. Kanata E, Golanska E, Villar-Piqué A, Karsanidou A, Dafou D, Xanthopoulos K, Schmitz M, Ferrer I, Karch A, Sikorska B, Liberski PP, Sklaviadis T, Zerr I, Llorens F
Cerebrospinal fluid neurofilament light in suspected sporadic Creutzfeldt-Jakob disease.
J Clin Neurosci 60: 124-127 (2019)

827. Sayas CL, Medina M, Cuadros R, Ollá I, García E, Pérez M, Ferrer I, Hernández F, Avila J.
Role of tau N-terminal motif in the secretion of human tau by End-Binding proteins.
PLoS One 14: e0210864 (2019)

828. di Domenico A, Carola G, Calatayud C, Pons-Espinal M, Muñoz JP, Richaud-Patin Y, Fernandez-Carasa I, Gut M, Faella A, Parameswaran J, Soriano J, Ferrer I, Tolosa E, Zorzano A, Cuervo AM, Raya A, Consiglio A
Patient-specific iPSC-derived astrocytes contribute to non-cell-autonomous neurodegeneration in Parkinson's disease.
Stem Cell Reports12: 213-229 (2019)

829. Aso E, Martinsson I, Appelhans D, Effenberg C, Benseny-Cases N, Cladera J, Gouras G, Ferrer I, Klementieva O
Poly(propylene imine) dendrimers with histidine-maltose shell as novel type of nanoparticles for synapse and memory protection.
Nanomedicine 17: 198-209 (2019)

830. López-González I, Pinacho R, Vila È, Escanilla A, Ferrer I, Ramos B
Neuroinflammation in the dorsolateral prefrontal cortex in elderly chronic schizophrenia.
Eur Neuropsychopharmacol 29: 384-396 (2019)

831. Olivé M, Engvall M, Ravenscroft G, Cabrera-Serrano M, Jiao H, Bortolotti CA, Pignataro M, Lambrughi M, Jiang H, Forrest ARR, Benseny-Cases N, Hofbauer S, Obinger C, Battistuzzi G, Bellei M, Borsari M, Di Rocco G, Viola HM, Hool LC, Cladera J, Lagerstedt-Robinson K, Xiang F, Wredenberg A, Miralles F, Baiges JJ, Malfatti E, Romero NB, Streichenberger N, Vial C, Claeys KG, Straathof CSM, Goris A, Freyer C, Lammens M, Bassez G, Kere J, Clemente P, Sejersen T, Udd B, Vidal N, Ferrer I, Edström L, Wedell A, Laing NG
Myoglobinopathy is an adult-onset autosomal dominant myopathy with characteristic sarcoplasmic inclusions.
Nat Commun 10:1396 (2019)

832. Gazulla J, Ferrer I, Izquierdo-Alvarez S, Alvarez S, Sánchez-Alcudia R, Bestué-Cardiel M, Seral M, Benavente I, Sierra-Martínez E, Berciano J
Hereditary primary lateral sclerosis and progressive nonfluent aphasia.
J Neurol 266: 1079-1090 (2019)

833. Andrés-Benito P, Gelpi E, Povedano M, Ausín K, Fernández-Irigoyen J, Santamaría E, Ferrer I
Combined transcriptomics and proteomics in frontal cortex area 8 in frontotemporal lobar degeneration linked to C9ORF72 expansion.
J Alzheimers Dis 68: 1287-1307 (2019)

834. Andrés-Benito P, Povedano M, Torres P, Portero-Otín M, Ferrer I
Altered dynein axonemal assembly factor 1 expression in C-boutons in bulbar and spinal cord motor-neurons in sporadic amyotrophic lateral sclerosis.
J Neuropathol Exp Neurol 78: 416-425 (2019)

835. Canerina-Amaro A, Pereda D, Diaz M, Rodriguez-Barreto D, Casañas-Sánchez V, Heffer M, Garcia-Esparcia P, Ferrer I, Puertas-Avendaño R, Marin R
Differential aggregation and phosphorylation of α-synuclein in membrana compartments associated with Parkinson disease.
Front Neurosci 13: 382 (2019)

Copyright: © 2026 The author(s). This is an open access article distributed under the terms of the Creative Commons Attribution 4.0 International License (https://creativecommons.org/licenses/by/4.0/), which permits unrestricted use, distribution, and reproduction in any medium, provided the original author and source are credited, a link to the Creative Commons license is provided, and any changes are indicated. The Creative Commons Public Domain Dedication waiver (https://creativecommons.org/publicdomain/zero/1.0/) applies to the data made available in this article, unless otherwise stated.

836. Gazulla J, Ferrer I, Berciano J
Reader response: The underacknoledged PPA-ALS: a unique clinicopathologic subtype with strong heritability.
Neurology 92(12) e1366 (2019)

837. Ferrer I, Aguiló-García M, Carmona M, Andrés-Benito P, Torrejón-Escribano B, Garcia-Esparcia P, Del Rio JA
Involvement of oligodendrocytes in tau seeding and spreading in tauopathies.
Front Aging Neurosci 11:112 (2019)

838. Lachen-Montes M, Gonzalez-Morales A, Palomino M, Ausin K, Gómez-Ochoa M, Zelaya MV, Ferrer I, Perez-Mediavilla A, Fernández-Irigoyen J, Santamaria E
Early-onset molecular derangements in the olfactory bulb of Tg2576 mice: novel insight into the stress-responsive olfactory kinase dynamics in Alzheimer's disease.
Front Aging Neurosci 11:141 (2019)

839. Hernández F, Cuadros R, Ollá I, García C, Ferrer I, Perry G, Avila J
Differences in structure and function between human and murine tau.
Biochim Biophys Acta Mol Basis Dis 1865: 2024-2030 (2019)

840. Aso E, Fernández-Dueñas V, López-Cano M, Taura J, Watanabe M, Ferrer I, Luján R, Ciruela F
Adenosine A_{2A}-cannabinoid CB_1 receptor heteromers in the hippocampus: cannabidiol blunts Δ^9-tetrahydrocannabinol-induced cognitive impairment.
Mol Neurobiol 56: 5382-5391 (2019)

841. Villar-Piqué A, Schmitz M, Hermann P, Goebel S, Bunck T, Varges D, Ferrer I, Riggert J, Llorens F, Zerr I
Plasma YKL-40 in the spectrum of neurodegenerative dementia
J Neuroinflammation 16: 145 (2019)

842. Giménez-Roldán S, Morales-Asín F, Ferrer I, Spencer PS
Historical setting and neuropathology of lathyrism: Insights from the neglected 1944 report by Oliveras de la Riva.
J Hist Neurosci 3:1-26 (2019)

843. Esteve P, Rueda-Carrasco J, Inés Mateo M, Martin-Bermejo MJ, Draffin J, Pereyra G, Sandonís Á, Crespo I, Moreno I, Aso E, Garcia-Esparcia P, Gomez-Tortosa E, Rábano A, Fortea J, Alcolea D, Lleo A, Heneka MT, Valpuesta JM, Esteban JA, Ferrer I, Domínguez M, Bovolenta P
Elevated levels of Secreted-Frizzled-Related-Protein 1 contribute to Alzheimer's disease pathogenesis.
Nat Neurosci 22: 1258-1268 (2019)

844. Fernández-Dueñas V, Gómez-Soler M, Valle-León M, Watanabe M, Ferrer I, Ciruela F
Revealing adenosine A2A-dopamine D2 receptor heteromers in Parkinson's disease post-mortem brain through a new AlphaScreen-base assay.
Int J Mol Sci 20: 3600 (2019)

845. López-Aguilar J, Bassi GL, Quilez ME, Martí JD, Tavares OR, Xiol EA, Rigol M, Luque N, Guillamat R, Ferrer I, Torres A, Blanch L
Hippocampal Damage during Mechanical Ventilation in Trendelenburg Position: A Secondary Analysis of an Experimental Study on the Prevention of Ventilator-Associated Pneumonia
Shock 52: 75-82 (2019)

846. González-Fernández C, Gonzalez P, Andres-Benito P, Ferrer I, Rodríguez FJ.
Wnt signaling alterations in the human spinal cord of amyotrophic lateral sclerosis cases: spotlight on Fz2 and Wnt5a.
Mol Neurobiol 56: 6777-6791 (2019)

freeneuropathology.org

Copyright: © 2026 The author(s). This is an open access article distributed under the terms of the Creative Commons Attribution 4.0 International License (https://creativecommons.org/licenses/by/4.0/), which permits unrestricted use, distribution, and reproduction in any medium, provided the original author and source are credited, a link to the Creative Commons license is provided, and any changes are indicated. The Creative Commons Public Domain Dedication waiver (https://creativecommons.org/publicdomain/zero/1.0/) applies to the data made available in this article, unless otherwise stated.

847. Tort F, Ugarteburu O, Texidó L, Gea-Sorlí S, García-Villoria J, Ferrer-Cortès X, Arias Á, Matalonga L, Gort L, Ferrer I, Guitart-Mampel M, Garrabou G, Vaz FM, Pristoupilova A, Rodríguez MIE, Beltran S, Cardellach F, Wanders RJ, Fillat C, García-Silva MT, Ribes A
Mutations in TIMM50 cause severe mitochondrial dysfunction by targeting key aspects of mitochondrial physiology.
Hum Mutat 40: 1700-1712 (2019)

848. Kanata E, Llorens F, Dafou D, Dimitriadis A, Thüne K, Xanthopoulos K, Bekas N, Espinosa JC, Schmitz M, Marín-Moreno A, Capece V, Shormoni O, Andréoletti O, Bonn S, Torres JM, Ferrer I, Zerr I, Sklaviadis T
RNA editing alterations define manifestation of prion diseases
Proc Natl Acad Sci U S A 116: 19727-19735 (2019)

849. Erro ME, Zelaya MV, Mendioroz M, Larumbe R, Ortega-Cubero S, Lanciego JL, Lladó A, Cabada T, Tuñón T, García-Bragado F, Luquin MR, Pastor P, Ferrer I
Globular glial tauopathy caused by MAPT P301T mutation: clinical and neuropathological findings.
J Neurol 266: 2396-2405 (2019)

850. Pesini A, Iglesias E, Bayona-Bafaluy MP, Garrido-Pérez N, Meade P, Gaudó P, Jiménez-Salvador I, Andrés-Benito P, Montoya J, Ferrer I, Pesini P, Ruiz-Pesini E
Brain pyrimidine nucleotide synthesis and Alzheimer disease.
Aging 2019; 11: 8433-8462 (2019).

851. Villar-Piqué A, Schmitz M, Lachmann I, Karch A, Calero O, Stehmann C, Sarros S, Ladogana A, Poleggi A, Santana I, Ferrer I, Mitrova E, Žáková D, Pocchiari M, Baldeiras I, Calero M, Collins SJ, Geschwind MD, Sánchez-Valle R, Zerr I, Llorens F
Cerebrospinal Fluid Total Prion Protein in the Spectrum of Prion Diseases.
Mol Neurobiol 56: 2811-2821 (2019)

852. Pamplona R, Borras C, Jové M, Pradas I, Ferrer I, Viña J
Redox lipidomics to better understand brain aging and function.
Free Radic Biol Med 144: 310-321 (2019)

853. Jove M, Pradas I, Dominguez-Gonzalez M, Ferrer I
Lipids and lipoxidation in human brain aging. Mitochondrial ATP-synthase as a key lipoxidation target.
Redox Biol 23; 101082 (2019)

854. Blennow K, Dia-Lucena D, Zetterberg H, Villar-Pique A, Karch A, Vidal E, Hermann P, Schmitz M, Ferrer I, Zerr I, LLorens F
CSF neurogranin as a neuronal damage marker in CJD: a comparative study with AD.
J Neurol Neurosurg Psychiatr 90; 846-853 (2019)

855. Llorens F, Hermann P, Villar-Piqué A, Diaz-Lucena D, Nägga K, Hansson O, Santana I, Schmitz M, Schmidt C, Varges D, Goebel S, Dumurgier J, Zetterberg H, Blennow K, Paquet C, Baldeiras I, Ferrer I, Zerr I
Cerebrospinal fluid lipocalin 2 as a novel biomarker for the differential diagnosis of vascular dementia
Nat Commun 11: 619 (2020)

856. Gazulla J, Ferrer I, Berciano J
The clinical and radiological profile of primary lateral sclerosis: an annotation on its pathological and clinical background.
J Neurol 267: 574 (2020)

857. Torres P, Cacabelos D, Pairada J, Bauer KC, Boada J, Fontdevila L, Rossi C, Povedano M, Ferrer I, Pamplona R, Finlay BB, Portero-Otín M, Ayala V
Gender-specific beneficial effects of docosahexaenoic acid dietary supplementation in G93A-SOD1 amyotrophic lateral sclerosis mice.
Neurotherapeutics 17: 269-281 (2020)

Copyright: © 2026 The author(s). This is an open access article distributed under the terms of the Creative Commons Attribution 4.0 International License (https://creativecommons.org/licenses/by/4.0/), which permits unrestricted use, distribution, and reproduction in any medium, provided the original author and source are credited, a link to the Creative Commons license is provided, and any changes are indicated. The Creative Commons Public Domain Dedication waiver (https://creativecommons.org/publicdomain/zero/1.0/) applies to the data made available in this article, unless otherwise stated.

858. Del Cerro I, Villarreal MF, Abulafia C, Duarte-Abritta B, Sánchez SM, Castro MN, Bocaccio H, Ferrer I, Menchón JM, Sevlever G, Nemeroff CB, Soriano-Mas C, Guinjoan SM
Disrupted functional connectivity of the locus coeruleus in healthy adults with parental history of Alzheimer's disease.
J Psychiatr Res123: 81-88 (2020)

859. Gazulla J, Ferrer I, Berciano J
Reader response: The underacknowledged PPA-ALS: A unique clinicopathologic subtype with strong heritability.
Neurology 94: 282-283 (2020).

860. Llorens F, Villar-Piqué A, Hermann P, Schmitz M, Calero O, Stehmann C, Sarros S, Moda F, Ferrer I, Poleggi A, Pocchiari M, Catania M, Klotz S, O'Regan C, Brett F, Heffernan J, Ladogana A, Collins SJ, Calero M, Kovacs GG, Zerr I
Diagnostic accuracy of prion disease biomarkers in iatrogenic Creutzfeldt-Jakob disease.
Biomolecules 10(2) (2020)

861. Ferrer I, Zelaya MV, Aguiló García M, Carmona Tech M, López-González I, Andrés-Benito P, Lidón L, Gavín R, Garcia-Esparcia P, Del Rio JA
Relevance of host tau in tau seeding and spreading in tauopathies.
Brain Pathol 30: 298–318 (2020)

862. Gavín R, Lidón L, Ferrer I, Del Rio JA
The quest for cellular príon protein functions in the aged and neurodegenerating brain.
Cells 9: 591 (2020)

863. Jové M, Pradas I, Mota-Martorell N, Cabré R, Ayala V, Ferrer I, Pamplona R
Succination of protein thiols in human brain aging
Front Aging Neurosci 12: 52 (2020)

864. Martínez-González L, Rodríguez-Cueto C, Cabezudo D, Bartolomé F, Andres-Benito P, Ferrer I, Gil C, Martín-Requero A, Fernández-Ruiz J, Martínez A, de Lago E
Motor neuron preservation and decrease of in vivo TDP-43 phosphorylation by protein CK-1δ kinase inhibitor treatment.
Sci Rep 10: 4449 (2020)

865. Ferrer I, Andrés-Benito P, Zelaya MV, Aguirre MEE, Carmona M, Ausín K, Lachén-Montes M, Fernández-Irigoyen J, Santamaría E, Del Rio JA
Familial globular glial tauopathy linked to MAPT mutations: molecular neuropathology and seeding capacity of a prototypical mixed neuronal and glial tauopathy.
Acta Neuropathol 139: 735-771 (2020)

866. Torres P, Andrés-Benito P, Fernàndez-Bernal A, Ricart M, Ayala V, Pamplona R, Ferrer I, Portero-Otin M
Selected cryptic exons accumulate in hippocampal cell nuclei in Alzheimer's disease with and without associated TDP-43 proteinopathy.
Brain 143(3):e20 (2020)

867. Benseny-Cases N, Alvarez-Marimon E, Aso E, Carmona M, Klementieva O, Appelhans D, Ferrer I, Cladera J
In situ structural characterization of early amyloid aggregates in Alzheimer's disease transgenic mice and Octodon degus.
Sci Rep 10: 5888 (2020)

868. Guijarro IM, Garces M, Andres-Benito P, Marín B, Otero A, Barrio T, Carmona M, Ferrer I, Badiola JJ, Monzón M
Assessment of glial activation response in the progress of natural scrapie after chronic dexamethasone treatment
In J Mol Sci 21: 3231 (2020)

Copyright: © 2026 The author(s). This is an open access article distributed under the terms of the Creative Commons Attribution 4.0 International License (https://creativecommons.org/licenses/by/4.0/), which permits unrestricted use, distribution, and reproduction in any medium, provided the original author and source are credited, a link to the Creative Commons license is provided, and any changes are indicated. The Creative Commons Public Domain Dedication waiver (https://creativecommons.org/publicdomain/zero/1.0/) applies to the data made available in this article, unless otherwise stated.

869. Llorens F, Villar-Piqué A, Schmitz M, Diaz-Lucena D, Wohnlage M, Hermann P, Goebel S, Schmidt I, Glatzel M, Hauw JJ, Sikorska B, Liberski PP, Riggert J, Ferrer I, Zerr I
Plasma total prion protein as a potential biomarker for neurodegenerative dementia: diagnostic accuracy in the spectrum of prion diseases.
Neuropathol Appl Neurobiol 46: 240-254 (2020)

870. Lidón L, Urrea L, Llorens F, Gil V, Alvarez I, Diez-Fairen M, Aguilar M, Pastor P, Zerr I, Alcolea D, Lleó A, Vidal E, Gavín R, Ferrer I, Del Rio JA
Disease-specific changes in reelin protein and mRNA in neurodegenerative diseases.
Cells 9:1252 (2020)

871. Bartolomé F, Kryzanowska A, de La Cueva M, Pascual C, Antequera D, Spuch C, Villarejo-Galende A, Rabano A, Fortea J, Alcolea D, Lleó A, Ferrer I, Hardy J, Abramov AY, Carro E
Annexin 5 prevents amyloid-beta-induced toxicity in choroid plexus: implications in Alzheimer's disease.
Sci Rep 10:931 (2020)

872. Maes T, Mascaró C, Rotllant D, Pio Lufino MM, Estiarte A, Guibourt N, Cavalcanti F, Griñan-Ferré C, Pallàs M, Nadal R, Armario A, Ferrer I, Ortega A, Valls N, Fyfe M, Martinell M, Castro Palomino JC, Buesa Arjol C
Modulation of KDM1A with vafidemstat rescues memory deficit and behavioral alterations.
PLoS One 15: e0233468 (2020)

873. Duarte AC, Santos J, Costa AR, Ferreira CL, Tomás J, Quintela T, Ishikawa H, Schwerk C, Schroten H, Ferrer I, Carro E, Gonçalves I, Santos CRA
Bitter taste receptors profiling in the human blood-cerebrospinal fluid-barrier.
Biochem Pharmacol 177: 113954 (2020)

874. Ferrer I, Andrés-Benito P, Sala-Jarque J, Gil V, Del Rio JA
Capacity of seeding and spreading of argyrophilic grain disease in a wild-type murine model: comparisons with primary age-related tauopathy.
Front Mol Neurosci 13:101 (2020)

875. Monti N, Cavallaro RA, Stoccoro A, Nicolia V, Scarpa S, Kovacs GG, Fiorenza MT, Lucarelli M, Aronica E, Ferrer I, Coppedè F, Troen AM, Fuso A
CpG and non-CpG Presenilin1 methylation pattern in course of neurodevelopment and neurodegeneration is associated with gene expression in human and murine brain.
Epigenetics 15:781-799 (2020)

876. Aso E, Andrés-Benito P, Grau-Escolano J, Caltana L, Brusco A, Sanz P, Ferrer I
Cannabidiol-enriched extract reduced the cognitive impairment but not the epileptic seizures in a Lafora disease animal model.
Cannabis Cannabinoid Res 5:150-163 (2020)

877. Lopez-Perez O, Badiola JJ, Bolea R, Ferrer I, Llorens F, Martin-Buriel I
An Update on Autophagy in Prion Diseases.
Front Bioeng Biotechnol 8:975 (2020)

878. Del Rio JA, Ferrer I
Potential of Microfluidics and Lab-on-Chip Platforms to Improve Understanding of "prion-like" Protein Assembly and Behavior.
Front Bioeng Biotechnol 8:570692 (2020)

Copyright: © 2026 The author(s). This is an open access article distributed under the terms of the Creative Commons Attribution 4.0 International License (https://creativecommons.org/licenses/by/4.0/), which permits unrestricted use, distribution, and reproduction in any medium, provided the original author and source are credited, a link to the Creative Commons license is provided, and any changes are indicated. The Creative Commons Public Domain Dedication waiver (https://creativecommons.org/publicdomain/zero/1.0/) applies to the data made available in this article, unless otherwise stated.

879. Diaz-Lucena D, Escaramis G, Villar-Piqué A, Hermann P, Schmitz M, Varges D, Santana I, Del Rio JA, Martí E, Ferrer I, Baldeiras I, Zerr I, Llorens F
A new tetra-plex fluorimetric assay for the quantification of cerebrospinal fluid β-amyloid42, total-tau, phospho-tau and α-synuclein in the differential diagnosis of neurodegenerative dementia.
J Neurol 267:2567–2581 (2020)

880. Vidal-Domènech F, Riquelme G, Pinacho R, Rodriguez-Mias R, Vera A, Monje A, Ferrer I, Callado LF, Meana JJ, Villén J, Ramos B
Calcium-binding proteins are altered in the cerebellum in schizophrenia.
PLoS One 15: e0230400 (2020)

881. Younas N, Zafar S, Shafiq M, Noor A, Siegert A, Arora AS, Galkin A, Zafar A, Schmitz M, Stadelmann C, Andreoletti O, Ferrer I, Zerr I
SFPQ and tau: critical factors contributing to rapid progression in Alzheimer's disease.
Acta Neuropathol 140:317-339 (2020)

882. Lidón L, Vergara C, Ferrer I, Hernández F, Ávila J, Del Rio JA, Gavín RTau protein as a new regulator of cellular prion protein transcription.
Mol Neurobiol 57:4170–4186 (2020)

883. Fourcade S, Goicoechea L, Parameswaran J, Schlüter A, Launay N, Ruiz M, Seyer A, Colsch B, Calingasan NY, Ferrer I, Beal MF, Sedel F, Pujol A
High-dose biotin restores redox balance, energy and lipid homeostasis, and axonal health in a model of adrenoleukodystrophy.
Brain Pathol 30: 945-963 (2020)

884. Pérez-Areales FJ, Garrido M, Aso E, Bartolini M, De Simone A, Espargaro A, Ginex T, Sabaté R, Pérez B, Andrisano V, Puigoriol-Illamola D, Pallàs M, Luque FJ, Loza MI, Brea JM, Ferrer I, Ciruela F, Messeguer A, Muñoz-Torrero D
Centrally active multi-target anti-Alzheimer agents derived from the antioxidant lead CR-6.
J Med Chem 63: 9360-9390 (2020)

885. Gerovska D, Irizar H, Otaegui D, Ferrer I, López de Munanin A, Arauzo-Bravo MJ.
Genealogy of the neurodegenerative diseases base on a meta-analysis of age-stratified incidence data.
Sci Rep 20: 18293 (2020)

886. Lachén-Montes M, Mendizuri N, Ausin K, Andres-Benito P, Ferrer I, Fernández-Irigoyen J, Santamaria E
Amyotrophic lateral sclerosis is accompanied by protein derangements in the olfactory bulb-tract axis.
Int J Mol Sci 21: 8311 (2020)

887. Santpere G, Telford M, Andres-Benito P, Navarro A, Ferrer I
The presence of human herpesvirus 6 in the brain in health and disease.
Biomolecules 10: 1520 (2020)

888. Andrés-Benito P, Povedano M, Domínguez R, Marco C, Colomina MJ, López-Pérez O, Santana I, Baldeiras I, Martínez-Yelámos S, Zerr I, Llorens F, Fernández-Irigoyen J, Santamaría E, Ferrer I
Increased C-X-C motif chemokine ligand 12 levels in cerebrospinal fluid as a candidate biomarker in sporadic amyotrophic lateral sclerosis.
Int J Mol Sci 21: 8680 (2020)

889. Ferrer I, Andrés-Benito P
White matter alterations in Alzheimer's disease without concomitant pathologies.
Neuropathol Appl Neurobiol 46; 654-672 (2020)

freeneuropathology.org

Copyright: © 2026 The author(s). This is an open access article distributed under the terms of the Creative Commons Attribution 4.0 International License (https://creativecommons.org/licenses/by/4.0/), which permits unrestricted use, distribution, and reproduction in any medium, provided the original author and source are credited, a link to the Creative Commons license is provided, and any changes are indicated. The Creative Commons Public Domain Dedication waiver (https://creativecommons.org/publicdomain/zero/1.0/) applies to the data made available in this article, unless otherwise stated.

890. Del Cerro I, Martínez-Zalacaín I, Guinea-Izquierdo A, Gascón-Bayarri J, Viñas-Diez V, Urretavizcaya M, Naval-Baudin P, Aguilera C, Reñé-Ramírez R, Ferrer I, Menchón JM, Soria V, Soriano-Mas C
Locus coeruleus connectivity alterations in late-life major depressive disorder during a visual oddball task.
Neuroimage Clin 28:102482 (2020)

891. Lachén-Montes M, Mendizuri N, Ausín K, Pérez-Mediavilla A, Azkargorta M, Iloro I, Elortza F, Kondo H, Ohigashi I, Ferrer I, de la Torre R, Robledo P, Fernández-Irigoyen J, Santamaría E
Smelling the Dark Proteome: Functional Characterization of PITH Domain-Containing Protein 1 (C1orf128) in Olfactory Metabolism.
J Proteome Res 19:4826-4843 (2020)

892. Guijarro I, Garces M, Andrés-Benito P, Marín B, Otero A, Barrio T, Carmona M, Ferrer I, Badiola JJ, Monzón M
Neuroimmune response mediated by cytokines in natural scrapie after chronic dexamethasone treatment.
Biomolecules 11:204 (2021)

893. Almeida-Santos D, Duarte AC, Gonçalves I, Ferreira CL, Ferrer I, Ishikawa H, Schwerk C, Schroten H, Santos CRA
Cadaverine and spermine elicit Ca 2+ uptake in human CP cells via a trace amine-associated receptor 1 dependent pathway.
J Mol Neurosci 71: 625-637 (2021)

894. Guinea-Izquierdo A, Giménez M, Martínez-Zalacaín I, del Cerro I, Canal-Noguer P, Blasco G, Gascón J, Reñé R,Rico I, Camins A, Aguilera C, Urretavizcaya M, Ferrer I, Menchón JM, Soria V, Soriano-Mas C
Lower Locus Coeruleus MRI intensity in patients with late-life major depression.
PeerJ 9:e10828 (2021)

895. Ferrer I, Andrés-Benito P, Carmona M, Assialioui A, Povedano M
TDP-43 vasculopathy in the spinal cord in sporadic amyotrophic lateral sclerosis (sALS) and frontal cortex in sALS/FTLD-TDP.
J Neuropath Exp Neurol 80: 229-239 (2021)

896. Shafiq M, Zafar S, Younas N, Noor A, Puig B, Altmeppen HC, Schmitz M, Matschke J, Ferrer I, Glatzel M, Zerr I
Prion protein oligomers cause neuronal cytoskeletal damage in rapidly progressive Alzheimer's disease.
Mol Neurodegener 16: 11 (2021)

897. Guijarro IM, Garcés M, Andrés-Benito P, Marín B, Otero A, Barrio T, Carmona M, Ferrer I, Badiola JJ, Monzón M
Neuroimmune response mediated by cytokines in natural scrapie after chronic dexamethasone treatment.
Biomolecules 11:204 (2021)

898. Morató X, Garcia-Esparcia P, Argerich J, Llorens F, Zerr I, Paslawski W, Borràs E, Sabidó E, Petäjä-Repo UE, Fernández-Dueñas V, Ferrer I, Svenningsson P, Ciruela F
Ecto-GPR37: a potential biomarker for Parkinson's disease.
Transl Neurodegener 10:8 (2021)

899. Puigdomenech-Poch M, Martinez-Muriana A, Andres Benito P, Ferrer I, Chun J, Lopez-Vales R
Dual role of lysophosphatidic acid receptor 2 (LPA2) in amyotrophic lateral sclerosis.
Front Cell Neurosci 15: 600872 (2021)

900. Gelpi E, Aldecoa I, Lopez-Villegas D, Abellan-Vidal MT, Mercadel-Fañanas P, Fortea J, Ribosa R, Morenas E, Gomez-Anson B, Molina-Porcel L, Ximelis T, Borrego S, Antonell A, Rovelet-Lecreux A, Klotz S, Andres-Benito P, Sanchez-Valle R, Ferrer I
Atrypical astroglial TDP-43 pathology in astroglial-predominant tauopathy.
Neuropath Appl Neurobiol 47: 1109-1113 (2021)

freeneuropathology.org

Copyright: © 2026 The author(s). This is an open access article distributed under the terms of the Creative Commons Attribution 4.0 International License (https://creativecommons.org/licenses/by/4.0/), which permits unrestricted use, distribution, and reproduction in any medium, provided the original author and source are credited, a link to the Creative Commons license is provided, and any changes are indicated. The Creative Commons Public Domain Dedication waiver (https://creativecommons.org/publicdomain/zero/1.0/) applies to the data made available in this article, unless otherwise stated.

901. Diaz-Lucena D, Kruse N, Thüne K, Schmitz M, Villar-Pique A, Gomes de Cunha JE, Hermann P, Lopez-Perez O, Andres-Benito P, Ladogana A, Calero M, Vidal E, Riggert J, Pineau H, Sim V, Zetterberg H, Blenow K, del Rio JA, Marín-Moreno A, Espinosa JC, Torres JM, Sanchez-Valle R, Mollenhauer B, Ferrer I, Zerr I, Llorens F
TREM2 expression in the brain and biological fluids in prion diseases.
Acta Neuropathologica 141: 841-859 (2021)

902. Jové M, Mota-Martorell N, Torres P, Ayala V, Portero-Otón M, Ferrer I, Pamplona R
The causal role of lipoxidative damage in mitochondrial bioenergeticc dysfunction linked to Alzheimer's disease.
Life 11: 238 (2021)

903. Andres-Benito P, Gelpi E, Jove M, Mota-Martorell N, Obis E, Portero-Otin M, Povedano M, Pujol A, Pamplona R, Ferrer I
Lipid alterations in human frontal córtex in ALS-FTLD-TDP43 proteinopathy spectrum are partly related to peroxisome impairment.
Neuropathol Appl Neurobiol 47: 544-563 (2021)

904. Zerr I, Villar-Piqué A, Hermann P, Schmitz M, Varges D, Ferrer I, Riggert J, Zetterberg H, Blennow K, Llorens F
Diagnostic and prognostic value of plasma neurofilament light and total-tau in sporadic Creutzfeldt-Jakob disease.
Alzheimers Res Ther 13:86 (2021)

905. Ramírez-Nuñez O, Jové M, Torres P, Sol J, Fontdevila L, Romero-Guevara R, Andrés-Benito P, Ayala V, Rossi C, Boada J, Povedano M, Ferrer I, Pamplona R, Portero-Otin M
Nuclear lipidome is altered in amyotrophic lateral sclerosis: a pilot study.
J Neurochem 158: 482-499 (2021)

906. Diaz Gonzalez M, Buberman A, Morales M, Ferrer I, Knafo S
Aberrant synaptic PTEN in symptomatic Alzheimer's patients may link synaptic depression to network failure.
Front Synap Neurosci 13: 683290 (2021)

907. Del Puerto A, Pose-Utrilla J, Simón-García A, López-Menendez C, Jimenez AJ, Porlan E, Pajuelo LSM, Cano-García G, Marti-Prado B, Sebastián- Serrano A, Sanchez-Carralero MP, Cesca F, Schiavo G, Ferrer I, Fariñas I, Campanero MR, Iglesias T
Kidins220 deficiency causes ventriculomegaly via SNX27-retromer-dependent AQP4 degradation.
Mol Psychiatr 26: 4611-6426 (2021)

908. Lidón L, LLaó-Hierro L, Nuvulone M, Aguzzi A, Avila J, Ferrer I, Del Rio JA, Gavín R
Tau Exon 10 Inclusion by PrPC through Downregulating GSK3β Activity
Int J Mol Sci 22:5370 (2021)

909. Álvarez-Marimon E, Castillo-Michel H, Reyes-Herrera J, Seira J, Aso E, Carmona M, Ferrer I, Cladera J, Benseny-Cases N
Synchrotron X-ray Fluorescence and FTIR Signatures for Amyloid Fibrillary and Nonfibrillary Plaques.
ACS Chem Neurosci 12:1961-1971 (2021)

910. Jimenez-Marin A, Diez I, Labayru G, Sistiaga A, Caballero MC, Andres-Benito P, Sepulcre J, Ferrer I, Lopez de Munain A, Cortes JM
Transcriptional signatures of synaptic vesicle genes define myotonic dystrophy type I neurodegeneration.
Neuropathol Appl Neurobiol 47: 1092-1108 (2021)

911. Jové M, Mota-Martorell N, Torres P, Portero-Otin M, Ferrer I, Pamplona R
New insights into human prefrontal cortex aging with a lipidomics approach.
Expert Rev Proteomics 18: 333-344 (2021)

Copyright: © 2026 The author(s). This is an open access article distributed under the terms of the Creative Commons Attribution 4.0 International License (https://creativecommons.org/licenses/by/4.0/), which permits unrestricted use, distribution, and reproduction in any medium, provided the original author and source are credited, a link to the Creative Commons license is provided, and any changes are indicated. The Creative Commons Public Domain Dedication waiver (https://creativecommons.org/publicdomain/zero/1.0/) applies to the data made available in this article, unless otherwise stated.

912. Andres-Benito P, Carmona M, Doet JY, Cassard H, Andreoletti O, Ferrer I
Differential astrocyte and oligodendrocyte vulnerability in murine Creutzfeldt-Jakob disease.
Prion 15:112-120 (2021)

913. Ferrer I, Andres-Benito P, Ausin K, Pamplona R, del Rio JA, Fernandez-Irigoyen J, Santamaria E
Dysregulated protein phosphorylation: a determining condition in the continuuum of brain aging and Alzheimer's disease.
Brain Pathol 31:e12996 (2021).

914. Rossi C, Fernández A, Torres P, Ramirez-Nuñez O, Granado-Serrano AB, Fontdevila L, Povedano M, Pamplona R, Ferrer I, Portero-Otin M
Cell stress induces mislocalization of transcription factors with mitochondrial enrichment.
Int J Mol Sci 22: 8853 (2021)

915. Sol J, Jové M, Povedano M, Sproviero W, Domínguez R, Piñol-Ripoll G, Romero-Guevara R, Hye A, Al-Chalabi A, Torres P, Andres-Benito P, Area-Gómez E, Pamplona R, Ferrer I, Ayala V, Portero-Otín M
Lipidomic traits of plasma and cerebrospinal fluid in amyotrophic lateral sclerosis correlate with disease progression.
Brain Commun 3:fcab143 (2021)

916. Torrillas De la Cal A, Paniagua-Torija B, Arevalo-Martin A, Faulkes CG, Jimenez AJ, Ferrer I, Molina-Holgado E, García-Ovejero D
The structure odf the spinal cord ependymal region in adult humans is a distinctive trait among mammals.
Cells 10: 2235 (2021)

917. Blanco-Palmero VA, Rubio-Fernández M, Antequera D, Villarejo-Galende A, Molina JÁ, Ferrer I, Bartolome F, Carro E
Increased YKL-40 but not C-reactive protein levels in patients with Alzheimer's disease. Biomedicines 9: 1094 (2021)

918. Barranco N, Plá V, Alcolea D, Sánchez-Domínguez I, Fischer-Colbrie R, Ferrer I, Lleó A, Aguado F
Dense core vesicles markers in the CSF and cortical tissues of patients with Alzheimer's disease.
Transl Neurodegener 10:37 (2021)

919. Fatimat Olabiyi B, Fleitas C, Zammou B, Ferrer I, Rampon C, Egea J, Espinet C
proNGF involvement in the adult neurogenesis dysfunction in Alzheimer's disease.
Int J Mol Sci 22: 10744 (2021)

920. Benseny-Cases N, Álvarez-Marimon E, Aso E, Carmona M, Klementieva O, Appelhans D, Ferrer I, Cladera J
In situ identification and G4-PPI-His-Mal-dendrimer-induced reduction of early-stage amyloid aggregates in Alzheimer's disease transgenic mice using synchrotron-based infrared imaging.
Sci Rep11:18368 (2021)

921. Antequera D, Moneo D, Carrero L, Bartolome F, Ferrer I, Proctor G, Carro ESalivary lactoferrin expression in a mouse model of Alzheimer's disease.
Front Immunol 12:749468 (2021)

922. Picó S, Parras A, Santos-Galindo M, Pose-Utrilla J, Castro M, Fraga E, Hernández IH, Elorza A, Anta H, Wang N, Martí-Sánchez L, Belloc E, Garcia-Esparcia P, Garrido JJ, Ferrer I, Macías-García D, Mir P, Artuch R, Pérez B, Hernández F, Navarro P, López-Sendón JL, Iglesias T, Yang XW, Méndez R, Lucas JJ
CPEB alteration and aberrant transcriptome-polyadenylation lead to a treatable SLC19A3 deficiency in Huntington's disease.
Sci Transl Med 13:eabe7104 (2021)

Copyright: © 2026 The author(s). This is an open access article distributed under the terms of the Creative Commons Attribution 4.0 International License (https://creativecommons.org/licenses/by/4.0/), which permits unrestricted use, distribution, and reproduction in any medium, provided the original author and source are credited, a link to the Creative Commons license is provided, and any changes are indicated. The Creative Commons Public Domain Dedication waiver (https://creativecommons.org/publicdomain/zero/1.0/) applies to the data made available in this article, unless otherwise stated.

923. Jove M, Cabre R, Mota-Martorell N, Martin-Gari M, Obis E, Ramos P, Canales I, Galo-Licona JD, Sol J, Nogueras L, Torres P, Portero-Otin M, Ayala V, Ferrer I, Pamplona R
Age-related changes in lipidome of rat frontal cortex and cerebellum are partially reversed by methionine restriction applied in old life.
Int J Mol Sci 22:12517 (2021)

924. Cuchillo-Ibañez I, Lennol MP, Escamilla S, Mata-Balaguer T, Valverde-Vozmediano L, Lopez-Font I, Ferrer I, Sáez-Valero J
The apolipoprotein receptor LRP3 compromisesAPP levels.
Alzheimers Res Ther 13:181 (2021).

925. Andres-Benito P, Carmona M, Jordán M, Fernández-Irigoyen J, Santamaria E, Del Rio JÁ, Ferrer I
Host tau genotype specifically designs and regulates tau seeding and spreading and host tau transformation following intrahippocampal injection of identical tau AD inoculum.
Int J Mol Sci 23: 718 (2022)

926. Conte M, Medici V, Malagoli D, Chiraello A, Cirrincione A, Davin A, Chkhñadze M, Vasuri F, Legname G, Ferrer I, Vanni S, Marcon G, Poloni TE, Guaita A, Franceschi C, Salvioli S
Expression patterns of perilipins in human brain during aging and in Alzheimer's disease.
Neuropathol Appl Neurobiol 48:e12756 (2022)

927. Ranea-Robles P, Galino J, Espinosa L, Schlüter A, Ruiz M, Calingasan NY, Villarroya F, Naudí A, Pamplona R, Ferrer I, Beal MF, Portero-Otín M, Fourcade S, Pujol A
Modulation of mitochondrial and inflammatory homeostasis through RIP140 is neuroprotective in an adrenoleukodystrophy mouse model.
Neuropathol Appl Neurobiol 48 : e12747 (2022)

928. Noor A, Zafar S, Shafiq M, Younas N, Siegert A, Mann FA, Kruss S, Schmitz M, Dihazi H, Ferrer I, Zerr I
Molecular profiles of amyloid-proteoforms in typical and rapidly progressive Alzheimer's disease.
Mol Neurobiol 59: 17-34 (2022)

929. Matamoros-Angles A, Hervera A, Soriano J, Martí E, Carulla P, Llorens F, Nuvolone M, Aguzzi A, Ferrer I, Gruart A, Delgado-García JM, Del Río JA
Analysis of co-isogenic prion protein deficient mice reveals behavioral deficits, learning impairment, and enhanced hippocampal excitability.
BMC Biol 20:17 (2022)

930. Mota-Martorell N, Andres-Benito P, Martin-Gari M, Galo-Licona JD, Sol J, Fernandez-Bernal A, Portero-Otín M, Ferrer I, Jove M, Pamplona M
Selective brain regional changes in lipid profile with human aging.
GeroScience 44: 763-783 (2022)

931. Ferrer I, Andres-Benito P, Sala-Jarque J, Gil V, Del Rio JA
Corrigendum: capacity for seeding and spreading of argyrophilic grain disease in a wild-type murine model; comparisons with primary age-related tauopathy.
Front Mol Neurosci 15:870475 (2022)

932. Zattoni M, Mearelli M, Vanni S, Colini Baldeschi A, Tran TH, Ferracin C, Catania M, Moda F, Di Fede G, Giaccone G, Tagliavini F, Zanusso G, Ironside JW, Ferrer I, Legname G.
Serpin Signatures in Prion and Alzheimer's Diseases.
Mol Neurobiol 59: 3778-3799 (2022)

freeneuropathology.org

Copyright: © 2026 The author(s). This is an open access article distributed under the terms of the Creative Commons Attribution 4.0 International License (https://creativecommons.org/licenses/by/4.0/), which permits unrestricted use, distribution, and reproduction in any medium, provided the original author and source are credited, a link to the Creative Commons license is provided, and any changes are indicated. The Creative Commons Public Domain Dedication waiver (https://creativecommons.org/publicdomain/zero/1.0/) applies to the data made available in this article, unless otherwise stated.

933. Andres-Benito P, Carmona M, Jordan-Pirla M, Torrejón-Escribano B, del Rio JA, Ferrer IDysregulated protein phosphorylation as main contributor of granulovacuolar degeneration at the first stages of neurofibrillary tangle pathology
Neuroscience 2021 Oct 30;S0306-4522(21)00534-0. doi: 10.1016/j.neuroscience.2021.10.023.
518:119-140 (2023)
934. Ferrer I, Andres-Benito P, Garcia-ESparcia P, López-González I, Valiente D, Jordán-Pirla M, Carmona M, Sala.-Jarque J, Gil V, Del Rio JA
Differences in tau seeding in newborn and adult wild-type mice.
Int J Mol Sci 23:4789 (2022)

935. López-Mengual A, Segura-Feliu M, Sunyer R, Sanz-Fraile H, Otero J, Mesquida Veny F, Gil V, Hervera Abad A, Ferrer I, Soriano-Fradera J, Trepat X, Farre R, Navajas D, Del Rio JÁ
Involvement of mechanical cues in the migration of Cajal-Retzius cells in the marginal zone during neocortical development.
Frontiers 10: 886110 (2022)

936. Ferrer I
The primary microglial leukodystrophies: a review.
Int J Mol Sci 23: 6341 (2022)

937. Ferrer I, Andres-Benito P, Ausin K, Cartas-Cejudo P, Lachén-Montes M, del Rio JÁ, Fernández-Irigoyen J, Santamaría E
Dysregulated brain protein phosphorylation linked to increased human tau expression in the hTau transgenic mouse model.
Int J Mol Sci 23: 6427 (2022)

938. Aoki I, Jurado P, Nawa K, Kondo R, Yamashiro R, Matsuyama HJ, Ferrer I, Nakano S, Mori I
OLA-1, na Obg-like ATPase, integrates hunger with temperature information in sensory neurons in *C.elegans*.
Plos Genetics 18: e1010219 (2022)

939. Ferrer I
The primary microglial leukoencephalopathies.
Encyclopedia 2022 https://encyclopedia.pub/entry/24628

940. Ferrer I
Alzheimer’s disease is an inherent, natural part of human brain aging: an integral perspective.
Free Neuropathology 3: 17 (2022)
941. Antequera D, Carrero L, Cunha Alves V, Ferrer I, Hernández-Gallego J, Municio C, Carro E.
Differentially aquaporin 5 expression in submandibular glands and cerebral cortex in Alzheimer’s disease.
Biomedicines 10: 1645 (2022) https://doi.org/10.3390/biomedicines100711645

942. Ferrer I, Andres-Benito P, Ausín K, Cartas-Cejudo P, Lachén-Montes M, Del Rio JÁ, Fernández-Irigoyen J, Santamaría E
Dysregulated protein phosphorylation in a mouse modelo of FTLD-tau.
J Neuropathol Exp Neurol 81: 696-706 (2022)

943. Parameswaran J, Goicochea L, Planas-Serra L, Pastor A, Ruiz M, Calingasan NY, Guilera C, Aso E, Boada J, Pamplona R, Portero-Otin M, de La Torre R, Ferrer I, Casasnovas C, Pujol A, Fourcade S
Activation of cannabinoid receptor 2 preserves axonal health rthrough GSK-3Beta/NRF2 axis in adrenoleukodystrophy.
Acta Neuropathol 144:241-258 (2022)

944. Roemer SF, Grinberg LT, Crary JF, Seeley WW, McKee AC, Kovacs GG, Beach TG, Duyckaerts C, Ferrer I, Gelpi E, Lee EB, Revesz T, Ehite III CL, Yoshida M, Pereira FL, Whitney K, Ghayal NB, Dickson DW
Rainwater Charitable Foundation criteria for the neuropathologic diagnosis progressive supranuclear palsy.
Acta Neuropathol 144:603-614 (2022)

Copyright: © 2026 The author(s). This is an open access article distributed under the terms of the Creative Commons Attribution 4.0 International License (https://creativecommons.org/licenses/by/4.0/), which permits unrestricted use, distribution, and reproduction in any medium, provided the original author and source are credited, a link to the Creative Commons license is provided, and any changes are indicated. The Creative Commons Public Domain Dedication waiver (https://creativecommons.org/publicdomain/zero/1.0/) applies to the data made available in this article, unless otherwise stated.

945. Torres P, Anerillas C, Ramirez-Nuñez O, Fernandez A, Encinas M, Povedano M, Andres-Benito P, Ferrer I, Ayala V, Pamplona R, Portero-Otin M
The motor neuron disease mouse model hSOD1-G93A shows a non-canonical profile of senescence biomarkers.
Dis Model Mech 15(8):dmm049059 (2022)

946. Gelpi E, Baiardi S, Nos C, Delavalle S, Aldecoa I, Ruiz-Garcia R, Ispierto L, Escudero D, Casado V, Barranco E, Boltes A, Molina-Porcel L, Bargallo N, Rossi M, Mammana A, Tiple D, Vaianella L, Stoegman E, Simonnitsch-Klupp I, Kasprian G, Klotz S, Höftberger R, Budka HG, Kovacs GG, Ferrer I, Capellari S, Sanchez-Valle R, Parchi P
Sporadic Creutzfeldt-Jakob disease VM1: phenotypic and molecular characterization of a novel subtype of human prion disease.
Acta Neuropathol Commun 10:114 (2022)

947. Jou C, Nascimiento A, Codina A, Montoya J, Lopez-Gallardo E, Emperador S, Ruiz-Pesini E, Montero R, Natera-de-Benito D, Ortez CI, Marquez J, Zelaya MV, Gutierrez-Mata A, Badosa C, Carrera-Garcia L, Exposito-Escudero J, Roldan M, Camara Y, Marti R, Ferrer I, Jimenez-Mallebrera C, Artuch R
Pathological features in paediatric patients with TK2 deficiency.
Int J Mol Sci 23:11002 (2022)

948. Assialioui A, Dominguez R, Ferrer I, Andres-Benito P, Povedano M
Elevated cerebrospinal fluid proteins and albumine determined a poor prognosis for spinal amyotrophic lateral sclerosis.
Int J Mol Sci 23: 11063 (2022)

949. Sala-Jurque J, Zimkowska K, Avila J, Ferrer I, Del Rio JA
Towards a mechanistic model of tau-mediated pathology in tauopathies: what can we learn from cell-based in vitro assays?.
Int J Mol Sci 23: 11527 (2022)

950. Ferrer I, Andres-Benito P, Carmona M, Del Rio JA
Common and specific marks of different tau strains following intra-hippocampal injection of AD, PiD, and GGT inoculum in hTau transgenic mice.
Int J Mol Sci 23: 15940 (2022).

951. Hernández F, Ferrer I, Pérez M, Zabala JC, Del Rio JA, Avila J
Tau aggregation.
Neuroscience 518:64-69 (2023).

952. Ferrer I
Hypothesis review: Alzheimer's overture guidelines
Brain Pathol 33:e13122 (2023)

953. Jové M, Mota-Martorell N, Obis E, Sol J, Martín-Garí M, Ferrer I, Portero-Otin M, Pamplona R
Ether lipid-mediated antioxidant defense in Alzheimer's disease.
Antioxidants 12, 293 (2023).

954. Jové M, Mota-Martorell N, Obis E, Sol J, Martín-Garí M, Ferrer I, Portero-Otin M, Pamplona R
Lipid adaptations against oxidative challenge in the healthy adult human brain.Antioxidants 12: 177 (2023)

955. Paz Cartas-Cejudo P, Lachen-Montes M, Ferrer I, Fernandez-Irigoyen J, Santamaria E
Sex-divergent effects on the NAD+-dependent deacetylase sirtuin signaling across the olfactory–entorhinal–amygdaloid axis in Alzheimer's and Parkinson's diseases.
Biol Sex Differences 14:5 (2023).

freeneuropathology.org

Copyright: © 2026 The author(s). This is an open access article distributed under the terms of the Creative Commons Attribution 4.0 International License (https://creativecommons.org/licenses/by/4.0/), which permits unrestricted use, distribution, and reproduction in any medium, provided the original author and source are credited, a link to the Creative Commons license is provided, and any changes are indicated. The Creative Commons Public Domain Dedication waiver (https://creativecommons.org/publicdomain/zero/1.0/) applies to the data made available in this article, unless otherwise stated.

956. Planas-Serra L, Launay N, Goicoechea L, Heron B, Jou C, Juliá-Palacios N, Ruiz M, Fourcade S, Casasnovas C, De La Torre C, Gelot A, Marsal M, Loza-Alvarez P, García-Cazorla À, Fatemi A, Ferrer I, Portero-Otin M, Area-Gómez E, Pujol A
Sphingolipid desaturase DEGS1 is essential for mitochondria-associated membrane integrity.
J Clin Invest 23:e162957 (2023). doi: 10.1172/JCI162957.

957. Andres-Benito P, Flores A, Busquet-Areny S, Carmona M, Ausin K, Cartas-Cejudo P, Lachén-Montes M, del Rio JA, Fernandez-Irigoyen J, Santamaria J, Ferrer I
Deregulated transcription and proteostasis in adult mapt knockout mouse.
Int J Mol Sci 24: 6559 (2023)

958. Ferrer I
The unique neuropathological vulnerability of the human brain to aging.
Aging Res Reviews 87:101916 (2023)

959. Andrés-Benito P, Íñigo-Marco I, Brullas M, Carmona M, Rio JAD, Fernández-Irigoyen J, Santamaría E, Povedano M, Ferrer I
Proteostatic modulation in brain aging without associated Alzheimer’s disease-and age-related neuropathological changes.
Aging (Albany NY) 15:3295-3330 (2023)

960. Alves VC, Figueiro-Silva J, Ferrer I, Carro E
Epigenetic silencing of OR and TAS2R genes expression in human orbitofrontal cortex at early stages of sporadic Alzheimer’s disease.
Cell Mol Life Sci 80(8):196 (2023)

961. Obis E, Sol J, Andres-Benito P, Martín-Gari M, Mota-Martorell N, Galo-Licona JD, Piñol-Ripoll G, Portero-Otin M, Ferrer I, Jové M, Pamplona R
Lipidomic Alterations in the Cerebral Cortex and White Matter in Sporadic Alzheimer's Disease.
Aging Dis 14: 1887-1916 (2023)

962. Vidal-Palencia L, Font C, Rebollada-Merino A, Santpere G, Andres-Benito P, Ferrer I, Pumarola M
Primary feline tauopathy: clinical, morphological, immunohistochemical, and genetic studies.
Animals 13: 2985 (2023)

963. Brewer MK, Torres P, Ayala V, Portero-Otin M, Pamplona R, Andrés-Benito P, Ferrer I, Guinovart JJ, Duran J
Glycogen accumulation modulates life span in a mouse model of amyotrophic lateral sclerosis.
J Neurochem 168:744-759 (2024)

964. Ruiz-Gabarre D, Vallés-Saiz L, Carnero-Espejo A, Ferrer I, Hernández F, Garcia-Escudero R, Ávila J, García-Escudero V.
Intron retention as a productive mechanism in human MAPT: RNA species generated by retention of intron 3.
EBioMedicine 100:104953 (2024) 965. Díaz M, Fabelo N, Martín MV, Santos G, Ferrer I
Evidence for alterations in lipid profiles and biophysical properties of lipid rafts from spinal cord in sporadic amyotrophic lateral sclerosis.
J Mol Med (Berl) 102:391-402 (2024)

966. Pons-Espinal M, Blasco-Agell L, Fernandez-Carasa I, Andrés-Benito P, di Domenico A, Richaud-Patin Y, Baruffi V, Marruecos L, Espinosa L, Garrido A, Tolosa E, Edel MJ, Otero MJ, Mosquera JL, Ferrer I, Raya A, Consiglio ABlocking IL-6 signaling prevents astrocyte-induced neurodegeneration in an iPSC-based model of Parkinson’s disease.
JCI Insight 9(3):e163359 (2024)

Copyright: © 2026 The author(s). This is an open access article distributed under the terms of the Creative Commons Attribution 4.0 International License (https://creativecommons.org/licenses/by/4.0/), which permits unrestricted use, distribution, and reproduction in any medium, provided the original author and source are credited, a link to the Creative Commons license is provided, and any changes are indicated. The Creative Commons Public Domain Dedication waiver (https://creativecommons.org/publicdomain/zero/1.0/) applies to the data made available in this article, unless otherwise stated.

967. Younas A, Younas N, Iqbal MJ, Ferrer I, Zerr I.
Comparactive interactome mapping of Tau-proterin in classical and rapidly progressive Alzheimer's disease identifies subtype-specific pathways.
Neuropathol Appl Neurobiol 50:e12964 (2024)

968. Victoria Cunha Alves V, Figueiro-Silva J, Trullas R, Ferrer I, Carro E
Olfactory receptor OR2K2 expression in human choroid plexus as a potential marker in early sporadic Alzheimer's disease.
Genes 15: 385 (2024)

969. Andrés-Benito P, Vázquez-Costa JF, Ñungo Garzón NC, Colomina MJ, Marco C, González L, Terrafeta C, Domínguez R, Ferrer I, Povedano M
Neurodegeneration biomarkers in adult spinal muscular atrophy (SMA) patients treated with Nusinersen
Int J Mol Sci 25: 3810 (2024)

970. Maturana-Quijada P, Chavarría-Elizondo P, Del Cerro I, Martínez-Zalacaín I, Juaneda-Seguí A, Guinea-Izquierdo A, Gascón-Bayarri J, Reñé R, Urretavizcaya M, Menchón JM, Ferrer I, Soria V, Soriano-Mas C
Effective connectivity of the locus coeruleus in patients with late-life Major Depressive Disorder or mild cognitive impairment.
Span J Psychiatry Ment Health 2024 Mar 6:S2950-2853(24)00015-2. doi: 10.1016/j.sjpmh.2024.02.002.

971. Ferrer I
Historical review: The golden age of the Golgi method in human neuropathology.
J Neuropathol Exp Neurol 83: 375–395 (2024).

972. Jacome D, Cotrufo T, Andres-Benito P, Lidón L, Martí E, Ferrer I, del Rio JA, Gavin R
miR-519a-3p found to regulate cellular prion protein during Alzheimer's disease pathogenesis: as a biomarker of asymptomatic stages.
BBA - Molecular Basis of Disease 1870: 167187 (2024)

973. Song F, Kovac V, Mohammadi B, Littau JL, Scharfenberg F, Matamoros Angles A, Vanni I, Shafiq M, Orge L, Galliciotti G, Djakkani S, Linsenmeier L, Černilec M, Hartman K, Jung S, Tatzelt J, Neumann JE, Damme M, Tschirner SK, Lichtenthaler SF, Ricklefs FL, Sauvigny T, Schmitz M, Zerr I, Puig B, Tolosa E, Ferrer I, Magnus T, Rupnik MS, Sepulveda-Falla D, Matschke J, Šmid LM, Bresjanac M, Andreoletti O, Krasemann S, Foliaki ST, Nonno R, Becker-Pauly C, Monzo C, Crozet C, Haigh CL, Glatzel M, Curin Serbec V, Altmeppen HCCleavage site-directed antibodies reveal the prion protein in humans is shed by ADAM10 at Y226 and associates with misfolded protein deposits in neurodegenerative diseases.
Acta Neuropathol 148:2 (2024)
974. Ferrer I
Alzheimer's Disease Neuropathological Change in Aged Non-Primate Mammals.
Int J Mol Sci 25: 8118 (2024)

975. Ferrer I
Amyloid-β Pathology Is the Common Nominator Proteinopathy of the Primate Brain Aging.
J Alzheimers Dis 100(s1):S153-S164 (2024)

976. Chavarría-Elizondo P, Del Río-Torné C, Maturana-Quijada P, Martínez-Zalacaín I, Juaneda A, Del Cerro I, Guinea-Izquierdo A, Gascón-Bayarri J, Reñé-Ramírez R, Urretavizcaya M, Ferrer I, Menchón JM, Soria V, Soriano-Mas C A

Altered activation of the dorsal anterior cingulate cortex during oddball performance in individuals at risk for Alzheimer's disease
J Psychiatry Ment Health 18:274-280 (2025)

Copyright: © 2026 The author(s). This is an open access article distributed under the terms of the Creative Commons Attribution 4.0 International License (https://creativecommons.org/licenses/by/4.0/), which permits unrestricted use, distribution, and reproduction in any medium, provided the original author and source are credited, a link to the Creative Commons license is provided, and any changes are indicated. The Creative Commons Public Domain Dedication waiver (https://creativecommons.org/publicdomain/zero/1.0/) applies to the data made available in this article, unless otherwise stated.

977. Lam I, Ndayisaba A, Lewis AJ, Fu Y, Sagredo GT, Kuzkina A, Zaccagnini L, Celikag M, Sandoe J, Sanz RL, Vahdatshoar A, Martin TD, Morshed N, Ichihashi T, Tripathi A, Ramalingam N, Oettgen-Suazo C, Bartels T, Boussouf M, Schäbinger M, Hallacli E, Jiang X, Verma A, Tea C, Wang Z, Hakozaki H, Yu X, Hyles K, Park C, Wang X, Theunissen TW, Wang H, Jaenisch R, Lindquist S, Stevens B, Stefanova N, Wenning G, van de Berg WDJ, Luk KC, Sanchez-Pernaute R, Gómez-Esteban JC, Felsky D, Kiyota Y, Sahni N, Yi SS, Chung CY, Stahlberg H, Ferrer I, Schöneberg J, Elledge SJ, Dettmer U, Halliday GM, Bartels T, Khurana V
Rapid iPSC inclusionopathy models shed light on formation, consequence, and molecular subtype of α-synuclein inclusions.
Neuron 112:2886-2909.e16 (2024) Erratum: Neuron (2025) Feb 1:S0896-6273(25)00044-3.

978. Cunha Alves V, Figueiro-Silva J, Trullas R, Ferrer I, Carro E
Olfactory Receptor OR2K2 Expression in Human Choroid Plexus as a Potential Marker in Early Sporadic Alzheimer's Disease.
Genes 15:385 (2024)

979. Sala-Jarque J, Gil V, Andrés-Benito P, Martínez-Soria I, Picón-Pagès P, Hernández F, Ávila J, Lanciego JL, Nuvolone M, Aguzzi A, Gavín R, Ferrer I, Del Río JA
The cellular prion protein does not affect tau seeding and spreading of sarkosyl-insoluble fractions from Alzheimer's disease.
Sci Rep 14:21622 (2024)

980. Torres P, Rico-Rios S, Ceron-Codorniu M, Santacreu-Vilaseca M, Seoane-Miraz D, Jad Y, Ayala V, Mariño G, Beltran M, Miralles MP, Andrés-Benito P, Fernandez-Irigoyen J, Santamaria E, López-Otín C, Soler RM, Povedano M, Ferrer I, Pamplona R, Wood MJA, Varela MA, Portero-Otin M
TDP-43 regulates LC3ylation in neural tissues through ATG4B cryptic splicing.
Acta Neuropathol 148: 45 (2024)

981. Assialioui A, Marco-Pascual C, Torrente-Segarra V, Domínguez R, Santos N, Peñafiel J, Juanola X, Povedano M, Ferrer IMicrovascular abnormalities in skin capillaries of individuals with amyotrophic lateral sclerosis.
Sci Rep 14(1):24648 (2024)

982. Andreu Matamoros-Angles A, Karadjuzovic E, Mohammadi B, Song F, Brenna S, Caroline Meister S, Siebels B, Vos H, Seuring C, Ferrer I, Schluter H, Kneussel M, Altmeppen HC, Schweizer M, Puig B, Shafiq M, Glatzel M
Efficient enzyme-free isolation of brain-derived extracellular vesicles.
J Extracell Vesicles 13:e70011 (2024)

983. Schweighauser M, Shi Y, Murzin AG, Garringer HJ, Vidal R, Murrell JR, Erro ME, Seelaar H, Ferrer I, van Swieten JC, Ghetti B, Scheres SHW, Goedert M
Distinct tau filament folds in human MAPT mutants P301L and P301T.
Nat Struct Mol Biol 32::1470-1478 (2025)

984. Chavarría-Elizondo P, Maturana-Quijada P, Martínez-Zalacaín I, del Cerro I, Juaneda-Seguí A, Guinea-Izquierdo A, Gascon-Bayarri J, Rene-Ramírez R, Urretavizcaya M, Ferrerj I, Menchon JM, Soria V, Soriano-Mas C, a Psychiatry and Mental Health Group, Neuroscience Program, Institut d'Investigació Biomèdica de Bellvitge
Altered resting-state functional connectivity in individuals at risk for Alzheimer's disease: a longitudinal studyInt J Clin Health Psychol 25: 100588 (2025)

985. Ramírez-Núñez O, Ceron-Codorniu M, Rico-Ríos S, Andrés-Benito P, Torres P, Ayala V, Vinyals A, Fernàndez-Bernal A, Maqsood S, Ferrer I, Pamplona R, Portero-Otin M
Nuclear pore complex dysfunction drives TDP-43 pathology in ALS.
Redox Biology 86:103824 (2025)

986. Raia T, Cavallaro RA, Diniz Ferreira Borges L, Cinti S, Bizzarri M, Ferrer I, Lucarelli M, Fuso A
One-carbon metabolism modulates miR-29a–DNA methylation crosstalk in Alzheimer's disease.
Alzheimer's Dement 21:e70703 (2025)

Copyright: © 2026 The author(s). This is an open access article distributed under the terms of the Creative Commons Attribution 4.0 International License (https://creativecommons.org/licenses/by/4.0/), which permits unrestricted use, distribution, and reproduction in any medium, provided the original author and source are credited, a link to the Creative Commons license is provided, and any changes are indicated. The Creative Commons Public Domain Dedication waiver (https://creativecommons.org/publicdomain/zero/1.0/) applies to the data made available in this article, unless otherwise stated.

987. Rebollada-Merino A, Pumarola J, Ferrer I
Altered endoplasmic reticulum stress response in non-infectious neuronal cytoplasmic inclusions in the brain of an aged dog.
Vet Res Commun 49(6):358 (2025).

988. Ferrer I
Biological Fluid Biomarkers in Human Prion Diseases with a Note on Biosafety.
Subcell Biochem 112:189-217 (2025)

989. Torres P, Pradas I, Fernàndez-Bernal A, Povedano M, Dominguez R, Jové M, Gonzalez-Mingot C, Ayala V, Ferrer I, Pamplona R, Portero-Otin M
Exploring platelet metabolomics and fatty acid profiles for ALS prognosis and diagnosis.
Sci Rep 15(1):34236 (2025).

990. Maturana-Quijada P, Chavarría-Elizondo P, Del Cerro I, Martínez-Zalacaín I, Juaneda-Seguí A, Guinea-Izquierdo A, Gascón-Bayarri J, Reñé R, Urretavizcaya M, Menchón JM, Ferrer I, Soria V, Soriano-Mas C
Effective connectivity of the locus coeruleus in patients with late-life Major Depressive Disorder or mild cognitive impairment.
Span J Psychiatry Ment Health18(3):199-207 (2025)

991. Antequera D, Vargas T, Ugalde C, Spuch C, Molina JA, Ferrer I, Bermejo-Pareja F, Carro E.Retraction notice to "Cytoplasmic gelsolin increases mitochondrial activity and reduces Aβ burden in a mouse model of Alzheimer's disease" [Neurobiology of Disease 36 (2009) 42-50].
Neurobiol Dis 215:107088 (2025)

992. Zafar S, Noor A, Younas N, Shafiq M, Dittmar K, Yagensky O, Schmitz M, Ferrer I, Hermann P, Zerr I
Proteomic Profiling Reveals Mitochondrial Dysregulation in Rapidly Progressive Alzheimer's: Role of DLDH in Amyloid Beta Aggregation.
Mol Neurobiol 63(1):73 (2025)

993. Erro ME, Zelaya MV, Eraña H, de Gordoa JSR, García-Amigot F, Simonovska-Serra A, Caballero MC, Ferrer I, Gelpi E, Jericó I, Castilla J
Variably protease-sensitive prionopathy: two new cases with motor neuron-dementia syndrome.
Ann Clin Transl Neurol 13:827–833 (2026)

994. Chu Yaiv K, Ferrer I, Söderberg-Nauder C, Religa D
Human cytomegalovirus infection and cognitive decline: Insights from population and experimental studies.
Front Aging Neurosci 17:1757461 (2026)

995. López-Sampere Y, Mengod Soler P, Roca-Pereira S, Vinyals A, Mato-Blanco X, Vela-Martínez M, Dakterzada F, Romero L, Santamaría E, Fernández-Irigoyen J, Ferrer I, Povedano M, Del Rio JA, Santpere G, Portero-Otín M, Piñol-Ripoll G, Andrés-Benito P
NRF2 deficit prevents pathologic Tau seeding and spreading in an induced tauopathy mouse model.
Redox Biol 91:104068 (2026)

996. Saleem T, Möbius W, Schmitz M, da Silva Correia A, Thomas C, Canaslan S, Hermann P, Göbel S, Zafar S, Root E, Stadelmann C, Andreoletti O, Hoppert M, Fleming Outeiro T, Ferrer I, Younas N, Zerr I
A distinct tau oligomer strain defines the molecular and proteomic landscape of rapidly progressive Alzheimer's disease.
Acta Neuropathol 151:27 (2026)

997. Costa AR, Ferreira CL, Bessa-Soares T, Duarte AC, Gonçalves I, Cavaco JE, Ferrer I, Furihata T, Marcelino H, Cascalheira JF, Santos CRA
The landscape of taste receptors in glioblastoma.
Biochem Pharmacol 250(Pt 2):118036 (2026)

Copyright: © 2026 The author(s). This is an open access article distributed under the terms of the Creative Commons Attribution 4.0 International License (https://creativecommons.org/licenses/by/4.0/), which permits unrestricted use, distribution, and reproduction in any medium, provided the original author and source are credited, a link to the Creative Commons license is provided, and any changes are indicated. The Creative Commons Public Domain Dedication waiver (https://creativecommons.org/publicdomain/zero/1.0/) applies to the data made available in this article, unless otherwise stated.

998. Romero-Murillo S, Lachén-Montes M, Cartas-Cejudo P, Anaya-Cubero E, de Miguel M, Extramiana L, Ferrer I, Fernández-Irigoyen J, Santamaría EEarly, sex-dependent and progressive proteomic imbalance during Alzheimer's disease progression. Biol Sex Differ;17(1):139 (2026)

999. Assialioui A, Povedano M, Senau M, Ferrer I, Arias L
Retinal Thickness and Vascular Density Changes in Amyotrophic Lateral Sclerosis assessed by Optical Coherence Tomography Angiography.
Biomedicines 14:1612 (2026)

1000. Blanch R, Esandi J, Guerrero-Yagüe R, del Cey J, Roig Soriano J, Lleo A, Carro E, Mestre-Francés N, Devau G, Ferrer I, Meseguer A, Lanciego JL, Almolda B, Bosch A, Chillón M
sKL/mKL mRNA ratio and protein localization define region and species-specific Klotho CNS signatures.
Genes and Disease 2026

Supplementary Books

Soriano E, JA del Rio, I Ferrer. Nonpyramidal neurons in the mammalian hippocampus: principles of organization and development. In: The neocortex. New York, London, Plenum Press, 1990, pp 185-192

Roca M, Carrió I, Prat L, Ferrer I, Mora J, Matín-Comín J. 111In labeling of human IgG using a DTPA-IgG Kit. In: radiolabeled blood elements. Recent Advances in Techniques and Applications. New York, London, Plenum Press, 1994, pp 61-65

Pineda M, Milá M, Ferrer I, Navarro c. NCL in European Countries: Spain. En: The neuronal ceroid lipofuscinoses (Batten disease). Amsterdam, Berlin, Oxford, IOS Press, 1999, pp 136

Ferrer I. Neurotrophin signalling in Alzheimer's disease: Implications in therapeutic strategies. Madrid, Cerebro y Mente, 2000, pp 304-322

Ferrer I. The neuropathological spectrum of Lewy body diseases. In: Neurodegenerative disorders associated with α-synuclein pathology. Tolosa E, Schulz JB, McKeith IG, Ferrer I (eds), Ars Medica, Barcelona, 2002, pp 73-82

Calopa M, Tolosa E, Ferrer I, Rey MJ, Cardozo A, Martí MJ, T. Ribalta. Cortical Lewy bodies in Parkinson's disease with dementia. In: Neurodegenerative disorders associated with α-synuclein pathology. Tolosa E, Schulz JB, McKeith IG, Ferrer I (eds), Ars Medica, Barcelona, 2002, pp 127-134

Berciano J, Vallderiola F, Ferrer I, Rumiá J, Pascual J, Marín C, Rey MJ, Tolosa E. Presynaptic parkinsonism in multiple system atrophy. En: Neurodegenerative disorders associated with α-synuclein pathology. Tolosa E, Schulz JB, McKeith IG, Ferrer I (eds), Ars Medica, Barcelona, 2002, pp 143-150

Ferrer I. Microcephaly. In: Developmental Neuropathology. Golden JA, Harding BN (eds), ISN Neuropath Press, Basel, 2004, pp 26-31

Ferrer I. Pathology of brain edema and brain swelling. In: Cerebrovascular diseases. Kalimo H (edit), ISN Neuropath Press, Basel 2005, pp 32-38

Ferrer I. Stress kinases involved in Alzheimer's disease, tauopathies, and APP transgenic mice. In: Gene-environment interplay in brain disease states. Palomo T, Beninger R, Kostrezwa RM, Archer T (eds), Graham Publish Co, Mountain Home, 2005, pp 519-530

Copyright: © 2026 The author(s). This is an open access article distributed under the terms of the Creative Commons Attribution 4.0 International License (https://creativecommons.org/licenses/by/4.0/), which permits unrestricted use, distribution, and reproduction in any medium, provided the original author and source are credited, a link to the Creative Commons license is provided, and any changes are indicated. The Creative Commons Public Domain Dedication waiver (https://creativecommons.org/publicdomain/zero/1.0/) applies to the data made available in this article, unless otherwise stated.

Ferrer I, Santpere G, Puig B. Immediate early genes, inducible transcription factors and stress kinases in Alzheimer's disease. In: Immediate early genes in sensory processing, cognitive performance and neurological disorders. Pinaud R, Tremere LA (eds), Springer, New York, 2006, pp. 243-260

Ferrer I, Kaste M, Kalimo H. Vascular diseases. In: Greenfield's Neuropathology. Eighth edition. Love S, Louis DN, Ellison DW (eds) Hodder Arnold, London, 2008, pp 121-240

Muñoz DG, Ferrer I. Neuropathology of hereditary forms of frontotemporal dementia and parkinsonism. In: Handbook of Clinical Neurology, vol 89: Dementias. Duyckaerts C, Litvan I (eds) Elsevier, Edinburgh, London, New York, Oxford, Philadelphia, St Louis, Sydney, Toronto, 2008, pp 393-414

Diaz M, Martin V, Fabelo N, Santpere G, Puig B, Marín R, Alonso R, Gomez T, Pérez JA, Ferrer I. Evidence for lipid alterations in lipid rafts from Alzheimer's disease human brain. In: New Trends in Alzheimer and Parkinson related disorders: ADPD 2009. Fisher A, Hanin I (eds) Medimon International Proceedings, 2009, pp 109-114

Ferrer I, Moreno D, Janué A. α-B-crystallin (HspB5) and myofribillar myopathies. In: Small stress proteins and human diseases. Simon S, Arrigo AP (eds), Nova Science Publishers, New York, 2010, pp 263-285

Naudí A, Jové M, Cacabelos D, Ramírez O, Ayala V, Ilieva EV, Kichev A, Ferrer I, Portero-Otin M, Pamplona R. Membrane unsaturation, selective neuronal vulnerability, and age-related neurodegenerative diseases. In: Lipid peroxidation: Biological implications, Catala A (edit) Transword Research Network, Keralan 2011

Naudi A, Jové M, Ayala V, Ramírez O, Cabré R, Prat J, Portero-Otín M, Ferrer I, Pamplona R. Region specific vulnerability to lipid peroxidation in the human central nervous system. In: Lipid Peroxidation" Intech open science, Rijeka, Croatia 2012

Aso E, Ferrer I. Potential therapeutic strategies to prevent the progression of Alzheimer to disease states. In: Understanding Alzheimer disease. Zerr I (edit). Intech 2013, pp 251-302.

Barrachina M, Martín M, Ciruela F, Ferrer I. Epigenetic modulation of adenosine A2A receptor: A putative therapeutical tool for the treatment of Parkinson's disease. In: Towards new therapies for Parkinson disease. Finkelstein DI (edit), Intech, 2012, pp. 295-312

Albasanz JL, Castillo CA, Barrachina M, Ferrer I, Martín M. Modulation of signal transduction pathways in senescence-accelerated mice P8 strain: a useful tool for Alzheimer's disease research. In: The clinical spectrum of Alzheimer's disease. The charge toward comprehensive diagnostic and therapeutic strategies. De la Monte S (edit). Intech, 2012, pp. 297-330

Olivé M, Ferrer I, Goldfarb LG. Disorders caused by mutant Z-disc proteins. In: Muscle Disease. Pathology and Genetics second Edition. Goebel HH, Sewry CA, Weller RO (eds). Wiley Blackwell; West Sussex 2013, pp: 163-177

Ferrer I. Brain Banking. In: Aminoff MJ and Daroff RB (eds) Encyclopedia of the Neurological Sciences, 2nd Edition, vol. 1, Oxford, Academic Press, pp. 467-473 (2014)

Ferrer I. Molecular methods. In Neuropathology of neurodegenerative diseases: a practical guide. Kovacs GG (edit), Cambridge University Press, Cambridge, 2015, pp 70-79

Ferrer I. Cerebrovascular diseases. In: Neuropathology of neurodegenerative diseases: a practical guide. Kovacs GG (edit), Cambridge University Press, Cambridge, 2015, pp 274-291

Ferrer I Neurodegeneration with brain iron accumulation. In: Neuropathology of neurodegenerative diseases: a practical guide. Kovacs GG (edit), Cambridge University Press, Cambridge, 2015, pp. 268-273

Copyright: © 2026 The author(s). This is an open access article distributed under the terms of the Creative Commons Attribution 4.0 International License (https://creativecommons.org/licenses/by/4.0/), which permits unrestricted use, distribution, and reproduction in any medium, provided the original author and source are credited, a link to the Creative Commons license is provided, and any changes are indicated. The Creative Commons Public Domain Dedication waiver (https://creativecommons.org/publicdomain/zero/1.0/) applies to the data made available in this article, unless otherwise stated.

Kovacs G, Ferrer I, Alafuzoff I. Concomitant pathologies II: Neurodegenerative conditions. In: Neuropathology of neurodegenerative diseases: a practical guide. Kovacs GG (edit), Cambridge University Press, Cambridge, 2015, pp. 292-298

Kalaria, Ferrer I, Lowe S. Chapter 2: Vascular disease, hypoxia and related conditions. In Greenfield's Neuropathology 9th Edition. Love S, Budka H, Ironside JW, Perry A (eds). CRC Press, Boca Raton, Florida, 2015 pp: 59-209

Ferrer I. Neuropathology and neurochemistry of nonmotor symptoms in Parkinson's disease. In: Top five contributions in Alzheimer and Parkinson disease. Ebooks www.avidscience.com, 2019

Pamplona R, Jove M, Pradas I, Mota-Martorell N, Ferrer I. Lipoxidation: features, neurological tissues, and aging. In: Factors affecting neurological aging; genetics, neurology, behaviour, and diet. Martin CR, Preedy VR, Rajendram R (eds) Academic Press, Elsevier, 2021, pp: 83-96

Ferrer I. Neurodegeneración. Aspectos generales. En: Neurologia, Zarranz JJ, 7 edicion. Elsevier 2024, pp: 801-804

Kalaria R, Englund E, Ferrer I. Cerebrovascular disorders. In: Greenfield's Neuropathology 10th edition, Editors: Smith C, Perry A, Kovacs G, Jacques T, CRC Press, Taylor and Francis Group, Boca Ratón, FL, 2025, pp: 75-246

Kovacs GG, Ferrer I. Neurodegenerative diseases: classification. In: Greenfield's Neuropathology 10th edition, Editors: Smith C, Perry A, Kovacs G, Jacques T, CRC Press, Taylor and Francis Group, Boca Ratón, FL, 2025, pp: 803-817.

Kovacs GG, Ferrer I. Diagnostic approach to the neuropathology of neurodegenerative diseases. In: Greenfield's Neuropathology 10th edition, Editors: Smith C, Perry A, Kovacs G, Jacques T, CRC Press, Taylor and Francis Group, Boca Ratón, FL, 2025, pp: 1226-1236

Ferrer I, Houlden H. Miscellaneous proteinopathies and neurodegenerative conditions. In: Greenfield's Neuropathology 10th edition, Editors: Smith C, Perry A, Kovacs G, Jacques T, CRC Press, Taylor and Francis Group, Boca Ratón, FL, 2025, pp: 1183-2000

Gazulla J, Ferrer I, Berciano J. The Cerebellum in Degenerative Motor Neuron Disease - an Example of Complexity of Neurodegenerative Disorders. In: Models in Neurodegenerative diseases, Editors Cendelin J, Tuma J, Manto M. Springer Nature, 2026, pp19-38

Copyright: © 2026 The author(s). This is an open access article distributed under the terms of the Creative Commons Attribution 4.0 International License (https://creativecommons.org/licenses/by/4.0/), which permits unrestricted use, distribution, and reproduction in any medium, provided the original author and source are credited, a link to the Creative Commons license is provided, and any changes are indicated. The Creative Commons Public Domain Dedication waiver (https://creativecommons.org/publicdomain/zero/1.0/) applies to the data made available in this article, unless otherwise stated.
